# Transcriptional analyses of peripheral blood lymphocytes obtained shortly after primary immunization discriminate vaccines against *Francisella tularensis*

**DOI:** 10.1128/iai.00208-26

**Published:** 2026-08-14

**Authors:** Roberto De Pascalis, Scott Espich, Hamda Khan, Mykia Toney, Chou Chao-Kai, Wu Wells, Karen L. Elkins

**Affiliations:** 1 Laboratory of Mucosal Pathogens and Cellular Immunology, Division of Bacterial, Parasitic and Allergenic Products, Center for Biologics Evaluation and Research, Food and Drug Administration 67149, Rockville, Maryland, USA; 2 Office of Director, Science Staff, Center for Biologics Evaluation and Research, Food and Drug Administration, Rockville, Maryland, USA; Helmholtz-Zentrum fur Infektionsforschung GmbH, Braunschweig, Germany

**Keywords:** *Francisella tularensis*, vaccines, primary vaccination, immune responses

## Abstract

The high virulence and pathogenesis of *Francisella tularensis* (*Ft*), responsible for tularemia, made it a target for bioweapon development during the “Cold War.” This prompted investigation of vaccines, starting with an older vaccine candidate denoted *Ft* live vaccine strain (LVS). LVS has limited efficacy against aerosol challenge with *Ft* subsp. Tularensis (Type A), the most virulent subtype. However, no vaccines, including LVS, have been licensed in the U.S. Animal studies indicate that *Ft* vaccines induce protective T-cell-mediated immune responses. In previous studies, analyses of leukocytes from vaccinated animals that were restimulated *in vitro* facilitated screening and selection of vaccines for more extensive *in vivo* evaluation. These studies demonstrated that a novel live attenuated vaccine strain derived from Type A *Ft*, denoted *ΔclpB*, was protective against aerosol challenge in animal models. In this study, we focused on evaluating immune responses immediately after vaccination of Fischer 344 rats with LVS and *ΔclpB* using multiple analytical approaches. While humoral immune responses were comparable between the two vaccines, we identified multiple differences in immune responses by analyses of peripheral blood leukocytes by flow cytometry and by relative gene expression. In particular, vaccination with LVS or *ΔclpB* resulted in differential expression of multiple genes at selected time points. Gene expression, in turn, predicted activation or inhibition of multiple biological pathways. These results suggest that immune responses shortly after primary vaccination may discriminate vaccines with different degrees of *in vivo* protection that could potentially be used as correlates of protection and extrapolate efficacy from animals to people.

## INTRODUCTION

*Francisella tularensis (Ft),* including subspecies tularensis (*Ftt*) and subspecies holarctica (*Fth*), causes the disease tularemia, an infection that has low incidence in the United States and is not a current U.S. public health concern (1). However, *Ftt*’s low infectious dose, its ability to be aerosolized, and its virulence made it attractive for bioweapon development (2). In the United States, *Ft* is currently classified as a Tier 1 bioterrorism Select Agent (https://www.selectagents.gov/sat/list.htm). The live vaccine strain (LVS), derived from the *Fth* in the 1950s (3–6), is the only vaccine under clinical investigation in the United States. However, LVS, which is administered by scarification, offered only partial protection against aerosol *Ftt* exposure during small human challenge studies (4, 7–9), and LVS remains unlicensed. Following the 2001 terrorist attacks, interest in a new vaccine against tularemia was renewed, and multiple novel vaccine candidates have been investigated (10, 11). At the same time, a novel regulatory pathway, known as the “Animal Rule,” was established by the FDA (12–14) to facilitate the approval of medical products when efficacy clinical trials are not feasible (9). The “Animal Rule” requires well-characterized animal models for product testing, ideally two species, as well as a scientifically valid means to bridge between estimates of efficacy from animals to people. Bridging would be greatly facilitated by the identification of correlates of protection.

Cynomolgus macaques have been qualified to test drug treatment of tularemia and have been used to evaluate vaccine-induced protection as well as immune responses against tularemia (15). However, the cost and limited supply of non-human primates (NHPs) make this model most appropriate for second-stage studies after evaluations in small animals. BALB/c and C57BL/6 mice have been the animal models most utilized for evaluating tularemia vaccine efficacy (16). However, their limitations in representing human immune responses (17) and in evaluating vaccine efficacy (18) prompted us and others to explore additional animal models (19, 20). Fischer 344 rats better reflect outcomes of *Ftt*, *Fth*, and *F. novicida* (*Fn*) infections in people and have proven useful in evaluating immune responses as well as protection. Using both mice and rats, we have taken advantage of an *in vitro* co-culture method where *Francisella*-infected macrophages are used to measure the ability of leukocytes, particularly T lymphocytes, to control intramacrophage bacterial growth (19, 21–24). These studies used lymphocytes derived from rodents administered with different vaccines and obtained a month or more after vaccination, representing adaptive memory immune responses. Using transcriptional analyses of leukocytes recovered from such co-cultures, we identified a working panel of correlates of vaccine-induced protection. We then applied this approach to screen multiple vaccines derived from *Fth* (23, 25), *Fn* (26), and *Ftt* (24) to predict vaccine efficacy. Our results indicated that two vaccines, SchuS4-*ΔclpB* (*ΔclpB*) and SchuS4-*ΔclpB-*Δ*capB,* should be used for more extensive studies (24). Subsequent *in vivo* vaccination and challenge studies in Fischer rats and in NHPs demonstrated substantial efficacy for these novel vaccines (27).

Other studies discovered genes that were differentially regulated in peripheral blood leukocytes (PBLs) and splenocytes from LVS-vaccinated C57BL/6J mice within 2 days–2 weeks after vaccination, without *ex vivo* restimulation provided by co-cultures (23). Similarly, in PBLs and splenocytes from Fischer rats, several genes of immunologic interest discriminated LVS from the sub-optimal vaccine LVS-R (19), and multiple genes were differentially regulated in PBLs from Fischer rats 2 weeks after LVS and *ΔclpB* vaccination (28). These results suggested that relative gene expression in blood cells obtained shortly after primary vaccination may also discriminate vaccines and predict *in vivo* protection outcomes.

Because both LVS and *ΔclpB* provided substantial *in vivo* protection against aerosol *Ftt* challenge in rats (24, 27, 28) and in cynomolgus macaques (27), in this study we used multiple analytical approaches to perform an extensive evaluation of immune responses at different time points after LVS and *ΔclpB* vaccination in Fischer rats. As expected, based on previous results (24), humoral immune responses were comparable between the two vaccines. Here, we identified multiple differences between the two vaccinated groups by flow cytometry and by transcriptional analyses of PBLs. These differences are now poised to be tested directly as predictive correlates.

## MATERIALS AND METHODS

### Experimental animals

Five-to-seven-week-old female Fischer rats, strain F344/NHsd, were purchased from Envigo (Indianapolis, IN) and age-matched within each experiment. Animals were housed two per cage in sterile cages with HEPA-filtered circulating air at the Division of Veterinary Services, CBER/FDA, and fed *ad libitum* with irradiated food and autoclaved chlorine dioxide-treated water. The room was maintained at negative pressure with temperature and humidity control as well as a 12-hour light on/off cycle.

### Bacteria and growth conditions

LVS (ATCC 29684) and SchuS4-*ΔclpB,* derived from *Ftt* (obtained from Dr. Conlan [10]), were grown to mid-log phase in modified Mueller-Hinton (MH) broth (Difco Laboratories, Detroit, MI), harvested, and frozen in aliquots in broth alone at −80°C (29, 30). Bacteria were periodically thawed for quality control by quantification of viability and colony-forming units (CFU) on MH agar plates.

### Bacterial immunizations

Rats were anesthetized with isoflurane and immunized by s.c. administration with 5 × 10^6^ CFU for each vaccine, diluted in 0.1 mL phosphate-buffered saline (PBS; BioWhittaker/Lonza, Walkersville, MD); control groups received 0.1 mL PBS. Actual doses of administered vaccines were determined by retrospective plate counts.

### Serum collection and PBL preparation

On days 3, 7, 10, 17, and 24 after immunization, rats were sacrificed, blood was collected by cardiac puncture, and spleens were harvested. Approximately 0.5–1 mL of blood was transferred into Z-Gel (Sarstedt, Germany) microtubes and centrifuged at 5,000 × *g* for 5′ at RT. The resulting sera were transferred into fresh microcentrifuge tubes and stored at −80°C until use. Alternatively, blood was collected in heparin and used to prepare PBLs, which were used either immediately for flow cytometry analyses or stored in RNA*later* (Ambion, Austin, TX) at −80°C for further gene expression analyses. Splenocytes were prepared as per standard protocol and used for flow cytometry analyses or stored in RNA*later*. In addition, aliquots of blood and of disrupted spleens were tested in MH plates for organ burden quantification.

### Evaluation of serum antibodies

Anti-*Francisella* rat serum antibody levels were evaluated by a modified ELISA as previously described (24). Briefly, vinyl flat-bottom ELISA plates (Thermo Fisher Scientific, Waltham, MA) were coated with 5 × 10^6^ CFU/well of LVS or *ΔclpB* in 100 μL of coating buffer (32 mM Na_2_CO_3_, 68 mM NaHCO_3_, pH 9.6), incubated for 2 hours at 37°C, 5% CO_2_, then overnight at 4°C. The plates were blocked with 200 μL of blocking solution (PBS with 10% liquid gelatin; Norland Products; Cranbury, NJ) for 1.5 hours at 37°C, 5% CO_2_. Serum samples and reference standards were serially diluted from 1:200 to 1:25,600 in blocking solution, and then 100 μL was added to each well for 1.5 hours at 37°C, 5% CO_2_. The reference standards, constituted of pooled immune sera from three rats that were collected 14 days after either one immunization for IgM or three immunizations for IgG, were used for quality control of the assay to ensure consistency of results. Bound anti-LVS or anti-*ΔclpB* antibodies were detected using 100 μL of goat anti-rat IgM or IgG antibodies, respectively, conjugated with horseradish peroxidase (Southern Biotech, Birmingham, AL) in blocking solution for 1 hour at room temperature. The plates were washed with PBS with 0.85% NaCl and 0.05% Tween-20 between each step. The plates were then developed using a solution of ABTS (KPL, Thermo Fisher Scientific), and the reaction was stopped with ABTS HRP Stop Solution (KPL). The optical density (OD) at 405 nm was read using a microplate reader (Synergy H1, Agilent) and analyzed using BioTek Gen5 Software (Agilent). Blank OD values, generated by developing wells that included all reagents without serum samples, were subtracted from sample values.

### Flow cytometry analyses

Single-cell suspensions were prepared from total splenocytes and PBLs. Cells were incubated with anti-CD32 Fc block (clone D34-485, BD Pharmingen, San Diego, CA) and stained with Live/Dead staining kit (Invitrogen, Carlsbad, CA). Cells were then washed in flow cytometry buffer (PBS with 2% FBS) and stained for cell surface markers. Antibody concentrations were previously optimized for use in multi-color staining protocols as required, using appropriate fluorochrome-labeled isotype-matched control antibodies. The following antibodies and clones were used: anti-CD161 (3.2.3), anti-CD4 (OX-35), anti-CD45R (HIS24), anti-MHCII (OX-17), anti-CD3 (G4.18), anti-CD8a (OX-8), anti-CD11b/c (OX-42), anti-granulocytes (RP-1), anti-CD45 (OX-1), anti-TCRβ (R73), and anti-CD45RA (OX-33). All antibodies were purchased from BD Biosciences. In all, 10,000 CD45^+^, live, singlet events were collected using an analytical Cytek Aurora flow cytometer (Cytek Biosciences, Fremont, CA). Data analyses were performed using FlowJo (Tree Star, Inc.) software.

### Gene expression analyses

PBLs stored in RNA*later* at −80°C were used to purify total RNA using RNeasy mini kits (Qiagen, Valencia, CA). The RNA was treated with DNase (Invitrogen, Thermo Fisher Scientific) followed by a clean-up step (RNAeasy). Total RNA quality was assessed using a 2100 Bioanalyzer (Agilent Technologies, Santa Clara, CA), and all samples showed RNA integrity numbers (RIN) of approximately 7.5 or higher. For real-time PCR analyses, up to 1 μg of RNA was used to synthesize cDNA (High-Capacity RNA-to-cDNA; Applied Biosystems, Carlsbad, CA). All reactions were performed as per manufacturer’s instructions. Semi-quantitative real-time PCR amplification of a panel of 19 genes of immunologic interest plus Gusb and RPS29, as housekeeping genes, all from Applied Biosystems, was completed with a ViiA 7 Real-Time PCR system (Applied Biosystems). After normalization, delta Ct (ΔCt) and the ratio between ΔCt of vaccine samples and naïve control samples were calculated (ΔΔCt), resulting in a semi-quantitative analysis (relative to naive control) of gene expression.

Poly(A)-enriched libraries were prepared using the Illumina Stranded mRNA Prep Kit (Illumina, San Diego, CA). Briefly, 200 ng of total RNA was poly(A)-selected, fragmented, and reverse-transcribed to generate cDNA. Double-stranded cDNAs were end-repaired, 3′-adenylated, indexed, and amplified with 13 cycles of PCR. Library quality and quantity were evaluated using the 2100 Bioanalyzer and the Qubit 4 Fluorometer (Invitrogen, Carlsbad, CA). Normalized libraries were sequenced on a NovaSeq 6000 System (Illumina, San Diego, CA) using paired-end 2 × 100 bp chemistry. Base call files were converted to FASTQ format using the bcl2fastq conversion software (Illumina). FastQC analysis confirmed high-quality sequencing reads. Downstream processing was performed using CLC Genomics Workbench v25 (Qiagen, Valencia, CA). Reads were aligned to the rat reference genome (GRCr8), and gene expression levels were quantified using the software’s RNA-seq analysis workflow.

### Statistical analyses

Microsoft Excel and GraphPad software were used to evaluate differences in bacterial growth, relative gene expression, and antibody titers. CFU data were log_10_ transformed and were measured using a log scale. Significant differences were evaluated using a two-tailed Student’s *t* test, with Welch’s modification and with a *P* value of < 0.05 indicating significance. For RNA-seq analyses, differential gene expression between vaccinated samples and controls was assessed using CLC Genomics Workbench v25 (Qiagen). Genes with an absolute fold change >1.5 and an adjusted *P* value (FDR) < 0.05 were considered significantly differentially regulated and were used for generating volcano plots and Venn diagrams. Pathway enrichment analysis was performed using Ingenuity Pathway Analysis (IPA, Qiagen). Pathway *P* values and activation z-scores were calculated based on the set of significantly regulated genes for each treatment condition.

## RESULTS

### Organ burden after vaccination

**Fig 1:**
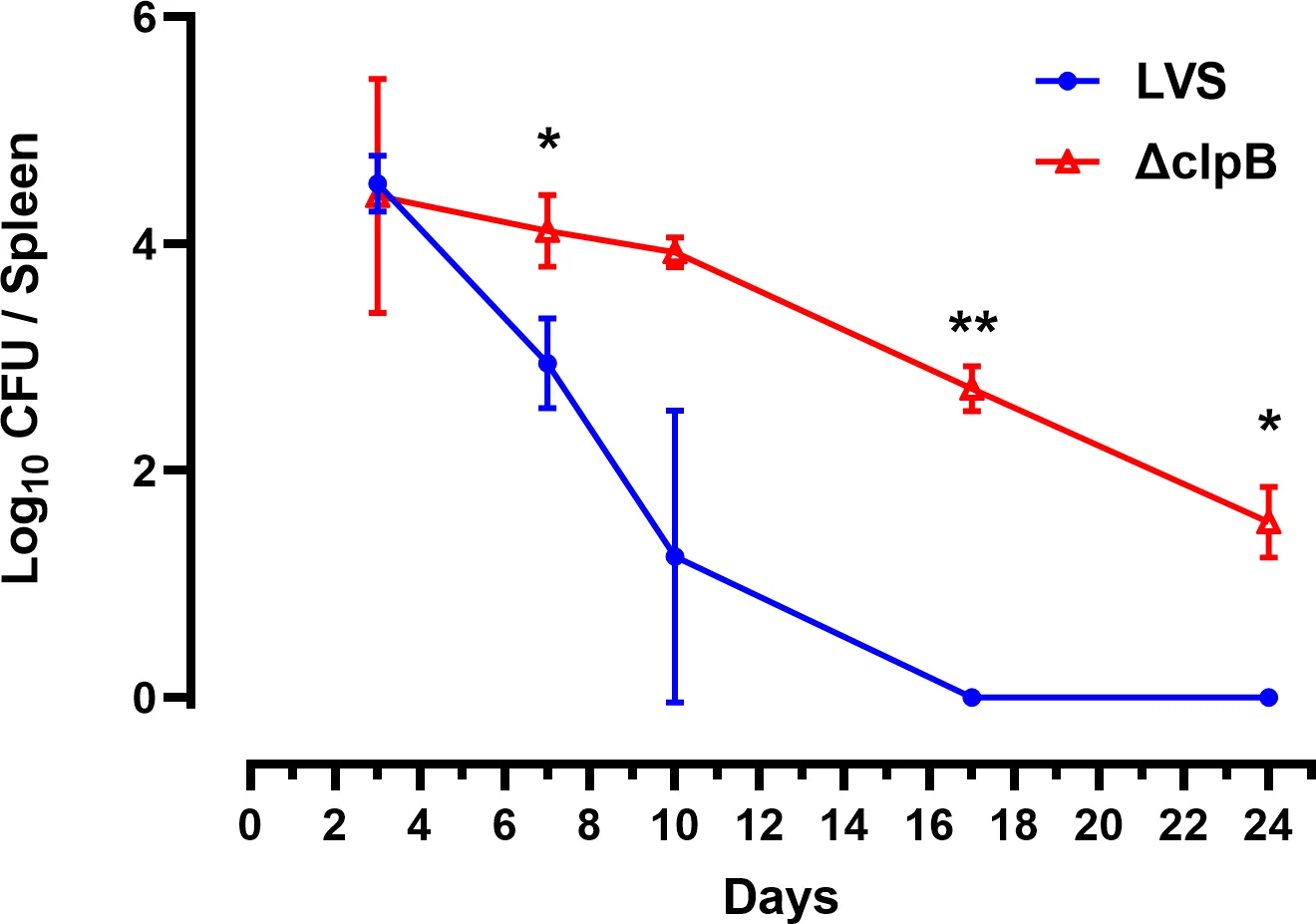
LVS is cleared faster than *ΔclpB* in spleens. Fischer 344 rats were vaccinated as described in Materials and Methods. At the indicated time points after vaccination, spleens were harvested and manually disrupted, and selected volumes were plated on MH plates. Values indicate mean and standard deviation derived from three animals per group. * and ** indicate significant differences, *P* < 0.05 and <0.005, respectively, as calculated by *t* test with Welch’s correction.

To evaluate clearance in detail of these live attenuated vaccines from organs, spleens were harvested, manually disrupted, and plated on MH plates for bacterial quantification. No substantial difference in cell yields between spleens from PBS and vaccine groups at different time points was observed. Three days after vaccination, LVS and *ΔclpB* CFUs recovered from the spleens were approximately 4.5 log_10_, although we noted considerable animal-to-animal variability (Fig. 1). Starting from day 7 after vaccination, LVS CFU numbers declined faster than those of *ΔclpB*, and by day 17, LVS was no longer detectable and presumably cleared from the spleens. Although at low amounts, *ΔclpB* persisted up to day 24 after vaccination. At days 7, 17, and 24, the differences between LVS and *ΔclpB* bacterial counts were significant. A similar quantification was performed on blood 3 days after vaccination. Consistent with previous studies, bacterial counts were less than 10 bacteria/mL and detectable in only a few animals.

### Serum antibody responses in vaccinated rats

**Fig 2:**
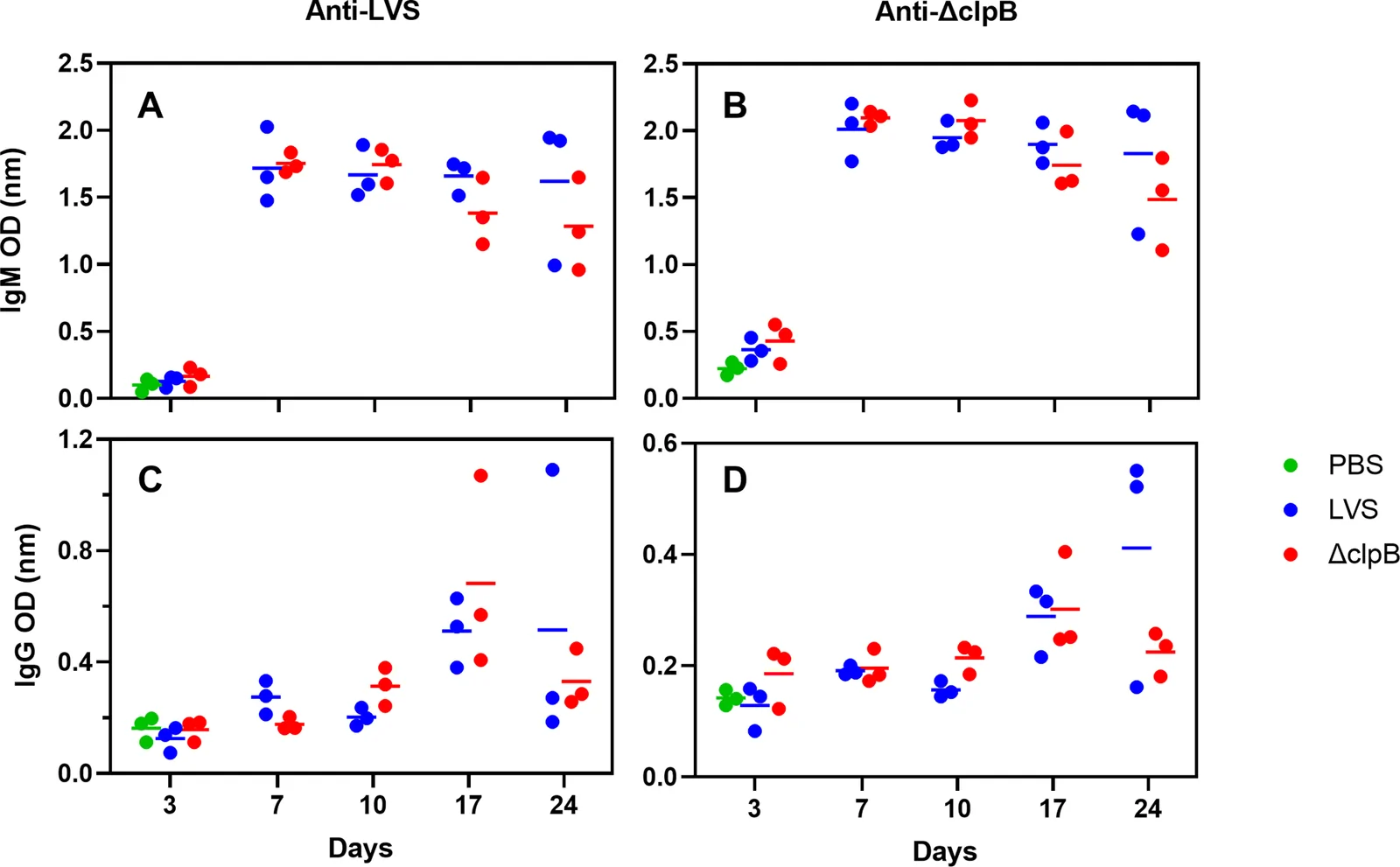
Serum anti-*Francisella* antibody titers from vaccinated rats show minimal differences between vaccine groups. At the indicated time points after vaccination, blood was collected, serum samples were prepared, and sera were analyzed for anti-LVS (**A and C**), anti-*ΔclpB* (**B and D**), IgM (**A and B**), and IgG (**C and D**) antibodies. Shown are the results from individual animals and the mean values obtained from three rats per group. At each time point, the differences between values from the two vaccine groups were calculated by *t* test with Welch’s correction; no differences were significant.

To evaluate humoral immune responses after vaccination, Fischer rats, immunized with LVS or *ΔclpB* or injected with PBS, were bled at selected time points, and sera from three individual animals per group were obtained. Initially, serial dilutions of sera and standards were analyzed to identify a single informative target dilution, which was then used to determine the relative quantities of IgM and IgG antibodies. Anti-LVS and anti-*ΔclpB* IgM antibodies were readily detectable in all vaccinated rats 7 days after vaccination, and levels started declining 17–24 days after vaccination (Fig. 2A and B). In contrast, IgG antibodies against LVS and *ΔclpB* reached a peak between days 17 and 24 (Fig. 2C and D). We noted substantial animal-to-animal variability, particularly at days 17 and 24, in both vaccine groups. Despite variability, there were no significant differences in IgG antibody levels between sera from LVS- and *ΔclpB*-vaccinated rats at any time point.

### Flow cytometry

**Fig 3:**
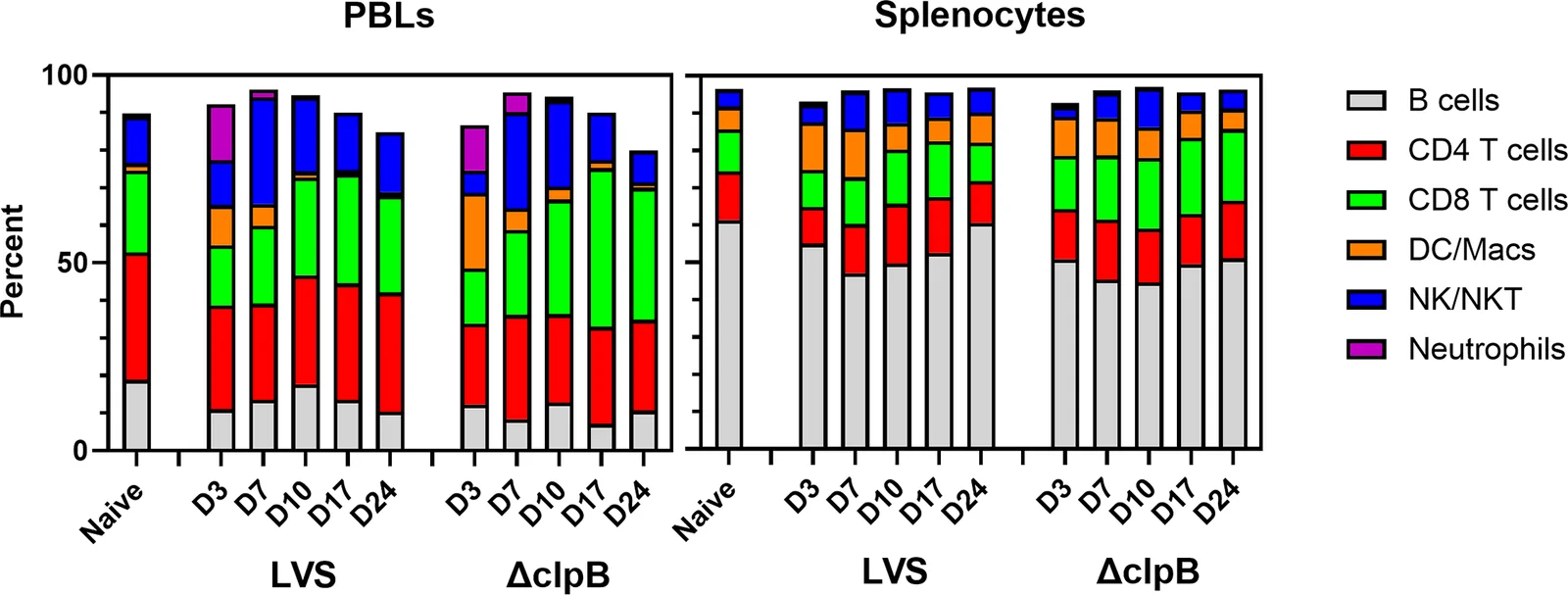
Cell type sub-populations change over time after vaccination. At the indicated time points after vaccination, three animals per group were sacrificed, blood was collected, spleens were harvested, and PBLs and splenocytes were prepared for analyses by multi-parameter flow cytometry. Leukocytes were gated as indicated in Fig. S1. After removal of fragments and aggregates, live CD45^+^ leukocytes were gated to identify B cells (CD45R^+^ CD45RA^+^), CD4 (CD3^+^ TCRαβ^+^ CD4^+^ CD8a^−^), CD8 T (CD3^+^ TCRαβ^+^ CD8a^+^ CD4^−^) cells, dendritic cells/macrophages (MHC II+CD161^+/−^), NK/NK T cells (MHC II^−^ CD161^+^), and neutrophils (MHC II^−^ CD161^−^ CD11b/c^+^ RP1^+^).

To evaluate cell populations after vaccination, PBLs and splenocytes from naïve and vaccinated rats were stained for major immune cell surface markers, analyzed by flow cytometry, and evaluated using a gating strategy as shown in Fig. S1. Numbers of PBLs and splenocytes from PBS and vaccine groups were comparable across all time points. Compared to naïve animals, B-cell proportions in both PBLs and splenocytes from LVS- and *ΔclpB*-vaccinated rats exhibited a relatively slight reduction at all time points (Fig. 3; Table S1). T-cell proportions, after initial relative reductions compared to non-B/T cells, increased over time after vaccination, particularly in PBLs. Among T cells, the proportion of CD4 T cells was higher at early phases after vaccination, while CD8 T cells were higher at late time points. Although this pattern was observed after either immunization, CD8a^+^ T-cell proportions trended higher after *ΔclpB* than after LVS vaccinations in both PBLs and splenocytes. Among non-B/T cells, DCs, monocytes, macrophages, and neutrophils increased at day 3, while NK and NKT cells peaked on day 7 after vaccination; thereafter, all progressively decreased. In addition, although not significantly different, we observed a few trends between the two vaccine groups. For example, the proportions of DCs and monocytes were higher on day 3 in PBLs from rats vaccinated with *ΔclpB*, while NK cell proportions were higher on day 7 in animals vaccinated with LVS.

### Real-time PCR

A panel of immune-related genes was used to evaluate their relative expression in PBLs, which was based on outcomes from previous studies of similar design using both mice and rats (19, 23, 24). However, for comparison, we also included genes that typically showed minimal differential expression but that potentially are involved in T-cell immune responses. The ratio of the values obtained versus those of the naive group indicated different patterns of expression after vaccination (Fig. S2). Some genes, such as IL-18bp and Nos2, appeared to be upregulated at early time points after vaccination. The expression of other genes, such as Tbet and CCL5, was higher across multiple time points. Interestingly, IFN-γ, a major mediator during both innate responses and adaptive T-cell responses, exhibited bi-phasic expression in PBLs from *ΔclpB*-vaccinated rats, with upregulation at both early and late time points but interspersed with decreased expression. PRF1 exhibited minimal regulation, while IL-2ra was the only gene downregulated across all time points. Several genes exhibited more upregulation in PBLs from *ΔclpB*-vaccinated rats compared to those from LVS-vaccinated animals across all time points, and in a few cases the differences were significant.

### RNA-Seq

**Fig 4:**
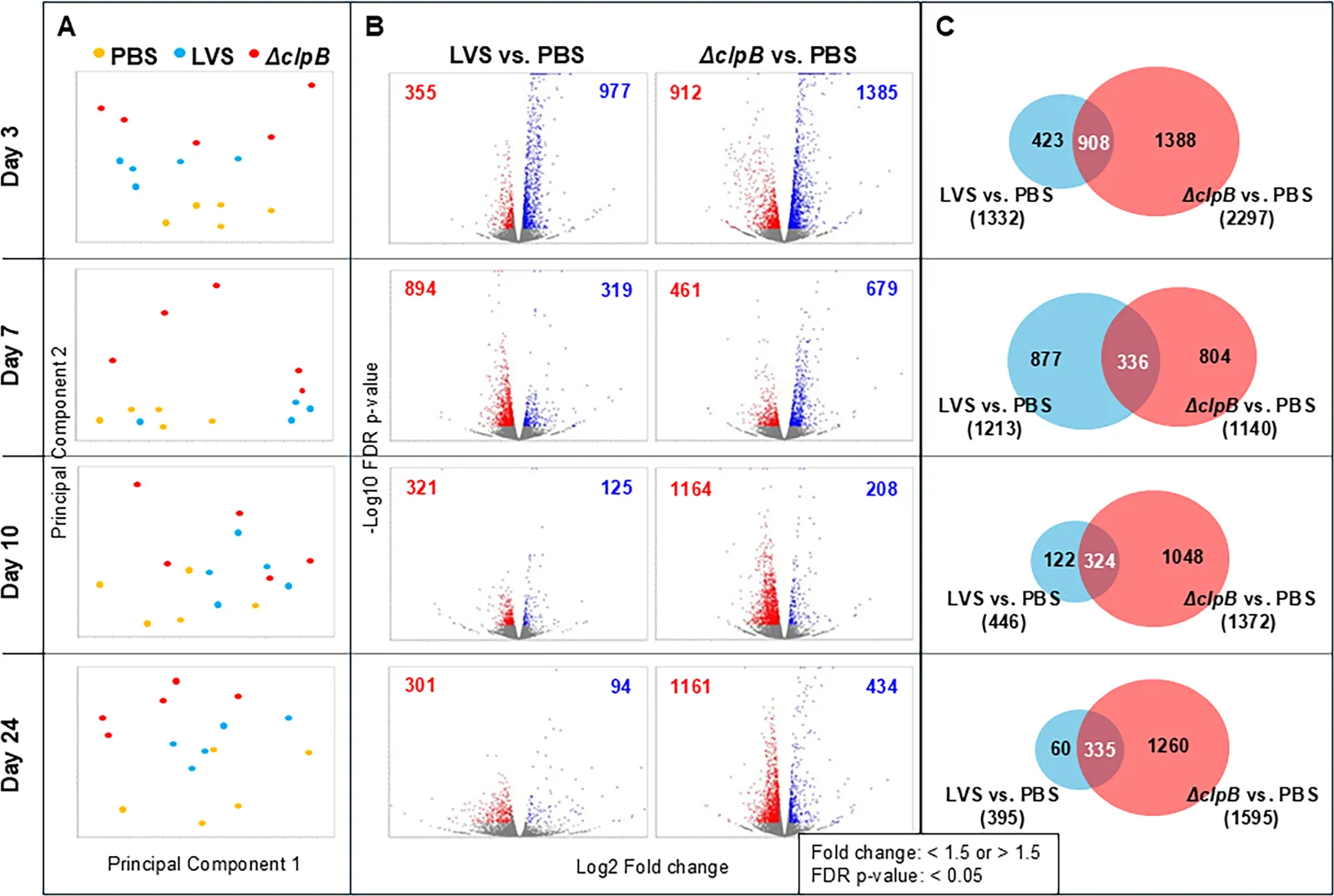
Relative gene expression of PBLs exhibits differences between vaccine groups by RNA-seq analyses. Total RNA from PBLs of vaccinated Fisher rats, prepared as described in Materials and Methods, was used to evaluate gene expression by RNA-seq analysis. The results were initially assessed by PCA (**A**) and then filtered by fold change and FDR *P* value to quantify upregulated (in blue) and downregulated (in red) genes (**B**) and to quantify the total number of genes that were both upregulated and downregulated after LVS and *ΔclpB* vaccination (**C**). In parentheses, for each vaccine, the total numbers of differentially regulated genes (both upregulated and downregulated) are indicated. This strategy assessed the overlap between the two vaccines.

Because qRT-PCR results were encouraging, aliquots of the RNA sample used for real-time PCR were sequenced for RNA-seq evaluation. The results from RNA-seq were initially evaluated by unbiased principal component analysis (PCA) using all data at different time points (Fig. 4A). The PCA results showed that the PBS samples grouped together, and on days 3 and 7 both vaccine groups were well distanced from the PBS group. In contrast, vaccinated samples from day 10 and day 24 were closer to the PBS group, suggesting a return to baseline. Although we observed animal-to-animal variability, within each vaccine group most of the samples grouped together. This was most noticeable on days 3 and 24, suggesting detectable differences between vaccines.

We then calculated the average of the five replicates for each group at each time point and calculated the ratio of each vaccine versus PBS. Finally, we filtered the results by a 1.5-fold difference and by an FDR *P* value of <0.05 (Table S2) and represented the results using volcano plots (Fig. 4B). A total of 4,494 genes were either upregulated or downregulated at one or multiple time points. Consistent with observations in the PCA plot (Fig. 4A), the early responses (day 3) to *ΔclpB* vaccination showed significantly more differentially regulated genes compared to other conditions (Fig. 4B). Notably, expression of more than half of these genes was uniquely affected by *ΔclpB* vaccination (Fig. 4C), suggesting that *ΔclpB* induced stronger responses compared to LVS. Although the number of differentially regulated genes following *ΔclpB* vaccination decreased after day 7, steady levels of gene regulation were then maintained throughout the study period and persisted longer compared to LVS vaccination (Fig. 4C). In contrast, fewer genes were affected by LVS vaccination. Except for increased downregulation on day 7, LVS triggered substantially fewer changes in gene expression compared to *ΔclpB* vaccination. Moreover, at each time point, at least 30% of genes that were differentially regulated overlapped between the two vaccine groups (Fig. 4C; Table S2). Taken together, with the exception of day 7, the number of genes that were differentially regulated was higher after *ΔclpB* than LVS vaccination. Furthermore, except for four genes (IL-21, IL-2RA, CCR3, and LTA), the genes initially quantified by real-time PCR (Fig. S2) showed an overall expression pattern that was consistent with the RNA-seq results (Table S2).

### Ingenuity pathway analysis

**Fig 5:**
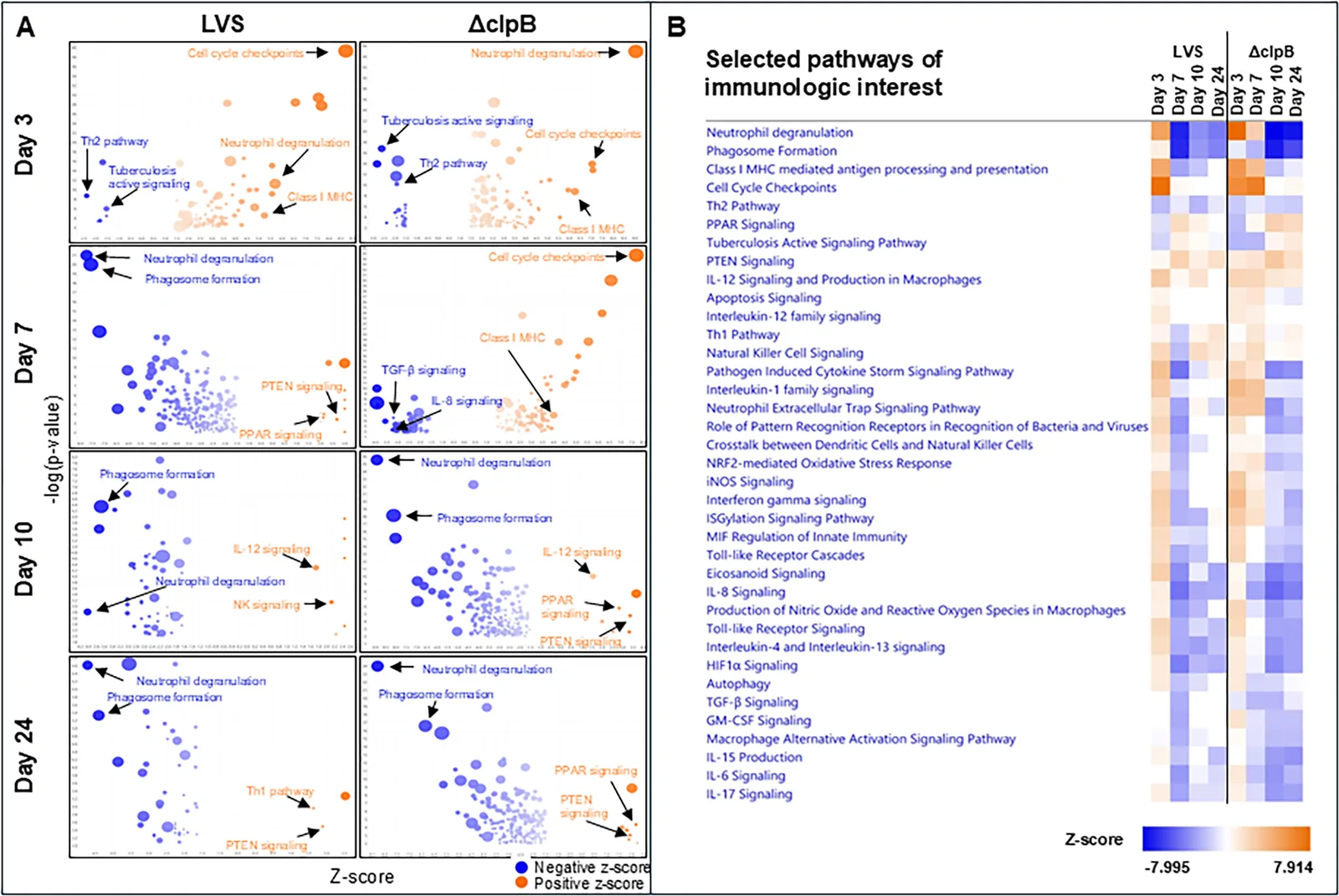
Ingenuity Pathway Analyses indicate different activation patterns between vaccine groups over time. Results from RNA-seq analyses, as described in Fig. 4 and Table S2, were used to identify canonical pathways that may be activated (in orange) or inhibited (in blue) after vaccination (**A**). Results were filtered by the −log *P* value > 2 and by the z-score < 1.5 or >1.5. The size of circles reflects the number of genes that overlap the pathway. For each time point and vaccine group, the 2–3 pathways of immunologic interest with the highest (activated) or lowest (inhibited) z-score are indicated. Pathways of immunologic interest were selected and sorted by z-score (**B**), while the entire list of pathways is described in Table S3.

The results from RNA-seq analyses were used to evaluate the canonical pathways that could be modulated following vaccinations by using IPA software. Differentially regulated canonical pathway results were filtered by −log *P* value > 2 and z-score <1.5 and >1.5 (Fig. 5A; Table S3). On day 3, most pathways were activated by either vaccination, but the pathway contents differed substantially between the two vaccinations. The most significantly elevated pathway following *ΔclpB* treatment was the neutrophil degranulation pathway, whereas the cell cycle checkpoints pathway showed the highest upregulation following LVS vaccination (Fig. 5A and B). Notably, the cell cycle checkpoints pathway became the most upregulated pathway following *ΔclpB* treatment by day 7. Meanwhile, the neutrophil degranulation pathway was dramatically downregulated on day 7 in the LVS group. Downregulation of the neutrophil degranulation pathway was observed by days 10 and 24 for both treatments. These results suggest that *ΔclpB* vaccination may trigger stronger and more prolonged early immune responses compared to LVS vaccination. Overall, the results indicate that the number of predicted pathways or functions, whether activated or inhibited, was greater following *ΔclpB* vaccination than after LVS vaccination.

### Working gene panel

**Fig 6:**
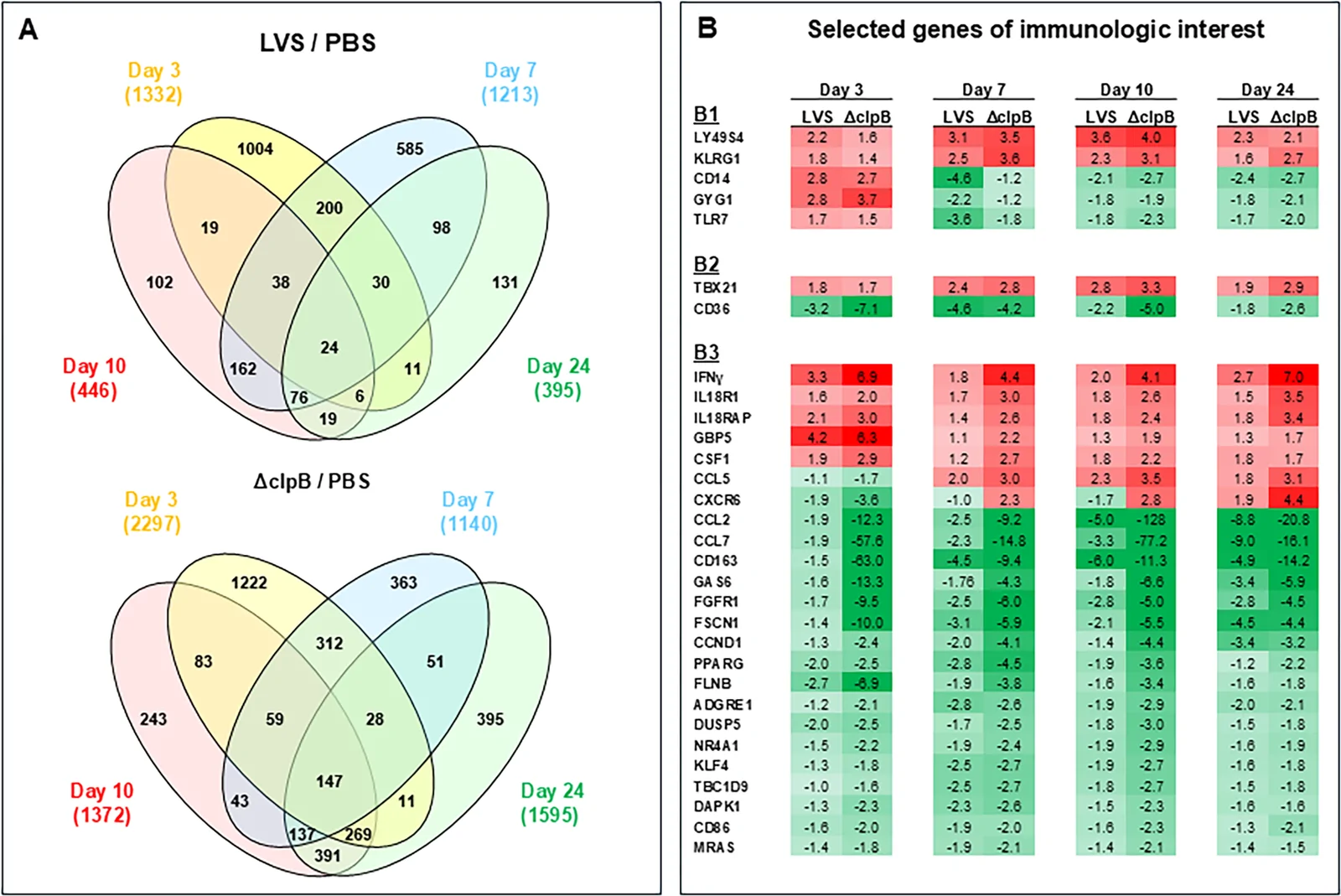
Genes of immunologic interest discriminate vaccine groups. Differentially regulated genes, as described in Fig. 4 and Table S2, were analyzed to identify features that overlap over time after LVS or *ΔclpB* vaccination (**A **and Table S4). Among these genes, several of immunologic interest (**B**) were associated with pathways described in Fig. 5B. Data indicate fold differences, showing upregulated (in red) or downregulated (in green) genes compared to PBS. For completeness, data with fold differences of <1.5 or >−1.5 were also included.

To identify a working panel of genes capable of discriminating the immune responses elicited by the two vaccines, we first focused on pathways with known roles in immune activation (Fig. 5B). These pathways suggested distinct temporal, quantitative, and potentially qualitative differences between vaccine groups. We then assessed the overlap in differential gene regulation across time points after each immunization (Fig. 6A). Notably, *ΔclpB* vaccination induced far broader transcriptional responses. We identified 147 differentially expressed genes after *ΔclpB* vaccination across all time points, compared with only 24 genes following LVS vaccination. Just six genes were shared between the two groups (Table S4). From these differentially expressed genes, we further narrowed our selection to those involved in the immune-related pathways identified in Fig. 5B (Fig. 6B). Within the LVS-associated genes (Fig. 6B [B1 set]), clear divergence between the two vaccine responses was apparent only at Day 7. In contrast, the genes associated with *ΔclpB* vaccination (Fig. 6B [B3 set]), as well as the small set common to both vaccines (Fig. 6B [B2 set]), exhibited consistently stronger upregulation or downregulation in *ΔclpB*-vaccinated rats across multiple time points. Collectively, therefore, these analyses identified candidate genes whose relative expression may be used to both discriminate vaccines with subtle differences in efficacy and to apply as predictive correlates.

## DISCUSSION

Due to the low incidence of tularemia in the United States and elsewhere, traditional clinical field trials likely cannot be used to assess vaccine efficacy. Consequently, characterization of vaccine-induced immune responses may represent the only practical approach to infer protection. However, no human immune correlates of protection against tularemia, or any intracellular pathogen, have yet been defined in people. To address these issues, animal models can help evaluate vaccine protection against aerosolized *F. tularensis*, identify immune markers associated with protection, and ultimately support translational bridging from animals to humans. The identification of common differentially expressed genes and immune pathways in tuberculosis models of nonhuman primates, Diversity Outbred mice, and humans (31) further supports the use of animal models to evaluate immune responses to vaccines against intracellular pathogens.

Previously, we established methods to evaluate adaptive immune responses in PBLs from vaccinated rodents (19, 23). Although additional studies are still required to obtain a comprehensive picture of the adaptive immunity against *Ft*, the primary objective of the previous studies was to screen potential *Ft* vaccine candidates. Using LVS as a benchmark, because it is the only U.S. candidate vaccine evaluated in people to date, as the benchmark, we compared vaccines derived from LVS, *F. novicida*, Type A *Ft* SchuS4 (23–26), and single antigens (R. De Pascalis and K.L. Elkins, unpublished data). Through these screenings using Fischer rats, we found that *Ftt-ΔclpB* (*ΔclpB*) and *Ftt-ΔclpB*/*ΔcapB* (*ΔclpB*/*ΔcapB*) elicited both immune responses and protection against challenge comparable to or better than LVS (24). These data supported their selection for further evaluation in NHPs. We then demonstrated that *ΔclpB* conferred protection against aerosol challenge in NHPs; although the sample size was limited, *ΔclpB* appeared to outperform LVS (27). Based on this body of evidence, the current study focused specifically on comprehensive and detailed comparisons of relevant immune responses of PBS-injected control rats with LVS- and *ΔclpB*-immunized rats at very early as well as later time points after vaccination, all without further *ex vivo* restimulation. This strategy was designed to capture informative changes in innate immune responses and the evolution of immune responses over time, with the ultimate goal of providing predictive biomarkers as quickly as possible after vaccination, as has been shown for BCG vaccination in rhesus macaques (32, 33) or humans (34).

Because both vaccines are live attenuated bacteria, we first analyzed their dissemination, replication, and clearance from animals (Fig. 1). We focused on spleens as a major target organ in the reticuloendothelial system. LVS and *ΔclpB* vaccination resulted in similar bacterial counts in spleens at day 3 post-vaccination, but bacterial levels and timing toward clearance differed thereafter. LVS was below detectable levels and presumably cleared by day 17, while *ΔclpB* persisted at low levels until day 24. The mechanism behind *ΔclpB*’s prolonged persistence is unclear; it may result from its faster replication rate and/or enhanced resistance to host immune responses compared to LVS, due to its derivation from Type A *Ftt* (10, 35).

In our previous studies evaluating anti-*Francisella* humoral immune responses, IgG assessments on days 14–39 after immunization showed no differences between LVS and *ΔclpB* vaccines (24, 28). Here, we extended this assessment to include IgM analyses at earlier time points when IgM typically peaks. Although animal numbers were limited, anti-LVS and anti-*ΔclpB* IgM and IgG titers were both comparable between vaccine groups (Fig. 2). Differences observed within each group on days 17 and 24 likely reflected animal-to-animal variability rather than true vaccine effects. These results are consistent with previous data indicating that humoral immune responses in rats or NHPs do not correlate with protection (24, 27, 28). Nonetheless, antibody evaluations remain useful for tracking vaccine “take” or potentially comparing optimal vs sub-optimal vaccines (19) or vaccination doses (27).

In typical innate immune responses, neutrophils and NK cells represent the first line of defense against bacteria, followed by dendritic cells, monocytes, and finally B and T cells. By selecting multiple time points, we could assess this profile through flow cytometry analyses. The results highlighted a few differences between vaccine groups in the relative distribution of dendritic cells, monocytes, and NK/NKT cells at early time points after vaccination (Fig. 3; Table S1). In addition, the relative percentage of T cells increased at late time points; of note, after *ΔclpB* vaccination, this increase was mainly due to CD8^+^ cells. Although these observations were limited by the small sample size, results in PBLs were supported by results analyzing splenocytes, where some differences between the two vaccines remained evident despite lower non-B-cell numbers compared to PBLs.

To evaluate gene expression in PBLs, initially we assessed selected genes by RT-PCR to confirm their differential regulation at chosen time points. Results confirmed that gene expression analyses are a valid tool for evaluating immune responses shortly after vaccination and detecting differences between vaccines (Fig. S2). Samples were then analyzed by RNA-seq. PCA analysis readily demonstrated differences between the two vaccines (Fig. 4A). After further analysis and filtering all data, the most striking difference was the higher number of genes and more pronounced upregulation and downregulation in the *ΔclpB* group (Fig. 4B and C; Table S2). In comparison, in LVS-vaccinated animals, relative gene expression values were lower and returned to baseline more rapidly than in *ΔclpB*-vaccinated rats. Notably, the biphasic expression of IFN-γ, particularly evident in PBLs from *ΔclpB*-vaccinated rats (Fig. S2), was confirmed by RNA-seq analysis (Table S2). This pattern may be associated with the role of IFN-γ as a mediator of both innate and adaptive immune responses, whereby distinct cell populations produce the cytokine at different time points during the response to *F. tularensis* infection (36).

Using these comprehensive gene expression data, IPA provided predictions of the most relevant signaling pathways after vaccination with LVS and *ΔclpB*. Not surprisingly, the IPA results reflected the genes’ upregulation and downregulation patterns, with *ΔclpB* appearing to induce responses involving more pathways and/or persisting longer than LVS (Fig. 5; Table S3). As might be expected, among the activated pathways and functions at days 3–7 are those involved in innate immune responses aimed at activating NK cells, myeloid cells, and leukocytes against microbial infection. In the early days after vaccination, the metabolic and cell cycle-related pathways appeared to be also highly activated following vaccination, while pathways such as the PPAR signaling appeared to be activated at later time points such as days 7–24 after either vaccination. Other pathways, such as the IL-12 signaling and the PTEN signaling pathways, were activated across all time points.

Overall, it appears that both LVS and *ΔclpB* induced strong immune responses immediately after vaccination, which may represent the foundation for generating protective immunity against virulent *Ft*. At present, it is not yet clear whether the higher number of upregulated or downregulated genes, and consequently the higher number of activated or inhibited pathways observed after *ΔclpB* vaccination, correlate directly with better protection. Some of these differences may be inherently related to the nature of the two vaccines. LVS is an attenuated strain derived from Type B *Ft* (*Fth*), whereas *ΔclpB* was derived from Type A *Ft* SchuS4 (*Ftt*). Moreover, *ΔclpB* replicates slightly faster than LVS and persists longer *in vivo* than LVS (Fig. 1). In addition, clpB is a chaperone important for protein quality control under stress. Its absence in *ΔclpB* may lead to the triggering of heightened danger signals through increased bacterial lysis, antigen release, and/or exposure of immunogenic intracellular components. These features could amplify innate sensing and antigen presentation, thereby activating a wider array of immune-related pathways.

Further studies are necessary to evaluate the impact of replication of these vaccines (Fig. 1), such as by comparing killed non-replicating vaccines or vaccines such as *ΔclpB-ΔfupA,* which replicates at a different rate than LVS or *ΔclpB*. Such studies would help determine whether differences in replication kinetics differentially influence the activation or inhibition of immune pathways (Fig. 5). Moreover, vaccines such as *ΔclpB-ΔcapB* and *ΔclpB-ΔwbtC*, which in rats provided protection comparable or inferior to *ΔclpB*, respectively, could also be useful to discriminate the impact of the common SchuS4 backbone. By evaluating the replication, gene expression profiles, and pathway activation patterns of *ΔclpB-ΔfupA*, *ΔclpB-ΔcapB*, and *ΔclpB-ΔwbtC* immediately following vaccination, using an approach similar to that described in this study, it may be possible to identify genes and pathways specifically associated with the most protective vaccine candidates, *ΔclpB and ΔclpB-ΔcapB,* by ruling out those associated with bacterial replication and the vaccine backbone.

Although both vaccines induced strong immune responses, our gene selection strategy, when considered collectively, detected potentially important differences in the magnitude and dynamics of host responses elicited by the two vaccines. Future studies will confirm individual gene-level signals and explore implicated mechanisms. Here, we demonstrated that the group as a whole discriminated the immune response against the two vaccines (19, 24, 37) and indicated pathway-level changes. By focusing on pathways known to mediate innate immunity (Fig. 5), we identified transcriptional programs that were differentially engaged following immunization. The substantially larger number of differentially expressed genes following *ΔclpB* vaccination suggested that this attenuated mutant drives broader activation of immune pathways than LVS. This, in turn, suggests more sustained intracellular signaling and eventually more cellular activation, more inflammasome activation, and more stress-related responses compared to LVS.

When examining specific genes linked to immune-related pathways (Fig. 6B), the differential patterns of regulation further underscored these differences. For the LVS-associated genes (Fig. 6B [B1 set]), divergence between the two vaccination groups became evident primarily at day 7, implying a more limited immunomodulatory effect of LVS. In contrast, most genes associated with *ΔclpB* vaccination or shared between both vaccines (Fig. 6B [B2 and B3 sets]) exhibited consistently greater degrees of expression changes in *ΔclpB*-vaccinated rats across multiple time points. These transcriptional responses may be indicative of stronger T-cell priming, improved dendritic cell maturation, and/or enhanced interferon-driven responses, all features that could contribute to more robust or durable protection. Notably, the RNA-seq findings were further validated by parallel microarray studies (De Pascalis et al., submitted for publication), which demonstrated an overall concordance between the results generated by the two platforms. Moreover, IPA, using either RNA-seq or microarray data, predicted similar signaling pathways following vaccination, further supporting the reproducibility and robustness of the observed transcriptional signatures.

Taken together, these findings suggest that *ΔclpB* vaccination engages a wider set of immune pathways and maintains transcriptional activation over a longer time period compared with LVS. Such differences may underlie the distinct immunogenic profiles of these two vaccines and could help explain subtle variations in their protective capacities (24, 27). Although multiple genes were differentially regulated after vaccination with LVS or *ΔclpB* at different time points, such as those involved in cell cycle and metabolism, these studies focused attention on a relevant set that includes those known to be involved in immune responses against microorganisms (Fig. 6B). Our approach to gene selection, while data-driven, is intentionally not limiting or prescriptive. Alternative selection criteria can be further evaluated and potentially integrated to complement or refine the gene panel we have identified. The specific genes highlighted in this analysis should be viewed as providing a focused, manageable panel that balances comprehensiveness with practical applicability. This curated set offers a strong foundation for initial implementation and for designing future mechanistic studies. Most immediately, these data support testing those that may serve as early biomarkers of vaccine-induced immunity and future efforts to define correlates of protection, while remaining open to expansion or modification as new evidence emerges.

## Data Availability

The raw sequencing reads generated in this study have been deposited in the NCBI Sequence Read Archive under BioProject accession no. PRJNA1478680.

## SUPPLEMENTAL MATERIAL

Fig. S1 iai.00208-26-s0001.pdf Gating strategy to identify cell types.

Fig. S2 iai.00208-26-s0002.pdf Gene expression by RT-PCR.

Table S1 iai.00208-26-s0003.pdf Leukocytes subpopulations.

Table S2 iai.00208-26-s0004.pdf Gene expression analysis by RNA-seq.

Table S3 iai.00208-26-s0005.pdf Canonical pathways analysis by IPA.

Table S4 iai.00208-26-s0006.pdf Selected genes.

ASM does not own the copyrights to Supplemental Material that may be linked to, or accessed through, an article. The authors have granted ASM a non-exclusive, world-wide license to publish the Supplemental Material files. Please contact the corresponding author directly for reuse.
