# Erratum for De Pascalis et al., “Transcriptional analyses of peripheral blood lymphocytes obtained shortly after primary immunization discriminate vaccines against *Francisella tularensis*”

**DOI:** 10.1128/iai.00516-26

**Published:** 2026-10-08

**Authors:** Roberto De Pascalis, Scott Espich, Hamda Khan, Mykia Toney, Chao-Kai Chou, Wells W. Wu, Karen L. Elkins

## ERRATUM

Volume 94, no. 9, e00208-26, 2026, https://doi.org/10.1128/iai.00208-26. Byline: Chao-Kai Chou’s and Wells W. Wu’s names should appear as shown in this Erratum.

