# Supplementary material for: Transcriptional analyses of peripheral blood lymphocytes obtained shortly after primary immunization discriminate vaccines against Francisella tularensis
Source: Infect Immun. 2026 Aug 14;94(9):e00208-26. doi: 10.1128/iai.00208-26 (PMC13552110; doi:10.1128/iai.00208-26)
Supplement: Supplementary file 1 [file iai.00208-26-s0001.pdf]

Fig. S1

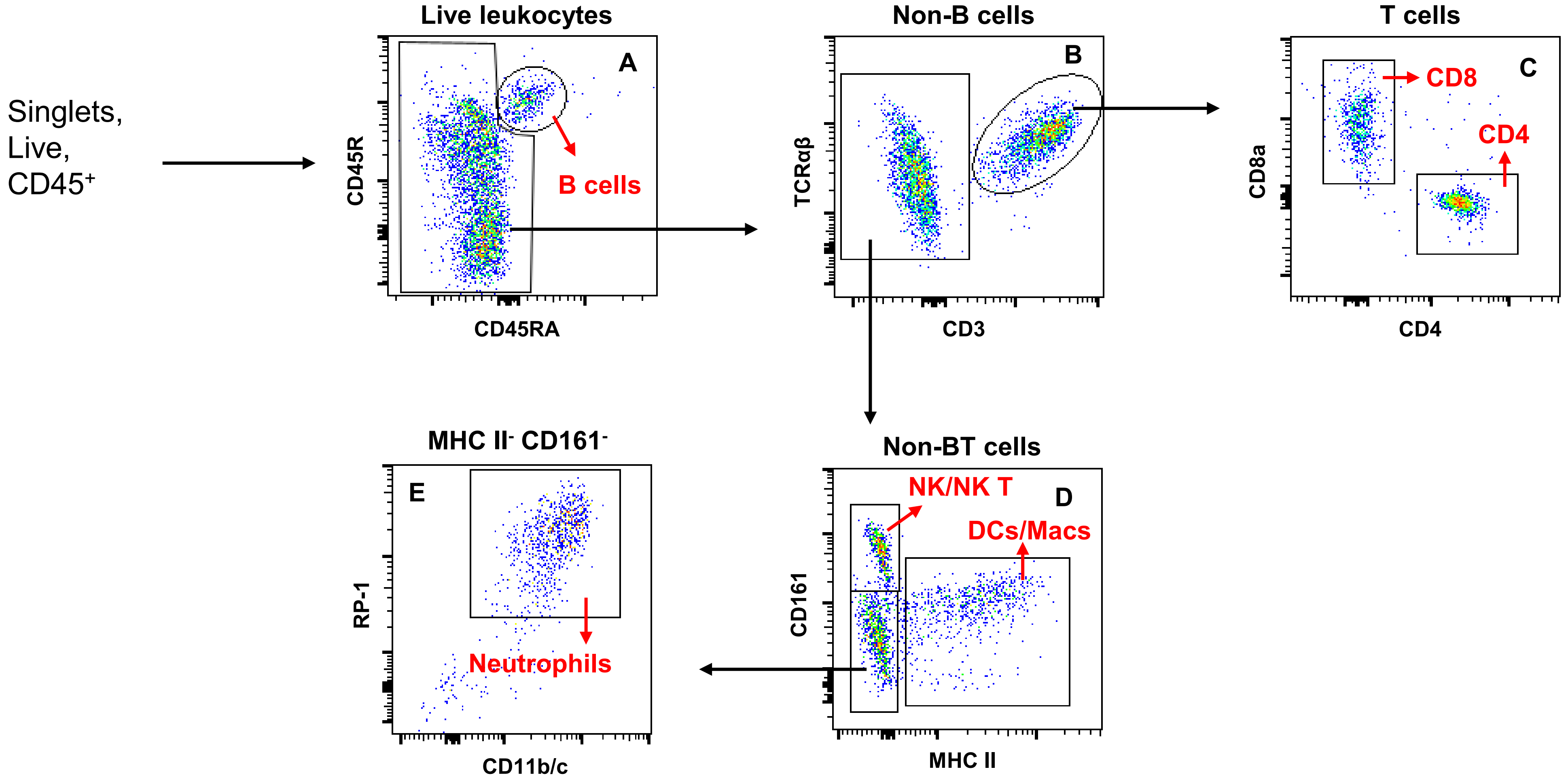

Figure S1. **Gating strategy to identify cell types.** After removing aggregates, fragments, and dead cells, $CD45^{+}$ live leukocytes were gated with CD45R and CD45RA to quantify B cells (A). To evaluate T cells, $CD45RA^{-}$ cells were first gated with TCRβ and CD3 (B), and then with CD8a and CD4 (C). Among non-B/T cells ($CD45RA^{-}$ $CD3^{-}$), MHC II and CD161 were used to identify dendritic cells, monocytes/macrophages (MHC II^{+}) and NK/NKT cells ($CD161^{+}$) (D). Finally, to identify neutrophils, MHC II^{-} $CD161^{-}$ cells were gated using RP-1 (E).
