# Supplementary material for: Transcriptional analyses of peripheral blood lymphocytes obtained shortly after primary immunization discriminate vaccines against Francisella tularensis
Source: Infect Immun. 2026 Aug 14;94(9):e00208-26. doi: 10.1128/iai.00208-26 (PMC13552110; doi:10.1128/iai.00208-26)
Supplement: Supplementary file 2 [file iai.00208-26-s0002.pdf]

Fig. S2

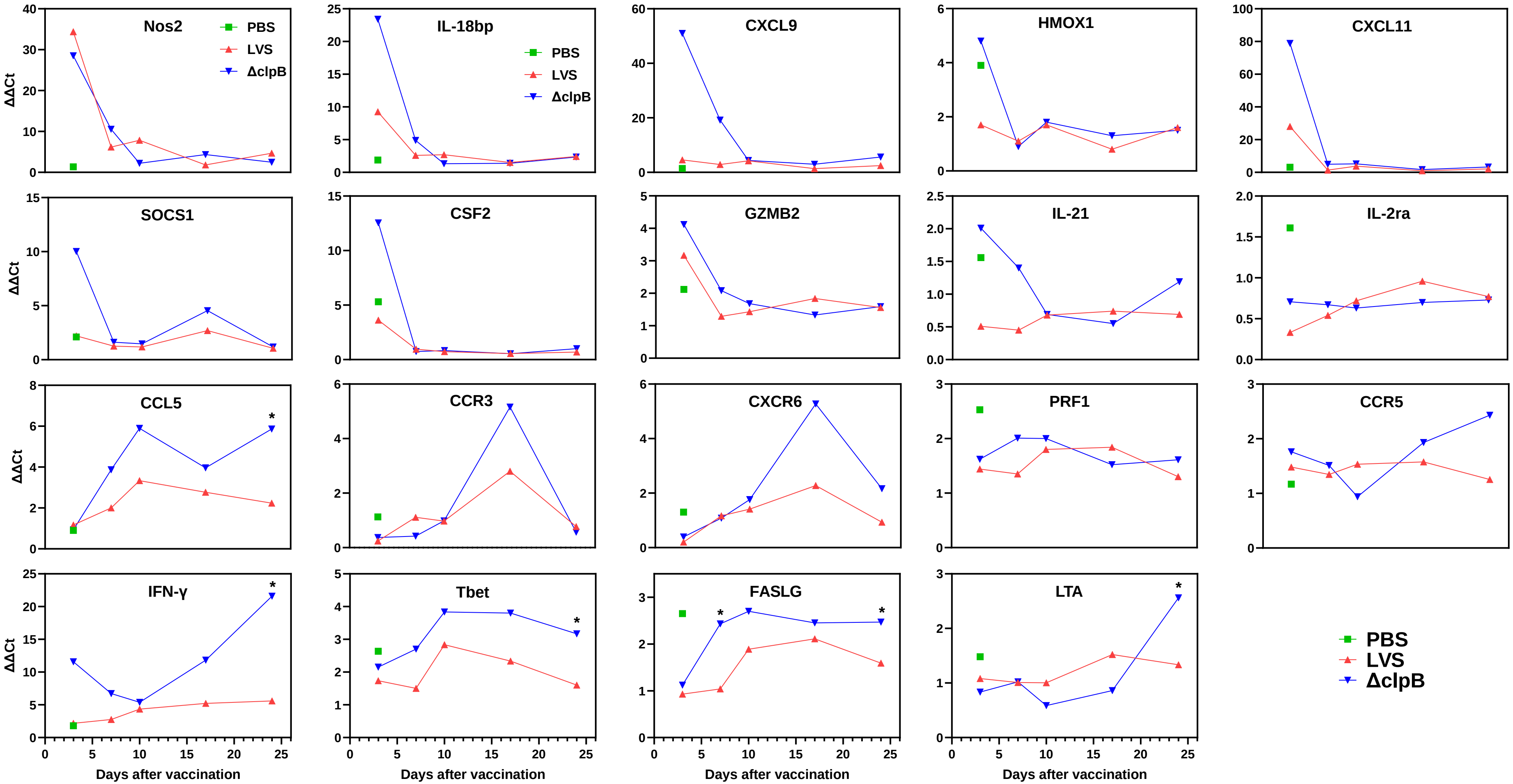

Figure S2. Relative gene expression of PBLs exhibits different patterns after vaccination by RT-PCR analysis. Fischer rats were vaccinated, and blood was collected as indicated. PBLs were prepared, and semi-quantitative analyses of gene expression were performed as described in Materials and Methods. Means of the ΔΔCt for each group were plotted. Within each time point, asterisks indicate significant differences (* = $P < 0.05$) between LVS and ΔclpB as calculated by t test with Welch's correction.
