# Supplementary material for: Transcriptional analyses of peripheral blood lymphocytes obtained shortly after primary immunization discriminate vaccines against Francisella tularensis
Source: Infect Immun. 2026 Aug 14;94(9):e00208-26. doi: 10.1128/iai.00208-26 (PMC13552110; doi:10.1128/iai.00208-26)
Supplement: Supplementary file 3 [file iai.00208-26-s0003.pdf]

Table S1. Leukocytes subpopulations in vaccinated Fischer rats

PBLs

	Naive	LVS					ΔclpB				
		Day 3	Day 7	Day 10	Day 17	Day 24	Day 3	Day 7	Day 10	Day 17	Day 24
**B Cells**	**18.9**±6.7	**11.0**±3.4	**13.5**±4.2	**17.7**±4.6	**13.5**±4.0	**10.4**±5.3	**12.3**±5.6	**8.4**±2.0	**12.8**±1.0	**7.1**±2.2	**10.7**±6.5
**T Cells**	**58.2**±1.4	**44.9**±5.7	**47.7**±6.0	**56.7**±7.3	**64.1**±3.9	**63**±5.3	**37**±7.4	**51.7**±3.0	**56.8**±4.9	**73.6**±1.8	**71.1**±2.6
CD4	58.2±3.1	61.8±3.8	53.7±3.8	51.2±1.9	48.3±4.4	50.3±8.3	58.3±7.9	53.7±4.2	41.6±12.5	35.2±3.0	34.1±9.0
CD8	37.5±0.7	35.7±3.9	43.7±4.0	45.8±1.5	45.4±5.0	40.9±7.8	39.4±7.9	43.7±3.7	53.7±12.9	57.3±2.7	49.3±2.2
**Non-BT**	**22.3**±7.0	**42.3**±6.0	**38.5**±1.8	**25.5**±6.6	**22.5**±1.8	**26.6**±9.0	**48.8**±11.0	**39.7**±2.7	**32.7**±7.9	**19.4**±4.0	**18.0**±2.6
DC/Mac	8.7±1.5	25.2±10.7	15.1±1.5	6.2±1.0	4.8±0.3	3.7±1.5	41.2±22.4	14.4±3.0	10.4±2.0	11.4±3.8	7.7±1.0
NK/NK T	55.5±6.3	28.3±3.4	73.8±5.0	77.9±10.8	67.8±1.4	60.3±17.4	12.2±6.9	64.6±11.8	70.4±16.7	65.1±6.7	47.1±8.0
Neutrophils	4.3±3.7	35.1±4.1	5.2±4.2	1.7±2.0	0.0±0	0.0±0	24.7±9.8	13.4±6.2	3.0±2.8	0.0±0	0.0±0

Splenocytes

	Naive	LVS					ΔclpB				
		Day 3	Day 7	Day 10	Day 17	Day 24	Day 3	Day 7	Day 10	Day 17	Day 24
**B Cells**	**61.4**±7.2	**55.0**±5.7	**47.1**±7.4	**49.7**±3.7	**52.5**±1.3	**60.6**±2.7	**50.9**±1.4	**45.4**±4.3	**44.6**±0.7	**49.5**±5.5	**51.1**±3.9
**T Cells**	**26.0**±7.1	**21.2**±0.7	**28.6**±1.1	**32.4**±0.3	**32.8**±1.9	**23.0**±5.5	**29.0**±2.2	**36.7**±3.6	**35.4**±2.7	**37.0**±5.8	**36.9**±4.2
CD4	49.9±2.6	46.6±5.4	46.1±1.1	49.4±3.8	45.8±0.6	48.6±5.4	46.3±5.3	43.9±1.7	40.9±1.4	36.4±1.6	42.0±5.5
CD8	42.9±1.7	46.6±4.4	44.1±0.7	44.7±4.9	45.6±1.5	44.8±3.9	48.8±7.0	46.5±1.5	53.4±1.8	55.0±0.6	52.0±5.0
**Non-BT**	**12.1**±0.5	**20.0**±5.2	**24.0**±6.5	**17.4**±3.5	**14.5**±1.7	**16.1**±3.7	**16.4**±3.0	**17.5**±1.8	**19.4**±2.1	**13.4**±0.3	**11.9**±0.9
DC/Mac	51.6±3.4	63.6±2.4	54.3±5.3	41.4±8.4	44.1±3.7	49.8±3.9	64.2±8.5	57.9±3.1	42.8±7.3	55.3±4.6	45.4±4.5
NK/NK T	37.8±9.9	24.0±5.3	41.5±5.2	52.6±10.8	46.1±4.8	41.3±3.7	16.3±7.1	38.5±4.0	53.5±8.2	35.7±3.5	43.0±5.2
Neutrophils	0.4±0.2	4.5±2.6	0.4±0.2	0.2±0.1	0.1±0.1	0.03±0.05	5.8±3.8	0.4±0.3	0.2±0.1	0.0±0	0.0±0

Table S1. Leukocytes populations in vaccinated Fisher rats. PBLs and splenocytes from vaccinated rats were prepared for analyses by multi-parameter flow cytometry, as described in Materials and Methods and Fig. 3. Leukocytes were gated as indicated in Fig. S1. After removal of fragments and aggregates, live $CD45^+$ leukocytes were gated to identify B, T, and non-B/T cells. Within T cells, CD4 and CD8 cells were quantified, and within non-B/T cells, dendritic cells, monocytes/macrophages, natural killer, NKT cells and neutrophils were identified. Data are represented by means and standard deviations. Data in bold indicate percent of total leukocytes; within T cells and non-BT cells, data indicate percent of subpopulations.
