# Supplementary material for: Transcriptional analyses of peripheral blood lymphocytes obtained shortly after primary immunization discriminate vaccines against Francisella tularensis
Source: Infect Immun. 2026 Aug 14;94(9):e00208-26. doi: 10.1128/iai.00208-26 (PMC13552110; doi:10.1128/iai.00208-26)
Supplement: Supplementary file 4 [file iai.00208-26-s0004.pdf]

Table S2. Genes differentially expressed in PBLs by RNA-seq. Total RNA from PBLs of vaccinated animals was used for gene expression analyses by RNA-seq, as described in Material and Methods and in Fig. 4. Section 1 lists alphabetically all the genes that were differentially regulated at all time points after LVS and *ΔclpB* vaccination. In sections 2 – 5, the list of genes was divided by time point and sorted by fold differences. Data values indicate fold differences, showing up- (in red) or down-regulated (in green) genes. Blanks indicate values that did not pass fold change and FDR p-value filters.

RNAseq gene expression: all time points
Fold change: < 1.5 or > 1.5
FDR p-value: < 0.05

	LVS / PBS				ΔclpB / PBS			
	Day 3	Day 7	Day 10	Day 24	Day 3	Day 7	Day 10	Day 24
1700066B19Rik								7.5
A2ml1								2.7
A3galt2					2.5		-2.2	
AA926063	7.5				9.3			
AABR07000382.1		2.5						
AABR07000398.1	2.2	5.0	1.7					
AABR07000658.1	2.7			-2.1		2.5		
AABR07001493.1					6.4			
AABR07001512.1	2.6					2.4		
AABR07001634.1					-1.9			
AABR07002001.1		-3.8	-2.7				-4.0	-1.8
AABR07002674.1	2.1				4.4			
AABR07002677.2								3.3
AABR07002898.1					4.3			3.9
AABR07002906.1		6.4						5.9
AABR07002969.1								-1.7
AABR07003304.2								-3.2
AABR07004993.1								3.5
AABR07006033.1					-6.1		-9.4	
AABR07006142.1		-2.2					-2.2	
AABR07006263.1	5.0				6.6			
AABR07006278.1	6.7	-9.8			10.4		-3.6	-3.6
AABR07006310.1	2.3	-6.3	-3.4	-2.3	2.5		-3.1	-2.1
AABR07006311.1		-2.8						
AABR07007121.1		1.6						
AABR07008568.1					-2.6			
AABR07008940.1		4.6		7.1		4.1		5.3
AABR07010563.1		-4.5						
AABR07011951.1	1.8	-1.6			1.7			
AABR07012475.2						-2.3		
AABR07012775.1		8.7	4.2		5.8			
AABR07013095.1	-9.1				-6.8			
AABR07013729.1						-4.6		
AABR07015055.1	1.6	3.4	1.7				2.0	1.8
AABR07015066.1	1.6	3.4	1.7				2.0	1.8
AABR07015078.1	1.6	3.5	1.7				2.1	1.8
AABR07015078.2	1.9	6.9		3.4		3.5	3.0	4.2

AABR07015080.2	1.6	3.3						2.0	1.8
AABR07016572.1	2.1					4.8	2.0		
AABR07016640.1									-2.7
AABR07016856.1	2.4					2.6			-3.6
AABR07017208.2		4.7						6.6	
AABR07017597.1				3.9					7.0
AABR07017604.1								4.9	
AABR07017626.1						-2.2			
AABR07017634.1						-2.1			
AABR07017635.2						-2.0			
AABR07017649.2						-2.2			
AABR07017669.1									4.5
AABR07017716.1						-2.5			
AABR07017737.1									2.5
AABR07017748.2									2.9
AABR07017818.1							3.1	4.6	4.3
AABR07017825.4									2.7
AABR07017825.5	-2.1								
AABR07017846.1	-1.7					-2.2			
AABR07017868.1						-2.4			
AABR07017868.3						-2.5			
AABR07017875.1						-2.5			
AABR07019083.1	2.1	-3.2							
AABR07019085.1									2.3
AABR07020815.1						-80.4		-8.3	-7.6
AABR07021022.1						-5.1		-4.8	
AABR07021221.1		-3.4	-2.3					-2.7	
AABR07021357.1								-6.1	
AABR07021717.1		-5.1					-4.1		
AABR07021946.1	-4.0					-3.5			
AABR07021996.1	-3.5								2.0
AABR07021998.1								-4.2	
AABR07022072.2	-3.0								
AABR07024972.1		-4.4	-5.8	-6.3				-6.3	-4.8
AABR07026311.1				-2.6		1.7			-2.4
AABR07027272.1			-3.4						
AABR07027447.1						-2.3			
AABR07027866.1		-5.2				-4.5			
AABR07027872.1						-1.7			
AABR07028113.1							5.0		
AABR07028523.1						-4.6			
AABR07028995.2				6.3					
AABR07029202.1			4.6	3.2				6.4	12.9
AABR07030086.2		-2.8	-2.5					-2.9	
AABR07030091.1		-13.8							
AABR07030544.1									7.3
AABR07030598.1						-9.6			
AABR07030773.1		-3.6						-3.1	
AABR07030793.1								-3.9	
AABR07030901.1		2.2					2.2	2.0	1.9
AABR07031489.1		-6.9						-5.8	
AABR07033324.1	1.7					1.8			

AABR07033887.1			-3.7						
AABR07034428.1	-3.4					-10.2			
AABR07034641.1		2.5		2.7			2.4		2.0
AABR07034669.1						-1.7			
AABR07034729.1		25.8	2.7				50.6		
AABR07034991.1						-10.2			
AABR07035273.1							-2.5		
AABR07035650.1	-3.2	-4.4					-3.9	-5.3	
AABR07035722.1		-2.4	-1.5			-1.6		-2.6	-1.6
AABR07035776.1								-3.1	
AABR07035796.1									1.8
AABR07035955.1							-4.0		-2.8
AABR07036301.1	-3.3	-2.5				-5.3	-7.1	-3.5	
AABR07037528.1									3.0
AABR07041096.1	-2.1							-2.4	
AABR07043075.1	3.7					8.1			
AABR07043421.1			-1.6						
AABR07044414.1		3.7	3.4			6.4		3.4	
AABR07044454.1						4.3		-2.9	
AABR07044820.1								-3.8	-4.1
AABR07049695.3						7.7			
AABR07049695.4	4.8								
AABR07049769.1								-6.3	-4.5
AABR07049960.1	4.7								
AABR07051326.1							-4.9		
AABR07051533.1						-2.4			
AABR07051533.2	-1.9		-2.0			-2.4			
AABR07051551.1	-1.8								
AABR07051565.1	-4.2								
AABR07051592.2						-2.0			
AABR07051639.1						-2.3			-1.6
AABR07051675.1							3.3		
AABR07051708.1						-1.9			
AABR07051733.1	-2.4								
AABR07052458.1						2.9			
AABR07052504.1								-4.5	-4.8
AABR07052529.1									-2.2
AABR07052608.1								6.1	7.1
AABR07053188.1							3.3		
AABR07053483.1		-4.2						-8.5	-2.7
AABR07053483.2								-4.0	-3.3
AABR07055805.1		64.3							
AABR07058124.4		-3.6						-2.6	
AABR07058464.1	4.4					9.1			
AABR07058479.1								1.6	1.9
AABR07058511.1			4.6				4.2	4.8	
AABR07058788.4									-4.5
AABR07058955.1	5.4								
AABR07058985.2							-1.9		1.6
AABR07059004.1									2.4
AABR07060872.1	-1.7	1.8							
AABR07060952.1									4.7

AABR07060953.1									3.5
AABR07060963.1						-3.0			
AABR07060979.1						-2.7			
AABR07060995.1	-7.5	-4.1				-3.5			
AABR07061001.1						-2.6			
AABR07061036.1						-2.3			
AABR07061052.1	-2.2								
AABR07061068.2									-2.9
AABR07062068.1							-5.8		
AABR07062157.1						-2.5			
AABR07062170.1						2.2			
AABR07062183.1		-3.8	-1.8	-1.8				-3.0	-2.3
AABR07062800.1						-2.5		-2.3	-2.3
AABR07063424.1	1.6	3.5	1.7					2.0	1.8
AABR07063740.2	-2.3					-2.9		-2.3	
AABR07064867.2						5.6			
AABR07064878.1									5.0
AABR07065448.1							-2.5		
AABR07065656.7							-3.8		
AABR07065656.8						21.2			
AABR07065676.1			-2.5			-2.3			
AABR07065680.1						-3.1			
AABR07065688.1						-3.2			
AABR07065693.1						-3.3			
AABR07065693.3						-1.8			
AABR07065699.1						-2.5			
AABR07065699.2						-1.9			
AABR07065699.3						-2.1			
AABR07065699.4						-2.8			
AABR07065714.1						-2.5			
AABR07065750.2						-1.9			
AABR07065750.3						-3.0			
AABR07065772.2		9.8							
AABR07065778.3						-3.9			
AABR07065815.1						-3.1			
AABR07065815.2						-2.8			
AABR07065821.1			-2.6			-3.0	6.2		
AABR07065823.1			-2.1			-2.8			
AABR07065827.1						-2.0			
AABR07065837.1						-2.5			
AABR07065889.2	-3.0					-2.5			
AABR07067267.1						-3.8			2.6
AABR07069282.1	4.3					2.8	3.6		
AABR07071392.1									-2.7
AABR07072400.1		12.9		13.8			10.0		8.5
AABR07072639.2							-4.4		
AABR07073039.1							3.2		
AABR07073391.1	-2.6								
Aars2								1.7	1.9
Aatk									-2.5
Abca17						-7.1		-3.4	-4.1
Abca4	3.1					6.7			

Abca5									6.6
Abcb1a									-1.7
Abcb6								-1.9	-1.7
Abcc3		-2.5		-1.7				-3.0	-1.8
Abcc5							-1.6		
Abcd2		-3.5						-4.5	-3.3
Abcg2		-2.5	-1.9			-2.1	-2.4	-2.9	-2.2
Abcg3l1								1.5	1.8
Abhd14b									-1.7
Abhd2			-1.8						
Abhd4							1.6		
Abi1		-1.6							
Ablim1						-1.5			
Abo									-4.0
AC109891.1						3.3			
AC111292.2								-5.2	
AC117058.1									-2.5
AC118414.1		15.4		15.6					9.6
AC120076.1						-2.6			
AC120310.1		3.4					2.5		
AC120712.2									2.1
AC127076.2							-2.5		
AC128901.1							3.3		
AC129753.1				4.3					
AC130391.3				3.9					
AC134759.1	-2.0								
AC135026.1							5.2		
AC141521.1		2.0	1.6						
AC141997.1	-2.2								
AC142181.1		1.6							
AC142360.1	5.1					5.5			
Acadsb							-3.1		
Acap2		-2.2							
Acer2							-2.7	-3.9	-2.4
Acer3							-1.6	-1.5	-1.6
Ache	-2.2					-2.8			
Ackr3								-3.4	
Acod1	47.9		2.7			118.5	9.1	6.9	2.2
Acot7							1.7		
Acp2	1.9					2.2			
Acp5	-1.6					-1.6			
Acpp	1.6					1.9			
Acpt	2.4					4.4			
Acsbg1	-5.1					-6.6		-3.5	
Acsl5						1.5			
Acss1	-1.5					-1.7			
Actb						1.5			
Actg1	1.5					1.6			
Actr2		-2.0							
Actr3b						2.5			
Acvr1		-2.5					-2.9	-3.1	
Acvr1b						1.8			

Acvr2b		-3.8					-3.9	
Acvrl1	2.0	-3.5	-1.8		2.0			-1.9
Acy3								-1.9
Ada	1.5	1.5			1.5			
Adam11								2.3
Adam15		-3.3	-2.1	-2.1		-2.1	-4.0	-2.8
Adam17					1.7			
Adam23					-18.0			-4.4
Adam8	-1.8						-1.5	
Adam9		-2.9	-2.0		2.0		-2.5	-2.1
Adamts10					-1.6			
Adamts12					-3.9	-4.4	-4.7	-3.3
Adamts14		1.7	1.5		-1.6			
Adamts2		-3.4					-3.4	-3.6
Adap1								-1.5
Adap2	2.0	-6.5	-2.7		2.2		-3.7	-2.5
Adarb1					-2.2			
Adcy10							-3.5	
Adcy3					-1.7			
Adcy4	2.0				3.8			-1.6
Adcy5								-2.5
Adcy6					-1.9	-1.8		
Add2		2.6		-2.7	3.0	4.1		
Add3					-1.9			
Adgb					9.7			
Adgra2		-4.5	-2.7		-2.1	-3.2	-4.3	-2.3
Adgra3				-2.8	-2.7	-3.3	-3.0	-4.1
Adgrb2						-4.7		
Adgre1		-2.8	-1.9	-2.0	-2.1	-2.6	-2.9	-2.1
Adgre4		-4.6	-2.6	-2.1			-2.8	
Adgrg1					-2.8			
Adgrg3					1.9	2.5		
Adgrg5					1.6			
Adgrg6		-2.6		-2.1		-3.0	-3.1	-2.7
Adgrl3	8.7				11.0			
Adgrv1	-2.7							
Adipor2		-2.2	-1.7					
Adora3								-2.5
Adra2a	4.6							
Adrb1					-2.9			
Adssl1		-2.1					-2.4	-2.2
Afdn		-4.4	-3.1		-3.3		-3.6	-3.5
Aff3					-2.1			
Agap1			-1.5				-1.8	-1.6
Agfg2							1.5	
Agl	-1.5	-1.9			-2.1	-2.3	-1.6	
Agmat					2.2			
Ago4					-2.4	-2.0		1.5
Agpat2							-2.1	-1.6
Agpat3								1.6
Agpat4		-1.7					-1.5	
Agrn					2.2			

Agrp									-3.4
Ahcy	1.8					1.8	1.5		
Ahnak									1.6
Ahr							-2.3		
Ahsp		2.9		-3.2		4.4	6.9		-2.1
Aida	1.7					1.8			
Aif1	2.1	-1.7				3.1			-1.8
Aim1						1.8			
Aim1l									1.9
Ak2	1.8					2.1	1.8		
Ak4	2.7	-4.5				2.5			
Ak7	2.4	-6.9				5.5		-4.7	-5.1
Ak9									-3.5
Akap1									1.9
Akap2									2.3
Akap9									1.5
Akr1b10	-2.2	-3.4				-4.1	-3.2	-3.3	-2.0
Akr1c14				19.0					
Akr7a3		3.0					4.9	2.2	
Alas1						2.7			
Alas2		3.1		-2.6		2.6	3.5	1.9	-2.1
Alb				11.8					
Alcam		-2.8						-1.9	
Aldh1b1									-11.0
Aldh2								-1.5	-1.6
Aldh3b1		-2.1				2.0		-2.4	-2.1
Aldh7a1	1.8					2.0			
Aldoa						1.9			
Aldoc						2.2			
Alox12		-2.6						-4.3	-3.4
Alox12e		-5.7							-2.9
Alox15				-3.0					-2.7
Alox5									-1.9
Alox5ap		-1.9						-2.5	-2.3
Alpk1						1.8			
Alpk2	-2.2	-4.7	-2.7			-6.1	-7.9	-4.1	
Alpl						7.7		-3.2	-3.0
Als2cr12						-2.6			
Amdhd2						1.8			
Amer1									2.3
Amfr		-1.6							
Amigo1	-1.6					-2.0			
Amigo2									-2.3
Amot	2.4								
Ampd3						1.9		-1.7	
Amt							2.0		2.2
Amz1	-1.7	-1.9				-2.5	-2.3	-2.0	
Anapc13							1.6		
Angpt1		-3.9	-2.4				-3.3	-2.6	
Angpt2									-2.9
Angptl2		-5.2	-9.4	-3.8				-11.2	
Angptl4	-2.1	-3.4	-2.3			-2.4		-3.8	-2.9

Angptl8						-14.8		-19.0	
Ank1		3.3				3.0	5.0		
Ankdd1a		-3.8							
Ankh						-1.8			
Ankle1	3.9					2.3			
Ankmy1								2.2	3.4
Ankrd10						-1.6			
Ankrd12									1.5
Ankrd22						8.7	4.7		
Ankrd26									1.6
Ankrd33b	-2.1					-2.6	-1.9	-2.5	
Ankrd44									1.6
Anln	3.5								
Anlnl1	3.0								
Ano8							-1.7		
Anos1						40.3			
Anpep	1.7	-2.4				2.3		-2.4	-1.9
Anxa1		-1.8						-2.8	-2.2
Anxa2						1.5			
Anxa3		-2.8		-3.1		2.4		-4.3	-4.1
Anxa4		-2.4	-1.6	-1.5		1.7		-2.1	-1.8
Anxa5		-2.2						-2.4	-2.0
Anxa6							1.7		
Aoah		-3.3	-2.0					-2.5	
Aox3		-3.9		-6.3		-14.0			-3.9
Ap1s1							1.5		
Ap1s2	1.7	-2.7		-1.6		1.9		-1.6	-1.9
Ap2a2		-1.5							
Ap2s1						1.9	1.6		
Ap3b2									-2.7
Ap3s1		-1.8							-1.6
Apaf1	1.6	-1.6							
Apba1		-7.5	-3.3	-2.7				-3.5	
Apbb1	-1.6					-1.9			
Apc									1.6
Apcdd1									-2.4
Apcdd1l						-3.0			
Aph1a						1.6			
Aplnr	-6.6					-7.7			
Aplp2		-1.7						-1.7	-1.6
Apobec1								-2.6	-2.0
Apobec2						-3.0			
Apoc3						-5.7		-6.7	-7.8
Apoe	-1.8	-1.6				-2.5		-2.0	-2.1
Apol11a	6.2					8.0	2.0		
Apol3	1.8					2.6	1.6		
Apol9a	9.8					20.1			
Apold1	2.2								
Apopt1_2		-3.7							
App	1.6					1.6			
Aqp1		2.9		-2.9		2.8	5.6		
Aqp12a						5.5			

Aqp9							-2.4	-1.9
Arap2							1.6	1.6
Arg2	3.3				17.8	3.5		
Arhgap11a	2.6				1.9			
Arhgap18		-1.8			-2.4		-2.6	-2.1
Arhgap19	3.3				2.0	2.4		
Arhgap22		-2.1					-2.5	-1.9
Arhgap24							-1.8	-1.6
Arhgap28		-4.9					-4.8	
Arhgap29								-3.7
Arhgap32					-8.8	-5.9		
Arhgap33	2.0							
Arhgap5			-2.9					
Arhgef10							-2.2	-2.2
Arhgef10l		-3.1	-1.6	-1.5		-2.1	-2.3	-1.8
Arhgef11							-2.2	
Arhgef18					-1.7			
Arhgef37				-3.5		3.8		
Arhgef39		2.4				2.4		
Arhgef5					-64.6	-6.9	-6.9	-5.9
Arid2								1.6
Arid3a		-1.7						
Arid3b		1.5	1.7				1.8	1.7
Arid4b								2.1
Arid5a					2.8			
Arl11		-2.2			-1.6		-2.7	-1.9
Arl14epl			3.5	4.1		5.6	7.5	9.6
Arl5b	1.7				1.8			
Arl6ip1						1.5		
Armc2					-2.8			
Armcx3								-1.6
Arntl					-1.6			
Arpc4					1.6			
Arpin		-2.4						-2.4
Arrb1		-2.4	-2.0					
Arrdc3					-1.7			
Arrdc4	4.7				4.6			
Arsa		-1.7				-2.0	-1.9	
Arsb	1.7					1.6		
Arsi								-2.6
Art3	6.1				8.3			
Art4	-1.5				-2.1			
As3mt	-1.6							
Asah1		-2.2					-1.9	-1.5
Asb13								1.9
Asb4					117.4			
Asb9	17.9				45.8			
Ascl2	-2.4	-3.4			-3.2	-2.8	-4.8	-2.5
Asf1b	4.4				2.9	4.5		
Asgr2					-15.4	-6.9	-8.0	-5.3
Ash1l						-1.6		
Asic1		-5.9			-8.2		-4.0	

Asic4								-8.4	
Asl	1.5					1.7			
Asmtl						1.5			
Asns						2.4	2.8		
Aspa						1.9			
Asph		-1.9				-1.8	-1.9	-1.6	
Aspm	3.2						2.4		
Asrgl1	1.7	-2.9		-2.0		2.9			-2.9
Ass1	8.8	-3.9		-2.2		14.9			-2.4
Asz1				-8.6		-79.1		-6.9	
Atad1						1.6			
Atad2	1.6								
Atad5	2.7								
Atcay			2.5	2.4				3.0	5.1
Atf3		-2.8	-2.6	-2.6				-3.6	-4.2
Atf5									-1.5
Atf6			-1.7						
Atg3						1.7			
Atn1									1.5
Atoh8									-6.9
Atox1						1.7			-1.6
Atp10a	3.1					4.4			
Atp10d	1.9					2.0			
Atp11a		-2.3	-1.6					-1.8	
Atp13a1						1.6			
Atp13a2		-1.7				2.5		-1.7	
Atp1a1		-1.7					-1.6	-1.6	
Atp1a3		-2.0				1.7		-2.4	-1.6
Atp1a4						4.2			
Atp1b1	1.7								
Atp1b2							3.8		
Atp2a2		-1.7							
Atp2a3								1.5	
Atp2b1		-1.9							
Atp2b4						-1.5			
Atp2c1		-2.2	-1.5						
Atp5i							1.7		
Atp5j2						1.5	1.7		
Atp5l							1.7		
Atp6ap1l						3.4	2.9		
Atp6ap2		-1.7				1.8			
Atp6v0a1						1.6			
Atp6v0c						2.1			
Atp6v1a		-1.7				1.7			
Atp6v1b2						1.7			
Atp6v1g1						2.2			
Atp6v1g2						3.5			-7.6
Atp8b4	1.9								1.6
Atp9a						-2.5			
Atpif1							1.6		
Atrnl1		-2.9	-2.4					-2.4	
Atxn1									4.3

Atxn7							-2.7		
Aunip	4.3					2.5	3.4		
Aurka	1.8					2.1	1.8		
Aurkb	3.4					2.4	3.0		
Axin2	-1.5					-2.1			
Axl				-6.5		-29.5	-7.0	-5.6	-6.1
B2m	1.5					1.8			
B3galnt1								-3.5	-3.5
B3galt5						-2.3			
B3gnt2	-1.5					-1.6			
B3gnt3	-4.3					-8.6	-3.3	-2.9	-1.8
B3gnt7			1.6				1.7	1.8	1.6
B3gnt8									-2.4
B3gnt9							3.1		
B4galnt1		1.6					1.8	1.6	
B4galt4						-2.9			
B4galt5	1.7	-1.8				2.1			
B4galt6		-2.1		-1.8			-2.7	-2.5	-2.0
Bace2							6.5	3.4	
Bach1			-2.1						
Bahcc1			-4.5					-4.7	
Baiap2						-1.7			
Baiap3	-1.8					-2.4			
Bak1	1.6					2.4	2.0		
Bambi								-4.2	-3.5
Bard1	3.2					2.1	2.5		
Basp1	2.2					1.6			
Batf						1.7	1.9		
Batf2	53.3					122.6	12.1	6.7	
Batf3						2.1			-2.6
Baz1a		-1.6							
Baz2a									1.7
Bbs10						-1.9			
Bcam								-7.4	-4.6
Bcar1				-4.4		-16.2		-4.1	-4.6
Bcar3	-1.9					-1.8			
Bcas2		1.5							
Bcat1	2.0	-1.7				3.3			
Bcl11b						-1.7			
Bcl2						-1.8			
Bcl2a1	1.5					1.8			
Bcl2l1						1.8			
Bcl2l1-ps1	6.5								
Bcl3	1.5					2.6			
Bcl6								-1.6	
Bcl7c		1.6							
Bcl9l						-1.8			1.5
Bco1								-3.5	-3.0
Bcr									1.6
Bdp1									1.8
Bean1		-6.8	-5.5					-5.1	-4.2
Begain		2.0	1.7			-1.9			-1.5

Bend5	-1.9								
Best1		3.6		5.6			3.9		2.6
Bfsp1									-2.4
Bglap								-3.7	-3.4
Bgn								-4.3	-2.6
Bhlhb9									1.7
Bhlhe22						-4.0	-3.7		
Bhlhe40		-1.9				-1.7	-1.8		
Bicdl1						-1.6			
Bid						1.9			
Bin1		1.6							
Birc3						2.1			
Birc5	3.7	2.1				2.8	3.6		
Birc6									2.4
Blk	-1.7								
Blnk	-1.6								
Blvra		-1.9						-1.8	-1.7
Bmi1						-1.8			
Bmp2				-4.6			-4.4	-3.2	-4.6
Bmp7						-2.1			
Bmx		-2.1						-3.3	-1.8
Bmyc				-5.5				-5.2	-6.4
Bnip2		-1.6							
Bnip3						2.3			
Boc						-2.4			
Bod1l1									1.6
Bola2		1.6					1.7		
Bora	1.8								
Bpgm		2.8		-2.0		2.2	3.3	1.7	-1.8
Bpi	-3.7								
Bpifa6							3.8		-5.6
Bptf									1.5
Brca1	2.1								
Brca2						1.7			
Brcc3		-1.6							
Bri3						1.6			
Brip1	2.7								
Brms1l_2			2.0						
Brs3						7.1			
Brsk1									2.2
Bsnd						150.4			
Bspry	1.9								
Bst1						1.9		-1.6	-1.8
Bst2	2.1	-1.8				2.9			-1.6
Btbd8_1						-1.9			
Btg2								-2.0	
Btla	-1.9					-1.8			
Btnl10							9.2		
Btnl9	-2.4					-3.1			
Bub1	5.2					3.0	3.2		
Bub1b	3.7						2.3		
C1galt1		-2.0							

C1qa						-12.4		-10.8	-7.5
C1qb						-17.4		-9.4	-7.1
C1qc						-11.8		-10.1	-7.5
C1ql3						-9.6		-5.5	
C1qtnf1	2.1	-2.3				3.2			1.9
C1qtnf6							1.9		-1.8
C1qtnf9							10.4		
C1r	7.4	-3.3				12.8			-2.1
C1rl						2.7		-2.6	-1.9
C1s	25.9					40.3			
C2	5.8			-2.0		7.8			-1.8
C2cd4b				-19.3		-68.8			-8.6
C3	2.5	-2.2				3.6			
C3ar1						-2.2		-2.1	
C4a_1	3.1		2.4			20.2	2.6	2.8	
C4a_2	4.7					7.8			
C4b	13.9					21.0	2.7		
C5ar1		-2.1				1.7		-2.3	-1.9
C5ar2								-2.7	
C6						-43.0			
Ca5b				-2.1		-2.2	-3.2	-3.3	-2.2
Cables1		-4.3						-4.6	
Cachd1								-4.9	
Cacna1a			2.0					2.1	1.8
Cacna1b	32.1					286.1	18.5		
Cacna1c						-14.0			-6.8
Cacna2d2									2.3
Cacnb4						-4.7			
Cacng4	2.1	1.7							
Cacng8						-6.4			
Cadm1		-3.8	-2.7			-3.5		-2.9	-2.1
Cadm3	-1.8	-3.4	-1.8			-2.9	-2.5	-3.5	-1.9
Cadps2	3.1					3.1			
Calcrl		-1.9		-1.5					
Cald1								-2.7	-3.0
Calhm2		-1.6						-1.7	
Calml4						-4.0			-3.3
Calr						2.1			
Camk1								-2.8	-3.2
Camk1g			78.9				206.3	215.7	154.6
Camk2a						-2.3			
Camk2d							-1.7		
Camk2n1						-2.8			
Camkk2		-2.0							
Camp						4.2	3.0		
Canx		-1.5							
Capg				-1.5		2.2		-1.8	-1.8
Capn12				-2.5				-2.8	-2.4
Capn2		-1.8							
Capn3	-5.6	-2.1				-10.2	-7.1	-3.1	
Capn5						-1.5			
Capn9		5.0					4.9		

Car1						2.4		-2.1
Car12	3.1					3.5		
Car13		-3.1						-3.3
Car2		1.8				2.3		
Car4	5.9				138.1	7.4		
Car8						3.4		-2.2
Card10	-2.5						-2.9	-2.1
Card14	10.9				32.1			
Card19							-1.6	-1.6
Card9		-2.5	-1.5	-1.5			-2.6	-2.1
Carmil1				-2.6	-4.1	-2.6	-3.8	-3.0
Carmil3					-2.8			
Carns1					-1.9			
Cars					1.5			
Casc4					-2.4			
Caskin1						-4.9		
Casp1					2.4			
Casp12	5.5				19.0			
Casp4	3.1				4.7			
Casp7	1.6				1.7			
Casq1		11.8						
Cass4							-2.1	
Cast		-2.0					-1.5	
Cat						1.8		
Catip		-2.6						
Cav1				-3.4	-8.6	-3.5	-3.8	
Cav2	-2.0				-2.9			
Cbfb		-1.6						
Cbln2							-4.7	-3.8
Cbr3								-3.0
Cbx7	-1.7				-2.0			
Ccdc102a	1.7				1.7			
Ccdc12		1.6				1.6		
Ccdc134					2.0			
Ccdc142					-1.7	-2.2		
Ccdc149								-4.2
Ccdc167						2.2		
Ccdc177								3.5
Ccdc34	1.9							
Ccdc80					-3.3		-3.5	-2.3
Ccdc88a		-4.2	-2.3				-2.3	
Ccdc92					2.0		-3.2	-2.3
Ccdc93					1.7			
Cck					4.0	3.3		
Ccl17								-3.4
Ccl2				-8.8	-12.3	-9.2	-127.9	-20.8
Ccl22		-5.7						-3.9
Ccl24					-54.7		-10.2	
Ccl4								3.5
Ccl5		2.0	2.3	1.8	-1.7	3.0	3.5	3.1
Ccl6		-1.7			-1.9		-2.5	-1.9
Ccl7				-9.0	-57.6	-14.8	-77.2	-16.1

Ccl9		-2.4	-1.8		-2.2	-2.6	-2.4	-1.9
Ccna2	5.5				3.3	4.2		
Ccnb1	4.0				2.6	4.7	1.7	
Ccnd1				-3.4	-2.4	-4.1	-4.4	-3.2
Ccnd2	1.8							1.6
Ccnd3					1.6	1.7		
Ccne1	4.3				3.7	5.1		
Ccne2	3.8				3.6			
Ccnf	3.8				2.6	4.0		
Ccpg1					-1.7			
Ccr1		-2.1					-3.2	-2.0
Ccr2	3.1	-4.1	-4.4		5.2			
Ccr4	-2.4				-5.0		-2.5	
Ccr5	3.1	-1.8			4.2			1.6
Ccr6	-1.9				-2.1			
Ccr7	-1.6				-2.3			
Ccr9	-1.6				-2.7	-1.7		
Ccrl2		-2.2	-1.7			-2.2	-2.6	-2.2
Ccser1								-2.8
Cd101					-2.0		-2.2	
Cd109					-2.6	-3.9		
Cd14	2.8	-4.6	-2.1	-2.4	2.7		-2.7	-2.7
Cd163					-63.0	-9.4	-11.3	-14.2
Cd163l1					-4.5	-2.6		
Cd177					17.6	7.7		
Cd180	1.7				1.9			
Cd19	-1.8	1.7						
Cd1d1		-1.8					-1.5	
Cd2		1.8	1.8	1.6		2.6	2.2	2.4
Cd200	-1.9				-1.8			
Cd200r1		-2.5		-2.1			-2.1	-2.4
Cd200r1l	1.9							
Cd226		-2.0						
Cd24	1.9			-1.9				-3.0
Cd244		-1.8			-1.6		-1.7	-1.6
Cd27		1.5						
Cd274	2.1				2.9			
Cd276					-3.3		-3.4	
Cd28					-2.1			1.6
Cd300a		-2.7	-1.7				-2.6	-1.9
Cd300e		-6.3	-2.1	-2.3			-3.6	-2.5
Cd300lb	-2.0	-2.2			-2.1		-2.8	-1.6
Cd300le		-2.8	-1.7	-1.5			-2.9	-2.1
Cd300lf	3.1				6.3			
Cd300lg				-13.0	-5.8	-11.3		-9.8
Cd302	1.9	-2.4			2.7		-1.9	-2.1
Cd33					4.1		-2.6	-2.1
Cd34		-3.8			-13.0			-3.7
Cd36	-3.2	-4.6	-2.2	-1.8	-7.1	-4.2	-5.0	-2.6
Cd3d	-1.5							
Cd3e					-1.6			
Cd3g	-1.5				-1.7			

Cd4	-1.7	-2.0	-1.8		-3.4	-1.9	-2.1	
Cd40					2.1			
Cd40lg					-1.8			
Cd44		-1.6					-1.6	
Cd52		3.7			2.7	5.4	2.3	
Cd55					-1.7			
Cd59		-1.8						
Cd5l					-4.9			
Cd6					-1.6			
Cd63							-2.0	-2.1
Cd68	1.8	-2.5		-1.5	2.1		-2.0	-1.9
Cd7					-1.5			-1.7
Cd72		1.7						
Cd74					1.9			
Cd79a	-2.1	1.8						
Cd79al	-2.1							
Cd79b	-2.0	1.6						
Cd80		-2.7						
Cd83	-1.6				-1.5			
Cd84		-1.7	-1.7				-1.7	
Cd86	-1.6	-1.9	-1.6		-2.0	-2.0	-2.3	-2.1
Cd8a						1.7	1.5	1.7
Cd8b	-1.6				-2.0			1.7
Cd9								-1.9
Cd93			-3.9				-2.8	
Cd99					1.6			-1.5
Cdc14b								1.9
Cdc20	4.1				2.5	3.8		
Cdc25a	1.6							
Cdc25b		1.8				2.4	1.5	
Cdc34						2.0		
Cdc42bpa		-3.5						
Cdc42bpb	1.8	-2.1			2.1		-2.1	-2.0
Cdc42bpg					-1.7			
Cdc42ep2	1.9				2.4			
Cdc42ep4		-2.6	-1.6				-2.4	-1.9
Cdc45	3.1				2.1	2.4		
Cdc6	4.6				3.3	4.9		
Cdc7	1.8							
Cdca2	4.2				2.4	3.6		
Cdca3	4.3				3.0	4.5		
Cdca4						1.6		
Cdca7	2.1	1.8				2.6		
Cdca8	3.5				2.5	3.5		
Cdh13	2.4							
Cdh23								1.7
Cdh5	3.4		-2.9		3.0			-3.2
Cdhr1	1.8	1.6	1.7			1.7	2.3	2.2
Cdk1	5.2				3.9	5.4		
Cdk17					-1.6			
Cdkn1a		-2.6	-2.3	-2.1	2.5		-4.6	-3.5
Cdkn1b					-1.6	-1.7		

Cdkn2a	3.9	3.1			3.3	6.7	3.2	3.1
Cdkn2aip					-1.5			
Cdkn2c	2.7					2.0		
Cdkn3	4.6	2.4			3.7	6.5		
Cdr2		2.1			1.9	2.3	1.8	
Cds1	1.9	-5.6			2.2			
Cdt1	2.2				1.7	2.2		
Ceacam1					-1.7	-1.8		
Ceacam18								1.9
Ceacam4							-3.6	-2.6
Ceacam6	-23.4				-47.2		-14.3	-12.9
Cebpa		-2.8	-1.6				-2.3	-2.2
Cebpb		-2.0			2.2	-2.0	-2.5	-2.2
Cebpd					1.8		-2.4	-2.0
Cebpe		-2.1				-2.0	-2.4	-1.9
Cecr6	-3.0	-3.8			-6.1	-5.2	-2.7	
Cela1								-2.7
Cela2a					11.0			
Cemip	2.3							
Cenpa	3.1				2.0	2.8		
Cenpe	4.9				2.8	3.0		
Cenpf	4.4				2.6	3.4		
Cenph	4.3				2.7	3.3		
Cenpi	4.0							
Cenpj					1.8			
Cenpk	3.5				2.5	3.4		
Cenpm	1.8	2.1			1.9	2.7		
Cenpn	2.0				1.9			
Cenpt	5.0				3.4	3.5		
Cenpu	3.7					2.6		
Cenpv					-2.2			
Cenpw	4.3				3.1	4.5		
Cep128	1.7							
Cep162								1.5
Cep170b					-1.9			
Cep295								1.5
Cep295nl							-6.5	
Cep350								2.0
Cep55	4.3				2.6	2.8		
Cep72	2.1							
Cep76								2.2
Cep97					-1.8			
Cercam		1.9	1.8			2.0	1.8	1.7
Cers1				105.7				
Cers4								2.2
Cers6		-3.4	-2.5		2.4			
Ces2c							-2.7	-2.4
Cetn3	-5.6							
Cfap126		2.6	1.8			2.2		
Cfap44	-2.1				-2.0			
Cfap57								-10.0
Cfb_1				-5.4	2.6	-3.0	-5.2	-6.2

Cfd				-2.7			-5.6	-4.2
Cfh		-3.9	-2.7	-3.4	-3.1	-2.9	-4.8	-4.7
Cfl1						1.6		
Cfp		-2.0		-1.9			-3.0	-2.5
Cgn								5.2
Cgnl1					-8.4			
Cgrrf1					1.6			
Chaf1a	1.8					1.6		
Chaf1b	2.2				1.8	3.0		
Chchd2		3.2		3.9		6.4		4.4
Chd3					-1.6			
Chd6					-1.5			
Chd7						-1.7		
Chi3l1		-1.7			1.8		-2.2	-1.7
Chid1					1.9			
Chmp2a					1.6			
Chmp2b					1.6			
Chn2	1.6					1.7	1.9	2.0
Chrdl1		-4.1					-6.6	
Chst10	-1.9							1.6
Chst12		-1.6					-1.7	-1.7
Chst13		-3.8					-2.4	-2.8
Chst3	-2.0				-1.7			
Chtf18	1.7							
Ciita	1.6				2.2			
Cirbp	-1.6							
Cisd3		1.8				1.8		
Cish				1.6			2.0	2.5
Cit	4.4							
Ckap2	3.0				2.1	2.4		
Ckap2l	4.6					2.9		
Ckap4					2.3			
Ckap5	1.6							
Ckb	1.6	-1.6			2.2		-1.7	-1.7
Cklf		-1.7			1.9		-1.7	-1.6
Cks1b	2.7				2.0	2.8		
Cks2	3.9				3.0	2.8		
Clca2		-2.9					-3.0	-4.0
Clcf1	-1.9				-1.9			
Clcn1								3.2
Clcn4	-1.8				-1.9			
Clcn7					1.8			
Cldn1						2.9		
Cldn10					-4.1			
Cldn3						11.3		
Cldn4								-2.2
Cldn7								-3.4
Clec10a		-3.8	-2.2	-2.8			-4.2	-3.9
Clec12a	1.7	-2.5			2.9		-2.0	-1.7
Clec12b						-2.9		
Clec1b							-4.2	-3.9
Clec2d2								1.5

Clec2l						2.1			-3.2
Clec4a		-7.5	-2.6					-4.4	-3.1
Clec4a1		-2.7	-1.5					-2.2	-1.9
Clec4a2	-1.5	-3.1				-1.6		-2.7	-2.1
Clec4a3		-3.6	-1.9	-1.5				-2.6	-1.9
Clec4b2		-2.7				2.9		-3.5	-2.9
Clec4d		-2.2						-2.7	-1.7
Clec4e								-2.6	
Clec4f								-3.3	-3.0
Clec5a						2.0		-2.5	-1.9
Clec7a		-2.5						-3.2	-1.9
Clec9a		-2.7	-2.2			-3.2			-3.4
Clic1						1.7			
Clic2						-2.4			-2.3
Clic4	1.8								
Clic5	2.3							1.8	2.5
Clip2	1.5	-1.7				1.8		-1.9	-1.6
Clip3									40.5
Clmp						3.6			
Cln6						1.5			
Cln8		-1.8						-1.7	-1.6
Clnk	2.2								
Clpb						1.6			
Clspn	4.2						2.7		
Clstn1									-1.5
Clu								-4.5	-3.2
Cma1						-4.4			
Cmah	-1.7					-2.2			
Cmklr1	2.5					2.7			
Cmpk1		-1.5							-1.5
Cmpk2	1.5					2.3			
Cmtm4							-2.8		
Cndp1						-23.8	-4.7	-4.8	-4.0
Cndp2	1.8	-1.6				2.4		-1.7	-1.7
Cnga1						-2.7			
Cnih2								-3.1	-3.2
Cnih3	-3.5								
Cnksr3		-1.9					-2.0	-1.8	-1.5
Cnnm4							-1.5		
Cnot6l		-1.6					-1.6		
Cntfr			8.5						5.9
Cntn1									-9.9
Cntnap2						2.4		-3.3	-3.9
Cobll1						-1.8			
Col15a1	2.4					5.4			
Col16a1						-4.4			-2.5
Col17a1							-2.0		
Col20a1	-1.7								
Col22a1	18.8					36.4	6.5		
Col26a1		2.3							
Col3a1								-14.2	
Col4a1								-5.2	-5.6

Col4a2						70.9			
Col4a4				-2.8		-3.8		-3.7	-2.7
Colec12	1.7	-4.5	-2.1	-1.9				-2.7	-1.9
Commd9						1.7			
Coro2a			-1.8					-2.0	
Coro6	-2.2						-3.0	-2.5	
Cox5a	1.5					2.1			
Cox5b						1.6	1.7		
Cox6a1							1.6		
Cox6b1_1						1.5	1.6		
Cox7a2							1.6		
Cox7b						2.0	2.0		
Cp	3.2					3.2			
Cpa2							3.2		
Cpa3						-11.0			
Cpe				-4.5					-4.8
Cpeb1						3.5			
Cpm						4.4			
Cpne2		-1.9						-2.0	
Cpne3		-2.7	-1.9						
Cpne4									-2.5
Cpne7	-2.7		1.8	1.7		-4.0		2.0	3.5
Cpne8						2.8		-3.0	-2.9
Cpped1	1.7	-2.4							-1.9
Cpq				-2.4				-2.5	-3.4
Cpt1c						4.0			
Cr2	-1.8					-1.5	-1.6		
Cracr2b									-69.0
Crat	1.6					1.7			-1.6
Crb3		4.1		4.3					3.3
Creb3l4		61.7		79.8					64.2
Crebzf						-1.6	-1.9		
Creg1				-1.6		2.2	2.3		-1.6
Creld2	1.6					2.6	1.8		
Crim1		-3.6					-2.9	-2.9	-2.3
Crip2	2.2								-2.0
Crispld2						5.0			
Crk		-1.9							
Crlf3						-1.6			
Crmp1						-2.1			1.7
Crtam						-2.8			
Cryba4	2.7					9.4	3.8		
Crybb3		4.6					4.2	3.6	
Crybg3		-3.3						-2.4	
Crygs		3.2							
Csdc2						-2.1			
Csf1	1.9		1.8	1.8		2.9	2.7	2.2	1.7
Csf1r		-2.4	-1.6					-2.2	-1.5
Csf2						5.7			
Csf2ra		-2.3		-1.5		2.1		-2.4	-2.3
Csf2rb	1.9	-2.7				3.1		-2.2	-2.0
Csf3r						2.8		-2.6	-1.8

Csgalnact2		-1.7				-1.6		
Csnk2b						1.5		
Cspg5					-3.1			
Csrp1								-1.6
Csrp2	4.9	4.0			4.5	4.9		
Cst3								-2.7
Cst7	2.2				2.1	1.9	1.6	
Cstb	1.5				2.1			-1.5
Ctbp2	1.7	-3.7			1.8		-2.6	-1.8
Ctbs		-1.9					-1.8	-1.6
Ctdspl		-2.6					-2.5	-2.2
Ctgf		-2.6						
Ctif	1.8				2.1			
Ctla4			-2.1				-2.8	-1.9
Ctnna1		-2.0	-1.6	-1.6		-1.7	-2.0	-1.7
Ctnnb1		-1.5						
Ctnnbip1	1.8				2.2			-2.0
Ctrc					2.3			
Ctsb							-1.6	-1.8
Ctsc					1.9			
Ctsd							-1.5	-1.5
Ctse	-1.5				-1.7			
Ctsf		-1.7			-1.9		-2.2	
Ctsh					1.9		-2.0	-2.0
Ctsl		-1.8	-1.9	-1.9	-1.7	-2.0	-2.8	-2.5
Ctss	1.5				1.8			
Ctsw			1.5			2.0	1.7	2.3
Ctsz	1.8	-1.7		-1.5	3.1		-1.8	-2.0
Cttn_1							-3.4	
Cttn_2							-2.5	-2.2
Cttnbp2nl		-3.2					-2.6	-2.7
Ctxn1			2.9					2.5
Cux2					-4.9		-3.8	
Cx3cl1	6.9				14.5			
Cx3cr1		-2.2			-2.7			2.2
Cxcl1					-18.8		-27.4	-12.5
Cxcl10	22.9	-3.5			48.2	3.0		
Cxcl11	22.7	-4.7			49.1	6.9		
Cxcl14					-37.3		-6.3	
Cxcl16	1.6	-1.6						
Cxcl2				-6.8			-46.2	-9.8
Cxcl3							-17.0	-7.3
Cxcl9	21.5	-2.6			58.6	8.2		
Cxcr2							-2.5	
Cxcr3				1.6		2.7		1.7
Cxcr4			-1.9				-2.4	-1.6
Cxcr5	-1.7							
Cxcr6	-1.9			1.9	-3.6	2.3	2.8	4.4
Cxxc4		-3.7					-3.8	-3.3
Cyb561d2						1.7		
Cyb5r1						-1.6	-1.6	
Cyba					2.3			

Cybb	1.6	-2.1		-1.7	1.7		-1.7	-1.8
Cybrd1		-1.9					-1.9	
Cycs				3.2				
Cyfip1	1.7	-3.6	-1.9	-1.8	1.8		-2.3	-1.8
Cyld		-1.7						
Cyp26a1				-8.2	-33.3	-6.8	-11.2	-14.6
Cyp27a1	5.0				10.7			
Cyp2d1		-2.9		-2.7			-3.2	-3.3
Cyp2d2		3.5						
Cyp2s1	-1.8	1.6			-1.8			
Cyp2u1					-1.9			
Cyp39a1	3.3				2.9			
Cyp4f17	-1.6							
Cyp4f18					2.3		-1.6	-1.7
Cyp4f5					-1.6			
Cyr61							-5.6	-3.9
Cysltr1				-4.1	3.0			
Cystm1					3.2			
Dab2		-3.3		-2.9			-4.9	-3.2
Dab2ip	1.7							-2.4
Dact1		-19.6						
Dact2							-9.6	
Dad1						1.6		
Dagla							-7.7	-6.1
Daglb							-1.6	
Dao	-3.0				-3.2			
Dap							-1.9	-1.8
Dapk1		-2.3		-1.6	-2.3	-2.6	-2.3	-1.6
Dapk2		1.5					1.7	1.6
Dapp1					2.0			
Daxx					1.8			
Dbf4	1.6							
Dbi	1.6				2.0	2.0		
Dbn1					-2.7		-2.5	-2.6
Dbnl					2.0			
Dchs1					-22.0		-4.7	-4.7
Dclk1	3.1	3.1	5.0	2.4	3.0	11.5	9.2	10.2
Dclre1c					1.6			
Dcst2				4.2				
Dctpp1	2.0				2.0	2.6		
Dcun1d2	-144.5		-135.8				-145.4	-141.3
Ddah1					2.5		-3.4	-2.2
Ddias	3.8					3.0		
Ddit3							-1.8	-1.6
Ddn							-5.0	
Ddr1							-3.9	-2.8
Ddt						1.5		
Ddx17						-1.7		
Ddx25	-2.1							
Ddx3x		-1.7						
Ddx58	1.8							
Deaf1		1.7						

Def8					1.6			
Defa5		16.7					13.2	
Defa7		19.5					12.0	
Defb14	-4.7				-4.4	-7.5	-3.1	-3.1
Defb24					6.0			
Degs2					2.4			-2.1
Dennd2c		-2.5				-2.4	-3.1	-2.5
Depdc1	5.3				3.0	4.1		
Depdc1b	2.5					3.5	2.1	
Depdc7				-2.8			-3.2	
Dfna5		-3.8	-2.4				-3.5	
Dgat1							-2.1	-1.6
Dgat2					3.0		-2.7	-2.0
Dgka	-1.5							
Dgkg			-1.9					
Dhcr24						1.8		1.7
Dhcr7						1.9		1.6
Dhdds					1.6		-1.5	
Dhfr	2.0					2.1		
Dhrs11		1.7			1.8	2.0		
Dhrs3	-1.7				-3.5		-1.7	-1.9
Dhx58	1.7	-1.8			2.6	-1.6		-1.5
Diaph3	3.6							
Dio3	-9.5						-14.4	
Dirc2					-1.7			
Dixdc1		-5.2			-3.4			-3.2
Dlat	1.5				1.7			
Dlg5			-4.0				-4.4	
Dlgap4					1.6			
Dlgap5	2.7				1.6	2.0		
Dmrtc2	-2.2				-2.7			
Dmtn						2.5		
Dmxl2		-3.6					-2.7	-1.9
Dnaaf1						2.9		
Dnah6	-2.7				-4.9			
Dnah8						2.8		-3.7
Dnajb11					1.6			
Dnajb4					-1.6			
Dnajb9						-1.8		
Dnajc1		1.5						
Dnajc16		-2.3	-1.8					
Dnajc17		1.7						
Dnajc22							-2.4	-2.0
Dnase1l3					-4.3			-3.4
Dnase2b						3.5		
Dner							-4.5	-4.2
Dnhd1								1.6
Dntt		3.4				-2.6		
Dock1		-2.8	-2.2	-2.4			-3.4	-2.5
Dock2			-1.9					
Dock3						-6.4		
Dock5								2.0

Dock7	1.7								
Dock9									1.8
Dok1								-1.5	-1.5
Dok2									1.5
Dok3						1.7			-1.6
Dok4		-2.7						-3.1	-2.2
Dopey1									1.6
Dpagt1	1.6					2.5			
Dpep2				-1.7				-2.0	-2.1
Dpm2							1.6		
Dpp4						-1.8			
Dpy19l1		-2.0					-1.6	-1.6	-1.7
Dpysl2	1.5	-2.2	-1.6						
Dpysl5						-2.5			
Dram1		-1.8					-1.8	-1.7	
Dsc1		-13.0	-4.5					-4.6	
Dsc2	2.3								
Dscc1	2.7						2.0		
Dse		-2.1					-2.1	-1.6	
Dsg1	5.1					6.0			
Dsg3	1.7	-3.8		-1.8		2.6		-2.6	-2.1
Dsn1	1.5								
Dsp						-4.4	-4.3		
Dst									1.7
Dstn						1.7			
Dstyk							-1.9		
Dtl	4.2						3.0		
Dtnb	2.2					3.0			
Dtnbp1						1.8			
Dtx3		1.7							
Dtx3l	2.4					3.2			
Dtx4								-2.0	
Duox1								-3.9	-4.2
Duoxa1						-12.5	-22.9	-11.1	-6.6
Duoxa2						8.3			
Dus2	-1.6								
Dusp1								-6.2	-2.5
Dusp11		2.0							
Dusp2		1.7					2.2	1.9	2.2
Dusp22								-1.8	-1.8
Dusp3		-1.9					-1.7	-2.0	-1.6
Dusp4	3.0								
Dusp5	-2.0	-1.7	-1.8			-2.5	-2.5	-3.0	-1.8
Dusp6		-2.8	-1.5	-1.5			-2.2	-2.1	-1.9
Dut	2.3					1.7	2.3		
Dvl3									1.6
Dydc1									-3.0
Dync1li2							-1.5		
Dynlrb1							1.5		
Dynlrb2									-3.4
Dynlt1	1.5					2.3	1.6		
Dzf17							-1.8		

Dzip1						-2.5			
E2f1	2.1								
E2f2						1.7			
E2f7	4.4								
E2f8	6.1					3.5	4.5		
Ebi3						2.0			
Ece1						1.7		-1.7	
Ecm1						-7.1	-2.9	-2.7	
Ect2	4.6					2.4	2.8		
Edar						-4.5			-4.3
Edaradd									3.0
Edil3		-5.5						-5.5	
Ednrb						-41.2			-7.6
Eef1akmt1							1.9		
Eepd1								-3.5	-2.7
Efcab11	3.1								
Efhd1						-3.2			-2.9
Efhd2	1.5								
Efna2		-3.5						-2.5	
Efnb2							-2.0		
Efs	-10.0					-12.7			-8.8
Egfl8						3.8			
Egfr		-9.5		-4.3				-4.3	
Egr1			-6.8	-4.8				-7.7	-5.1
Ehd3						-1.5			
Ehd4		-1.5						-1.7	-1.7
Ehf	23.5					19.5			
Eif2ak2		-1.6				1.8			
Eif2ak3						-1.5			
Eif4e2							1.6		
Eif4e3	1.8	-1.7				2.3			
Eif4ebp1						2.0			
Elane						5.5			-5.3
Elf4									1.7
Elk3						-1.5			
Ell3	-2.0								
Elob							1.7		
Elof1							1.7		
Elovl5						-1.7			
Emb			-1.7					-1.8	-2.0
Eme1	5.6					3.7	4.4		
Emid1		1.8							
Emilin2		-4.0	-2.4	-2.0				-3.4	-2.2
Eml1	5.4					7.1			
Eml2	1.6					1.7			
Emp1			1.8			-4.3	1.8	2.7	3.4
Emp3							1.6		
Emsy									1.6
En1									3.2
Eng				-3.4		-2.5	-2.7	-5.3	-4.2
Enho							-6.3	-5.2	
Enkur		-2.6						-3.5	-2.5

Eno1						1.7			
Eno3	-1.8					-2.4	-1.9	-1.7	
Enpep	-6.7							-11.2	
Enpp1						-2.1			
Enpp2						-2.6			
Enpp3				-3.5		-4.5			-4.0
Enpp4	2.4					2.9			
ENSRNOG00000000891									1.7
ENSRNOG00000014312		1.7					1.7		
ENSRNOG00000033109						-4.5			
ENSRNOG000000341	-1.6	1.7							
ENSRNOG00000043164						-1.8			1.7
ENSRNOG00000047185							1.6		
ENSRNOG00000047191						-5.1			
ENSRNOG00000047276						2.0		-1.5	-1.6
ENSRNOG000000480	-2.2					-2.3			
ENSRNOG000000480	-1.7								
ENSRNOG00000051592							-1.6		
ENSRNOG00000051719		-3.6	-2.3						
ENSRNOG00000051999						-1.9			2.0
ENSRNOG00000055238									2.2
ENSRNOG00000056059						-1.9			1.7
ENSRNOG000000562	3.7					2.5	4.3		
ENSRNOG000000576	2.8								
ENSRNOG00000058824		8.1					12.5		
ENSRNOG00000059750							1.8		
ENSRNOG000000598	-1.7					-2.2			
ENSRNOG00000060184						-3.0			
Entpd1	1.7					2.4			
Entpd2							1.9		
Entpd4						-1.5			
Eomes	1.8			1.5			1.9	1.7	1.8
Epb41l1						2.7		-3.8	
Epb41l5									2.1
Epb42				-3.8		2.6	3.7		-2.6
Epc2						-1.5			
Epha1								-3.3	
Epha2						-4.4	-2.4		-2.6
Ephb2			-3.4					-6.6	-3.4
Ephb4							-4.2	-4.1	-3.6
Ephb6						-1.9			
Ephx1	-1.7					-2.5		-1.6	-1.6
Epm2aip1							-1.9		
Eps8				-2.0					-2.2
Eps8l1								-2.4	
Eps8l2	1.9								
Epsti1	1.6					1.7			
Epx							3.4		
Erbb3	4.2					6.9	2.3	2.2	2.2
Ercc6l	1.8								
Erg						5.3			-6.2
Erlin1		-1.6							

Ermp1	1.5				1.7			
Ern1		-1.6						
Erp29					1.6			
Esam							-2.4	-2.6
Esco2	3.9				2.2	2.4		
Esd						1.6		
Espl1	4.4				2.8	3.4		
Espn	-2.1				-5.6			2.1
Espnl					6.6			
Esrp2				3.2				2.3
Etfb	1.6	1.8			1.5	2.1		
Ethe1					2.0		-1.9	-1.7
Etnk1						-1.5		
Ets1					-1.9			
Ets2		-2.0					-2.0	-1.6
Etv1				-4.1		-4.1	-3.9	-3.8
Etv5						-2.1	-2.0	
Ewsr1		3.0	2.2					
Exnef	1.9							
Exo1	4.7				3.5	4.2		
Exo5		-1.8					-1.6	
Exoc3l2	-2.0		2.5		-2.4		2.9	3.9
Exoc3l4					3.2			
Exoc4			-1.8					
Eya2					-2.0			
Ezh2	2.1				1.6			
Ezr					-1.6			
F10	4.2	-8.6			5.5		-3.4	-5.5
F11r						-2.2	-4.0	-3.5
F12					5.3			
F13a1		-3.0				-2.6	-4.7	-3.8
F2r					-1.7			
F2rl1					-3.5			
F2rl2							-3.7	-2.5
F2rl3							-2.2	
F3					4.5			
F5		-2.5	-2.1	-2.0			-3.2	-2.5
F8						-1.9		
Faah	8.6							
Fabp1				17.2				
Fabp3					3.1			
Fabp4		-5.0			-6.6	-6.1	-6.6	-4.7
Fabp5	2.6				3.8	3.1		-1.8
Fads1							-3.8	-2.9
Faim_1		-186.5		-216.2				
Fam102b	2.5		-2.6					
Fam111a	4.3				3.2	3.8		
Fam114a1					3.1			
Fam117b					-1.5			
Fam120a		-2.0						
Fam124a	2.0							1.9
Fam129b		-2.5	-1.9	-1.8			-2.5	-2.2

Fam129c				-2.1		1.9			-2.2
Fam131b								2.9	5.6
Fam131c									-2.7
Fam135a		-5.5						-3.7	-3.4
Fam13a						-14.8			-4.8
Fam149a									-2.3
Fam162a						1.6			
Fam168b		-1.5							
Fam184a		-3.2	-2.5				-2.5	-3.2	-2.6
Fam189b	-1.5					-1.7			
Fam198b		-3.4		-2.9		-2.6	-2.9	-3.7	-3.1
Fam199x			-3.5				-4.1		
Fam19a3				1.6		-2.8			1.8
Fam208a									1.6
Fam208b									1.5
Fam20a				-6.8		-14.2			-5.8
Fam20c		-3.3	-2.2	-2.2				-3.3	-2.9
Fam212a	-2.3								
Fam213a		2.0				1.9	2.1		
Fam214b						2.0			
Fam26f							1.6		
Fam3b						-8.7	-6.1	-5.3	-5.6
Fam43a							-1.6	-1.5	-1.7
Fam46a		-3.8		-1.8			-2.8		
Fam46b								-21.3	-19.6
Fam49a		-1.6							
Fam57a							-4.2		
Fam65b						-1.7			
Fam71a		-19.9		-26.1			-21.5	-71.5	-69.2
Fam72a	2.6					2.7	2.6		
Fam78a						-1.7			
Fam83d	3.5					2.6	2.7		
Fam83h	1.8								
Fam84b		-3.9							
Fam89b							1.8		
Fam8a1							-2.5		
Fancd2	3.5					2.3	2.5		
Fanci	2.2								
Far1		-1.6				1.6			
Far2									-4.6
Farp1									2.5
Fas						1.9			
Faslg	1.7		1.6	1.6			1.6	2.2	2.2
Fat1						-2.7	-3.6	-3.3	
Fat3	6.8								
Fat4									8.9
Faxdc2				-1.9					
Fblim1						-5.8		-4.4	
Fbll1						-3.0			
Fbln1		-3.8	-2.2	-2.8				-4.2	-4.9
Fbln5								7.1	16.4
Fbln7						-4.5			

Fbn1	3.7					4.7		2.5	
Fbxl15	1.7					2.3			
Fbxl2								-5.0	
Fbxl21						-4.0			
Fbxl4						-1.7			
Fbxl5						2.8			
Fbxo21						-1.9			
Fbxo32						-1.9			
Fbxo39						2.1			
Fbxo5	3.0					1.9	2.3		
Fbxo6						1.6			
Fbxw8	1.6								-1.8
Fcar						2.5			
Fcer1a							3.4		
Fcer1g						2.1			
Fcer2	-2.2	1.6				-2.3			
Fcgbp	-1.6					-2.1			
Fcgr1a	1.7	-1.8				3.2		-1.7	-1.7
Fcgr2b	-1.8	-1.6		-1.5		-2.5		-2.0	-2.0
Fcgrt									-1.8
Fcho1		1.5							
Fcho2		-2.0					-1.8	-1.6	
Fchsd2	-1.5					-1.6			
Fcmr	-2.0	1.5							
Fcna		-2.5		-3.1			-2.6	-4.4	-4.0
Fcnb	2.8	-7.6	-2.1	-2.0		4.1			-2.9
Fcrl1	-1.9	1.6							
Fcrl2						-4.7		-8.7	-8.0
Fcrl6	4.0					3.0			
Fcrla	-1.9	1.6							
Fech		2.6		-2.0		2.2	3.5	1.8	
Fen1	1.6						2.0		
Fer	1.9								
Fes		-1.8				2.2		-2.0	-1.7
Fetub						3.6		-2.3	
Fez2	1.8					2.9			
Ffar2						6.1			
Fgd4		-2.5	-1.8				-2.3	-2.5	
Fgf12						2.7		-2.5	
Fgf13							3.3		4.6
Fgf2									37.3
Fgfbp1		12.2							
Fgfr1						-9.5	-6.0	-5.0	-4.5
Fgl2		-1.6							
Fgr		-1.7				1.6		-1.7	
Fhl1		-2.6						-4.3	-3.7
Fhl3								-2.0	-1.8
Fhod3								-6.8	
Fignl1	4.0					2.6	2.6		
Filip1		-4.9	-3.3					-3.9	-3.3
Filip1l		-2.2							
Fis1		1.8					1.8		

Fkbp10	2.2							
Fkbp1b						5.2		
Fkbp2	1.5				2.2	1.8		
Flnb	-2.7	-1.9		-1.6	-6.9	-3.8	-3.4	-1.8
Flot2					1.8			
Flt3		-3.3	-2.4		-2.7		-3.4	-3.0
Flt3lg		1.7						
Flvcr2					6.7			
Fmn1			-5.0					
Fmnl2	2.4				2.4			
Fmnl3					-1.7			
Fmr1						-2.0		
Fn1		-2.7	-2.6	-3.5	-2.1		-5.0	-3.3
Fn3k		4.1				4.0		
Fndc3b		-3.7	-2.4				-3.1	
Fndc4							-12.5	
Fndc7		-9.7						
Fnip1		-1.6				-1.7		
Fnip2			-2.6					
Focad	2.2							
Folr2					-12.0	-4.7	-8.7	-5.0
Fos		-2.6	-3.8			-2.3	-13.1	-3.0
Fosb			-3.5			-3.3	-7.3	-2.2
Fosl2							-2.4	
Foxm1	3.3				2.1	2.3		
Foxo1					-1.8			
Foxp3	-1.6							
Fpr1							-3.2	
Fpr2	-2.9	-3.7	-2.0	-1.7	-3.4	-2.6	-2.6	
Fpr3		-2.5				-3.0		
Frat2	-1.7				-1.8	-1.9		
Frk						-6.1	-10.5	-7.5
Frmd4a			-2.8					
Frrs1		-2.6	-1.8	-1.7			-2.2	-1.7
Fry							-1.6	
Fryl								1.6
Fsbp	2.7							
Fscn1				-4.5	-10.0	-5.9	-5.5	-4.4
Fscn3	2.1				2.2			
Fst	-2.3				-3.0	-3.3	-3.6	
Fth1							-2.1	-2.0
Ftl1					2.0			-1.7
Fuca2	1.8				2.4			-1.8
Fxyd2		-2.4					-2.6	-2.3
Fxyd5						1.6		
Fxyd6					-40.8		-7.6	
Fyttd1						-2.0		
Fzr1						1.7		
G0s2	-2.7	-2.4	-2.3		-2.4	-2.8	-4.9	-2.4
G4					1.5			
G6b		-3.2					-4.1	-2.7
Gaa							-1.5	

Gab1	2.9	-4.3			3.9			-3.3
Gabarap					1.6			
Gabbr1			1.5					1.9
Gabrr2					1.9			
Gadd45a							-2.5	-2.0
Gadd45b					1.8			-1.8
Gadd45g	3.3				8.9			
Galns					1.7			
Galnt3					1.8			
Galntl5	2.3	-3.1						
Gamt							-1.6	-1.7
Gapdh	1.7				2.4	1.8		
Gapdh-ps2					3.2	1.8		
Gapt		-4.8	-2.4	-3.3		-3.1	-6.4	-4.1
Gas1					6.6			
Gas2l1	2.3	-2.7			1.9		-2.5	-2.1
Gas2l3	2.4							
Gas6					-13.3	-4.3	-6.6	-5.9
Gas7	1.8	-3.4	-2.3	-2.0	2.5		-2.2	
Gata2						2.2		
Gata3					-1.9			
Gata6					-42.1			
Gatm		-3.1		-1.9			-3.1	-2.7
Gba2					-1.5			
Gbp1	60.3				123.0			
Gbp2	6.8				17.5	3.3	2.2	
Gbp4	3.4				4.9		1.7	1.6
Gbp5	4.2				6.3	2.2	1.9	1.7
Gch1					2.7			-1.9
Gchfr		-1.8					-2.6	-2.1
Gclm					1.8	1.9		
Gcnt2	-1.6	-2.3			-1.7	-2.2	-2.1	
Gcsam			-2.3		-3.2			-2.1
Gcsh					1.8			
Gda	2.1	-2.8	-2.1		2.2			
Gdf1					-3.5			-3.0
Gdf10	3.4				2.5			-2.8
Gdf11								1.9
Gdf15							-7.9	-10.3
Gdf3	-2.3				-3.3	-3.6	-4.8	-2.8
Gdpd1					2.9			
Gdpd2							-4.1	-3.1
Gem		1.9	1.8				2.0	
Gen1	2.2							
Gfap					4.4	7.5		
Gfi1		1.6	1.7			1.8	2.0	1.9
Gfi1b							-2.5	-2.4
Gfra3					-1.9			
Ggct	1.6				2.4			
Ggh		-2.1		-1.6				-1.6
Ggn					2.1		-2.6	-2.2
Ggt1	2.2				7.8	3.7		

Ggt5				-1.8				-4.3	-2.8
Ggta1							-1.7		
Gigyf1						-1.7	-1.7		
Gimap6						-1.7			
Gimap8									1.5
Gins1	3.2					2.6	4.0		
Gins2	2.0						2.4		
Gins3	2.0						1.9		
Gipc2						3.1			
Gjb2		-2.8	-2.0	-1.9		-3.5	-3.7	-3.3	-2.0
Gjc1				-10.5		-10.2	-4.8	-7.0	-7.5
Gjc2	-3.0								
Gk						2.3		-2.0	
Glcci1								1.7	
Glipr1								-1.6	
Glipr2	1.5					2.7			
Glis2						-3.5	-2.6	-2.7	-2.6
Glrx	1.7	-1.6				2.6			-1.7
Glrx5		2.1				1.7	2.3		
Glt1d1						4.7			
Gm2a		-1.7						-1.9	-1.7
Gmnn	2.6					2.4	2.9		
Gmpr								-3.0	-2.8
Gna12									-1.6
Gna15						1.6			
Gnaz								-3.2	
Gnb4	1.9								
Gng10		-2.0				1.9		-1.9	-1.9
Gng11						2.1		-3.8	-3.8
Gng3	3.8	8.3	5.5			6.1	28.1	6.0	8.4
Gng8								-2.4	
Gngt2		-1.6							
Gnpda1						1.6	1.6		
Gnrhr								-7.0	
Golga7b						-3.5			-2.2
Golm1	-1.6					-2.6			
Gon4l									1.5
Gp1ba		-2.9						-4.1	-2.8
Gp1bb_1								-2.7	-2.4
Gp1bb_2						2.0		-4.5	-4.2
Gp6		-3.2					-2.5	-4.1	-3.8
Gp9								-4.2	-3.9
Gpat3									-1.9
Gpc1	2.8						6.5		
Gpm6b								-3.3	-3.2
Gpnmb	2.1	-2.4				3.3		-3.1	-1.9
Gpr137						2.0			
Gpr137b		-3.1	-2.0				-2.7	-2.4	-1.7
Gpr141	2.1	-4.7				2.4			-2.2
Gpr146						2.0			
Gpr149						-37.3			
Gpr150	-2.3					-2.5			

Gpr155						-1.8			
Gpr157	2.2	-2.9				2.5			-3.0
Gpr160	1.6								
Gpr171			1.7				1.7	2.0	1.8
Gpr174						-1.6			
Gpr176	-2.7					-2.7			
Gpr183	-2.0					-2.7			
Gpr25							2.2		
Gpr27		-3.6						-5.0	
Gpr31									-4.6
Gpr34		-5.1				-3.7		-3.1	-2.5
Gpr39						-56.9	-14.9	-8.4	-5.4
Gpr4	-2.5								-3.4
Gpr68	-1.6					-2.1			
Gpr84	5.7					15.2			
Gpr88	2.4					3.0			
Gprc5a				-5.8		-9.0	-4.6	-7.0	-5.2
Gprc5b									-5.6
Gprc5c		-7.8		-3.5				-3.2	-3.6
Gprc5d		-2.6		-2.7		-6.6	-2.4	-4.9	-4.0
Gpsm2	3.6					2.1	2.4		
Gpt2		-3.0				2.2		-2.3	
Gpx1				-1.5				-1.6	-2.1
Gpx3								-4.0	-2.9
Gpx7							7.6		
Gramd1c		-2.8							
Gramd3	1.7	-2.5				2.1			-1.9
Grap		1.5							
Grap2						-1.6			
Grasp									1.6
Grcc10		2208.0	169.0	341.4		305.9		194.4	59.6
Grem1									-4.5
Gria1						-3.7			
Grid2ip								3.7	
Grik3						-48.5			-9.6
Grik5	2.0					2.9			
Grina						2.7	1.8		
Gripap1						1.7			
Grk3		-3.9	-2.2					-2.3	
Grk5		-1.9						-2.0	
Grm4						-4.3			
Grn		-1.8				1.7		-2.1	-2.0
Gsap	1.6					1.9			
Gsdma								-2.7	-2.7
Gsdmc						2.3			
Gsg2						-1.6			
Gsr		-2.1						-1.8	-1.6
Gsta1								-3.0	-2.9
Gsta4		3.8				4.3	6.7		
Gstm1	-1.5								
Gstt2						-1.7			
Gtf2i						-1.8			

Gtf2ird1	-1.8				-2.2			
Gtse1	3.4				2.4	3.3		
Gtsf1	3.3				6.5			
Gucy1a2					-5.8			
Gucy1a3						-2.2		
Gucy1b2		28.4						
Gucy1b3		-2.8				-2.5	-3.1	-2.4
Gusb	1.6							
Gxylt1						-1.6		
Gyg1	2.8	-2.2	-1.8	-1.8	3.7		-1.9	-2.1
Gys1					1.9			-1.6
Gzma	3.1				2.7			
Gzmb	2.8				2.7			
Gzmbl1	3.4				8.3			
Gzmbl3	1.8	-2.5	-1.9			-2.7	-2.1	-2.3
Gzmc	45.6	175.4	118.7	292.7	326.8	250.9	31.7	122.7
Gzmk	1.7	1.9	1.9			3.1	2.4	1.7
Gzmm	1.5	2.1	2.2	1.7		3.7	2.9	2.9
H1fx		2.0				1.8		-1.6
H2afx	2.2				1.6	2.1		
H2afz						1.6		
H3f3b							-1.7	
H3f3c								-1.7
Haao		2.3					2.3	
Hacd2					1.6			
Hacd4							-2.1	-2.0
Hadha					1.5			
Hagh		2.0			1.8	2.5		-1.5
Hapln4								7.6
Haus4	1.5	1.7				2.0		
Havcr2		-2.4						
Hba-a2_1		3.5		-2.3	2.7	4.1	1.9	-2.1
Hba-a2_2		3.4		-2.3	2.5	3.6	1.8	-2.2
Hba-a3		4.0						
Hbb		3.4		-2.3	2.5	3.7	1.9	-2.1
Hbe1	6.2				10.7	3.0		-2.1
Hbegf					-3.6			
Hbq1b				-2.9		4.3		
Hcar2		-2.4					-3.4	-2.3
Hck		-2.2			1.6		-1.9	-1.7
Hcn2	2.3							
Hcst						1.6		
Hdac11					-2.1			
Hdac7					-1.5			
Hdc						3.6		
Hdgfrp3					-1.7			
Heatr9			3.4	2.4			4.3	8.7
Hebp1	2.1				3.6		-1.9	-1.9
Heca					-1.7	-1.7		
Hectd4								1.6
Heg1					-2.0			1.6
Hells	4.3							

Hemgn				-2.9	2.4	3.4		
Hepacam2	-2.4				-2.5			
Heph					-9.4	-4.3	-4.0	-2.7
Herc1								1.6
Herc2								1.5
Herc6		-1.9			2.6	-1.8		
Hes1	-3.6	-2.9	-2.6	-1.9	-6.2	-7.6	-6.7	-3.1
Hes5	-2.1							
Hexb								-1.5
Hfe					-2.7			
Hgd					-3.8			-4.8
Hgf		-2.8						-1.9
Hhex								-1.6
Hid1				-3.0				-2.6
Hif1a	1.5	-1.7			1.6			
Higd1a					1.7			
Hip1	1.5	-2.2			1.8		-1.7	
Hip1r	-1.5							
Hirip3	1.5							
Hist1h1c	-1.6		-1.7		-1.9		-2.2	
Hist1h2an			-4.0					
Hist1h2bcl1							-1.7	-1.7
Hist1h2bk	4.3				4.0	4.0		
Hist1h3a	4.7				4.7	5.0		
Hist1h3b_1	5.5							
Hist2h3c2_1	4.8				4.1			
Hist2h3c2_2	2.9							
Hist3h2ba		1.9				2.1		
Hjurp	4.1					2.8		
Hk2	2.5	-1.6			4.0			
Hk3	17.5				35.6	2.8		
Hlf					-5.4			
Hlx	1.7	-1.8			3.2		-2.1	-2.0
Hm13					1.9			
Hmbs					1.6	1.7		
Hmgb2	1.8	1.8			1.5	2.7		
Hmgb2l1	1.8					1.8		
Hmgb3	1.7							
Hmgn2					1.5			
Hmgn3	-1.7							
Hmmr	3.7					2.4		
Hmox1	1.7	-1.9			2.1			-1.5
Hmox2					1.5			
Hmx3					183.1			
Hn1l	1.5							
Hnf1a		2.7	2.0				2.5	2.1
Hnrnpc			1.6		1.7			
Hoga1					2.4			
Homer1								-5.0
Hook1	4.0				9.7			
Hopx	1.8				2.3	1.7		
Hormad2								2.1

Hp		-2.3		-1.8		2.4		-3.2	-3.1
Hpd			14.8	9.8					
Hpdl						-3.2			
Hpgd						-10.1			
Hprt1		61.9		47.9			36.3		58.6
Hps3		-1.6							
Hrh3		-5.5		-2.1					
Hs3st1	-1.9					-2.1			
Hs3st3a1		-11.6	-7.2					-4.3	-3.0
Hs3st3b1						-1.8	-1.9		
Hs6st1	3.0					4.6			
Hsd11b1	-1.9	-2.3				-5.0		-1.8	
Hsd17b1									-2.0
Hsd3b7	2.5					4.7			-1.8
Hsf4							-3.2	-2.4	
Hsp90aa1	276.0	1173.4							2451.6
Hsp90b1						1.7			
Hspa13		-1.6							
Hspa1a			-19.3					-9.4	
Hspa1b								-4.2	
Hspa2	2.0								
Hspa5						1.9			
Hspa8_2	1.9								
Hspb1				-5.5		-4.6		-14.6	-8.6
Hsph1									1.8
Htr2a	-2.7								
Htr7	4.1					6.1			
Htra1						11.5			
Htra3						-1.9			-3.0
Huwe1									2.0
Hvcn1	-1.8					-2.2			
Hyal2									-1.7
Hyou1						1.7			
Iah1						1.6			
Icam1	2.5	-1.7				2.7			
Icam5						-2.3			
Icos						-1.8			
Icoslg	-1.5								
Id1						3.2		-2.8	-2.2
Id2		-1.8							
Idh1		-1.9				1.6		-1.6	-1.6
Idh2	1.5					1.8			
Idi1	1.5								
Ids			-2.1						
Ier2								-2.0	
Ier3				-3.4			-3.3	-6.6	-5.9
Ier5								-1.7	
Ier5l			-2.0				-2.0	-3.2	-3.9
Ifi27l2b	-1.5								
Ifi30	1.8					2.8			-1.8
Ifi35						1.7			
Ifi44						2.2			

Ifi47	4.1				7.4	2.3	1.8	
Ifih1		-1.8						
Ifit1							-2.6	
Ifit1bl					3.3			
Ifit2		-4.1			2.4		-2.6	-2.1
Ifit3		-2.5			2.9		-2.7	-2.3
Ifitm1					2.7	2.3		
Ifitm10	2.5				3.6			
Ifitm2					1.6	1.8		
Ifitm3	2.5				19.8		-2.5	-3.1
Ifitm6	3.1				32.2	6.8		
Ifng	3.3			2.7	6.9	4.4	4.1	7.0
Ifngr2					1.8		-1.7	-1.6
Ift122					2.1			
Ift43					2.8			
Ift57					3.0			
Ift80	-1.5				-1.9			
Igdcc4					-6.6			
Igf1		-8.0	-5.7				-7.4	-4.9
Igf2bp2	4.8				9.3			
Igf2bp3					5.8			
Igfbp5								-4.9
Igfbp6					-26.5	-4.4	-6.9	-12.6
Igh-6		2.5				2.7		
Igll1					13.1			
Igsf6		-2.4			1.7		-2.1	-1.6
Igsf7							-2.4	
Igsf8					1.7			
Igtp	4.0				6.1	2.4	1.9	
Ikbke					1.5			
Ikzf3								2.5
Il10	2.7							
Il10ra		-1.9						
Il11ra1				-4.4			-3.3	-4.4
Il12b					-4.2			
Il12rb1	2.1		2.1	1.8		2.9	2.9	3.4
Il12rb2	4.6				3.0	2.7		2.9
Il13ra1		-2.8			1.9		-2.4	-2.0
Il15					1.9			
Il15ra	3.7				6.8			
Il17ra							-1.6	
Il17rc	-3.2						-4.4	
Il17re	-3.5				-6.2			
Il18				-2.4			-2.2	-3.0
Il18bp	6.5				16.8	2.5		
Il18r1			1.8	1.5	2.0	3.0	2.6	3.5
Il18rap	2.1		1.8	1.8	3.0	2.6	2.4	3.4
Il1a					6.1			
Il1b							-2.3	
Il1r1							-3.3	
Il1r2	-2.1				2.1		-3.9	-2.1
Il1rl1				-3.6	-3.7			-3.1

Il1rn		-1.9				2.4		-1.9	-1.6
Il22ra2	-2.5								
Il23r	-3.6					-7.8			
Il27	15.2					25.2			
Il27ra						-1.6			
Il2rb	1.7						1.8		
Il34		3.6	6.7	3.5			15.8	14.5	39.2
Il36b		-2.7						-2.3	-2.1
Il3ra						1.7			-2.1
Il4i1	-1.9					-1.8			
Il5ra							-3.8		
Il6st							-2.3		
Il9r	-2.1					-2.5			
Ildr1						-2.9			
Impa2	1.7					2.1			
Impad1_2			-587.1						
Incenp	2.6					1.8	2.3		
Inf2		-1.9				1.6		-1.8	
Inhbb						-40.6			
Ino80d									2.2
Inpp4a		-2.7	-1.8						
Inpp5f							-1.7		
Insig2	1.5								
Insl3		2.1							
Insrr		7.8							
Ints6l							-1.7		
Ipo7		-1.7							
Ipo9									1.7
Iqcf3	6.8					12.2			
Iqgap3	2.5					2.8			
Iqsec2						1.8			
Irf1	3.3					5.1	2.1	1.6	
Irf2bp2			-1.6					-1.8	
Irf5		-1.9	-1.5					-1.8	-1.7
Irf6	-1.6					-2.0	-1.6		
Irf7		-2.5				3.1		-1.9	-2.2
Irf8	1.8					1.9			
Irgm	4.6					7.4	2.5	2.0	
Irs1						-4.6	-3.6		
Irs3						-2.2			
Isca1		1.5					1.6		
Isg15	1.7	-2.1				5.8		-2.0	-2.2
Isg20						2.7	1.8		
Islr	2.2					2.4			
Isoc2b	1.7					2.3			-1.6
Ispd						3.0			
Itga2b								-4.6	-3.4
Itga4									1.8
Itga5		-2.0				1.8		-2.4	-1.6
Itga6		-1.8					-2.2	-2.0	-1.8
Itga9	3.5					2.7			
Itgad									-1.7

Itgae	-1.7				-1.9			
Itgb1		-1.6						
Itgb2					-1.7			
Itgb3		-3.8	-2.2				-2.7	
Itgb5						-2.0	-2.1	-2.0
Itgb7							-1.7	-1.7
Itk					-1.6			
Itpkb					-1.9			
Itpr3					-1.6			
Itpripl1	1.8							
Itsn1			-2.0	-2.2				-1.9
Jade2					-2.1			
Jag1		-2.9				-3.0		
Jak2					1.7			
Jakmip1					-1.6			
Jam2								-1.6
Jaml							-1.8	
Jarid2	1.6							
Jchain					-2.4	2.0		
Jdp2					1.7			
Jmy					-2.1			
Jsrp1		2.1						
Jun			-2.5				-4.7	-2.1
Junb			-2.1				-4.1	-1.9
Jup	-1.6				-2.0			
Kank1		-3.1	-2.2					-2.2
Kank2								-1.8
Kars					1.9			
Kat6a								1.6
Kat6b					-1.7			
Katnal2						9.7	5.6	6.3
Kazn	7.8				14.3			
Kbtbd11					-1.9	-2.2		
Kcna3		-3.2	-2.4					
Kcnab1		-3.4				-2.5	-4.2	-2.8
Kcnab3					-2.1			
Kcne3		-9.3	-2.7	-2.2			-3.5	-2.9
Kcne5	1.8							-2.2
Kcng2		-10.2			-3.0		-4.2	-2.7
Kcng3	4.8				7.9			
Kcnh2	-1.7				-2.1			
Kcnh3	-4.5				-3.3			
Kcnj1	-2.8				-2.5			
Kcnj12					-4.4			
Kcnj5	-5.5				-4.9			
Kcnk1								-1.8
Kcnk12					2.9			
Kcnk13		-3.1					-3.6	-3.7
Kcnk5	2.0		2.1			2.8	2.7	2.9
Kcnk7	1.9					3.0	2.3	2.3
Kcnn4	-1.5							
Kcnq5								2.3

Kcns1	-2.8				-5.5		-2.9	
Kcns3		-4.4	-2.3				-3.8	-3.6
Kctd17						5.8		
Kctd19	7.8				19.1			
Kctd4							5.2	5.2
Kctd9	1.6							
Kdelc2	1.5							-1.6
Kdelr3					2.6			
Kdm5b	-1.5				-1.7			
Kdr		-4.0	-3.1	-2.1		-3.0	-5.5	-3.3
Kel		2.7		-3.0		4.8		
Khdrbs3	-2.0							
Kif11	4.0				2.2	2.7		
Kif13a						-2.2		
Kif14	5.2							
Kif15	3.4				1.9	2.3		
Kif18a	2.6					2.2		
Kif18b	3.9				2.4	3.6		
Kif19	2.0							
Kif1b		-2.1	-1.6	-1.6	1.6		-2.0	-1.8
Kif20a	4.9				3.0	3.9		
Kif20b	4.7				2.5	2.8		
Kif21b								1.7
Kif22	3.9				2.8	3.7		
Kif23	4.1				2.5	2.8		
Kif24	3.0							
Kif2c	5.3				3.2	4.2		
Kif4a	4.5				3.0	3.8		
Kif7					-2.4			
Kifc1	3.0	1.8			2.1	2.8	1.5	
Kifc2	-1.7							
Kifc3				-2.7		-2.5	-3.2	-3.1
Kit					-2.5		-2.3	-2.4
Kiz					-1.7			
Klf1				-2.3	2.4	2.5		-2.4
Klf10		-2.1					-2.1	-1.6
Klf2					-1.6			
Klf4		-2.5	-1.9	-1.6	-1.8	-2.7	-2.7	-1.8
Klf6		-2.5	-2.3			-2.0	-2.2	
Klf8							-11.2	
Klf9					-2.4	-1.9		
Klhdc8a	-6.1							
Klhdc9					2.0			
Klhl11								1.9
Klhl13								-5.6
Klhl3								2.2
Klhl30							-5.3	
Klhl41	-2.8	-3.7						
Klhl42								1.7
Klhl9		-1.9						
Klkb1		1.7	1.8					
Klra1	2.0	2.6	3.2	3.3		3.4	3.0	2.9

Klra2		-1.9			1.8		-2.3	-1.8
Klra5_3			1.8	1.6		1.8	1.8	
Klra5_4		2.5	2.5	1.7		2.3	2.2	
Klrb1a	1.6				1.6	1.8		
Klrb1c						1.5		1.5
Klrc1				2.0		3.8	3.3	5.6
Klrc3			2.3			1.7	2.4	2.8
Klrd1			1.5		-1.5	1.7	1.7	
Klre1	1.7	1.7	1.8			2.6	2.1	1.9
Klrg1	1.8	2.5	2.3	1.6		3.6	3.1	2.7
Klri1	2.0							-1.7
Klri2	1.8					1.9	1.7	
Klrk1								1.8
Kmo							-2.0	-1.9
Kmt2a								3.1
Kmt2c								1.9
Kmt2d								1.8
Kmt2e								1.6
Kmt5c		1.6						
Knl1	4.3				2.4			
Knstrn	3.7	2.0			2.5	3.6		
Kntc1	3.4							
Kpna2	2.2				1.9	2.1		
Kpna4		-2.1						
Krt75		-2.0				-2.0	-1.9	
Kynu					1.9			
L1cam	-2.3				-2.8	-1.8		1.7
L3mbtl3						-1.6		
Lacc1	7.0	-2.5			7.5			
Lactb	1.7				2.1			
Lag3		2.1	1.8			2.5	2.0	1.5
Lair1							-9.6	
Lama2					-2.8			
Lama3					-3.2			
Lamb2							-3.1	-3.4
Lamb3	-3.3	-3.2	-2.4		-4.8	-6.7	-5.7	-1.8
Lamp1					1.7			
Lamp2		-2.3		-1.6	1.6		-1.7	-1.5
Lamtor3					1.7			
Lancl1					-1.5			
Lap3	3.0				3.9			
Laptm4b		-2.2		-2.3	-2.0	-2.9	-4.3	-3.9
Large1		-2.5					-2.7	-2.4
Larp1								1.5
Larp1b					-1.5			
Lax1	-1.6	1.7						
Layn	5.0				6.4			
Lbp	-1.7	-1.7					-2.5	-1.7
Lck						1.6		
Lcn2					8.9			
Lctl						6.6	10.1	11.3
Ldha					1.7			

Ldhc							-2.6	
Ldlr		-2.1					-2.1	
Ldlrad3	-1.7	-3.0			-2.9	-3.4	-2.2	-1.7
Ldlrap1					-1.9	-1.9		
Lef1					-1.7			
Lefty1	8.4				9.0			
Lefty2		-7.6					-5.1	
Lemd3						-1.5		
Lgals1						2.5		
Lgals2							-3.8	-4.6
Lgals3	1.5	-2.3			2.3		-2.6	-2.3
Lgals3bp	1.5				1.8			
Lgals5		2.3		-2.7	3.4	5.1		-2.4
Lgalsl							-2.3	-3.3
Lgmn		-1.9	-1.6		-1.6	-1.9	-2.4	-1.9
Lhb					-4.0			
Lhfp					-3.3	-5.8		-4.5
Lhfpl2		-6.3	-2.6				-7.1	-3.5
Lif			-10.2	-13.0	-5.0	-9.6	-27.5	-15.8
Lig1	1.9					1.8		
Lilra5		-3.1	-1.9				-2.3	
Lilrb3	10.6	-2.9			9.7			
Lilrb3a		-2.3			1.6		-2.7	-2.0
Lilrb3l		-4.4	-3.4				-4.3	-2.0
Lilrb4		-1.6			2.1	-1.9	-1.7	
Lilrc2		-2.8					-2.5	-1.7
Lim2	2.3				2.3	3.1	3.1	
Lima1		-2.1			-2.0		-2.2	-1.6
Lime1						1.8	1.6	1.7
Limk1					-1.5			
Limk2	2.2				3.3			
Lims1			-1.8					
Lingo3							-3.6	
Lipa		-2.0				-1.7	-2.0	-1.7
Lipg	1.9	-5.7			2.9			-1.9
Liph		-4.1	-3.0				-6.1	-3.2
Lipm	-7.4				-10.6	-21.6		
Litaf					2.4			
Lix1		7.1						
Lix1l	1.5							
Lmbr1l	-1.5							
Lmf2						1.5		
Lmna		-2.6	-2.0	-1.9			-3.5	-2.7
Lmnb1	2.7				2.1	1.7		
Lmnb2	1.8							
Lmo2		-1.7				-1.8	-1.7	-1.6
Lmo7	1.6						1.8	1.7
Lmtk2	1.6				1.8			
Lmtk3		3.4						
LOC100359539	2.2					2.9		
LOC100359600	2.4				1.8			
LOC100359687							-1.5	-1.5

LOC100359978						-2.9			
LOC100360087						1.8			-1.8
LOC100360169								-15.6	
LOC100360218	3.6					5.1			
LOC100360573	-3.8						-5.6	-6.0	
LOC100360575_1		-3.2				1.6		-2.7	-1.9
LOC100360575_2								-2.1	
LOC100360581						-2.0			
LOC100360891						-2.2			
LOC100360940		-4.1		-4.7					-4.9
LOC100361052						-3.5			
LOC100361087						3.2	2.7		
LOC100361105						-2.9			
LOC100361457			-335.0						
LOC100361706_1	-1.6	3.2					4.0		
LOC100361706_2	-1.8	1.8					1.6		
LOC100362149							1.7		
LOC100362298	53.6	76.6							76.4
LOC100362909	8.0								
LOC100363502						1.7			
LOC100364062	2.4					2.6			
LOC100364500		1.5				1.5			
LOC100909671						-2.0			
LOC100909700						7.1	6.1		
LOC100909856						2.1			
LOC100909857						47.1			
LOC100909879		-3.7	-2.1					-3.5	-1.9
LOC100910138		3.8	5.9	3.5			5.4	6.9	7.4
LOC100910163						-77.3	-72.0	-23.1	-9.8
LOC100910252	5.0					2.7	3.2		
LOC100910270	2.0					2.7			-1.6
LOC100910446		5.6							4.8
LOC100910554	3.2					3.3			
LOC100910636_2								-2.1	
LOC100910650								-4.5	-3.4
LOC100910669		-2.4	-1.6			-1.7		-2.7	-1.6
LOC100910792							6.8		
LOC100910801		-3.9				-2.5	-4.3	-4.4	-2.3
LOC100910838						-2.7		-2.9	
LOC100910882				1.6					1.6
LOC100910979_1	8.9								14.7
LOC100910979_2	2.3					3.2	1.7	1.9	
LOC100911101						6.0			
LOC100911104	3.0					4.9			-1.5
LOC100911109								6.2	
LOC100911166									51.4
LOC100911204	6.2								
LOC100911215						-1.8			
LOC100911238						1.7			
LOC100911271						-1.9			
LOC100911272						3.2			
LOC100911319							-2.3		

LOC100911403					-5.1		-3.7	
LOC100911453	1.7							
LOC100911483					2.0	1.5		
LOC100911516								-2.1
LOC100911519	2.0				2.2			
LOC100911548							-9.2	
LOC100911562	1.9				2.0			1.6
LOC100911575		1.9						
LOC100911581					-1.8			
LOC100911585	-1.6							
LOC100911615		5.6		6.4				9.4
LOC100911625		99.6	136.9	105.7				193.5
LOC100911660	1.8							
LOC100911730					2.9			
LOC100911825		-2.0			1.9		-2.6	-1.9
LOC100911830	1.9							
LOC100911840						1.8		
LOC100911851						-2.0	-2.1	-2.0
LOC100911881					3.7			
LOC100911917				2.2				2.2
LOC100911951						-55.9		
LOC100911956	29.9	72.5		95.6		55.0	30.1	95.6
LOC100911980					-2.3		2.2	2.3
LOC100912034				-3.6		-3.3		-6.6
LOC100912068							-2.9	
LOC100912163						-1.5		
LOC100912181	-1.7				-1.9			
LOC100912210						-5.1		-3.4
LOC100912218_2					1.9			
LOC100912228							-4.1	-3.3
LOC100912262					-2.0			
LOC100912485			14.6	14.0				
LOC100912498	-11.6	-17.6	-23.0	-15.0	-9.6	-24.6	-26.9	-27.8
LOC100912578		2.0						
LOC100912604						2.5		
LOC100912611	4.2				3.5	4.7		
LOC100912707					-2.0			
LOC100912917	1.8							
LOC102546864		-2.1						-1.6
LOC102547056	1.9							
LOC102547734							-4.8	
LOC102548472	-5.7							
LOC102549061	2.7				2.5			
LOC102549115		70.4			55.3			
LOC102549173	2.5				2.8			
LOC102549506			1.5		-1.8		1.7	1.8
LOC102549869					-1.7			
LOC102550441	-2.7							
LOC102550455						-2.3		
LOC102550456		2.2	1.7					
LOC102551095					-1.7			
LOC102551606			-19.4	2.3				2.6

LOC102552344	-4.0				-3.2			
LOC102553861	3.1							
LOC102553866					-2.0			
LOC102555453				-1.8				-2.1
LOC102555457	1.5				1.5			
LOC102555672	-1.5							
LOC102555924	2.0				2.7			
LOC102556085		-3.1	-3.1				-2.8	-2.2
LOC102556148	-2.2				-2.6			
LOC102556337			-7.2					
LOC103689927								-2.7
LOC103689942					3.0		-2.5	-2.2
LOC103689947		3.5		-3.0	3.4	6.4		-2.5
LOC103689968	-1.6				-1.6			
LOC103689996					2.0			
LOC103690020				-2.5			-2.5	-3.2
LOC103690118	2.5							
LOC103690131					-3.0			
LOC103690163	-1.8							
LOC103690166					-2.4			
LOC103690175								171.7
LOC103690190	3.0							
LOC103690326	-3.7						-5.0	
LOC103691013					-57.4			
LOC103691157					-2.9			
LOC103691261							-3.2	-2.5
LOC103691939				1.6				
LOC103692170		7.6						
LOC103692471		-2.9	-3.5			-5.8	-5.3	
LOC103692716	1.6				1.8			
LOC103692984						3.0		
LOC103693015	1.8							
LOC103694380					3.9	-73.7		
LOC103694853				-6.5	-3.6		-6.0	-3.6
LOC103694855		3.3		-2.5	2.6	3.9	1.9	-2.2
LOC103694857		3.5		-2.4	2.6	4.0	1.9	-2.1
LOC108348047		-2.0			2.3		-2.7	-1.9
LOC108348048	2.7				3.2			
LOC108348064					1.5		-1.5	-1.6
LOC108348072	1.7				2.2			-2.0
LOC108348078		-1.6						
LOC108348079				-5.4				
LOC108348091								-3.1
LOC108348096	364.2				335.4		218.7	161.5
LOC108348118			-2.3					2.2
LOC108348140	163.0	873.8						
LOC108348151								1.7
LOC108348155						-2.8		
LOC108348287		1.6						
LOC108350502		1.8				1.9		
LOC108351137	1.8				2.9	1.9		
LOC24906	2.1	-3.5		-1.8	3.4		-2.2	-2.5

LOC257642_1		10.7		6.7			5.6	3.6	7.2
LOC257642_3		27.1		29.1			19.0	12.1	31.1
LOC290595	2.0								
LOC303566	2.6					2.1	2.6		
LOC361016				-22.5		-49.1		-8.4	-13.4
LOC361346	1.6								
LOC497796	1.9					2.0			-1.8
LOC497963	3.0					9.9	3.3		
LOC498154						2.4			-1.6
LOC498265	1.6								
LOC498368								-4.1	-3.4
LOC499229									2.3
LOC499331						-1.7			
LOC500956						1.6			
LOC501110	-2.4	1.8				-1.9			
LOC502907						2.6			
LOC502908			5.7						4.8
LOC56764									-2.2
LOC679149		-4.9	-2.6			-2.8		-6.4	-2.5
LOC680121	1.7					1.9			
LOC680254		-3.3		-2.7				-3.4	-3.6
LOC680322	5.2								
LOC680910		-1.8						-2.6	-1.7
LOC681292			1.6				1.8	2.1	1.9
LOC681325		-2.2							-1.6
LOC681341	-1.8	-2.4	-1.6			-3.2	-3.6	-2.8	
LOC681355		-7.0	-3.8					-12.2	
LOC682206									2.1
LOC685048		-3.4				-2.6		-4.1	
LOC685067	4.0					4.4		1.8	2.0
LOC685157	-1.7	-2.2				-2.1		-2.6	
LOC685933		-3.0	-2.3						
LOC687679							1.8		
LOC688442							-6.1		
LOC688553	-2.0								
LOC688655	2.0					1.6			
LOC688684		7.1							
LOC689230	-3.4							-2.9	
LOC689574									-2.7
LOC690000						2.3			
LOC690020								2.4	2.7
LOC690288	-2.9					-3.5			2.5
LOC690483								-10.8	
LOC691141	2.1					3.1			-1.5
LOC691170						-2.2			
LOC691418		-4.2						-2.9	
LOC691695			-2.8				-3.3	-3.2	-2.9
LOC691716									-3.2
LOC691931						2.4			
LOC691995			2.3					2.3	
Lonrf3		-5.3				2.6		-5.5	-3.0
Loxl2									-2.4

Lpar1		-2.6				-2.1	-3.4	-2.3	-2.6
Lpar3									-2.6
Lpar4	-4.1							-7.1	-5.6
Lpar6						-1.7			
Lpcat2		-1.9						-2.1	-1.7
Lpcat3						1.6			
Lpgat1		-2.2							
Lpin1						-1.7			
Lpin2	2.2					3.5			
Lpl		-2.6		-2.5		-4.0	-4.4	-3.9	-2.3
Lpo						-27.9			
Lrch1						1.5			
Lrfn4						1.8			
Lrg1						2.1		-2.3	-1.8
Lrig1						-1.8	-1.6		
Lrig3									-4.0
Lrp1	1.9	-2.1				2.8		-1.9	
Lrp12									-1.8
Lrp4		-3.4	-2.3	-2.3				-4.4	-2.5
Lrp8	1.8					2.1			
Lrpap1		-1.8	-1.5					-1.6	
Lrr1	4.1						3.9		
Lrrc25		-2.2						-2.2	-1.7
Lrrc3							3.8		
Lrrc34						-2.2			
Lrrc39									12.9
Lrrc49		-2.3				1.9		-2.7	-2.4
Lrrc59	1.6					1.9			
Lrrc66	-3.5								
Lrrc72	3.5								
Lrrc75b						1.8	1.9		2.2
Lrrc8d		-1.6							
Lrrk2						1.6			
Lsm2	1.8					1.7	2.0		
Lsm4		1.7					1.7		
Lsr						-1.9			
Lst1						2.4			-1.6
Lta4h		-1.5				1.7			-1.6
Ltb4r						2.3		-2.9	-3.0
Ltbp1				-4.7		-7.8	-5.0	-5.7	-4.1
Ltbp3						-55.4	-7.8	-7.9	-7.4
Ltbp4			2.1					3.3	4.5
Ltbr		-2.3				2.3		-2.2	-2.0
Ltc4s						-13.3	-5.2	-7.6	-6.7
Luc7l3							-1.6		
Luzp1	1.6								
Lvrn						10.2			
Ly49i2	3.0					3.7	2.2	1.8	
Ly49i5		2.2	2.2				1.9	2.1	
Ly49s4	2.2	3.1	3.6	2.3			3.5	4.0	2.1
Ly49s6		2.4	3.8	2.3			2.9	4.3	4.5
Ly49si1		-1.7						-1.7	-1.8

Ly49si2	1.9	-2.8		-1.8	2.2			-2.0
Ly49si3	1.9	-3.0		-1.8	2.2			-1.9
Ly6al					3.3		-4.7	-2.8
Ly6e					2.1			
Ly6g6c							-4.7	-4.0
Ly6g6d	-2.4				-2.5		-2.3	
Ly6g6f					2.0		-3.7	-3.6
Ly6h					31.6			
Ly6l					2.7			
Ly75					2.0			
Ly86					2.0			-1.6
Ly9					-2.1			
Lyc2	2.3	-2.6			3.1		-2.5	
Lyl1							-1.7	-1.9
Lyn		-1.6			1.7		-1.6	-1.6
Lyplal1							-2.2	-2.1
Lyve1					-12.7	-6.2	-5.5	-5.5
Lyz2	1.9	-3.3	-1.8	-1.6	2.7		-2.8	-2.4
Lyzl1						3.6		
Macf1								1.7
Mad1l1					-1.8			
Mad2l1	2.5					2.4		
Maf		-2.5	-1.8		-2.2		-2.2	
Mafb		-3.1	-2.4	-2.6		-3.4	-4.3	-2.9
Mafg		-2.1					-1.9	
Mafk					1.7			
Maged1	-6.9				-5.4		-4.1	
Magi1		-2.9	-2.2		-2.6	-3.9	-3.1	
Magoh						1.6		
Magohb	2.2				1.7	1.8		
Majin	8.8				13.8			
Mal		-3.6	-1.8	-1.6			-2.6	-1.8
Malt1					-1.5			
Mamdc2				-6.0	-96.7	-11.0	-8.7	-8.7
Mamstr					-13.0			-8.8
Manf					2.1			
Maoa		-3.0	-2.2	-2.7			-3.9	-2.4
Maob	-2.9	-3.0	-1.8		-4.3	-4.3	-3.9	-1.9
Map1a	-3.9	-2.8			-90.1	-3.9	-8.8	
Map1lc3a		-1.6				-1.6	-2.2	-2.1
Map2k3				-4.5				
Map2k6					-1.7			
Map3k10					-1.7			
Map3k8						-1.6		
Map3k9		-1.9	-1.8				-2.1	-1.5
Map4k2					-1.6			
Map6				1.7		1.9	2.1	3.3
Mapk1		-1.6						
Mapk10	3.4				3.0			
Mapk13							-3.0	
Mapk15		2.3	1.8		-1.9			-2.1
Mapkapk2	1.6				1.9			

Mapkapk3		-1.7							
Marc1						-9.9		-4.9	-4.1
March2									-1.5
March3						-2.2			
March7		-1.9	-1.7			-1.6	-1.9		
March9									1.6
Marcks		-2.1				2.0		-2.0	-1.9
Marcksl1	2.9					3.2			
Mark1								1.7	
Marveld1	-1.8	-3.0	-2.1			-2.4		-3.4	-2.2
Mast4				-99.4				-8.5	
Mastl	3.9					2.5			
Matn2									-3.5
Max						1.6			
Mb21d1						2.1			
Mb21d2								-3.7	-4.2
Mbnl3	2.4						2.3		
Mboat1	1.6	-2.6				2.2		-2.0	-2.0
Mboat2	2.0								
Mboat7l1						1.7		-1.8	-1.6
Mbp			-1.5			-1.7			
Mbtps1						-1.5			
Mcemp1						3.2		-2.3	-1.8
Mcf2l						-1.8	-2.7		
Mcm10	3.1					3.2	2.4		
Mcm2	1.7						1.9		
Mcm4	2.0						1.5		
Mcm5	2.8					2.0	2.8		
Mcm6	1.8						1.7		
Mcm7							1.5		
Mcph1	1.7								
Mcpt1l1						-7.6			
Mcpt1l4									-2.0
Mcpt2								-3.4	
Mcpt4								-5.6	
Mcpt8						1.7	2.8		-1.9
Mcpt8l2									-2.0
Mcrip2	1.8					3.5			
Mctp1		-4.1	-2.2					-2.9	-2.1
Mctp2						1.6			
Mcub	1.7					2.0			-1.6
Mdfic		-1.9							
Mdm4									1.9
Mdn1									1.9
Me1		-2.7						-3.7	-2.9
Med12									1.6
Mef2a		-1.9							
Mef2b	-1.7					-2.2	-1.9		-1.7
Mef2c	-1.6					-1.6			
Mefv	1.8	-2.4				2.4		-1.8	-1.7
Megf8						2.1		-2.3	-2.0
Megf9		-3.2	-2.6					-2.3	

Meis1								-2.8	
Melk	4.7					2.7	3.3		
Mepce			-186.1						
Mest		-4.1						-4.6	
Metrn							3.3		
Metrnl		-1.9				-1.5	-1.7	-2.2	-2.0
Mettl7a						-1.6			
Mettl9						1.9			
Mex3b	-1.6					-2.3			
Mfap2						7.0			
Mfap3l								-3.4	
Mfge8						-1.9			
Mfsd1		-1.8							
Mfsd10		1.6							
Mfsd11						1.5			
Mfsd12		-2.2				1.9			
Mfsd13a								-1.6	
Mfsd14a									1.9
Mfsd14b		-1.8							
Mfsd2a						2.7	2.8		
Mfsd2b				-4.3					-4.2
Mfsd4						-2.7			
Mfsd6		-1.8					-1.9	-1.8	
Mfsd7	3.3					5.1			
Mga									1.8
Mgam						2.6			
Mgat2		-1.6							
Mgat4a	-1.6					-2.0	-2.0		
Mgat5b		14.6							
MGC105567	5.7					10.4	4.1	2.2	1.5
MGC105649	2.6					3.6	1.7		
MGC116202	2.5								
MGC95208						1.8			
Mgrn1		-1.6						-1.7	
Mgst1		-2.0				2.2		-2.1	-2.1
Mgst2								-1.8	-2.3
Micall2									-2.9
Micu1								-1.6	-1.5
Mid2	-2.1					-2.6			
Mier2		1.6							
Mif4gd	-1.5								
Miga1						-2.2			
Mip						2.9			
Mis18a	4.4					3.3	3.1		
Misp						3.2			
Mitd1						1.9			
Mitf	1.8	-4.9		-2.1		2.1		-2.3	-2.5
Mk1		8.9		15.1					9.1
Mki67	5.0					3.7	4.1		
Mkrn1							1.7		-1.6
Mlf1							4.5		
Mlkl	2.2					4.4			

Mllt10						-1.7		
Mllt3								1.6
Mllt6					-1.6	-1.8		
Mlxipl					-19.1	-6.4	-6.0	
Mmp14	18.5			-7.4	49.2	4.0		
Mmp19		-5.9	-2.8	-2.5			-3.7	-3.3
Mmp2					-287.5	-12.3		-57.0
Mmp8					3.8			
Mmp9					3.2		-1.9	-1.6
Mmrn1		-2.8					-4.2	-2.7
Mmrn2					-2.4			
Mms22l	2.6							
Mnda	2.4	-4.5		-2.0	3.5		-2.6	-2.5
Mob3c					1.7			
Mocos	1.8				2.9		-2.0	-1.7
Mocs1		-2.0					-1.7	
Morc1					-2.5			-2.0
Morc4		-3.1				-2.9	-4.6	-3.8
Mov10					1.7			
Mpa2l	2.6				2.7		1.8	2.3
Mpc1					1.5			
Mpl		-3.0					-4.6	-2.7
Mpo							-7.4	
Mpp3					-6.7			
Mpp5						-1.9		
Mpst					1.7	2.1		
Mpv17l2					1.8			
Mpzl2	-2.0				-2.7		-1.9	
Mras		-1.9			-1.8	-2.1	-2.1	-1.5
Mrc1		-4.6	-2.5		-2.4		-6.8	-3.3
Mrc2	-2.7	-2.6			-4.2	-4.8	-2.7	
Mrgprx3							-4.1	
Mrm1					-1.6			
Mroh2a	2.5				5.1			
Mroh2b					2.9			
Mrpl13						1.6		
Mrpl27					1.6			
Mrpl33								-1.5
Mrpl41						1.6		
Mrpl54					1.5			
Mrps11						1.8		
Mrps21						1.6		
Mrps6					2.3		-1.8	-1.7
Mrvi1							-3.6	-2.3
Ms4a2								-2.0
Ms4a4a					-6.2			-3.4
Ms4a4c					-2.1			
Ms4a6a	2.6	-3.0		-1.7	4.6		-2.3	-2.6
Ms4a6bl					1.7			
Ms4a6c					-1.8			
Ms4a7	5.6				7.6			
Ms4a8				-8.2	-36.3	-7.2	-7.6	-4.8

Msantd3								-3.8
Msc		6.1	7.8	5.4		13.0	10.7	13.6
Msh5								-2.9
Msl2		-1.8				-1.7		
Msln							-8.9	
Msr1				-4.5		-5.4	-5.4	-6.5
Msrb1					2.2		-1.8	-1.8
Msrb3	1.8							
Mss51		2.1	2.0					
Mt1	2.5	2.4			3.3	3.6		
Mt2A	4.1	4.0			7.7	12.4		
Mt-atp6		1.8	1.6			2.5		
Mt-atp8						3.5		1.5
Mtbp	1.7							
Mt-co1		1.6			1.8	2.8		
Mt-co2		1.6				2.1		
Mt-cox3		1.7	1.6			2.6		
Mt-cyb		2.0	1.7			3.0		1.5
Mterf2	-1.8							
Mtg2		1.5						
Mt-nd1		1.9	1.6			2.6		
Mt-nd2		1.8				2.9		
Mt-nd3						1.7		
Mt-nd4		1.8	1.7			2.5		1.5
Mt-nd4l						3.3		1.7
Mt-nd5		2.2	1.8		1.6	4.5	1.8	2.3
Mt-nd6				1.9		2.1		2.3
Mtss1					-2.1	-1.9		
Mturn					-2.0			
Mtus1		-2.2			1.9		-2.7	-2.0
Muc13						3.0		3.5
Muc20					2.4		-4.6	-3.2
Mvb12a					1.7			
Mvb12b					-1.7			
Mvd	1.6				2.1			
Mvp					1.7			
Mx2		-2.1				-2.6	-1.5	-1.9
Mxd1	1.5				2.2			
Myb						-2.0		
Mybl1	2.4							
Mybl2	4.9				3.4	5.6		
Mybpc3					2.9			
Mycl					-1.7			
Myct1		-3.2					-4.2	-3.1
Myd88					1.6			
Myh14	-2.4				-7.6			
Myh2	1.9							-3.0
Myh9l1			-2.4					
Myl9							-4.4	-3.2
Mylk		-3.0				-2.7	-3.8	-2.8
Mylk2				-3.7	4.1	3.7		-2.5
Myo16						4.2		

Myo19								1.8
Myo1b						-3.3		
Myo1f		-1.5						
Myo5a	1.9				2.0			
Myo5b						-8.6	-4.5	-4.3
Myo5c						3.0		
Myo7a		-6.5	-3.9	-2.8			-6.2	-3.0
Myof	2.2	-2.2			2.4			
Mypn					-5.1		-6.1	
Mypop						2.0		
Myrf							-4.9	
Mzb1		1.8				2.1		
N4bp1	1.6				2.0			
N4bp2l1	1.6				1.8			
Naa20					1.6			
Naa38						1.7		
Naa40	1.8							
Naaa	-1.7	-1.5			-2.0	-1.9	-2.1	-1.9
Nacc2								-2.4
Nadk		-1.7					-1.6	
Naga		-1.6					-1.6	-1.5
Naip6	1.9	-1.9			1.9			
Nampt	5.3	-1.6			10.4			
Nap1l1					-1.6			
Napa						1.6		
Naprt					2.6		-2.1	-2.0
Napsa		-1.6			1.5		-1.7	-1.7
Narf					1.9			
Nat9		1.5						
Natd1						1.6		
Nbl1					-4.6		-3.2	-3.0
Nbn	1.6	-1.8						
Ncald			1.5		-2.4		1.8	1.8
Ncapd2	2.5				1.7	2.4		
Ncapd3	1.9							
Ncapg	6.0				3.0	3.1		
Ncapg2	1.8							
Ncaph	4.8				2.9	3.8		
Ncbp2_2			-130.4					
Nceh1		-1.7						
Ncf1					1.8			-1.7
Ncf2		-1.9			1.8		-2.0	-1.7
Ncf4					2.0		-2.1	-1.9
Nckipsd					1.6			
Ncr1	1.6	1.5	1.7				1.8	
Ncs1	3.9							
Ndc80	3.3				2.2	2.8		
Ndrg1		-2.1	-1.6				-2.1	-1.7
Ndrg2	-2.2				-2.9			
Ndst1	2.0	-4.2	-3.4	-2.3			-3.1	
Ndufa12						1.8		
Ndufa3					1.5			

Ndufa5						1.6			
Ndufaf7	1.5	-1.7				1.5			
Ndufb10							1.5		
Ndufb7						1.8	1.6		
Nebl									1.6
Nectin2									-6.2
Nectin4								-4.7	
Nedd4						-1.7	-1.8		
Nefh									1.6
Neil1	-1.6	1.7							
Neil3	4.2					4.0			
Nek10						3.1			
Nek2	5.3								
Nek9			-1.6						
Net1		-1.6	-1.5					-1.6	-1.6
Neu3				1.8				2.3	2.4
Neurl1								-5.4	-3.4
NEWGENE_1304700		2.2							
NEWGENE_1306714	2.9					2.5	2.2		
NEWGENE_1310139			-3.0						
NEWGENE_1559832		-3.9							
NEWGENE_2319083		-4.0				-3.2	-3.0		
NEWGENE_2724		-2.5						-4.6	-3.4
NEWGENE_621351_1								-11.0	
NEWGENE_621438						68.8			
NEWGENE_68440		1.6							
Nf1x		-2.8						-2.1	
Nfam1	2.2	-2.1				3.7		-1.7	-1.6
Nfatc2									2.6
Nfatc3						-1.6			
Nfatc4		2.0	1.9			2.3			
Nfe2		-1.9				1.8		-2.4	-2.2
Nfe2l1		-1.8							
Nfe2l2	1.5	-1.8				1.6			
Nfia						-2.8			
Nfil3		-1.9						-2.5	-2.0
Nfkbia						1.5			
Nfkbiz		-2.0	-2.1	-2.1			-3.3	-2.9	-1.9
Nfs1_2	-65.8					-53.2		-65.5	-63.8
Nfu1									-1.5
Ngef	4.2					4.5			
Ngfr	-1.9					-3.5	-2.9	-2.1	
Ngp						4.8	4.2		
Nhp2						2.2			
Nid2						-8.4			
Nilr1		-2.3				1.6		-2.3	-1.5
Ninj1		-2.2		-1.7				-2.6	-2.4
Ninj2							3.2		
Nipa1		-5.1							
Nipa2		-1.7				1.6			
Nipal1						-1.6			
Nipsnap3b	1.6					2.0			-1.6

Nkg7	2.0	2.2	2.2	1.7	1.6	3.4	2.7	2.3
Nkpd1			3.7				4.2	3.6
Nktr								1.6
Nlrc3								1.6
Nlrc4	1.9	-2.6			2.5		-1.8	
Nlrc5	2.0				1.9		1.7	2.4
Nlrp12							-2.9	
Nlrp3		-1.9					-2.3	-1.6
Nlrx1		-2.1					-2.2	-1.8
Nme1					1.5			
Nme6					1.8			
Nmi					1.7			
Nmnat3							-2.3	-2.5
Nnmt						3.0		
Noct					3.5			
Nod1	2.8				4.1			
Nod2	1.8	-2.0			2.1			
NOS2	3.0				9.9	3.3		
Nostrin	3.1	-2.6			2.9			
Notch3			-4.0					
Nov					-6.0	-4.9		
Nova2		3.1	3.2			3.6	4.8	6.0
Npc2							-1.8	-1.7
Npff	-2.3							
Npl		-3.1		-1.7		-2.7	-2.7	-2.1
Npnt							-4.0	-5.5
Npr1	1.9	-2.8		-1.9	3.3		-2.6	-2.4
Nptn		-1.5						
Nptx1					-10.0			
Nptxr							-3.6	
Npy		-3.8		-4.5		-4.6	-3.7	-19.9
Npy1r	5.4				4.5			
Nqo1		-2.0			2.0		-2.0	-1.8
Nqo2							-2.0	
Nr0b2								-2.2
Nr1d1	-1.7				-2.8			
Nr1d2					-1.7	-1.7		
Nr1h3	2.2	-2.6			2.5			-1.7
Nr2f6					1.6			
Nr3c1_2			-2.2					
Nr3c2					-7.0			
Nr4a1		-1.9	-1.9		-2.2	-2.4	-2.9	-1.9
Nr4a3								2.7
Nrcam							-6.0	-3.8
Nrg1	2.6				6.6			
Nrg2	3.7				3.6			-2.0
Nrg4					24.8			
Nrgn							-2.5	-3.0
Nrip1					1.6			
Nrn1							-10.4	-6.0
Nrp1		-2.6				-2.5		
Nrp2	3.9				5.6			

Nrtn					3.0			
Nrxn2		-3.1			-16.1	-8.7	-5.8	-4.6
Ns5atp9	5.4				3.7	5.5		
Nsd1								1.8
Nsd2	2.4				1.8			
Nsg1	-2.1							
Nsl1	2.0							
Nsmce1		1.7						
Nt5c		1.6						
Nt5e			-1.9		-2.5		-2.1	-2.1
Ntm				-10.5	-27.6	-6.9		-7.2
Ntn1	3.7				6.3			
Ntng2								1.8
Ntsr1					-79.1		-6.2	
Nuak1	-2.0	1.5			-1.8			
Nuak2					1.9			
Nucb2		-1.8	-1.7	-1.6			-2.1	-2.0
Nudt2						1.6		
Nudt4							-1.8	
Nudt9					1.7			
Nuf2	4.8				3.2	3.8		
Numa1					-1.5			
Numbl		-2.7					-2.5	-2.4
Nup210						1.6		
Nup210l				-1.9				
Nusap1	4.6				2.8	3.9		
Nwd1	-2.6				-4.9		-3.0	
Nxpe1					-1.7			
Nxph3					-3.4			-3.0
Nxph4								-3.0
Nxt2	1.7							
Oaf		-2.0		-1.7	2.4		-2.3	-2.4
Oas1a					2.9			
Oas1b					2.7		-1.9	-1.7
Oas1i			-411.3			-332.6		
Oas2		-4.8				-3.6	-4.1	-3.2
Oas3		-2.4			2.4			-1.7
Oasl		-2.2			1.7		-2.5	-2.2
Oasl2					2.7			
Oat		-1.8					-1.6	
Obscn							-4.3	
Ogfr					1.7			
Ogfrl1		-1.8					-2.4	-1.8
Ogt						-1.8		
Oip5	3.5							
Oit3	2.8							
Olah	-2.0							
Olfm1	3.4	-2.9	-1.7		5.6			-1.9
Olfm4					3.8			
Olfml2a					-29.0	-5.6	-8.0	-6.0
Olfml3		4.0						
Olr1		-2.7					-2.6	-2.1

Olr1584	3.2				7.0			
Olr1587					2.6			
Olr1588					3.1			
Olr292_1	-3.2		-2.0		-4.9		-2.9	-2.0
Oma1	2.1				2.3			
Ophn1							-4.1	-3.4
Oplah		1.9						
Opn3					2.2		-2.5	-2.3
Orc1	6.5				3.8	3.9		
Orc3	1.7				1.9			
Orc6	1.8							
Orm1					3.9	12.5		
Oscar					2.5		-2.3	-2.1
Osgin1					1.8		-1.7	
Osm			-2.5				-3.8	-2.4
Ostm1		-1.8						
Otud1			-1.8			-2.5	-1.8	-1.6
Otud4								1.6
Otud7b								1.6
P2rx1					2.0		-2.3	-1.9
P2rx4					1.7			
P2ry1		-5.4	-2.1				-2.2	-2.1
P2ry10		-1.6			-1.9	-1.7		
P2ry12	2.4	-4.3			2.6		-3.2	-2.7
P2ry13	2.4	-3.1			3.2		-2.4	
P2ry14	2.2				2.9			
P2ry2		-4.5	-2.3	-2.2			-3.6	-2.6
P2ry6					2.2			-1.7
P3h3								-2.0
P4ha1	1.7	-2.3			2.4		-1.9	
P4ha2				-3.7		-4.1	-5.1	-4.9
P4hb					1.7			
Pacs1					-1.8			
Pacsin3					-3.5			
Padi2		-2.3	-1.9				-2.3	-2.5
Padi4					2.1		-2.2	-1.8
Pak1	1.6	-2.5			2.5		-2.2	-1.9
Pak6	-2.4				-2.7			
Pald1					-2.4			-2.5
Palld_2		-3.3			-2.4	-4.1	-2.7	
Palm							-2.0	-2.0
Palm3						3.0		
Pam							-2.9	-3.0
Pank3						-1.5		
Panx2							-2.4	
Papln	-3.1	-3.7			-3.7		-3.8	
Papss2	2.0	-2.8						
Paqr4	1.9							
Paqr6								-2.2
Paqr8	2.3				2.4			
Paqr9		3.3						
Pard3		-2.4				-2.7		

Pard6b						-2.1			
Parg							-1.6		
Parp10						1.9			
Parp12	1.6	-1.9				2.1			
Parp14	1.6					2.1			
Parp3	1.8					2.7			
Parp8						1.8			
Parp9	1.7					2.5			
Parpbp	4.9					3.2	3.7		
Parvb				-2.4				-3.6	-3.3
Parvg						1.7			
Pask	2.2								
Pax5	-1.9	2.6							
Pbk	5.1					2.9	2.6		
Pbx3	1.6					2.0			
Pc								-1.9	
Pced1b		1.6							
Pcf11							-1.5		
Pcgf2		2.2	2.0				2.1	1.8	2.3
Pcmtd2							-1.7		
Pcna	1.6						1.6		
PCOLCE2						-32.8			
Pcsk1						-2.2	-3.8		-2.0
Pcyox1		-2.8	-2.1					-1.9	
Pcyt1b									4.5
Pdcd1							2.3		
Pde2a	-1.6					-1.9			
Pde3a		-9.1						-4.0	-2.8
Pde3b						-1.8	-1.8		
Pde4a	1.9								
Pde4d						-1.6			
Pde5a		-9.2						-5.1	
Pde6d		1.5					1.6		
Pde6h	-2.0	2.6							
Pdgfa								-3.1	-3.3
Pdgfrl	7.6					11.0			
Pdia3						1.8			
Pdia4						1.9			
Pdia5	-1.8					-2.3		-1.5	
Pdia6						1.7			
Pdk3						1.8			
Pdlim1								-1.9	-2.0
Pdlim4				-5.3		-14.0	-5.9	-6.7	-4.7
Pdxk	1.5								
Pdzd3								-2.4	
Pdzd4	-2.0					-2.5			
Pdzd9				13.2			10.7		
Peak1									2.4
Peli2		-4.1	-3.4					-3.8	-2.5
Per1						-1.5			
Pet100						1.6			
Pf4						2.1		-3.6	-3.7

Pfdn1		1.5				1.6		
Pfdn4						1.8		
Pfkfb4		-2.3					-2.2	
Pgam1					1.7			
Pgam2	-5.3							
Pgap2		-1.8					-1.6	
Pgk1					1.7			
Pglyrp1					4.3		-2.2	-1.9
Pglyrp2					-1.8			
Pglyrp4	-3.2						-3.1	
Pgm2					1.6			
Pgm2l1					-1.8			
Phactr2	2.5				2.8			
Phc3								1.7
Phf1	-1.7							
Phf19	2.1							
Phf20l1_1		-1.9				-2.0		
Phgdh		1.6				2.0		
Phip								1.7
Phkg2		1.5						
Phldb1							-2.8	
Phlpp2								1.8
Phospho1	-1.7					2.2		
Phyhd1					2.7			
Pi16		-1.7					-2.1	
Pi4k2a			-1.6					
Pianp		3.7	6.1	4.2		4.6	9.4	15.4
Picalm					1.7			
Pid1		-3.4		-2.1		-2.6	-4.3	-3.3
Pif1	4.0							
Pigm		-1.9						
Pigr	-2.1							
Pigx						1.6		
Pigz_1	3.5				13.4			-5.1
Pigz_2		-6.9			9.0		-6.6	-4.7
Pik3ap1		-1.7						
Pik3c2a		-1.8						
Pik3c2g		-15.3						-6.8
Pik3ip1	-1.7				-2.1			
Pik3r6							-2.0	-1.8
Pikfyve						-1.6		
Pim1	1.6				2.2		-1.8	
Pim2		1.6						
Pink1								-1.5
Pip4k2b								1.5
Pipox							-4.5	
Pir		-3.7				-3.0	-2.7	
Piwil4					2.6			
Pkdcc	2.0				2.3			-2.1
Pkia	2.1							
Pkib								-1.6
Pkig	3.2				4.4			

Pkm		-1.8	-1.6		1.6		-1.9	-1.6
Pkmyt1	1.7	1.7				1.7		
Pknox2	-2.1				-2.3			
Pla1a							-4.2	-3.4
Pla2g16	1.8				2.2	1.7		
Pla2g2a							-4.6	-3.4
Pla2g2d	2.2				2.8			
Pla2g2f		2.1	2.1		-2.8		2.1	2.8
Pla2g4a	2.5	-2.9			3.3			-1.7
Pla2g6		1.6						
Pla2g7		-1.8					-2.6	-2.0
Plau	3.1			-4.3	5.9		-4.7	-9.9
Plaur		-1.8			1.6		-2.5	-1.8
Plbd1		-1.6	-1.5				-1.9	-2.0
Plcb1		-8.0	-4.1	-2.9			-4.7	-2.6
Plcb2							-1.8	-1.5
Plcb3	1.6	-1.8			2.3		-1.6	
Plcd1							-1.6	
Plcg1					-1.8			1.5
Plch2					-2.7			
Plcl1					-3.1			
Pld1		-3.2	-1.9	-1.7	2.1		-2.6	-1.9
Pld3					-1.8			
Pld4		-2.4	-1.6		1.7		-2.2	-1.9
Pld6					-4.5	-4.8		
Plek	1.6				2.1			
Plekha5					1.8			
Plekha7					-1.9			
Plekhb1		3.1		-2.8	2.4	5.2		
Plekhb2		-1.6			1.5		-1.8	
Plekhf1					-2.1			
Plekhg1	-4.0				-5.9			
Plekhg2								1.5
Plekhg3		-1.9					-1.9	
Plekhg5			1.6					
Plekhm3			-3.1					
Plekho1								-1.6
Plekho2	2.6				2.4			
Plin2		-2.3			1.6		-2.2	-1.7
Plk1	4.2				2.8	4.3		
Plk2				-6.3	-6.1	-6.4	-12.7	-8.2
Plk3							-2.1	
Plk4	3.3				2.0			
Plk5	3.9				2.2			
Plod2								-2.7
Plp1		-2.1				-3.0	-2.5	-2.0
Plp2						1.6		
Plppr3		3.0			-4.1			
Pls1								-1.7
Pltp				-4.5	-5.9	-4.7	-6.0	-5.6
Plvap		-2.6	-1.8				-2.6	-2.6
Plxdc2		-2.5	-1.9	-2.1			-2.6	-2.2

Plxna1	1.6					1.8			
Plxna2						-5.2			-3.0
Plxna3		-3.1						-3.2	
Plxnb2		-2.6	-1.8	-1.6				-2.6	-1.8
Plxnd1							1.7		2.5
Pmaip1		-1.8						-1.8	-1.8
Pmf1	1.7						2.0		
Pml	1.6								
Pmp22				-2.4		-15.3	-4.9	-5.6	-3.9
Pnisr							-1.7		
Pnmal2						-7.7		-9.3	
Pnp	2.5					3.5			
Pnpla2						1.5			
Pnpla8		-1.5					-1.6		
Poc1a	2.2						2.7		
Podnl1	-1.8					-2.2			
Pola1	2.1								
Pole	1.6						1.6	1.5	1.8
Pole2	2.5					1.9	2.2		
Pole3		-17.9							
Polg2	-1.6								
Polq	3.7								
Polr2a							1.7		1.5
Polr2l	3.4	-348.1		-401.3			-313.8		
Pomgnt2				-5.6		-4.6		-5.1	-4.3
Pomp						2.2			
Pomt2						-1.6			
Pon1		-2.5					-2.5		
Pon3	-3.0	-2.8				-4.0	-3.0	-2.8	-1.7
Pop5						-1.6			
Popdc2				-2.0		-6.4	-2.9	-4.0	-2.3
Porcn							-2.3	-2.7	
Pot1b	-1.6								
Pou2af1	-1.6								
Pou6f1						-2.4			
Pp2d1				11.7					5.2
Ppard	1.5					3.0			
Pparg	-2.0	-2.8				-2.5	-4.5	-3.6	-2.2
Ppargc1b	2.0					2.7			
Ppbp								-3.2	-3.6
Ppdpf	-2.0								
Ppef1			-2.6					-2.6	
Ppial4d				-2.0		2.1			-3.1
Ppih	1.5						1.8		
Ppil1							1.5		
Ppm1h							-1.9		
Ppm1j						-2.0			
Ppox						1.6			
Ppp1cb		-2.0					-1.6		
Ppp1r11							1.5		
Ppp1r12b									2.3
Ppp1r14a									-4.4

Ppp1r14c					-2.5			
Ppp1r15a			-1.9				-2.8	-1.6
Ppp1r36	2.0							
Ppp1r3b					2.2			
Ppp1r3d		-2.0			1.8		-2.1	-1.8
Ppt2	2.0				3.0			
Pqlc1							-1.8	-1.6
Pqlc3		-2.1		-1.6				
Pram1					4.1		-2.2	-2.1
Prc1	3.3				2.4	1.9		
Prcp		-1.7					-1.5	
Prdm1	1.5		2.0	1.6		2.1	2.6	2.8
Prdx1	1.5				1.9			
Prdx2		1.6			2.3	2.2		
Prdx5	1.8	-1.5			3.0		-1.6	-1.8
Prelid2					-1.9			
Prf1	1.9							
Prg2					3.0	4.0		
Prg4				-4.1	-3.0		-4.7	-3.9
Prim1	1.7					1.7		
Prkag2						-1.7		
Prkcdbp		5.0	5.0			6.0		
Prkd1	-2.9	-2.5			-4.4		-4.0	
Prkd3								1.5
Prkx	1.6							
Prlhr					11.4			
Prmt2								1.5
Prnp	-1.9				-2.5			1.9
Procr								-2.7
Prodh1		-2.2				-2.0	-2.2	-2.0
Prok2	-2.3						-3.7	
Pros1					-3.1	-2.4		
Prox1				-27.8	-14.8	-13.5	-8.6	-12.0
Prpf39						-1.7		
Prpf4b						-1.7		
Prr11	6.4				3.1	3.6		
Prr16						-7.2		
Prr5	2.0					1.8	1.6	
Prr5l	2.4				3.5			
Prrc2b								1.7
Prrg1		-8.9		-2.7				
Prrt1	-1.6				-1.7			
Prrt2						-3.1		
Prss3		9.5				18.4		
Prss34						4.7		
Prss35	5.1				8.7			
Prss39		2.1	1.8					
Prss3b					-2.6			
Prtfdc1							-2.5	-2.8
Psap		-2.3	-1.7	-1.5	1.6		-2.1	-2.0
Psat1	2.5				1.8	3.4	1.9	1.5
Psca					-13.7	-3.9		

Psd						-3.1			
Psd3				-3.9				-4.1	-2.8
Psen2	2.0					2.5			
Psma2						1.6	1.7		
Psma5	1.6					2.0	1.6		
Psma7						1.6			
Psmb1							1.5		
Psmb10						2.0	1.7		
Psmb2						1.7	1.6		
Psmb3							1.6		
Psmb4							1.5		
Psmb8	1.6					2.7	1.7		
Psmb9	2.2					3.7	2.2	1.6	
Psmd8						1.6			
Psme1						1.7	1.6		
Psme2	2.2					3.3	2.0		
Psmf1	1.8					2.3	2.0		
Pstpip1						1.7			
Pstpip2	2.2	-1.8				3.7			
Ptafr		-2.0				1.9		-2.6	-1.8
Ptbp3			-1.7						
Ptch2						6.8			
Ptcra		5.8							
Pten		-1.7							
Pter	1.9					2.8			
Ptger1									2.9
Ptger3	3.5					3.7	2.1		
Ptger4						-1.7			
Ptges	5.5					8.2			
Ptges3l						2.7			
Ptgfrn									-1.9
Ptgir		-2.4						-2.2	-1.6
Ptgis				-7.6		-27.5		-7.2	
Ptgr1		-3.3				2.7			-3.0
Ptgs1	2.9			-2.0		4.5		-2.8	-3.0
Ptgs2			-3.2			2.9		-3.3	-3.1
Ptk2	-1.8					-3.2	-2.8	-2.3	-1.9
Ptk7								-2.9	
Ptp4a1		-1.7							
Ptp4a3	-1.8	1.6							
Ptpn1						1.5			
Ptpn12		-1.7							
Ptpn13								-3.2	-2.1
Ptpn22						-1.7			
Ptpn4	1.5							1.6	
Ptpn5	3.6					2.3			2.3
Ptprcap		1.7					1.8		
Ptpre		-1.6							
Ptprg						-7.4			5.1
Ptprj	1.7	-1.9				2.0		-1.5	
Ptprk						-2.4			
Ptprm		-2.2		-1.9				-2.3	-2.0

Ptprn		5.8							
Ptpro		-2.7						-2.1	-1.7
Ptprs						-2.5	-2.2		
Ptpru						5.7			
Ptrf				-5.0		-20.0	-6.2	-6.3	-4.6
Ptrhd1							2.0		
Pttg1	2.7	2.5				2.9	5.4		
Purb		-1.7							
Pvt1									1.7
Pwwp2b	1.6	-2.7						-2.3	-1.8
Pxdc1	-2.1	1.6							
Pxylp1								1.5	
Pygl						1.9			
Pym1		1.6							
Qpct	2.5					2.7			
Qprt	-2.0					-2.4			
Qrfpr	-3.4	-3.1	-1.8			-6.9	-5.4	-4.2	-2.0
Rab11fip5		-3.2	-3.0	-2.2		-2.7		-4.1	-2.7
Rab13	2.8	-3.3		-2.1		4.3		-2.5	-2.7
Rab19							1.7	1.5	
Rab1a						1.8			
Rab20	1.9					2.8			
Rab24		1.5							
Rab29			1.5						
Rab30	-1.9					-1.8			
Rab31		-1.5						-1.9	-1.8
Rab32	2.5	-2.7				4.0		-2.0	-2.1
Rab37						-1.7			
Rab38		-3.9						-3.2	-3.2
Rab39a	2.4					3.4			
Rab3il1							3.4		
Rab3ip						-1.7			
Rab43	1.8		-1.9						
Rab44						3.8		-2.2	-2.3
Rab7b		-3.4	-2.1			-2.6		-3.2	-2.4
Racgap1	4.4					2.5	3.2		
Rad18	1.9								
Rad51	4.1					2.7	2.8		
Rad51ap1	2.1								
Rad51b	3.4								
Rad54b	3.1								
Rad54l	3.1					2.1	2.4		
Raet1d	4.0					3.2			
Raet1l	2.5								
Rag1	11.1	34.1				5.0			
Rag2		14.4				4.9			
Rai1						-1.5			
Ralb		-1.7				1.6		-1.8	
Ralgapa1									1.6
Ralgds	2.7					7.8			
Ralgps2	-1.7					-2.3	-1.7		
Ramp2		3.2	2.9				5.9	2.5	2.8

Ramp3	-2.1								
Ran	1.6						1.7		
Ranbp9		-1.6					-1.6		
Rap1b		-1.7							
Rap2a		-1.7							
Rapgef5						-2.8			
Rapgefl1	-2.4					-3.4			
Rarb						-4.1			-2.7
Rarg		-1.6						-2.0	
Rarres1				-3.9				-4.2	-3.3
Rasal2								-6.3	
Rasgrp3									-1.6
Rasgrp4		-2.6				2.0		-2.5	
Rasl10a	2.6								
Rasl11b						-2.5			
Rassf4	2.4	-2.3				2.9		-1.7	-1.7
Rassf6						-2.6			-2.4
RatNP-3b		8.8				6.6		7.2	
Rbfox2	3.3								
Rbl1	1.7								
Rbl2						-1.7			
Rbm20						2.0		-2.0	
Rbm26							-1.6		
Rbm43						1.5			
Rbm47		-3.3						-2.8	
Rbpj_1		-1.8							
Rbpjl	-2.4								
Rbpms									-1.5
Rcan1	1.5					1.6			
Rcc1	1.5						1.6		
Rcn1	1.7								
Rcn3	-2.4					-2.8		-1.8	-1.7
Rdm1		1.7							
Rec114		3.5				2.5	4.0		
Reep2								-3.2	-3.4
Reep3		-1.6							
Reep6	-2.3					-2.0			
Rell1		-1.6					-1.6	-1.5	
Renbp	1.7					2.1			
Reps2			-16.8						
Rere						1.6			
Retnlg		-2.4				3.3		-2.8	-2.7
Rfc3							1.7		
Rflna						-2.8			-2.0
Rflnb						-1.7			
Rfx2		-3.0		-2.2		-3.0	-3.1	-4.0	-2.3
Rfxap							1.6		
Rgcc						-1.9			
RGD1304694		-2.0	-1.5				-1.7	-2.0	-1.6
RGD1304810		4.2				3.5	8.5		
RGD1304884		-3.3	-2.2					-3.1	-1.9
RGD1305184	1310.7					2839.4	185.7	299.9	53.1

RGD1305464					-1.6			
RGD1305807					-3.6			-3.2
RGD1306556	1.8							
RGD1306739	-2.5				-2.0			
RGD1307100								1.7
RGD1307182							-2.7	-2.5
RGD1307461			2.1	2.6		2.7	3.2	4.0
RGD1308065	4.6	2.8			4.1	5.3		
RGD1308117								1.6
RGD1308564						3.7		
RGD1308751	5.2							
RGD1309079		-1.7						
RGD1309350	2.3	1.9			2.3	3.3	1.9	
RGD1309594	1.6				1.6			
RGD1309808	1.8				2.1			1.5
RGD1309995						-2.0		
RGD1310081						-2.9		
RGD1310335	4.7				2.5	2.7		
RGD1310507		2.1		-2.4		3.6		
RGD1310819				-3.6	-4.7	-4.5	-4.9	-4.1
RGD1311084					2.7			
RGD1311575		-3.5				-2.7	-4.0	-2.4
RGD1311595								1.5
RGD1311739		1.7			1.8	1.8		-1.5
RGD1311744	4.8				5.4			
RGD1311756	2.1	-5.0		-2.2	2.6		-2.8	-2.3
RGD1311946							-2.0	
RGD1559482		-3.6	-2.0	-1.8			-4.0	-2.7
RGD1559588_1	-2.0						-3.0	
RGD1559662						2.8		-2.1
RGD1559962	2.9					4.9		
RGD1559972		3.1				3.6		
RGD1560281	-1.7	-2.8	-1.7		-2.1	-2.5	-3.7	-1.7
RGD1560455					-1.7			
RGD1560470								1.8
RGD1561143	-1.7	-1.7			-2.1		-2.4	-1.5
RGD1561149					-3.0	-2.9	-2.6	-2.6
RGD1561157					1.9			
RGD1561252						192.8		55.1
RGD1561277					-2.0			
RGD1561662		-3.2		-3.7	-3.9	-4.0	-6.0	-4.6
RGD1561730	-1.9	-2.4	-1.9		-1.8		-4.3	-1.8
RGD1561778		-3.7	-2.0	-1.6			-3.0	-1.9
RGD1562652					1.8			
RGD1562667	2.1					1.8		
RGD1562758					2.6			
RGD1563091	2.8	-2.2			4.5			
RGD1563354	-3.2	-2.7			-5.9	-3.3	-2.3	
RGD1563818		-17.1		-22.9		-13.1		-22.2
RGD1564129							-4.4	-2.9
RGD1564571					-8.3			
RGD1564606						2.3		

RGD1564664	-2.2				-2.4			
RGD1565033					-1.9			
RGD1565048								-3.6
RGD1565355				-2.7	2.7			-3.0
RGD1565410	7.2							
RGD1565536					-3.5			-4.3
RGD1565641						1.7		
RGD1565844							-5.4	
RGD1565989					-2.2	-2.4		
RGD1566029					-3.2			
RGD1566138				-3.5		-3.7		-2.6
RGD1566226	-2.5	-3.2			-3.3			
RGD1566368			-3.6		-6.5	-10.2	-7.8	-3.1
Rgl1					-4.6	-3.5	-2.4	-1.8
Rgs12	-1.5				-1.9			
Rgs18		-3.1	-1.9				-2.5	-1.9
Rgs2			-2.6	-1.8		-1.8	-3.8	-2.8
Rgs5					3.7			
Rgs6							-2.7	
Rhag							-2.8	-2.9
Rhbdf1	-2.1				-3.8	-2.5	-3.2	
Rhbdf2					2.2			
Rhbdl1								2.5
Rhbdl3							-4.5	-3.3
Rhbg	2.0				2.7			
Rhd		2.9		-2.8	2.7	5.0		
Rhob			-2.2	-1.8		-2.3	-4.1	-2.3
Rhoc	-2.1				-3.1	-1.7	-2.2	-2.0
Rhoh	-1.5							
Rhoq		-1.8				-1.8		
Rhou								6.9
Rhov		-1.7					-1.9	-1.8
Rhox5	2.9				4.7			
Rhpn2								-2.9
Rictor								2.2
Rilp							-2.1	-1.8
Rims2					3.1			
Rin2		-3.3	-1.6	-1.6			-3.1	-2.3
Rinl						1.6		
Ripk1					1.5			
Ripk2	1.6				2.3			
Ripk3	2.1				3.7	1.7		
Rmdn3					2.3			
Rmi2	13.5				14.7	7.7		
Rn50_10_0706.2	-2.0				-2.3			
Rn50_10_0890.5		7.3						
Rn50_10_0892.1						-2.6		
Rn50_13_0834.1								2.1
Rn50_20_0026.4								-3.2
Rn50_4_1360.2		-8.4						
Rn50_5_0814.26					-2.4			
Rn50_7_1163.2					3.0			

Rn50_X_0744.5	953.9								
Rn50_X_0752.3							-3.4		2.3
Rn60_1_2212.3								-2.3	
Rn60_10_0648.2	2.5					17.5	3.0		
Rn60_10_0910.5		16.5		19.0			10.3		12.3
Rn60_19_0375.6		10.3		8.2			6.7		7.2
Rn60_2_1868.2	-2.0								
Rn60_20_0030.4	-2.9					-2.8			-5.1
Rn60_20_0051.7						12.4			
Rn60_20_0053.4						-4.3			
Rn60_20_0069.2	2.7	-4.9				4.1			
Rn60_20_0141.4	2.4					5.3	2.8		
Rn60_7_0127.2		10.4		18.8			14.1		11.5
Rn60_7_0127.5				10.8			7.6		5.7
Rnase2	2.0	-9.3		-2.1					-3.4
Rnase4				-2.6				-3.0	-2.8
Rnaseh2a						1.7			
Rnasek						1.6			
Rnasel	1.5					2.6			
Rnaset2	1.7					2.2			
Rnd3	1.9	-2.2				2.2			
Rnf10							1.8		
Rnf103							-1.6		
Rnf112								-2.9	
Rnf114	1.8					2.7			
Rnf11l1								-1.9	-1.7
Rnf125						-1.7			
Rnf128	2.4	-3.8		-2.3		2.5			-2.6
Rnf13		-1.6							
rnf141		-2.3	-1.9						
Rnf144a	-1.7								
Rnf144b	1.7	-1.8				1.8			
Rnf149		-3.1	-1.8				-2.2	-2.0	-1.5
Rnf150						-2.5		-2.2	
Rnf166									-1.7
Rnf19b	1.5					1.8		1.6	1.8
Rnf2						-1.5			
Rnf213	2.3					2.9		1.7	1.8
Rnf39						-7.7			
Rnf43								1.9	2.4
Rnh1						1.6			
Rnpep	1.5					1.7			
Rock1		-1.6							
Rora									3.6
Rorc	-2.4					-6.1	2.1		
Rp2		-2.1							
Rpa1						-1.5			
Rpa3	1.6						2.0		
Rpe		-1.5							
Rph3al	-1.6					-1.8			
Rpl13	-2.5		-3.3						
Rpl26-ps1		3.5		4.3			3.5		2.5

Rpl28		1.6							
Rpl31l4		2.6		1.8			2.1		
Rpl32_2							7097.3		
Rpl34-ps1		1.7							
Rpl35al1		-358.1	-403.3			-350.8	-34.4	-51.5	-410.7
Rpl3l									-2.3
Rpl5l1		-3.0		-2.6					-2.6
Rpl6-ps1		1.6							
Rpl9				-1.9					-1.9
Rplp2		1.8							
Rpp21		1.6							
Rpp25	2.5					2.6		-3.8	-3.0
Rps10l1		1.7							
Rps15		1.6							
Rps19	-1.5		-1.5			-1.9	-1.6	-1.7	
Rps27l						1.7	1.8		
Rps4x_1						-111.9			-133.6
Rps4y2	1.9					2.0	2.5		
Rps6_1		-10.4	-78.0				-63.2	-10.9	
Rps6ka2		-1.7					-1.7	-1.8	
Rps6ka3		-1.6							
Rps6kc1						1.5			
Rpusd3	-1.7								
Rrbp1	1.5	-1.6				1.7			
Rrm1	1.9						1.5		
Rrp1b									1.9
Rsad2		2.0				2.7	2.3		-2.1
Rsbn1							-1.7		
Rsph1						3.0			
Rspo1								-2.2	-1.8
RT1-A1						1.7			
RT1-A2						1.6			
RT1-Ba	1.6					2.0	1.7		
RT1-Bb	1.7					2.1			
RT1-CE1	1.8								
RT1-CE10						1.7			
RT1-CE16_2						1.6			
RT1-CE4						1.6			
RT1-CE5						1.7			
RT1-CE6						1.7			
RT1-Da	1.7					2.1	1.7		
RT1-Db1	1.6					2.1			
RT1-Db2	-2.3					-3.0			
RT1-DMb_1	1.6					1.9			-1.7
RT1-DMb_2						1.9			-1.8
RT1-DOa	-1.8	1.9							
RT1-DOb	-1.7					-1.5			
RT1-Ha	-2.2								
RT1-M2	4.4								
RT1-N3	2.0					2.6			
RT1-S2						2.3			
RT1-S3	1.7					2.1			

RT1-T24-3	1.6				2.4			
RT1-T24-4					1.8			-1.5
Rtkn2	4.7							
Rtn1	-3.5				-16.0			
Rtn4		-1.8						
Rtn4rl1	-1.9				-1.8			
Rtp3		-3.2					-3.7	
Rtp4	1.9				3.8	1.9		
Rufy4					3.5		-2.5	
Runx3								1.6
Rusc2		-2.5	-2.7				-3.6	-2.2
Rxra		-3.3	-2.2				-2.2	
Ryr2					-7.8			
S100a11					2.3		-1.9	-1.7
S100a3						3.0		
S100a4		-2.1		-1.5			-2.5	-2.2
S100a6		-1.9					-2.1	-2.0
S100a8					5.5		-2.0	-1.8
S100a9					4.9		-2.1	-1.8
S1pr1					-1.9			
S1pr2	1.6				2.0			
S1pr4	-1.6							
S1pr5					-2.3			1.8
Saa4					1.8			
Sacs							1.6	1.8
Samd3		2.2	2.2				2.2	2.1
Samhd1	2.0	-2.7	-1.7		1.8			
Samsn1		-2.3	-1.5				-1.8	
Sap25		1.8	1.6					
Sapcd2	4.7				3.2	4.5		
Sardh								-3.0
Sash1					-6.0	-4.0	-4.6	-3.3
Sat1					1.5		-1.5	-1.6
Satb1					-1.7			
Sbf2		-2.4	-1.7				-2.0	
Sbno2					2.7			
Sbspon								2.6
Scamp1	1.5	-1.8		-1.5	1.9		-1.7	-1.9
Scamp5							-4.0	-3.1
Scaper					-1.7			
Scarb1	1.9	-2.2	-1.6		2.2		-1.8	-1.6
Scarb2						-1.6		
Scarf1	3.8				5.2			-2.4
Sccpdh				-60.0		-268.4		-339.8
Scfd1					1.7			
Scgb3a1	-4.0						-3.0	
Scimp	2.5				5.6			-1.7
Scin	2.5		-1.9					-1.8
Scly					2.1			
Scml2	3.4							
Scml4					-1.6			
Scn1b				-2.6	-8.7	-3.6	-4.2	-3.2

Scn4a	-2.7				-2.5			
Scn5a					-4.2			
Sct					7.7			
Scube1					3.0			
Sdc1	1.8					2.1		
Sdc3				-2.2				-2.4
Sdcbp		-1.5			1.7			
Sdf2l1					2.2			
Sdhb					1.7			
Sdk2	-2.6				-7.3	-2.7	-3.2	-3.8
Sdpr							-5.5	-4.4
Sec11c					1.8			
Sec14l5		-6.6					-4.5	
Sec22a					-1.6			
Sec61b					1.7			
Sec61g		1.6			1.8	2.2		
Secisbp2l		-1.7				-1.7		
Sectm1a	22.8				45.2	3.9		
Sectm1b	2.5				3.3	3.1		
Selenbp1		57.3			99.8	61.8		
Selenoh						1.5		
Selenoi								1.7
Selenon	1.9		-2.5				-2.5	
Selenop		-2.2	-1.8		-3.6	-2.2	-2.2	-1.8
Selp				-4.4	-13.2	-4.3	-5.8	-4.8
Sema3c		-10.6			5.3		-4.9	-5.7
Sema4a					1.9			1.6
Sema4c								-2.7
Sema6b					1.9		-1.9	
Sema6d						-3.2	-3.5	
Sema7a			1.6	1.7		1.6	2.1	2.3
Semg1	-3.3				-4.7	-5.3	-3.8	
Sept10		-4.3						
Sept11	1.6							
Serf1	2.6				2.9			
Serinc1		-1.8						
Serinc2					2.3			
Serinc3		-1.7						
Serpinb10		-3.1		-3.5			-4.1	-3.7
Serpinb11		-2.6				-2.5	-3.9	-3.8
Serpinb1a	1.6	-2.0		-2.2	6.1		-3.0	-2.8
Serpinb6		-1.9	-1.7			-2.8	-4.2	-2.8
Serpinb6b		1.7				1.8		
Serpinb8		-2.7		-2.1	-6.5	-2.6	-4.1	-3.0
Serpinb9	1.6					2.6	2.0	2.1
Serping1	21.2				70.3	3.5		
Sertm1					-6.1			-3.5
Sesn1	-1.7				-2.2			
Sestd1								-3.4
Setd1b								1.8
Setx					-1.5	-1.8		1.6
Sfmbt1	1.6							

Sfmbt2	5.1				9.1			
Sfn			1.6					
Sftpb					4.0			
Sftpd							-5.4	-4.3
Sfxn3					-1.8			
Sfxn5							-2.2	
Sgcb		-2.2						
Sgk3		-2.1						
Sgms1					-1.5	-1.8		
Sgms2	1.7	-2.8			2.2		-2.4	
Sgo1	4.0				2.6	2.4		
Sgo2	4.5				3.0	3.0		
Sgpp1	1.6	-1.9			1.9			-1.5
Sgpp2	-7.7				-4.2	-6.9		
Sgsm1					-15.6			
Sh2b2	-2.3				-2.2			
Sh2d1a						1.7		
Sh2d1b		-2.6	-2.0				-2.2	
Sh2d1b2							-3.0	
Sh2d2a		1.8	1.7			2.2	1.8	1.8
Sh2d4a								-5.4
Sh2d5								-1.9
Sh3bgrl3					1.5	1.6		
Sh3bp1	1.5				1.6			
Sh3bp2					1.5			
Sh3bp4		-2.1			-1.8	-2.1	-2.5	-2.1
Sh3bp5	-1.8				-1.5			
Sh3gl3					-4.4			
Sh3pxd2b		-17.3	-9.1				-3.7	
Sh3yl1					2.2			-1.9
Shank3				-6.5	-10.9	-7.3	-5.5	-6.8
Shc2							-2.6	
Shcbp1	5.2				3.3	3.3		
She					-3.7	-2.1		
Shisa4	3.9				4.3			
Shtn1					-10.3			-4.0
Sidt1							1.6	1.6
Sidt2					2.0			
Sigirr		1.6						
Siglec1	4.9	-11.6			7.9		-3.5	-3.6
Siglec10	-1.5	1.6						
Siglec5							-2.7	-1.8
Siglec8		-2.1					-3.3	-1.9
Siglech		-4.5	-4.6	-3.1			-8.8	-4.1
Sik1			-2.5	-2.2		-2.7	-3.6	-2.6
Sik2						-1.6		
Sipa1l2		-1.9					-2.3	-1.8
Sirpa		-1.9					-2.1	-1.6
Sirt2						1.7		
Sirt4					-1.8			
Sit1	-1.7				-1.8	1.7		
Six1					5.3			

Ska1	5.3					3.7	4.6		
Ska2	2.1					1.7	1.9		
Ska3	3.8					2.7	3.4		
Skap1						-1.7			
Skap2		-1.7				1.8		-1.6	
Skil							-2.2		
Sla2									1.8
Slamf1							1.8		1.8
Slamf6	-1.6					-1.7			
Slamf7			-2.6						
Slamf8	8.0	-3.4				11.9			-1.8
Slamf9		-3.9		-2.4				-2.9	-4.2
Slbp	1.6						1.6		
Slc11a1	1.6	-2.0				2.3		-1.8	-1.6
Slc12a3	-2.1					-2.4			
Slc13a5						-4.0		-4.6	
Slc14a1						-1.7			
Slc15a3		-1.8				1.7		-1.9	-1.7
Slc16a2						-8.6			
Slc16a3		-1.6						-2.5	-1.8
Slc16a7				-3.3		-2.7			-2.5
Slc17a7							57.6		
Slc18a2		-3.3						-3.0	-2.8
Slc19a1						1.7			
Slc1a3	295.7					1180.9			
Slc1a4						1.7			
Slc22a15								-2.4	-1.8
Slc22a17	-1.5								
Slc22a23								-3.1	-3.0
Slc22a3							3.1		
Slc24a3		-3.1						-3.1	
Slc24a4									-4.6
Slc25a13		-2.9							
Slc25a16		-1.9				1.7			
Slc25a23						-2.2			
Slc25a24		-2.1		-1.5					
Slc25a27	3.5					4.4			
Slc25a28	1.6					2.7			
Slc25a36							-2.1		
Slc25a39		2.0					2.2		
Slc25a4							1.8		
Slc27a2						3.6			2.6
Slc27a3		-2.4					-2.5		-2.4
Slc28a2		-1.5					-1.7	-1.6	
Slc29a1						-2.0			
Slc29a2							15.5		
Slc29a3	1.7					2.8		-1.8	-1.6
Slc2a1						2.0			
Slc2a10						-17.0	-8.0		
Slc2a3							1.8		
Slc2a4								-4.1	-3.6
Slc2a6	1.7	-1.7				2.0			-1.6

Slc2a8						1.6			
Slc2a9	1.6					1.8			
Slc30a1	1.5					1.8			
Slc30a9							-1.6		
Slc32a1								-3.3	
Slc35a1						1.6			
Slc35a5						2.1			
Slc35b1						1.7			
Slc35e3		-1.8						-1.6	
Slc35f5	1.9					2.6			
Slc36a2	-5.6			-9.8		-78.2	-10.1	-13.4	-15.4
Slc36a3		-2.9							
Slc37a1						1.6			
Slc37a2		-2.0				1.6		-2.5	-1.6
Slc37a3						1.5			
Slc38a5		3.4		-2.7			3.4		
Slc38a6	2.2					3.0			
Slc38a7	1.7					2.9			
Slc39a14									1.5
Slc39a8	2.0					2.4			
Slc40a1_1							9.9		6.5
Slc40a1_2		-2.5						-3.8	-2.5
Slc41a2	4.5					7.9			
Slc43a1	-1.7					-2.3			
Slc43a3								-2.3	
Slc44a1		-2.3		-1.9				-2.4	-2.2
Slc44a4								9.0	11.7
Slc45a3	-2.3					-2.0			
Slc45a4	1.9					2.4			
Slc4a1		3.0		-3.0		2.5	6.1	2.2	
Slc4a11						-1.7			
Slc4a7									2.3
Slc4a8									4.7
Slc6a4								-3.6	-2.9
Slc6a6		-2.6	-1.9						
Slc6a9						2.3			
Slc7a2	3.8					6.9			
Slc7a7				-1.8				-2.1	-2.1
Slc7a8		-2.9		-1.7				-3.0	-2.3
Slc8a1	3.4	-3.7	-2.8			4.2			
Slc8b1						2.0			
Slc9a3r1							1.6		
Slc9a3r2						-14.2	-4.6	-6.4	
Slc9a9						-1.7			
Slco1a2									2.4
Slco2b1						5.2			
Slfn13			1.6			1.8			
Slfn14						2.6	3.9		-2.5
Slfn4		-1.7				2.9	-1.8		
Slirp						1.7	1.9		
Slk		-2.7	-1.7						
Slpi								-3.3	-3.7

Slpil2								-4.4	-4.1
Smad1	2.0					2.4			
Smad3						-1.5			
Smad4							-1.6		
Smad6	4.1					5.6			
Smad9	2.3	-3.1				3.6			
Smagp		-1.9				-1.5		-2.4	-1.7
Smarca2						-1.6	-1.7		
Smarcd3	2.5					6.0			
Smc2	2.5								
Smg1									1.9
Smim5		-3.2	-2.5			-2.4		-3.8	-3.0
Smpd3		-5.2	-3.1					-2.9	-2.2
Smpd5		2.1	1.8						
Smpdl3a		-2.8	-1.9	-1.9				-2.7	-2.4
Smpdl3b		-2.5	-1.9	-1.7				-3.0	-2.6
Smtn	2.1								
Smtnl2	-2.6								
Snai1						2.0			
Snca		3.4				3.2	4.1	2.3	
Snd1						1.5			
Snf8						1.5			
Snn	-1.6					-1.6			
Snrnp25		1.6							
Snrpa	1.5					1.8	2.0		
Snrpc							1.6		
Snrpd1	1.6					1.6	1.8		
Snrpd2		1.6							
Snrpg				190.0					
Snta1	-2.7					-3.6	-3.6	-3.2	-2.0
Sntb1	1.9								
Sntg2						4.9			
Snx10	2.9	-2.1				3.2			
Snx18		-2.1					-1.8	-1.8	-1.7
Snx21									-1.7
Snx22						-3.5			
Snx24	4.8	-3.2				8.3			-2.7
Snx8	-1.6	1.6							
Soat1		-3.4	-1.8					-2.4	-1.5
Soat2	-3.0					-3.1	-8.3	-3.6	
Socs1	4.7					13.7	1.9	2.0	
Socs3			-2.7			2.6		-3.6	-2.4
Sod1						1.8			
Sod2	2.1	-1.7				4.2			-1.6
Soga1			-4.4						
Sort1		-1.9				1.6		-2.4	-1.6
Sos1									1.6
Sost						-13.8		-16.6	-15.4
Sowahc	2.2	-5.3	-2.9	-2.4				-4.4	-2.6
Sox13		-2.1						-2.5	-2.1
Sox15	-2.2								
Sox6							3.8		

Sp110					1.7			
Sp140					1.7			
Spaca9					-7.3			
Spag5_2	4.2				2.7	3.3		
Sparc					-2.2			
Spata13							1.6	1.6
Spata18	-11.0	-4.9			-34.4		-7.2	
Spata6					-2.1			
Spc24	3.7				2.8	3.1		
Spc25	3.7				2.6	3.1		
Spdl1	1.8							
Spdya					-4.3			
Specc1					1.8	2.5		
Speg					-2.0			
Spen								1.7
Spetex-2F							-5.8	-5.2
Spg20		-1.8				-1.7	-1.6	
Sphk1	-1.7				-2.1			
Spi1		-1.5			1.9		-1.8	-1.7
Spic	2.3	-4.2			3.1			-2.0
Spink2		135.8	166.0	193.9	112.7	976.2	654.8	698.1
Spint1			1.6		-1.9		1.7	1.5
Spn	-1.6				-2.6			
Spns3	1.9	-2.1			2.1			-1.8
Spock2						-4.1		
Sppl2a		-1.5			1.7			
Spr						1.6		
Spred1	2.0	-2.7				-2.6		
Spred3					4.0			
Spry4								-2.5
Spryd7								-1.9
Spta1					2.6	3.4		
Sptb		2.5		-2.5	2.3	3.5		
Sptbn2					-42.1		-12.6	-11.4
Sptbn4			2.8	2.3			2.9	5.0
Sqrdl		-2.2	-1.5				-2.2	-1.9
Srcap								1.6
Srcin1					-2.0	-2.4		1.9
Srgap1					-72.6			
Srgap2		-1.7					-1.6	
Srms	-3.6				-4.2			
Srpk3	-2.0							
Srrm2								1.7
Srxn1		-5.7						
Ssbp3					-1.6			
Ssc5d	3.0				5.5			
Ssfa2		-1.6						
Ssh3	1.6				2.0			
Sspn					-2.3	-2.4		
Sspo	-3.8				-4.2	-8.6	-2.5	
Ssr2						1.6		
Ssr3					1.8			

Sstr3		1.6							
Sstr4		-7.2						-4.8	
St14		-2.6	-2.2	-2.3				-3.5	-3.3
St3gal2									-1.9
St3gal4						1.7			
St6gal1						-1.7			
St6galnac4						1.6			
St6galnac5						-6.0			
ST7	1.9					3.8			
St8sia1						-1.6			
Stab1						-3.2	-4.5		
Stap1						-1.6			
Stard10		1.8							
Stard13	-1.8					-2.0			
Stard4		-1.8							
Stard5	1.8					2.3			
Stard8	2.0	-3.0				2.8		-2.3	-1.9
Stat1	2.7					3.1	1.6	1.7	
Stat2	1.8					2.6			
Stat3						1.5			
Stau2								-2.1	-1.9
Steap3								-2.9	-2.4
Steap4								-2.8	
Stfa2	-2.6							-2.3	
Stfa2l2	-2.1			1.7				-2.1	
Stil	3.9					2.6	2.9		
Stk24						-1.6			
Stk26						-1.7			
Stk3	1.6					1.9			
Stk36						2.1			
Stmn1	2.9	1.8				2.0	3.0		
Stom		-1.7						-2.2	-1.9
Stoml1						1.6			
Ston2								-3.4	
Stra6	-2.6								
Stra8							15.1	9.5	10.8
Strbp						-1.6			
Strip2	7.5	-6.0				13.0			
Stx11	1.5					2.4			
Stx1b						3.5			
Stx3	1.9	-1.5				2.6			
Stxbp1	1.7					2.0			
Stxbp2						1.6			
Stxbp6						-5.0			
Sub1		1.6					2.4	1.5	
Sucnr1	3.6					12.3			
Sulf1	1.7		1.7					1.9	
Sulf2	2.5	-2.7		-2.1		3.6		-1.9	-2.0
Sult1a1	-5.4	2.9	2.7			-5.4		2.4	
Sult2b1	-2.1								
Sun2						-1.9			
Susd2				2.0					1.7

Suv39h1l1	1.5							
Suz12		-1.9						
Sv2b					-2.2			
Svbp					2.0			
Svip						2.1		
Syde1								2.4
Syne1								1.6
Syne2								1.7
Syt11					-1.6		1.6	1.8
Syt15	-3.3				-7.8	-3.4		
Syt3							-2.7	
Syt5							-4.1	-3.6
Syt6	66.3	315.4						
Sytl1	-1.5				-1.9			
Sytl2_1	1.5		1.6			1.8	1.7	1.8
Sytl3			1.7			2.1	2.1	2.0
Sytl4							-5.9	
T				2.5	3.7	3.3	2.9	5.4
Tac4		-3.5		-2.4			-3.9	-3.4
Tacc2					-1.8			
Taf1						-1.8		
Tagap					1.8			
Tal1		-2.0				-2.1	-2.3	-2.3
Taldo1					2.4		-1.6	-1.6
Tanc1							2.2	2.9
Tap1	2.5				3.5	1.9	1.6	
Tap2					1.9			
Tapbp	1.6				2.1			
Tapbpl	1.7				2.0			
Tarm1							-4.0	
Tas1r2	2.2				3.1			
Tas2r140					-2.6			
Tax1bp1							-1.7	-1.8
Tax1bp3							-1.7	
Tbc1d2		-1.9					-1.9	-1.6
Tbc1d30					-1.8			
Tbc1d4					-2.4			
Tbc1d8		-2.4					-1.8	
Tbc1d9		-3.1	-1.8	-1.5	-1.6	-2.7	-2.7	-1.8
Tbcb					1.5			
Tbkbp1					1.9			
Tbpl1					1.7			
Tbx21	1.8	2.4	2.8	1.9	1.7	2.8	3.3	2.9
Tbx4							-9.5	
Tbxa2r	-1.6				-1.9			
Tbxas1	1.7	-2.7		-2.5	1.7		-3.1	-3.1
Tcam1					-16.1	-5.8	-5.6	-6.5
Tcf12			-1.7					
Tcf15								-7.0
Tcf19	1.6					2.0		
Tcf7					-2.1			
Tcf7l1					-2.7			

Tcf7l2	1.5	-1.8			1.9	-2.0		
Tcirg1	1.7				2.5			
Tcn2		-1.9			1.5		-2.8	-2.0
Tcp11l1	3.0				5.5			
Tcp11l2					-1.6			
Tcte1		7.0		9.1			6.4	8.7
Tdrkh					-1.8			
Tead3		4.3		4.8		4.1		3.2
Tecpr1					-1.7			
Tek		-3.0		-2.5	-8.4	-3.2	-4.2	-3.4
Tenm3	10.0				16.0			
Tesc							-2.2	-1.9
Tet2								1.5
Tex12_1							-2.0	
Tex9		1.7						
Tf		-2.6	-2.1	-2.1			-4.4	-3.1
Tfap2c	4.5				7.7			
Tfap4	-1.5				-1.8			
Tfdp1	1.6							
Tfdp2		2.1				3.4		
Tfe3		-2.1	-1.8				-1.8	
Tfec	6.4	-3.0			8.0			
Tfpt					1.5			
Tgfa	-4.7	-3.6	-2.1		-7.0	-3.1	-5.6	-1.9
Tgfb2				-3.9	-14.8	-5.1	-3.9	-4.0
Tgfb3		-3.9				-4.9	-12.7	-4.8
Tgfbi		-4.7	-2.2	-1.8			-3.5	-2.5
Tgfbr1		-1.6						
Tgfbr3				-2.6	-3.6		-2.7	-2.6
Tgm1					3.9		-3.7	-3.1
Tgm2	2.7	-2.0			3.1			
Thbd		-3.9	-2.8	-3.7	-2.0	-3.9	-7.8	-5.5
Thbs1		-6.0	-2.6				-5.1	-2.5
Thbs3					-4.6	-3.2		
Themis					-2.3			2.2
Themis2		-1.8			1.7		-1.8	-1.5
Thoc2								1.7
Thra					-1.8			
Thrb					-2.0	-2.1		
Thsd7b						3.3		2.4
Thy1		2.2						-2.1
Tiam1		-2.2	-1.7					
Tiam2					-2.5			
Ticam2	2.1	-2.2			2.1			-1.6
Ticrr	4.5					2.5		
Tie1							-1.6	-1.7
Tifa	2.5	-1.8			4.0			-1.9
Tifab	2.1	-2.8			3.0			-1.7
Tigit	1.8					1.9		
Timd2						3.3		
Timd4					-13.7		-5.3	-5.7
Timeless	1.9							

Timp2		-2.1		-2.0		-2.3	-2.1	-3.9	-2.7
Timp3		-10.5					-4.1	-3.4	-4.2
Tinagl1							2.7		
Tjp1	2.6								
Tk1	3.2					2.4	2.8		
Tkt		-1.6						-1.7	-1.6
Tlcd1		3.7		4.1			3.2		3.0
Tlr10	2.0	-2.2				3.1			
Tlr11		-3.0				-3.0			-2.8
Tlr12		-3.0	-2.3			-2.3			-3.4
Tlr13		-2.7	-1.6					-2.7	-1.7
Tlr2	1.5	-2.0				2.2		-1.7	-1.6
Tlr3		-2.0							-2.0
Tlr4	3.1	-2.3				3.4			
Tlr5	3.1		-2.1			2.7		-2.2	
Tlr6						1.7			
Tlr7	1.7	-3.6	-1.8	-1.7				-2.3	-2.0
Tlr8		-3.8	-2.4					-3.0	
Tm4sf19						4.8			
Tm6sf1		-3.3		-1.7				-2.7	-2.4
Tm9sf3		-1.8							
Tmbim1	-2.4					-2.0			
Tmbim4						1.7			
Tmcc2		1.9		-2.5			2.5		-2.2
Tmcc3		-2.2				-2.6	-3.4	-2.4	-1.7
Tmed3						1.5			
Tmed7		-2.1							
Tmem108						4.4			
Tmem117								-3.9	-3.2
Tmem123						-1.5			
Tmem136						-2.5			
Tmem140	2.1					4.7			
Tmem150b	1.7					2.6			
Tmem150c	5.1					4.9			
Tmem151b								2.1	2.3
Tmem156		1.6	1.6						
Tmem163	-1.8					-2.0			
Tmem176a	1.7	-2.0		-2.0		1.7		-2.6	-2.6
Tmem176b		-2.6		-2.3				-3.5	-3.0
Tmem181							1.7		1.6
Tmem184b								-1.6	
Tmem192						1.7			
Tmem2						2.0			
Tmem204	-1.7					-2.2			
Tmem206	-1.8	1.5				-1.6			
Tmem246									-2.6
Tmem256						1.7			
Tmem268						2.3			
Tmem30a		-1.9							
Tmem33						1.7			
Tmem37		-2.4						-2.7	-3.1
Tmem38a	-2.0								

Tmem50b	2.0	-1.9				2.9			-1.6
Tmem51								-2.1	-2.1
Tmem57						-1.6			
Tmem65		-2.7							
Tmem79				4.2			3.6		2.9
Tmem80	-1.5								
Tmem86a	2.2					2.9			-1.9
Tmem86b		2.0					2.5		
Tmem9							1.6		
Tmem97						1.7	2.4		
Tmie							3.5		
Tmod1		3.6				3.2	4.6		
Tmpo	1.7					1.8			
Tmtc1	9.8					5.8			
Tmtc2		-9.0	-3.7					-5.3	
Tmtc3		-2.6							
Tmx1		-1.6							
Tmx3		-2.1							
Tnfaip2	4.4	-2.0				5.7			
Tnfaip3		-2.0	-2.0				-2.1		
Tnfaip8l3	6.1	-3.6				8.0			-2.1
Tnfrsf11a	4.2					4.7			
Tnfrsf13b						-2.0			
Tnfrsf14		1.6							
Tnfrsf17							2.6		
Tnfrsf1a		-1.8				2.2		-1.8	-1.6
Tnfrsf1b		-2.5	-1.6					-1.8	
Tnfrsf21		-2.1	-1.5	-1.7				-2.3	-1.8
Tnfrsf25	-1.6	1.6	1.5						2.0
Tnfrsf26	-1.6								
Tnfrsf8		-2.3							
Tnfsf11						-2.5			
Tnfsf12									-1.8
Tnfsf13		-2.8		-1.7				-2.3	-2.0
Tnfsf13b						1.9			
Tnfsf14	1.7					4.0			
Tnfsf8						-2.0			
Tnfsf9			2.1					2.4	4.4
Tnik									1.7
Tnip2						1.5			
Tnk1						-1.8			
Tnks1bp1						-1.5			
Tnn						-2.6			
Tnnc1						2.9			
Tnni1	2.1					2.2			-3.4
Tnni2									-5.7
Tnni3k			2.4	2.8				2.8	3.0
Tnnt1	3.2								
Tnp2						45.8			
Tnrc6a			1.5						
Tnrc6c									1.5
Tns1						2.9			

Tns3		-2.0						-1.7	
Tnxb						-27.3			
Tob1			-2.0				-2.0	-2.3	
Tob2		-1.7							
Tonsl	2.9					2.3	2.2		
Top2a	4.8					2.9	3.0		
Tor1aip2_1						1.5			
Tor1aip2_2						1.5			
Tor3a						1.7			
Tox						-1.6			
Tp53bp2						-1.7			
Tp53i11						-1.6			
Tp53inp2		-1.8						-1.7	-1.7
Tp73		3.9					6.2	4.3	4.9
Tpbg							-3.9		
Tpd52		-1.8				1.8		-2.2	-2.0
Tpd52l1								-3.3	-3.1
Tph1						-7.7			
Tpi1	1.9					3.2	1.7		
Tppp							-3.7		
Tppp3				-3.6		-5.1	-2.8	-6.6	-4.3
Tprkb									44.9
Tpsab1						-18.7			
Tpsb2						-17.6			
Tpst2							1.8	1.5	1.7
Tpx2	4.2					3.0	3.9		
Tra2a			1.5						
Trafd1						2.3			
Traip	4.2					2.9	3.5		
Trappc1							1.6		
Trappc2l						1.5			
Trat1	-1.5					-2.1			
Trbv1						-1.8			1.5
Trem1		-2.8	-2.0	-1.8		2.1		-3.2	-2.6
Trem2						-4.5		-3.4	
Trem3		-2.0				2.6		-1.8	-1.5
Treml1								-4.3	-4.0
Trex1	2.0					3.0			-1.6
Trib1		-4.8	-3.7	-2.3				-4.6	-2.7
Tril		7.1							
Trim10						2.8	3.2		
Trim13		-2.3						-2.0	
Trim16		-6.3	-2.7			-3.2		-3.6	-3.9
Trim2									-2.7
Trim21	1.7					2.3			
Trim36		-5.3						-3.9	
Trim46	-1.7								
Trim47				-3.3				-3.1	-3.6
Trim55		-3.7	-2.5					-3.9	-2.5
Trim59								1.5	
Trim72						2.0			
Trim9						4.7			

Trio									1.6
Triobp						1.8			
Trip13	2.7					1.8	2.0		
Troap	4.7					3.4	3.4		
Trpc6		-3.4						-5.4	-2.9
Trpm1								-5.3	
Trpm2	2.9					3.5			
Trpm6	-1.8					-2.3			
Trps1			-2.2						
Trpt1		2.9		2.3					
Trrap									1.5
Try5		4.1				-2.9	10.6		
Tsc1									1.5
Tsc22d1		-1.9						-2.9	-2.3
Tshr									2.4
Tshz1									1.7
Tspan12		-2.6					-3.0	-4.9	-3.6
Tspan18	-3.6					-8.2	-3.8	-3.2	
Tspan2		-1.7				1.7		-1.5	-1.5
Tspan31		-1.9				1.7		-1.8	-1.7
Tspan32						-1.7			
Tspan33		2.2				1.9	2.5		
Tspan4									-3.1
Tspan5						1.7			-1.7
Tspan8								-2.2	-2.0
Tspan9								-2.1	-2.5
Tspear									1.8
Tspo						3.0		-1.6	-1.7
Tspoap1						-2.2			
Tspyl4						-2.7			
Tspyl5						-3.5			
Tssc4		1.6							
Tstd3						2.0			
Ttc25						-3.0			
Ttc3							-1.6		
Ttk	3.7					2.5	2.7		
Ttyh2								-3.4	
Tuba1a						1.6			
Tuba1b	1.9					1.7			
Tuba1c	2.0					2.8			-1.7
Tuba8	3.3					9.8			
Tubb1		-3.8	-2.3				-2.3	-5.3	-2.9
Tubb2a		-2.3	-1.8				-1.9	-3.9	-2.9
Tubb2b			-2.0					-2.8	-2.7
Tubb4b	1.7					1.6	1.7		
Tubb5	1.8						1.9		
Tubb6				-4.0			-3.5	-5.4	-4.6
Tusc3	2.0					2.1			
Txk								1.6	
Txn1	2.9					4.9	1.7		
Txn2						1.5			
Txndc16						-1.7			

Tyms	3.1					2.3	3.4		
Tyro3								-3.2	-2.3
Tyrobp						1.9			-1.6
Uaca								-3.2	
Uap1l1						1.5			
Uba7	1.6					2.9			
Ubac1		1.6					1.7		
Ubash3a						-1.7			
Ubc								-1.6	
Ubd	7.2					17.8	5.7	2.7	
Ube2c	4.6	2.1				3.9	4.8		
Ube2d3		-1.6							
Ube2e2									-2.0
Ube2l3						1.6			
Ube2l6	2.1					4.4			-1.9
Ube2o							1.6		
Ube2s							1.6		
Ube2t	4.5					2.6	3.1		
Ubl5							1.7		
Ubp1							-1.7		
Ubqln2							-1.6		
Ubr1									1.7
Ubr4									1.5
Ubxn11						2.5			
Ucp2							2.1		
Ucp3		-2.5							
Ugt1a5	1.9	-3.0		-2.1		2.5		-2.2	-2.4
Uhrf1	4.9					2.9	3.4		
Ulk2							-1.7		
Unc13b						-2.1			
Unc93b1	1.5	-1.7				2.0		-1.8	-1.9
Upp1	2.8					8.2			-1.7
Uqcrb_2						1.6	1.8		
Uqcrq						1.5			
Usf1						1.5			
Usmg5							1.7		
Usp15						1.5			
Usp18	2.0	-2.1				4.0			-1.9
Usp28						-1.5			
Usp33							-1.7		
Usp44						-2.1			1.5
Utrn									1.5
Vangl1	2.5								
Vars	1.5								
Vash1		-4.2	-2.7					-3.5	
Vat1l									2.5
Vcan	1.7	-9.4	-2.3	-2.3		2.3		-3.0	-2.5
Vcl						1.7			
Vdac1	1.6					1.7			
Vdac2						1.5			
Vegfa								-2.8	
Vegfc						-2.6			-3.2

Vgf							-6.5	-4.5
Vil1				-6.8	-31.8	-6.1	-7.3	-7.9
Vim	1.8	-1.7			2.1		-1.5	-1.6
Vldlr		-3.5	-3.8	-2.2		-2.8	-3.7	-2.7
Vnn3					-3.3		-3.7	
Vpreb2					23.0			
Vps13a								2.0
Vps13c	1.6		-1.6					
Vps29					1.5			
Vps35		-1.5						
Vps37b					-1.5			
Vps37d	-1.9	2.0						
Vps41		-1.6						
Vps54					1.6			
Vps9d1					1.6			
Vsig10					-3.8			
Vsig10l	1.7				1.7			
Vsig2		-5.6					-5.3	
Vwa3b	3.7				10.9			
Vwa5a	2.2				3.0			
Vwa5b2								1.6
Vwa8			-2.1					
Wapl		-1.6						
Wars	3.2				4.9	1.8		
Wasf1					6.0			
Wasf2			-1.8					
Wbp11		2.2						
Wdfy2						-1.7		
Wdfy3							-2.1	
Wdhd1	1.9							
Wdr13						-2.1		
Wdr6					-1.6			
Wfdc15b							-2.9	
Wfs1	1.7				2.2			
Whamm					1.6			
Whrn							-1.8	
Wipf3					-1.8			1.6
Wipi1					1.8		-2.0	-2.0
Wls					-2.0			
Wnt2					-42.9			
Wnt4					-4.9	-7.3	-7.4	-13.7
Wnt6	2.3				4.5			
Wrn								1.6
Wscd2	4.5				11.1			
Wtip		-4.0			2.5			-3.2
Wwc2				-2.9		-3.7	-3.0	-4.0
Wwtr1	3.0							-4.0
XAF1	1.6	-2.0			2.2			
Xbp1	1.7	-1.9	-1.6		2.5		-1.6	
Xcl1	2.1				2.1		-1.8	-1.7
Xcr1					-5.5			-3.2
Xdh		-2.6			2.0		-2.3	-1.8

Xirp1			19.8				23.9	22.4	57.2
Xkr5	2.6								
Xkrx	-1.9					-2.1			
Xpnpep1	1.6					1.8			
Xpnpep2						2.2			
Xrcc2	2.1								
Xrcc3	3.3								
Xylb						2.1			
Ybx3		2.2		-1.7		2.2	2.6	1.7	
Yme1l1									1.8
Ypel2		-2.8	-2.0					-2.2	
Ypel3	-1.6								
Ypel5									-1.5
Ythdc2									1.7
Ywhag						1.9			
Zadh2						-1.7			
Zbed3						-1.8			
Zbp1	1.6					2.7		1.5	
Zbtb16						-4.4	-4.1	-3.4	
Zbtb32						2.5	3.5	1.7	1.7
Zbtb4									1.6
Zbtb42		-2.1							
Zbtb44							-1.7		
Zbtb46		-3.4	-2.1			-2.1		-2.5	
Zbtb7b		-1.7							
Zc2hc1a		-2.8				2.1			-2.0
Zc3h11a							-1.6		
Zc3h12a								-1.7	
Zc3h12c								-2.7	
Zc3h12d	-1.7								
Zc3h6						-1.9			
Zcchc24	1.5	-2.5		-1.5		1.7	-2.0	-2.0	-1.7
Zcchc6		-1.9							
Zdhhc1							-2.7	-3.7	-3.1
Zdhhc13	1.5					1.7			
Zdhhc15	2.3								
Zdhhc2						3.4			
Zdhhc21	2.2								
Zfand2a						1.7			
Zfand5						1.7			
Zfat						-1.7			
Zfc3h1							-1.9		
Zfp106			-1.6						
Zfp111		-4.3					-8.4	-3.7	
Zfp14									3.8
Zfp238							-1.8		
Zfp296	-1.7								
Zfp3						-1.9			
Zfp318						-1.7			
Zfp36								-2.6	-1.7
Zfp362						-1.9			
Zfp367	2.1								

Zfp382								1.9
Zfp385a	1.7	-1.6			2.3			-1.6
Zfp385c					-2.0			
Zfp395					-2.2			
Zfp398								1.7
Zfp472					-1.7			
Zfp503		-2.9	-3.0			-3.4	-2.8	-2.9
Zfp507								1.6
Zfp512b					-1.7			
Zfp532							-3.0	
Zfp575								2.0
Zfp579		1.9	1.6					
Zfp605					-2.3			
Zfp62					-1.7			
Zfp622	195.1							
Zfp703		-2.1					-2.4	-1.8
Zfp710		-2.4	-1.9				-1.9	
Zfp819								2.2
Zfp821		1.7						
Zfp869					-1.6	-1.7		
Zfp939					-9.1			
Zfp956					-1.8			
Zfp958		1.8	1.8					
Zfx						-1.6		
Zfyve16	1.7							
Zfyve19		1.7						
Zfyve9		-4.1	-2.2			-2.6	-3.1	-1.7
Zhx2					-2.2			
Zic3								2.4
Zmiz2	477.0				335.4		215.8	
Znf768								-1.8
Znfx1					2.1			
Znhit1					2.2	1.7		
Zrsr1					-2.1			
Zscan26						-1.8		
Zwilch	3.6							
Zyx					1.7			
**Genes numbers**	1332	1213	446	395	2297	1140	1372	1595

RNAseq gene expression. Day 3
Fold change: < 1.5 or >1.5
FDR p-value: < 0.05

	LVS / PBS Day 3	ΔclpB / PBS Day 3
RGD1305184	1310.70	2839.37
Slc1a3	295.71	1180.88
LOC108348096	364.18	335.44
Zmiz2	477.03	335.43
Gzmc	45.61	326.84
Cacna1b	32.05	286.08
Car4	5.95	138.14
Gbp1	60.28	123.01
Batf2	53.27	122.62
Acod1	47.94	118.48
Serping1	21.18	70.30
Cxcl9	21.49	58.62
Mmp14	18.52	49.18
Cxcl11	22.73	49.09
Cxcl10	22.90	48.25
Asb9	17.87	45.78
Sectm1a	22.82	45.19
C1s	25.88	40.26
Col22a1	18.81	36.43
Hk3	17.47	35.56
Ifitm6	3.07	32.24
Card14	10.92	32.11
Il27	15.24	25.21
C4b	13.91	21.01
C4a_1	3.08	20.22
Apol9a	9.77	20.13
Ifitm3	2.50	19.76
Ehf	23.47	19.45
Kctd19	7.82	19.05
Casp12	5.53	18.96
Ubd	7.21	17.85
Arg2	3.25	17.82
Rn60_10_0648.2	2.51	17.50
Gbp2	6.85	17.47
Il18bp	6.46	16.77
Tenm3	10.00	15.96
Gpr84	5.75	15.19
Ass1	8.79	14.91
Rmi2	13.54	14.67
Cx3cl1	6.95	14.49
Kazn	7.82	14.27
Majin	8.76	13.85

	Up-regulated genes Count	
	**LVS**	**ΔclpB**
**Both vaccines**	685	685
**LVS only**	292	
**ΔclpB only**		700
**Total Up-regulated**	977	1385

	Down-regulated genes Count	
	**LVS**	**ΔclpB**
**Both vaccines**	223	223
**LVS only**	131	
**ΔclpB only**		689
**Total down-regulated**	355	912
**Total genes**	**1332**	**2297**

Socs1	4.67	13.74
Pigz_1	3.54	13.42
Strip2	7.48	12.99
C1r	7.38	12.84
Sucnr1	3.63	12.31
Iqcf3	6.81	12.25
Slamf8	7.99	11.90
Wscd2	4.48	11.08
Adgrl3	8.70	11.04
Pdgfrl	7.63	11.03
Vwa3b	3.68	10.93
Hbe1	6.23	10.67
Cyp27a1	5.03	10.66
AABR07006278.1	6.71	10.44
MGC105567	5.66	10.43
Nampt	5.33	10.36
LOC497963	3.01	9.88
NOS2	3.01	9.88
Tuba8	3.28	9.84
Lilrb3	10.63	9.70
Hook1	3.98	9.66
Cryba4	2.65	9.44
AA926063	7.49	9.31
Igf2bp2	4.80	9.25
Sfmbt2	5.10	9.09
AABR07058464.1	4.44	9.05
Lefty1	8.43	8.97
Gadd45g	3.33	8.88
Prss35	5.12	8.68
Gzmbl1	3.38	8.35
Art3	6.10	8.29
Snx24	4.75	8.29
Ptges	5.53	8.18
Upp1	2.75	8.15
AABR07043075.1	3.68	8.10
Tnfaip8l3	6.06	8.02
Apol11a	6.19	8.01
Tfec	6.45	7.95
Siglec1	4.93	7.90
Slc41a2	4.55	7.90
Kcng3	4.76	7.88
Ralgds	2.68	7.83
C4a_2	4.71	7.81
C2	5.84	7.78
Ggt1	2.24	7.78
Tfap2c	4.47	7.67
Mt2A	4.10	7.66
Ms4a7	5.65	7.63

Lacc1	6.99	7.46
Ifi47	4.13	7.44
Irgm	4.62	7.36
Eml1	5.36	7.12
Olr1584	3.21	6.98
Erbb3	4.18	6.94
Slc7a2	3.82	6.90
Ifng	3.26	6.88
Il15ra	3.66	6.80
Abca4	3.15	6.65
AABR07006263.1	4.95	6.63
Nrg1	2.63	6.61
Gtsf1	3.32	6.52
Layn	5.03	6.36
Gbp5	4.15	6.35
Cd300lf	3.14	6.33
Ntn1	3.67	6.30
Htr7	4.12	6.14
Igtp	4.04	6.12
Serpinb1a	1.61	6.11
Gng3	3.83	6.07
Dsg1	5.08	6.04
Smarcd3	2.51	6.01
Plau	3.09	5.88
Isg15	1.69	5.76
Tmtc1	9.75	5.76
Tnfaip2	4.43	5.75
Scimp	2.51	5.64
Nrp2	3.87	5.63
Olfm1	3.37	5.62
Smad6	4.08	5.60
Ssc5d	3.04	5.53
F10	4.23	5.53
Ak7	2.37	5.50
AC142360.1	5.12	5.46
Tcp11l1	2.96	5.45
Col15a1	2.35	5.44
RGD1311744	4.84	5.43
Rn60_20_0141.4	2.38	5.33
Scarf1	3.78	5.21
Ccr2	3.13	5.20
Mfsd7	3.34	5.14
LOC100360218	3.60	5.11
Mroh2a	2.53	5.08
Irf1	3.28	5.08
Rag1	11.10	5.00
Tmem150c	5.11	4.92
LOC100911104	3.04	4.91

Txn1	2.94	4.90
Gbp4	3.39	4.89
Wars	3.19	4.87
AABR07016572.1	2.13	4.82
Rhox5	2.88	4.74
Casp4	3.06	4.73
Tmem140	2.06	4.71
Fbn1	3.72	4.71
Tnfrsf11a	4.24	4.69
Hsd3b7	2.53	4.68
Hist1h3a	4.75	4.66
Arrdc4	4.74	4.63
Hs6st1	3.04	4.62
Ms4a6a	2.63	4.59
Npy1r	5.40	4.51
Wnt6	2.28	4.51
Ngef	4.20	4.51
Ptgs1	2.90	4.49
RGD1563091	2.80	4.47
Csrp2	4.87	4.46
Ube2l6	2.11	4.45
LOC685067	3.96	4.43
Pkig	3.21	4.41
Slc25a27	3.55	4.41
Atp10a	3.14	4.40
AABR07002674.1	2.07	4.38
Mlkl	2.23	4.36
Acpt	2.39	4.35
Shisa4	3.90	4.32
Rab13	2.79	4.29
Ccr5	3.11	4.25
Slc8a1	3.39	4.20
Sod2	2.13	4.16
Hist2h3c2_1	4.81	4.13
Nod1	2.79	4.10
RGD1308065	4.55	4.10
Fcnb	2.82	4.08
Rn60_20_0069.2	2.72	4.07
Hist1h2bk	4.27	4.05
Neil3	4.24	4.03
Tnfsf14	1.67	4.01
Rab32	2.49	4.01
Tifa	2.52	3.97
Hk2	2.54	3.96
Usp18	2.00	3.95
Gab1	2.92	3.91
Cdk1	5.17	3.90
Lap3	2.95	3.89

Ube2c	4.61	3.89
ST7	1.93	3.80
Fabp5	2.64	3.80
Adcy4	2.01	3.76
Orc1	6.53	3.76
Rtp4	1.93	3.75
Nfam1	2.23	3.74
Gyg1	2.77	3.74
Ska1	5.30	3.73
Ns5atp9	5.43	3.72
Ptger3	3.53	3.72
Pstpip2	2.24	3.70
Ly49i2	3.04	3.69
Mki67	5.02	3.69
Ccne1	4.26	3.68
Ripk3	2.15	3.68
Cdkn3	4.55	3.68
Eme1	5.56	3.67
Psmb9	2.23	3.67
Smad9	2.26	3.63
Ifitm10	2.53	3.62
Nrg2	3.75	3.61
MGC105649	2.60	3.59
C3	2.48	3.57
Ccne2	3.76	3.56
Hebp1	2.10	3.56
Sulf2	2.51	3.55
Trpm2	2.94	3.54
Exo1	4.70	3.53
E2f8	6.14	3.52
LOC100912611	4.16	3.52
Mnda	2.39	3.52
Mcrip2	1.79	3.50
Lpin2	2.16	3.48
Prr5l	2.40	3.46
Tap1	2.45	3.46
Pnp	2.47	3.45
Tlr4	3.08	3.44
Troap	4.68	3.40
LOC24906	2.05	3.40
Rab39a	2.44	3.39
Cenpt	5.03	3.39
Mybl2	4.89	3.36
Shcbp1	5.20	3.35
Mt1	2.46	3.35
Npr1	1.90	3.34
Gpnmb	2.10	3.34
Sectm1b	2.46	3.33

Limk2	2.17	3.32
Mis18a	4.45	3.32
Psme2	2.15	3.32
Pla2g4a	2.54	3.31
Cdkn2a	3.91	3.30
LOC100910554	3.23	3.30
Cdc6	4.62	3.29
Bcat1	1.96	3.29
Ccna2	5.49	3.26
Snx10	2.93	3.25
Cp	3.16	3.24
Raet1d	4.03	3.23
Fcgr1a	1.72	3.23
Tpi1	1.88	3.23
Sapcd2	4.70	3.21
Hlx	1.68	3.21
LOC100910979_2	2.33	3.21
Marcksl1	2.91	3.19
LOC108348048	2.70	3.19
Dtx3l	2.42	3.19
Nuf2	4.77	3.18
Parpbp	4.92	3.18
Fam111a	4.33	3.17
Kif2c	5.26	3.17
C1qtnf1	2.11	3.16
Mcm10	3.09	3.16
P2ry13	2.35	3.15
Tlr10	2.00	3.15
Cadps2	3.07	3.14
Stat1	2.68	3.13
Csf2rb	1.93	3.13
Lyc2	2.34	3.12
Ctsz	1.83	3.11
Aif1	2.14	3.11
Prr11	6.36	3.10
Tas1r2	2.19	3.08
Spic	2.29	3.08
Tgm2	2.73	3.07
Cenpw	4.30	3.05
LOC691141	2.09	3.05
Cdca3	4.31	3.05
Cdh5	3.36	3.04
Il18rap	2.11	3.04
Kif20a	4.85	3.04
Ppt2	2.00	3.04
Depdc1	5.26	3.04
Ppard	1.54	3.03
Bub1	5.23	3.02

Cks2	3.89	3.02
Prdx5	1.77	3.01
Dtnb	2.20	3.01
Mapk10	3.42	3.00
Vwa5a	2.24	3.00
Fcrl6	4.03	3.00
Trex1	2.03	3.00
Sgo2	4.51	3.00
Gpr88	2.36	2.99
Dclk1	3.08	2.98
Tpx2	4.20	2.98
Slc38a6	2.17	2.97
Il12rb2	4.56	2.97
Kif4a	4.50	2.95
Ncapg	5.98	2.95
Tifab	2.14	2.95
Grik5	2.02	2.95
LOC108351137	1.76	2.94
Cyp39a1	3.30	2.94
Ncaph	4.81	2.93
Uhrf1	4.92	2.93
Pttg1	2.71	2.92
Csf1	1.90	2.91
Bst2	2.07	2.91
Rnf213	2.35	2.91
Serf1	2.55	2.90
Clec12a	1.66	2.90
Asf1b	4.37	2.90
P2ry14	2.23	2.90
Enpp4	2.39	2.89
Nostrin	3.11	2.89
Mocos	1.77	2.89
Traip	4.24	2.89
Tmem86a	2.17	2.88
Fez2	1.85	2.88
Rassf4	2.39	2.88
Pbk	5.12	2.88
Top2a	4.77	2.88
Cd274	2.14	2.88
Uba7	1.63	2.87
Slc38a7	1.67	2.86
Lipg	1.89	2.85
Asrgl1	1.69	2.85
Tmem50b	1.99	2.85
Ifi30	1.78	2.85
LOC102549173	2.53	2.84
Plk1	4.21	2.84
Spc24	3.69	2.84

Pter	1.91	2.84
Stard8	2.02	2.83
Iqgap3	2.48	2.83
Slc29a3	1.70	2.82
Cenpe	4.87	2.82
Pla2g2d	2.22	2.81
Rab20	1.86	2.80
Lrp1	1.89	2.78
Nusap1	4.63	2.78
Kif22	3.88	2.78
Espl1	4.35	2.77
AABR07069282.1	4.27	2.77
Birc5	3.72	2.77
Tuba1c	1.99	2.75
Phactr2	2.53	2.75
LOC100910270	2.00	2.75
Qpct	2.51	2.75
Parp3	1.83	2.74
Lyz2	1.90	2.74
Fam72a	2.58	2.73
LOC100910252	5.04	2.73
Itga9	3.53	2.73
Cmklr1	2.54	2.73
Cd302	1.92	2.73
LOC102555924	2.03	2.73
Ska3	3.76	2.72
Gzma	3.07	2.71
Glipr2	1.55	2.70
Ppargc1b	2.04	2.70
Rhbg	1.99	2.70
Zbp1	1.63	2.69
Icam1	2.52	2.69
Melk	4.65	2.69
Cd14	2.79	2.69
Tlr5	3.13	2.68
Spag5_2	4.23	2.68
Psmb8	1.65	2.67
Gzmb	2.83	2.67
Rnf114	1.80	2.66
Slc25a28	1.58	2.66
Mpa2l	2.56	2.66
Cenph	4.31	2.66
Rad51	4.06	2.65
Stat2	1.76	2.65
Rpp25	2.52	2.65
P2ry12	2.39	2.64
Apol3	1.80	2.64
Tmem150b	1.74	2.63

Rnasel	1.52	2.63
Glrx	1.73	2.63
RT1-N3	1.96	2.63
LOC100364062	2.45	2.63
Ube2t	4.46	2.62
Ccnf	3.84	2.62
Cenpf	4.39	2.62
Sgo1	3.96	2.61
RGD1311756	2.08	2.61
Fignl1	4.00	2.61
Slc35f5	1.94	2.60
Bcl3	1.53	2.59
Cep55	4.31	2.59
Dsg3	1.69	2.59
Ccnb1	3.95	2.59
Stx3	1.88	2.59
Dhx58	1.69	2.59
Creld2	1.62	2.58
AABR07016856.1	2.39	2.57
Gins1	3.21	2.57
Fam83d	3.46	2.57
Stil	3.94	2.57
Spc25	3.72	2.56
Parp9	1.68	2.55
Ak4	2.66	2.54
Aunip	4.26	2.54
Dpagt1	1.56	2.54
Nlrc4	1.94	2.53
LOC102549061	2.69	2.53
Kif23	4.05	2.52
Nr1h3	2.24	2.52
Cdc20	4.08	2.52
ENSRNOG000000562	3.67	2.52
Gas7	1.78	2.52
Ugt1a5	1.94	2.51
Gpr157	2.17	2.51
Gdf10	3.40	2.51
Knstrn	3.66	2.51
RGD1310335	4.67	2.51
NEWGENE_1306714	2.86	2.50
Racgap1	4.38	2.50
Rnf128	2.40	2.49
Cenpk	3.51	2.49
Mastl	3.89	2.49
Psen2	1.96	2.47
AABR07006310.1	2.28	2.47
Xbp1	1.73	2.46
Pak1	1.57	2.46

Tcirg1	1.66	2.46
Cdca8	3.53	2.46
Kif20b	4.70	2.45
Ttk	3.73	2.45
Cdc42ep2	1.92	2.45
Cndp2	1.82	2.45
Gapdh	1.71	2.44
Gpr141	2.13	2.43
Fuca2	1.83	2.43
Mefv	1.79	2.42
Myof	2.19	2.42
Knl1	4.28	2.41
Paqr8	2.31	2.41
Kif18b	3.95	2.40
Ggct	1.58	2.40
Slc45a4	1.89	2.40
Entpd1	1.72	2.40
Slc39a8	1.99	2.39
Islr	2.22	2.39
Cdca2	4.22	2.38
Plekho2	2.58	2.38
Aurkb	3.36	2.38
Stx11	1.52	2.38
Fmnl2	2.37	2.38
Ect2	4.56	2.38
Gmnn	2.57	2.38
Bak1	1.65	2.37
Gtse1	3.41	2.37
Prc1	3.28	2.36
P4ha1	1.68	2.36
Smad1	1.98	2.36
Tk1	3.16	2.35
RT1-T24-3	1.63	2.35
Tonsl	2.94	2.34
Lgals3	1.51	2.34
Oma1	2.05	2.34
Psmf1	1.75	2.34
Eif4e3	1.77	2.33
Stard5	1.76	2.32
Dynlt1	1.51	2.32
Isoc2b	1.71	2.32
RGD1309350	2.26	2.32
Tyms	3.15	2.32
Zfp385a	1.66	2.31
Ripk2	1.65	2.31
Ptpn5	3.59	2.30
Lim2	2.29	2.30
Plcb3	1.63	2.29

Hopx	1.84	2.28
Ankle1	3.89	2.28
Trim21	1.74	2.27
Fbxl15	1.67	2.26
Pkdcc	2.01	2.25
Fancd2	3.54	2.25
Cmpk2	1.52	2.25
Vcan	1.74	2.25
Slc11a1	1.63	2.25
Anpep	1.70	2.25
Adap2	1.96	2.24
Pim1	1.62	2.24
Plk5	3.90	2.24
Fkbp2	1.54	2.24
Mboat1	1.65	2.23
Tnni1	2.08	2.23
Gda	2.06	2.23
Cds1	1.94	2.23
Rnaset2	1.69	2.23
LOC108348072	1.72	2.22
Pla2g16	1.82	2.20
Acp2	1.90	2.20
Ndc80	3.29	2.20
Tlr2	1.51	2.20
Ckb	1.59	2.19
Esco2	3.88	2.17
Wfs1	1.71	2.17
Ctnnbip1	1.82	2.17
Fscn3	2.10	2.17
Ly49si2	1.89	2.17
Mxd1	1.54	2.16
Kif11	4.05	2.16
Scarb1	1.88	2.16
Ly49si3	1.90	2.16
Rnd3	1.85	2.16
XAF1	1.57	2.15
Sgms2	1.69	2.15
LOC100911519	2.03	2.15
Ciita	1.56	2.15
Hmox1	1.68	2.15
Cst7	2.21	2.15
Aurka	1.81	2.14
Ak2	1.82	2.14
Renbp	1.75	2.14
Nod2	1.76	2.14
Lactb	1.66	2.13
LOC303566	2.62	2.13
RT1-Bb	1.70	2.12

Cstb	1.52	2.12
Gpsm2	3.64	2.12
Cdc45	3.08	2.12
Parp12	1.57	2.11
Bard1	3.15	2.11
Impa2	1.72	2.11
Vim	1.82	2.11
Lrp8	1.84	2.11
Foxm1	3.34	2.11
Kifc1	3.00	2.10
Spns3	1.88	2.10
Mitf	1.84	2.10
Rad54l	3.06	2.10
Tapbp	1.62	2.10
Lmnb1	2.74	2.10
RT1-Db1	1.57	2.09
Plek	1.59	2.09
Xcl1	2.15	2.09
Tusc3	2.01	2.08
B4galt5	1.66	2.08
Cox5a	1.53	2.08
Parp14	1.63	2.08
Ckap2	3.04	2.07
Gramd3	1.75	2.07
Mvd	1.55	2.07
RT1-S3	1.69	2.07
Cdc42bpb	1.84	2.07
Ctif	1.80	2.06
Cd68	1.81	2.06
Ticam2	2.15	2.05
RT1-Da	1.74	2.05
RGD1309808	1.80	2.05
Nipsnap3b	1.61	2.05
Aldh7a1	1.79	2.05
N4bp1	1.55	2.04
Cenpa	3.11	2.04
RT1-Ba	1.60	2.04
Atp10d	1.87	2.04
Dbi	1.61	2.04
S1pr2	1.65	2.03
Ptprj	1.68	2.03
Stxbp1	1.75	2.02
Slc2a6	1.72	2.02
Mcub	1.66	2.02
LOC497796	1.88	2.01
Tapbpl	1.65	2.00
Stmn1	2.92	1.99
LOC100911562	1.87	1.99

Dctpp1	2.04	1.99
Ssh3	1.64	1.98
Unc93b1	1.53	1.98
Mcm5	2.81	1.98
Pbx3	1.58	1.98
Myo5a	1.85	1.97
Plk4	3.29	1.97
Rps4y2	1.88	1.96
Arhgap19	3.27	1.96
Acvrl1	1.97	1.96
Cks1b	2.74	1.95
Psma5	1.58	1.95
LOC680121	1.67	1.94
Stk3	1.60	1.94
RT1-DMb_1	1.57	1.93
Arhgap11a	2.57	1.93
Irf8	1.75	1.92
Cd180	1.73	1.92
Pole2	2.49	1.92
Fbxo5	2.95	1.92
Naip6	1.89	1.92
Cenpm	1.82	1.90
Lrrc59	1.62	1.90
Orc3	1.70	1.90
Kif15	3.42	1.90
Scamp1	1.53	1.89
Gsap	1.55	1.89
Gas2l1	2.26	1.89
Mapkapk2	1.55	1.88
Cenpn	1.98	1.88
Nlrc5	1.95	1.87
Ap1s2	1.67	1.87
Prdx1	1.50	1.87
Sgpp1	1.60	1.87
Kpna2	2.19	1.86
Tcf7l2	1.52	1.86
Acpp	1.61	1.86
Xpnpep1	1.55	1.84
Lmtk2	1.61	1.84
Aida	1.69	1.84
B2m	1.54	1.84
Trip13	2.68	1.83
Incenp	2.62	1.83
Rnf144b	1.70	1.83
Snrpa	1.54	1.82
Plxna1	1.57	1.82
Rnf19b	1.51	1.82
Ctbp2	1.71	1.82

Nsd2	2.42	1.82
Clip2	1.51	1.81
N4bp2l1	1.58	1.81
Arl5b	1.73	1.80
Idh2	1.50	1.80
Ctss	1.54	1.80
Slc30a1	1.55	1.80
Slc2a9	1.56	1.80
Ahcy	1.77	1.80
Chaf1b	2.24	1.80
LOC100359600	2.37	1.79
Psat1	2.49	1.79
Hip1	1.53	1.78
Tmpo	1.69	1.78
Samhd1	1.97	1.78
LOC103692716	1.64	1.78
Bcl2a1	1.52	1.77
Lgals3bp	1.52	1.76
AABR07033324.1	1.67	1.76
Cyfip1	1.68	1.75
Ncapd2	2.46	1.75
Ermp1	1.53	1.74
Eml2	1.56	1.74
Ska2	2.12	1.74
Rrbp1	1.51	1.73
Zcchc24	1.52	1.73
Casp7	1.60	1.73
Lsm2	1.77	1.73
Asl	1.53	1.72
Rnpep	1.51	1.71
Tmem176a	1.68	1.70
Ccdc102a	1.68	1.70
Vdac1	1.59	1.69
Dut	2.33	1.69
Tuba1b	1.86	1.69
Crat	1.58	1.68
Cdt1	2.20	1.68
AABR07011951.1	1.82	1.68
Vsig10l	1.69	1.68
Epsti1	1.56	1.68
Zdhhc13	1.51	1.68
Magohb	2.16	1.68
Dlat	1.52	1.68
Cybb	1.63	1.68
Tbxas1	1.65	1.66
Tbx21	1.85	1.65
H2afx	2.18	1.65
RGD1309594	1.56	1.64

LOC688655	2.02	1.64
Basp1	2.19	1.64
Klrb1a	1.62	1.63
Rcan1	1.50	1.62
App	1.55	1.62
Dlgap5	2.67	1.61
Sh3bp1	1.53	1.59
Nkg7	2.05	1.58
Snrpd1	1.56	1.58
Ezh2	2.15	1.58
Hif1a	1.51	1.57
Tubb4b	1.71	1.57
Actg1	1.53	1.56
Nfe2l2	1.52	1.56
Etfb	1.60	1.55
Ada	1.53	1.55
LOC102555457	1.52	1.53
Hmgb2	1.78	1.52
Ndufaf7	1.53	1.52
Grcc10		305.87
Hmx3		183.09
Bsnd		150.41
Asb4		117.42
Spink2		112.73
Selenbp1		99.77
Col4a2		70.92
NEWGENE_621438		68.76
LOC102549115		55.28
LOC100909857		47.09
Tnp2		45.77
Anos1		40.33
Ly6h		31.64
Nrg4		24.81
Vpreb2		22.97
AABR07065656.8		21.16
Cd177		17.59
Igll1		13.11
Rn60_20_0051.7		12.44
Htra1		11.46
Prlhr		11.35
Cela2a		11.03
Lvrn		10.18
Adgb		9.70
Pigz_2		8.96
Lcn2		8.93
Ankrd22		8.67
Duoxa2		8.26
AABR07049695.3		7.74

Alpl	7.68
Sct	7.67
LOC100909700	7.11
Brs3	7.10
Mfap2	7.04
Ptch2	6.84
Flvcr2	6.73
RatNP-3b	6.65
Gas1	6.62
Espnl	6.57
AABR07001493.1	6.41
AABR07044414.1	6.37
Il1a	6.13
Ffar2	6.06
Wasf1	6.01
LOC100911101	5.98
Defb24	5.95
Igf2bp3	5.85
AABR07012775.1	5.83
Csf2	5.75
Ptpru	5.74
AABR07064867.2	5.61
Elane	5.55
Aqp12a	5.52
S100a8	5.48
Six1	5.35
F12	5.31
Sema3c	5.30
Erg	5.29
Slco2b1	5.20
Crispld2	5.02
Rag2	4.92
Sntg2	4.88
S100a9	4.87
Ngp	4.81
Tm4sf19	4.79
Glt1d1	4.73
Trim9	4.67
F3	4.47
Gfap	4.44
Cpm	4.43
Ahsp	4.41
Tmem108	4.38
Gsta4	4.32
Pglyrp1	4.30
AABR07002898.1	4.30
AABR07044454.1	4.25
Atp1a4	4.19

Camp	4.17
Cd33	4.14
Pram1	4.08
Mylk2	4.05
Sftpb	4.02
Cpt1c	4.00
Cck	3.99
Spred3	3.96
Orm1	3.91
LOC103694380	3.88
Tgm1	3.86
Olfm4	3.84
Mmp8	3.81
Egfl8	3.79
Rab44	3.78
LOC100911881	3.73
Rgs5	3.66
T	3.65
Slc27a2	3.58
Fetub	3.57
Clmp	3.57
Cpeb1	3.55
Noct	3.52
RGD1304810	3.49
Rufy4	3.46
Stx1b	3.46
Atp6v1g2	3.45
Lgals5	3.44
Atp6ap1l	3.41
LOC103689947	3.37
Zdhhc2	3.37
Retnlg	3.32
Ly6al	3.31
AC109891.1	3.31
Ifit1bl	3.26
Misp	3.25
Id1	3.24
LOC100361087	3.23
Gapdh-ps2	3.22
Snca	3.22
Mmp9	3.22
LOC100911272	3.21
Tmod1	3.20
Cystm1	3.19
Exoc3l4	3.19
Mcemp1	3.17
Fam114a1	3.15
Rims2	3.12

Irf7	3.12
Olr1588	3.11
Gipc2	3.08
Nek10	3.07
Fabp3	3.06
Cysltr1	3.03
Ank1	3.03
Scube1	3.03
Ispd	3.03
Dgat2	3.02
Add2	3.01
Tspo	3.00
Ift57	2.99
Nrtn	2.99
Prg2	2.98
Rn50_7_1163.2	2.97
Rsph1	2.96
LOC103689942	2.96
Tnnc1	2.94
Mybpc3	2.93
AABR07052458.1	2.93
Mroh2b	2.90
Ifit3	2.90
Slfn4	2.89
Kcnk12	2.89
Mip	2.89
Tns1	2.89
LOC100911730	2.89
Oas1a	2.87
Clec4b2	2.86
Ptgs2	2.86
Gdpd1	2.85
Aqp1	2.83
Trim10	2.82
Cpne8	2.81
Arid5a	2.80
Fbxl5	2.79
Ift43	2.78
Csf3r	2.76
Isg20	2.74
Oasl2	2.73
Epb41l1	2.73
Fgf12	2.72
Hba-a2_1	2.71
Phyhd1	2.71
Cd52	2.70
Gch1	2.70
Ifitm1	2.70

Oas1b	2.70
Rsad2	2.70
Rhd	2.70
Ptgr1	2.67
C1rl	2.67
Ly6l	2.67
RGD1565355	2.67
Mfsd2a	2.67
RGD1311084	2.66
Ptges3l	2.66
Grina	2.66
Sbno2	2.65
Alas1	2.65
Lonrf3	2.64
RGD1562758	2.63
Mgam	2.63
Epb42	2.62
Herc6	2.62
Olr1587	2.61
Piwil4	2.61
Spta1	2.61
LOC502907	2.60
Naprt	2.60
Cfb_1	2.58
LOC103694857	2.58
Alas2	2.58
Trem3	2.57
Socs3	2.57
LOC103694855	2.55
Kdelr3	2.55
Slfn14	2.55
Hbb	2.53
Rec114	2.53
Slc4a1	2.53
Hba-a2_2	2.52
Fcar	2.51
Zbtb32	2.51
Ddah1	2.50
Wtip	2.49
Atp13a2	2.49
Actr3b	2.49
Oscar	2.49
Ubxn11	2.48
A3galt2	2.45
Cdkn1a	2.45
Hoga1	2.43
Taldo1	2.43
Oaf	2.42

LOC691931	2.42
Cntnap2	2.42
Cers6	2.42
LOC498154	2.42
Il1rn	2.41
Oas3	2.41
Hemgn	2.41
Degs2	2.41
Casp1	2.41
Anxa3	2.40
Lst1	2.38
Plekhb1	2.38
Hp	2.38
Asns	2.37
Litaf	2.37
Muc20	2.36
Klf1	2.36
Ifit2	2.35
LOC108348047	2.34
Rmdn3	2.34
Tmem268	2.34
Ctrc	2.33
Cyp4f18	2.33
Bnip3	2.32
Mrps6	2.32
Slc6a9	2.32
Ltb4r	2.30
Gsdmc	2.29
Nfatc4	2.29
LOC690000	2.29
Gk	2.28
Sptb	2.28
Serinc2	2.28
Trafd1	2.28
Ckap4	2.28
Prdx2	2.26
Cyba	2.26
S100a11	2.26
Ltbr	2.26
RT1-S2	2.25
Pomp	2.25
Opn3	2.24
Nhp2	2.24
Bpgm	2.24
Xpnpep2	2.23
Ppp1r3b	2.23
Mgst1	2.23
Fech	2.23

Creg1	2.22
AABR07062170.1	2.21
Atp6v1g1	2.20
Rhbdf2	2.20
Ybx3	2.20
Msrb1	2.19
Gpt2	2.19
Aldoc	2.19
Znhit1	2.18
Tnfrsf1a	2.18
Fes	2.17
Agmat	2.17
Capg	2.17
Cebpb	2.17
P2ry6	2.16
Sdf2l1	2.16
Sh3yl1	2.16
Agrn	2.15
Ifi44	2.15
Clec2l	2.15
Mb21d1	2.14
Slc35a5	2.14
Lrg1	2.13
Fcer1g	2.12
Trem1	2.12
Lilrb4	2.12
Xylb	2.12
Zc2hc1a	2.11
LOC100909856	2.11
Megf8	2.11
Cd40	2.11
Ly6e	2.11
Ppial4d	2.10
Znfx1	2.10
Scly	2.09
Manf	2.09
Fbxo39	2.08
Calr	2.08
Padi4	2.08
Ift122	2.08
Batf3	2.08
Birc3	2.08
Pf4	2.08
Atp6v0c	2.07
Stk36	2.07
Ggn	2.06
Csf2ra	2.06
Pld1	2.06

Gng11	2.05
Gpr146	2.05
Ly86	2.05
Psmb10	2.04
Snai1	2.04
P2rx1	2.04
ENSRNOG00000047276	2.04
Svbp	2.03
Nqo1	2.03
Klhdc9	2.02
Adam9	2.02
Ly6g6f	2.01
Rasgrp4	2.01
Slc2a1	2.01
LOC100911483	2.01
Fam214b	2.01
Ly75	2.00
Xdh	2.00
Eif4ebp1	2.00
Gp1bb_2	2.00
Marcks	1.99
Dbnl	1.99
Clec5a	1.99
Gpr137	1.99
Sidt2	1.99
Tstd3	1.98
LOC103689996	1.98
Ccdc92	1.98
Aldh3b1	1.98
Rbm20	1.98
Ftl1	1.97
Slc8b1	1.97
Ebi3	1.97
Il18r1	1.97
Trim72	1.96
Ccdc134	1.96
Ncf4	1.96
Tmem2	1.96
Cox7b	1.96
Dapp1	1.95
Ethe1	1.95
Tspan33	1.95
Tbkbp1	1.95
Aspa	1.95
Nuak2	1.95
Aldoa	1.94
Il15	1.94
Il13ra1	1.94

Ap2s1	1.93
Fam129c	1.93
Hspa5	1.93
Tnfsf13b	1.93
Gng10	1.93
Gys1	1.93
Sema4a	1.93
Gabrr2	1.93
Adgrg3	1.93
RT1-DMb_2	1.92
Lrrc49	1.92
Mfsd12	1.92
Fam213a	1.92
Tyrobp	1.92
Tap2	1.91
Mtus1	1.90
Ptafr	1.90
LOC100912218_2	1.90
Ampd3	1.89
Mettl9	1.89
Cdr2	1.89
Ywhag	1.89
Kynu	1.89
Fas	1.89
Cd74	1.88
Pdia4	1.88
Spi1	1.88
Bst1	1.88
Hm13	1.87
Bid	1.87
Ctsh	1.87
Pygl	1.86
Chid1	1.86
Mitd1	1.86
Sema6b	1.86
Kars	1.86
Ctsc	1.86
Parp10	1.86
RGD1561157	1.85
LOC100911825	1.85
Narf	1.85
Cklf	1.85
Iqsec2	1.85
Galnt3	1.85
Skap2	1.85
Lrrc75b	1.85
Eif2ak2	1.84
Clcn7	1.84

Ssr3	1.84
Amdhd2	1.84
LOC100360087	1.84
Plekha5	1.84
Mpv17l2	1.84
Nfe2	1.84
Pdk3	1.83
Pdia3	1.83
Ndufb7	1.83
Ifngr2	1.83
Gclm	1.81
Dtnbp1	1.81
Sod1	1.81
Aim1	1.81
Gadd45b	1.81
Tpd52	1.80
Slfn13	1.80
RGD1311739	1.80
Acvr1b	1.80
Lrfn4	1.80
Atp6ap2	1.80
Specc1	1.79
Osgin1	1.79
Bcl2l1	1.79
Ppp1r3d	1.79
Flot2	1.79
Triobp	1.79
Nme6	1.79
RT1-T24-4	1.79
Wipi1	1.78
RGD1562652	1.78
Gcsh	1.78
Alpk1	1.78
Cebpd	1.78
Tagap	1.78
Parp8	1.78
Hagh	1.77
MGC95208	1.77
Mt-co1	1.77
Sec11c	1.77
Saa4	1.77
Sec61g	1.77
Klra2	1.77
Dhrs11	1.76
Chi3l1	1.76
Ncf1	1.76
Ncf2	1.76
Cenpj	1.76

Daxx	1.76
Itga5	1.75
Rab1a	1.75
Atp6v1a	1.75
LOC100363502	1.75
St3gal4	1.75
Mafk	1.75
Batf	1.74
Psmb2	1.74
Rps27l	1.74
Slc1a4	1.74
Anxa4	1.74
Eno1	1.74
Atp6v1b2	1.74
LOC100911238	1.74
Pgam1	1.74
Mov10	1.74
Gripap1	1.74
Sppl2a	1.73
Clic1	1.73
Atg3	1.73
Slc15a3	1.73
Glrx5	1.73
Igsf6	1.73
Ogfr	1.73
Picalm	1.73
Lta4h	1.73
RT1-A1	1.73
Tspan31	1.73
Pdia6	1.73
Slc35b1	1.73
Tspan5	1.72
Igsf8	1.72
Mpst	1.72
Zfand2a	1.72
Hyou1	1.72
Ccdc93	1.72
Mcpt8	1.72
Il3ra	1.71
Slirp	1.71
Psme1	1.71
Oasl	1.71
Ifi35	1.71
C5ar1	1.71
Lamp1	1.71
Lyn	1.71
RT1-CE6	1.71
Hnrnpc	1.71

Dstn	1.71
Adam17	1.71
Galns	1.71
Dok3	1.71
Nudt9	1.71
P2rx4	1.71
Zyx	1.71
Tlr6	1.71
Brca2	1.71
Mvb12a	1.70
Pstpip1	1.70
Mvp	1.70
Pgk1	1.70
Hsp90b1	1.70
Ece1	1.70
Mboat7l1	1.70
Nmi	1.69
Zfand5	1.69
Sdhb	1.69
Atp1a3	1.69
Tmem192	1.69
Commd9	1.69
Tmem33	1.69
Tspan2	1.69
Ms4a6bl	1.69
Sec61b	1.69
Slc19a1	1.69
Tmbim4	1.69
Grn	1.69
Tmem97	1.69
Themis2	1.69
Sdcbp	1.69
Tmem256	1.68
Jak2	1.68
Scfd1	1.68
Tbpl1	1.68
Lamtor3	1.68
RT1-CE5	1.67
Sp140	1.67
Parvg	1.67
Jdp2	1.67
Mob3c	1.67
Higd1a	1.67
Slc25a16	1.66
Sp110	1.66
Atox1	1.66
E2f2	1.66
AABR07026311.1	1.66

Vcl	1.66
Ldha	1.66
P4hb	1.66
RT1-CE10	1.66
Pld4	1.66
Tor3a	1.65
Rnaseh2a	1.65
Atp13a1	1.65
Bri3	1.65
Atad1	1.65
Slc2a8	1.65
Hacd2	1.64
Ifitm2	1.64
Nrip1	1.64
Vps9d1	1.64
Fam162a	1.64
Mt-nd5	1.64
St6galnac4	1.63
Lrrk2	1.63
Ppox	1.63
Sort1	1.63
Psma2	1.63
Gnpda1	1.63
Dhdds	1.63
Chmp2a	1.63
Psap	1.63
Dclre1c	1.63
Nr2f6	1.63
Aph1a	1.62
LOC500956	1.62
Psma7	1.62
Naa20	1.62
Tuba1a	1.62
Clpb	1.62
Slc37a1	1.62
Plaur	1.62
Lilrb3a	1.62
Chmp2b	1.61
Ccnd3	1.61
Ralb	1.61
Dlgap4	1.61
Iah1	1.61
Stxbp2	1.61
Fgr	1.61
Far1	1.61
Pet100	1.61
Stoml1	1.61
Nipa2	1.60

Idh1	1.60
Atp6v0a1	1.60
Slc37a2	1.60
Lpcat3	1.59
Cox5b	1.59
Plin2	1.59
Psmd8	1.59
Nckipsd	1.59
Gabarap	1.59
Rnh1	1.59
Ndufa5	1.59
Def8	1.59
Dnajb11	1.58
Cgrrf1	1.58
RT1-CE4	1.58
Myd88	1.58
Uqcrb_2	1.58
Rnasek	1.58
Pgm2	1.58
Hck	1.57
Vps54	1.57
Arpc4	1.57
LOC100360575_1	1.57
Kif1b	1.57
RT1-A2	1.57
Pkm	1.57
Cd99	1.57
Mrpl27	1.56
Hmbs	1.56
Whamm	1.56
Max	1.56
Fbxo6	1.56
Gna15	1.56
Lamp2	1.56
Rere	1.56
RT1-CE16_2	1.56
Slc35a1	1.55
Adgrg5	1.55
Mctp2	1.55
Inf2	1.55
Ube2l3	1.55
Erp29	1.55
Nilr1	1.55
Snd1	1.55
Ptpn1	1.55
Rps6kc1	1.55
Tmed3	1.55
Stat3	1.55

Tnip2		1.54
Ripk1		1.54
LOC108348064		1.54
Plekhb2		1.54
Sh3bgrl3		1.54
Snf8		1.54
Nfkbia		1.54
Lrch1		1.54
Mrpl54		1.53
Slc37a3		1.53
Sat1		1.53
Napsa		1.53
Txn2		1.53
Tcn2		1.53
Uap1l1		1.53
Usp15		1.53
Actb		1.53
Ndufa3		1.52
Tbcb		1.52
Hadha		1.52
Hmgn2		1.52
Cars		1.52
Nme1		1.52
Tfpt		1.52
Asmtl		1.52
Anxa2		1.52
Uqcrq		1.52
Trappc2l		1.52
Acsl5		1.52
LOC100364500		1.52
Atp5j2		1.52
Cln6		1.52
Vdac2		1.51
Mpc1		1.51
Tor1aip2_2		1.51
Pnpla2		1.51
Cox6b1_1		1.51
Tor1aip2_1		1.51
Sh3bp2		1.51
Hmox2		1.51
Mfsd11		1.51
Vps29		1.50
Usf1		1.50
G4		1.50
Rbm43		1.50
Ikbke		1.50
Rn50_X_0744.5	953.89	
Hsp90aa1	276.00	

Zfp622	195.15
LOC108348140	163.03
Syt6	66.32
LOC100362298	53.60
LOC100911956	29.87
LOC100910979_1	8.92
Faah	8.57
LOC100362909	7.99
RGD1565410	7.19
Fat3	6.78
Bcl2l1-ps1	6.53
LOC100911204	6.22
Hist1h3b_1	5.47
AABR07058955.1	5.43
Nek2	5.33
RGD1308751	5.22
Kif14	5.17
LOC680322	5.16
AABR07049695.4	4.76
AABR07049960.1	4.70
Rtkn2	4.68
Ckap2l	4.63
Adra2a	4.57
Ticrr	4.50
Cit	4.44
RT1-M2	4.43
E2f7	4.36
Hells	4.31
Clspn	4.21
Dtl	4.17
Lrr1	4.10
Hjurp	4.08
Pif1	4.03
Cenpi	4.03
Ncs1	3.92
Ddias	3.76
Hmmr	3.72
Bub1b	3.67
Polq	3.66
Cenpu	3.65
Zwilch	3.60
Diaph3	3.59
Oip5	3.54
Anln	3.48
Lrrc72	3.47
Polr2l	3.41
Rad51b	3.38
Kntc1	3.38

Scml2	3.37
Rbfox2	3.31
Xrcc3	3.31
Aspm	3.23
Tnnt1	3.21
LOC102553861	3.11
Car12	3.08
Rad54b	3.08
Efcab11	3.06
Kif24	3.01
LOC103690190	3.01
Anlnl1	3.01
Wwtr1	2.97
Dusp4	2.95
Hist2h3c2_2	2.93
RGD1559962	2.87
ENSRNOG000000576	2.81
Oit3	2.76
Gpc1	2.75
Cdkn2c	2.73
Fsbp	2.71
Dscc1	2.71
Il10	2.68
Brip1	2.67
AABR07000658.1	2.67
Atad5	2.66
Kif18a	2.64
Mms22l	2.63
Rasl10a	2.63
AABR07001512.1	2.60
Tjp1	2.58
Xkr5	2.55
Raet1l	2.54
Fam102b	2.54
Mad2l1	2.51
Vangl1	2.49
MGC116202	2.49
Smc2	2.47
Depdc1b	2.47
Scin	2.47
LOC103690118	2.46
Gas2l3	2.43
Cdh13	2.43
Mbnl3	2.41
Amot	2.38
Mybl1	2.36
Zdhhc15	2.31
Cemip	2.30

Hcn2	2.28
Clic5	2.26
Dsc2	2.26
Galntl5	2.25
Fanci	2.24
Zdhhc21	2.24
Poc1a	2.24
Pask	2.23
Gen1	2.23
Apold1	2.23
Crip2	2.22
Fkbp10	2.21
Clnk	2.20
Sowahc	2.20
Focad	2.20
Ly49s4	2.18
AABR07000398.1	2.16
LOC100359539	2.15
Smtn	2.15
Pola1	2.15
Cep72	2.14
Xrcc2	2.13
Cacng4	2.12
Pkia	2.12
Rad51ap1	2.11
Phf19	2.11
Brca1	2.10
Zfp367	2.08
RGD1562667	2.07
Cdca7	2.07
Il12rb1	2.06
E2f1	2.06
AABR07019083.1	2.05
Papss2	2.05
LOC290595	2.04
Kcnk5	2.03
Nsl1	2.03
Klra1	2.03
Prr5	2.02
Hspa2	2.02
Ndst1	2.00
Klri1	2.00
Ppp1r36	2.00
Dhfr	2.00
Gins3	2.00
Fam124a	1.99
Gins2	1.98
Rnase2	1.97

Arhgap33	1.97
Mcm4	1.96
Spred1	1.96
Mboat2	1.96
Kif19	1.95
Kcnk7	1.95
Eps8l2	1.95
Wdhd1	1.92
LOC102547056	1.91
Ccdc34	1.91
LOC100911830	1.90
Timeless	1.90
Ncapd3	1.90
Prf1	1.89
Pde4a	1.89
Fer	1.89
Hspa8_2	1.89
Cd200r1l	1.88
Bspry	1.88
Lig1	1.88
Atp8b4	1.88
Gnb4	1.87
Myh2	1.87
Sntb1	1.87
Rrm1	1.87
Paqr4	1.86
Rad18	1.86
Cd24	1.86
Exnef	1.86
AABR07015078.2	1.85
Selenon	1.85
Hmgb2l1	1.85
Cdc7	1.85
Tubb5	1.85
Itpripl1	1.84
RGD1306556	1.84
Lmnb2	1.84
Gzmbl3	1.84
Klrg1	1.84
Bora	1.84
Msrb3	1.83
Spdl1	1.83
Klri2	1.82
Clic4	1.81
Ncapg2	1.79
Chaf1a	1.78
Ddx58	1.78
Kcne5	1.78

LOC103693015	1.77
Sdc1	1.77
Naa40	1.77
Rab43	1.77
Orc6	1.77
Tigit	1.76
Mcm6	1.76
LOC100911660	1.76
Fam83h	1.76
Ercc6l	1.76
Ccnd2	1.75
LOC100912917	1.75
Cdhr1	1.75
Eomes	1.75
RT1-CE1	1.75
Nxt2	1.75
Il2rb	1.75
Arsb	1.74
Mcm2	1.74
Mcph1	1.74
Pmf1	1.74
Rbl1	1.72
Klre1	1.72
Faslg	1.71
Hmgb3	1.70
Dab2ip	1.70
Rcn1	1.70
Mtbp	1.70
Gzmk	1.69
Sulf1	1.69
Dock7	1.69
Tlr7	1.69
LOC100911453	1.67
Cep128	1.67
Atp1b1	1.67
Prim1	1.67
Colec12	1.66
Pkmyt1	1.66
Cpped1	1.66
Zfyve16	1.65
Chtf18	1.65
AABR07063424.1	1.65
LOC498265	1.64
Fen1	1.64
Lmo7	1.64
Pole	1.64
Pml	1.64
Tcf19	1.63

AABR07015066.1	1.63
Prkx	1.62
Fbxw8	1.62
Ckap5	1.62
AABR07015055.1	1.62
Sept11	1.62
AABR07015078.1	1.62
LOC361346	1.61
Atad2	1.61
Vps13c	1.60
Luzp1	1.60
AABR07015080.2	1.60
Cxcl16	1.60
Apaf1	1.59
Ran	1.59
Nbn	1.59
Tfdp1	1.58
Cdc25a	1.58
Serpinb9	1.58
Dbf4	1.58
Chn2	1.58
Sfmbt1	1.57
Gusb	1.57
Gpr160	1.57
Slbp	1.57
Pcna	1.57
Jarid2	1.56
Ncr1	1.56
Pwwp2b	1.56
Rpa3	1.56
Kctd9	1.55
Suv39h1l1	1.54
Haus4	1.54
Efhd2	1.54
Insig2	1.54
Gzmm	1.54
Ppih	1.54
Prdm1	1.54
Kdelc2	1.53
Rcc1	1.53
Dpysl2	1.52
Pdxk	1.52
Ptpn4	1.52
Dsn1	1.52
Hn1l	1.51
Idi1	1.51
Sytl2_1	1.51
Lix1l	1.50

Hirip3	1.50	
Vars	1.50	
Il1r2	-2.13	2.07
RT1-DOb	-1.72	-1.51
Sh3bp5	-1.78	-1.53
Cr2	-1.79	-1.54
Cd83	-1.56	-1.55
Snn	-1.57	-1.55
LOC103689968	-1.55	-1.56
Acp5	-1.56	-1.56
B3gnt2	-1.52	-1.57
Clec4a2	-1.51	-1.58
Mef2c	-1.58	-1.59
Tmem206	-1.78	-1.60
Fchsd2	-1.54	-1.61
Fam189b	-1.55	-1.66
Cd3g	-1.52	-1.67
Gcnt2	-1.55	-1.68
Chst3	-1.96	-1.68
Prrt1	-1.60	-1.69
Acss1	-1.50	-1.71
Ctse	-1.55	-1.71
Slamf6	-1.60	-1.74
Kdm5b	-1.50	-1.75
Tfap4	-1.53	-1.77
Sit1	-1.74	-1.77
Btla	-1.89	-1.78
Rab30	-1.92	-1.78
RGD1561730	-1.93	-1.78
Bcar3	-1.94	-1.78
Il4i1	-1.92	-1.79
Rtn4rl1	-1.88	-1.81
Cyp2s1	-1.81	-1.82
Rph3al	-1.60	-1.82
Frat2	-1.66	-1.83
Cd200	-1.88	-1.83
Nuak1	-2.02	-1.83
Rps19	-1.54	-1.86
Rgs12	-1.51	-1.86
Ift80	-1.50	-1.86
Pde2a	-1.64	-1.87
LOC100912181	-1.72	-1.88
Sytl1	-1.52	-1.88
Clcf1	-1.93	-1.89
Hist1h1c	-1.61	-1.89
Itgae	-1.70	-1.90
Clcn4	-1.80	-1.90
Apbb1	-1.61	-1.90

LOC501110	-2.45	-1.91
Tbxa2r	-1.64	-1.94
Reep6	-2.32	-1.95
Slc45a3	-2.30	-1.96
Mgat4a	-1.55	-1.97
Cfap44	-2.13	-1.97
RGD1306739	-2.51	-1.98
Cd86	-1.61	-2.00
Cd8b	-1.61	-2.00
Stard13	-1.82	-2.01
Naaa	-1.72	-2.03
Irf6	-1.61	-2.03
Tmbim1	-2.37	-2.04
Jup	-1.61	-2.04
Cbx7	-1.74	-2.04
Tmem163	-1.84	-2.05
Amigo1	-1.60	-2.05
RGD1560281	-1.66	-2.06
RGD1561143	-1.70	-2.06
LOC685157	-1.69	-2.06
Trat1	-1.53	-2.07
Agl	-1.55	-2.07
Sphk1	-1.67	-2.08
Hs3st1	-1.93	-2.08
Pik3ip1	-1.69	-2.08
Art4	-1.53	-2.09
Gpr68	-1.57	-2.09
Cd300lb	-1.95	-2.10
Fcgbp	-1.59	-2.12
Ccr6	-1.88	-2.12
Kcnh2	-1.68	-2.14
Axin2	-1.50	-2.14
Xkrx	-1.89	-2.15
Podnl1	-1.83	-2.16
Cmah	-1.67	-2.16
Sh2b2	-2.26	-2.16
Sesn1	-1.68	-2.17
ENSRNOG000000598	-1.74	-2.17
Tmem204	-1.73	-2.18
Mef2b	-1.70	-2.19
AABR07017846.1	-1.67	-2.20
Gtf2ird1	-1.77	-2.21
Hvcn1	-1.78	-2.21
Ccr7	-1.58	-2.25
Rn50_10_0706.2	-1.98	-2.26
Trpm6	-1.78	-2.26
Slc43a1	-1.70	-2.29
Ralgps2	-1.66	-2.30

Mex3b	-1.64	-2.30
ENSRNOG000000480	-2.23	-2.30
Pknox2	-2.05	-2.32
Fcer2	-2.18	-2.34
Pdia5	-1.81	-2.34
Angptl4	-2.11	-2.36
Baiap3	-1.81	-2.38
Slc12a3	-2.07	-2.39
G0s2	-2.65	-2.40
Exoc3l2	-2.02	-2.40
RGD1564664	-2.23	-2.40
Marveld1	-1.81	-2.40
AABR07051533.2	-1.94	-2.41
Qprt	-1.99	-2.44
Eno3	-1.83	-2.45
Amz1	-1.68	-2.46
Kcnj1	-2.77	-2.48
Pdzd4	-1.97	-2.48
Dusp5	-2.01	-2.49
Prnp	-1.92	-2.50
Gpr150	-2.25	-2.51
AABR07065889.2	-2.99	-2.52
Hepacam2	-2.35	-2.52
Fcgr2b	-1.84	-2.53
Pparg	-1.99	-2.53
Apoe	-1.76	-2.53
Il9r	-2.09	-2.54
Ephx1	-1.68	-2.54
Ly6g6d	-2.42	-2.54
Scn4a	-2.73	-2.55
Spn	-1.56	-2.58
Ankrd33b	-2.06	-2.59
Mid2	-2.11	-2.60
LOC102556148	-2.25	-2.62
Golm1	-1.59	-2.63
Ccr9	-1.63	-2.65
Mpzl2	-1.96	-2.66
Gpr176	-2.70	-2.68
Pak6	-2.36	-2.69
Dmrtc2	-2.18	-2.71
Gpr183	-1.98	-2.75
Rn60_20_0030.4	-2.89	-2.76
Ache	-2.23	-2.77
L1cam	-2.29	-2.81
Rcn3	-2.37	-2.81
Nr1d1	-1.71	-2.82
Ldlrad3	-1.70	-2.86
AABR07063740.2	-2.31	-2.86

Cav2	-1.98	-2.87
Ndrg2	-2.16	-2.91
Cadm3	-1.83	-2.93
Fst	-2.31	-2.98
RT1-Db2	-2.32	-3.01
Btnl9	-2.42	-3.06
Soat2	-2.97	-3.13
Rhoc	-2.09	-3.14
Ptk2	-1.79	-3.17
LOC102552344	-3.96	-3.18
Dao	-2.97	-3.19
LOC681341	-1.81	-3.21
Ascl2	-2.39	-3.23
Kcnh3	-4.52	-3.28
Gdf3	-2.27	-3.31
RGD1566226	-2.48	-3.31
Fpr2	-2.94	-3.37
Cd4	-1.73	-3.42
Rapgefl1	-2.40	-3.44
Ngfr	-1.95	-3.48
AABR07060995.1	-7.52	-3.49
LOC690288	-2.87	-3.51
AABR07021946.1	-4.01	-3.52
Dhrs3	-1.74	-3.55
Cxcr6	-1.89	-3.59
Snta1	-2.72	-3.60
Papln	-3.07	-3.72
Rhbdf1	-2.12	-3.76
Cpne7	-2.72	-3.96
Pon3	-2.95	-3.96
Akr1b10	-2.15	-4.05
Sgpp2	-7.70	-4.20
Sspo	-3.82	-4.21
Mrc2	-2.70	-4.24
Srms	-3.60	-4.24
Maob	-2.95	-4.33
Defb14	-4.67	-4.39
Prkd1	-2.88	-4.42
Semg1	-3.32	-4.73
Lamb3	-3.34	-4.85
Nwd1	-2.62	-4.85
Olr292_1	-3.19	-4.90
Kcnj5	-5.53	-4.91
Dnah6	-2.70	-4.93
Hsd11b1	-1.89	-4.98
Ccr4	-2.35	-5.03
AABR07036301.1	-3.35	-5.29
Sult1a1	-5.43	-5.36

Maged1	-6.93	-5.39
Kcns1	-2.80	-5.54
Espn	-2.09	-5.57
Plekhg1	-4.03	-5.85
RGD1563354	-3.24	-5.90
Alpk2	-2.24	-6.07
Cecr6	-3.04	-6.12
Rorc	-2.41	-6.14
Hes1	-3.56	-6.21
Il17re	-3.51	-6.21
Acsbg1	-5.12	-6.61
AABR07013095.1	-9.08	-6.81
Qrfpr	-3.39	-6.90
Flnb	-2.69	-6.93
Tgfa	-4.74	-7.04
Cd36	-3.17	-7.13
Sdk2	-2.59	-7.27
Myh14	-2.39	-7.60
Aplnr	-6.59	-7.69
Syt15	-3.32	-7.80
Il23r	-3.64	-7.83
Tspan18	-3.59	-8.19
B3gnt3	-4.32	-8.57
LOC100912498	-11.63	-9.58
Capn3	-5.57	-10.21
AABR07034428.1	-3.38	-10.21
Lipm	-7.44	-10.59
Efs	-10.03	-12.68
Rtn1	-3.51	-15.99
Spata18	-10.98	-34.45
Ceacam6	-23.41	-47.18
Nfs1_2	-65.81	-53.16
Slc36a2	-5.60	-78.19
Map1a	-3.86	-90.08
Tnks1bp1		-1.50
Fam117b		-1.50
Ablim1		-1.51
Ehd3		-1.51
Numa1		-1.51
Usp28		-1.51
Vps37b		-1.51
Setx		-1.51
Larp1b		-1.51
Malt1		-1.52
Epc2		-1.52
Limk1		-1.52
Per1		-1.52
Rai1		-1.52

Metrnl	-1.52
Tmem123	-1.52
Rnf2	-1.53
Smad3	-1.53
Gba2	-1.53
Elk3	-1.53
Mbtps1	-1.53
Hdac7	-1.53
Atp2b4	-1.53
Cdkn2aip	-1.53
Klrd1	-1.53
Entpd4	-1.53
Lancl1	-1.54
Smagp	-1.54
Cd7	-1.54
Chd6	-1.54
Eif2ak3	-1.54
Rpa1	-1.55
Capn5	-1.55
Sgms1	-1.55
Grap2	-1.55
Stk24	-1.55
Tcp11l2	-1.56
Ssbp3	-1.56
Bicdl1	-1.56
Tmem57	-1.57
Crlf3	-1.57
Arntl	-1.57
Nipal1	-1.57
Sec22a	-1.57
Cdkn1b	-1.57
Chd3	-1.58
Nfatc3	-1.58
Pop5	-1.58
Lgmn	-1.58
Nap1l1	-1.58
Cd244	-1.59
Smarca2	-1.59
Pomt2	-1.59
Stap1	-1.59
Arl11	-1.59
RGD1305464	-1.59
St8sia1	-1.60
Itk	-1.60
Scml4	-1.60
Pde4d	-1.60
Jakmip1	-1.60
Gpr174	-1.60

Dnajb4	-1.60
Cd3e	-1.61
Zfp869	-1.61
Cyp4f5	-1.61
Cdk17	-1.61
Gsg2	-1.61
AABR07035722.1	-1.61
March7	-1.61
Map4k2	-1.61
Itpr3	-1.62
Strbp	-1.62
Mllt6	-1.62
Adamts10	-1.62
Il27ra	-1.62
Tp53i11	-1.62
Tbc1d9	-1.63
Syt11	-1.63
Mrm1	-1.63
Mettl7a	-1.64
Tox	-1.64
Adamts14	-1.64
Klf2	-1.64
Wdr6	-1.64
Ankrd10	-1.65
Cd6	-1.65
Ezr	-1.65
Crebzf	-1.65
Mbp	-1.65
Zfp62	-1.65
RGD1560455	-1.65
Lef1	-1.65
Fbxl4	-1.65
Zfp472	-1.65
Zfp318	-1.65
Zadh2	-1.66
Zfp512b	-1.66
Ptpn22	-1.66
Mvb12b	-1.66
LOC102551095	-1.66
Nr1d2	-1.66
Ccpg1	-1.66
Itgb2	-1.66
Slc9a9	-1.66
Fmnl3	-1.66
LOC102549869	-1.66
Nxpe1	-1.66
Rflnb	-1.67
LOC499331	-1.67

Ptger4	-1.67
Gstt2	-1.67
Bcl11b	-1.67
Heca	-1.67
Tp53bp2	-1.67
Ceacam1	-1.67
Mycl	-1.67
Lpin1	-1.68
Fam78a	-1.68
Slc4a11	-1.68
Tspan32	-1.68
Scaper	-1.69
Rab3ip	-1.69
Ubash3a	-1.69
Skap1	-1.69
Rnf125	-1.69
Ccl5	-1.69
Tecpr1	-1.69
Satb1	-1.69
Map3k10	-1.69
Rbl2	-1.69
F2r	-1.70
Lpar6	-1.70
Gigyf1	-1.70
Dirc2	-1.70
Nedd4	-1.70
Adcy3	-1.70
Cd55	-1.70
Elovl5	-1.71
Slc14a1	-1.71
St6gal1	-1.71
AABR07027872.1	-1.72
Map2k6	-1.72
Stk26	-1.72
Kat6b	-1.72
Txndc16	-1.72
Cdc42bpg	-1.73
Rab37	-1.73
Fam65b	-1.73
LOC100910669	-1.73
Ctsl	-1.73
Arrdc3	-1.73
AABR07034669.1	-1.73
Bhlhe40	-1.73
Kiz	-1.74
Gimap6	-1.74
Zfat	-1.74
Ccdc142	-1.74

Hdgfrp3	-1.75
Arhgef18	-1.75
Baiap2	-1.75
Bmi1	-1.75
Ms4a6c	-1.75
Pde3b	-1.75
Gpr155	-1.75
Bcl9l	-1.75
Ankh	-1.76
Cep97	-1.77
Mad1l1	-1.77
Cd40lg	-1.77
Lrig1	-1.77
Dpp4	-1.78
Cobll1	-1.78
Pgm2l1	-1.78
Plcg1	-1.78
Gtf2i	-1.79
Klf4	-1.79
Pglyrp2	-1.79
Hs3st3b1	-1.79
Mcf2l	-1.80
Foxo1	-1.80
Trbv1	-1.80
Tdrkh	-1.80
Tacc2	-1.81
LOC102549506	-1.81
Sirt4	-1.81
Icos	-1.81
Sh3bp4	-1.81
LOC100911215	-1.81
Pacs1	-1.82
Thra	-1.82
LOC100911581	-1.82
Wipf3	-1.82
Zbed3	-1.83
Asph	-1.83
Tbc1d30	-1.83
Tnk1	-1.83
Zfp956	-1.83
ENSRNOG00000043164	-1.83
Pld3	-1.84
Mras	-1.84
Bcl2	-1.84
Sfxn3	-1.84
AABR07065693.3	-1.85
Adcy6	-1.85
AABR07001634.1	-1.85

AABR07065699.2	-1.85
S1pr1	-1.85
Ephb6	-1.85
Bbs10	-1.85
Btbd8_1	-1.86
Ets1	-1.86
Kbtbd11	-1.86
ENSRNOG00000056059	-1.86
AABR07065750.2	-1.86
ENSRNOG00000051999	-1.86
Mfge8	-1.87
Carns1	-1.87
Fbxo32	-1.87
Zfp362	-1.88
Gata3	-1.88
Begain	-1.88
Cyp2u1	-1.88
Ccl6	-1.89
Itpkb	-1.89
Sun2	-1.89
Zc3h6	-1.90
Prelid2	-1.91
Plekha7	-1.91
RGD1565033	-1.92
Spint1	-1.92
Ctsf	-1.92
Zfp3	-1.92
Cep170b	-1.92
Htra3	-1.92
LOC100911271	-1.92
Add3	-1.92
Fbxo21	-1.93
Lsr	-1.93
Mapk15	-1.94
Rgcc	-1.94
Gfra3	-1.95
Ldlrap1	-1.95
P2ry10	-1.95
AABR07051708.1	-1.95
LOC100360581	-1.97
Zfp385c	-1.97
Speg	-1.97
Slc29a1	-1.97
RGD1561277	-1.99
LOC100909671	-1.99
Cd101	-1.99
AABR07065827.1	-2.00
Wls	-2.00

Heg1	-2.00
Srcin1	-2.01
Thbd	-2.01
Tnfsf8	-2.01
Laptm4b	-2.01
AABR07051592.2	-2.01
Tnfrsf13b	-2.02
LOC102553866	-2.02
AABR07017635.2	-2.02
LOC100912262	-2.02
Eya2	-2.02
LOC100912707	-2.03
Lima1	-2.03
Mturn	-2.04
Ppm1j	-2.04
Thrb	-2.05
AABR07065699.3	-2.05
Adgre1	-2.05
Unc13b	-2.06
Ms4a4c	-2.07
Enpp1	-2.07
Hdac11	-2.07
Abcg2	-2.08
Bmp7	-2.08
Zrsr1	-2.08
AABR07017634.1	-2.09
Spata6	-2.09
Ly9	-2.09
Csdc2	-2.09
Mtss1	-2.09
Aff3	-2.10
Usp44	-2.10
Zbtb46	-2.10
Adgra2	-2.11
Lpar1	-2.11
Jade2	-2.11
Jmy	-2.12
Cd28	-2.12
Crmp1	-2.12
Fn1	-2.13
Pard6b	-2.13
Kcnab3	-2.13
Tcf7	-2.14
Plekhf1	-2.14
Sv2b	-2.15
Lrrc34	-2.16
Tspoap1	-2.16
Maf	-2.17

C3ar1	-2.17
LOC691170	-2.17
Ccl9	-2.18
Slc25a23	-2.18
Ca5b	-2.19
Cenpv	-2.20
RGD1565989	-2.20
LOC100360891	-2.20
AABR07017649.2	-2.21
Zfp395	-2.21
Nr4a1	-2.22
March3	-2.23
Pcsk1	-2.23
Sparc	-2.23
AABR07017626.1	-2.23
Adarb1	-2.24
Zhx2	-2.24
Miga1	-2.25
Irs3	-2.25
AABR07065676.1	-2.25
Dapk1	-2.26
Tlr12	-2.28
Timp2	-2.28
Themis	-2.30
Sspn	-2.30
Icam5	-2.31
B3galt5	-2.31
AABR07061036.1	-2.32
AABR07027447.1	-2.32
Zfp605	-2.32
AABR07051639.1	-2.32
LOC100911980	-2.33
S1pr5	-2.34
Camk2a	-2.35
Arhgap18	-2.35
Kif7	-2.36
Jchain	-2.36
Mrc1	-2.36
Tbc1d4	-2.37
AABR07017868.1	-2.37
Klf9	-2.37
Clic2	-2.37
Ncald	-2.37
LOC103690166	-2.37
Palld_2	-2.38
AABR07051533.1	-2.39
Pald1	-2.40
Ccnd1	-2.41

Smim5	-2.42
Mmrn2	-2.42
Casc4	-2.42
Boc	-2.42
Ago4	-2.42
Pou6f1	-2.43
Ptprk	-2.44
Rn50_5_0814.26	-2.44
Kit	-2.46
AABR07065714.1	-2.46
AABR07017716.1	-2.46
Morc1	-2.46
Dzip1	-2.47
AABR07062800.1	-2.48
AABR07017868.3	-2.49
AABR07017875.1	-2.49
Rnf150	-2.50
Ppp1r14c	-2.50
Ptprs	-2.50
Rasl11b	-2.50
AABR07062157.1	-2.51
AABR07065699.1	-2.51
Dpysl5	-2.52
Tiam2	-2.52
AABR07065837.1	-2.53
Nt5e	-2.53
Atp9a	-2.53
Tnfsf11	-2.53
Eng	-2.54
LOC100910801	-2.54
Tmem136	-2.55
Fam198b	-2.56
AABR07008568.1	-2.56
Prss3b	-2.57
Tnn	-2.57
Magi1	-2.58
Tmcc3	-2.59
LOC685048	-2.59
Rassf6	-2.60
Vegfc	-2.61
Als2cr12	-2.61
Cd109	-2.62
Tas2r140	-2.62
Rab7b	-2.63
AABR07061001.1	-2.65
AC120076.1	-2.65
Enpp2	-2.65
Tspyl4	-2.66

Cx3cr1	-2.66
Hfe	-2.66
Slc16a7	-2.66
Mfsd4	-2.67
Rab11fip5	-2.68
Adgra3	-2.68
Plch2	-2.68
Dbn1	-2.69
Fat1	-2.70
Flt3	-2.70
Cnga1	-2.71
Tcf7l1	-2.71
LOC100910838	-2.71
AABR07060979.1	-2.72
Fam19a3	-2.75
Rflna	-2.76
Armc2	-2.77
AABR07065815.2	-2.78
Carmil3	-2.79
Lama2	-2.79
Nfia	-2.79
AABR07065823.1	-2.79
Crtam	-2.80
LOC679149	-2.81
Camk2n1	-2.81
Rapgef5	-2.82
Pla2g2f	-2.84
AABR07065699.4	-2.84
Adgrg1	-2.85
LOC100361105	-2.86
Try5	-2.86
Adrb1	-2.86
LOC100359978	-2.88
B4galt4	-2.92
LOC103691157	-2.93
Ildr1	-2.93
Ttc25	-2.96
LOC103690131	-2.97
RGD1561149	-2.97
Rfx2	-2.97
Kcng2	-2.98
Apobec2	-2.99
AABR07065821.1	-2.99
AABR07060963.1	-3.00
Apcdd1l	-3.01
Tlr11	-3.02
ENSRNOG00000060184	-3.02
Fbll1	-3.04

AABR07065750.3	-3.05
Prg4	-3.05
Psd	-3.08
Cfh	-3.08
AABR07065680.1	-3.10
AABR07065815.1	-3.11
Plcl1	-3.12
Cspg5	-3.13
Pros1	-3.14
AABR07065688.1	-3.15
NEWGENE_2319083	-3.17
Stab1	-3.17
Lama3	-3.17
Efhd1	-3.20
Clec9a	-3.21
Gcsam	-3.21
RGD1566029	-3.21
Trim16	-3.22
Hpdl	-3.24
AABR07065693.1	-3.25
Ccdc80	-3.30
Cd276	-3.32
Afdn	-3.33
Lhfp	-3.33
Vnn3	-3.34
Nxph3	-3.36
Dixdc1	-3.42
Glis2	-3.45
F2rl1	-3.46
RGD1565536	-3.46
LOC100361052	-3.46
Tspyl5	-3.47
Gjb2	-3.47
Snx22	-3.47
Gdf1	-3.49
Pacsin3	-3.50
Cadm1	-3.51
Golga7b	-3.54
RGD1305807	-3.59
LOC103694853	-3.61
Selenop	-3.63
Tgfbr3	-3.64
Hbegf	-3.65
Il1rl1	-3.67
She	-3.68
Gria1	-3.70
Gpr34	-3.74
AABR07067267.1	-3.75

Vsig10	-3.79
Col4a4	-3.81
Hgd	-3.83
AABR07065778.3	-3.86
Adamts12	-3.86
RGD1561662	-3.92
Fbxl21	-3.95
Bhlhe22	-3.96
Slc13a5	-3.97
Calml4	-3.98
Lpl	-4.03
Lhb	-4.04
Cldn10	-4.06
Rarb	-4.08
Plppr3	-4.09
Carmil1	-4.13
Scn5a	-4.15
Il12b	-4.23
Dnase1l3	-4.28
Grm4	-4.29
Rn60_20_0053.4	-4.33
Spdya	-4.33
Emp1	-4.33
Epha2	-4.36
Cma1	-4.36
Col16a1	-4.37
Kcnj12	-4.38
Sh3gl3	-4.39
Zbtb16	-4.40
Dsp	-4.42
Fbln7	-4.45
AABR07027866.1	-4.46
ENSRNOG00000033109	-4.46
Edar	-4.49
Cd163l1	-4.49
Enpp3	-4.51
Pld6	-4.53
Trem2	-4.54
Nbl1	-4.55
Rgl1	-4.56
Thbs3	-4.59
Pomgnt2	-4.59
AABR07028523.1	-4.63
Irs1	-4.64
Hspb1	-4.65
Cacnb4	-4.72
Fcrl2	-4.73
RGD1310819	-4.74

Cux2	-4.87
Cd5l	-4.88
Wnt4	-4.92
Lif	-5.01
Stxbp6	-5.05
Mypn	-5.05
Tppp3	-5.10
AABR07021022.1	-5.10
ENSRNOG00000047191	-5.11
LOC100911403	-5.14
Plxna2	-5.22
Hlf	-5.44
Xcr1	-5.47
Apoc3	-5.69
Fblim1	-5.80
Cd300lg	-5.81
Gucy1a2	-5.82
Pltp	-5.90
Sash1	-6.00
Nov	-6.01
St6galnac5	-6.05
Sertm1	-6.08
Plk2	-6.10
AABR07006033.1	-6.11
Ms4a4a	-6.19
Popdc2	-6.38
Cacng8	-6.44
Serpinb8	-6.47
RGD1566368	-6.50
Gprc5d	-6.56
Fabp4	-6.60
Igdcc4	-6.64
Mpp3	-6.66
Nr3c2	-6.98
Ecm1	-7.06
Abca17	-7.13
Spaca9	-7.29
Ptprg	-7.45
Mcpt1l1	-7.57
Pnmal2	-7.65
Rnf39	-7.73
Tph1	-7.73
Ltbp1	-7.75
Ryr2	-7.84
Asic1	-8.24
RGD1564571	-8.29
Tek	-8.41
Nid2	-8.42

Cgnl1	-8.44
Cav1	-8.62
Slc16a2	-8.65
Scn1b	-8.67
Fam3b	-8.69
Arhgap32	-8.76
Gprc5a	-9.01
Zfp939	-9.10
Heph	-9.37
Fgfr1	-9.52
C1ql3	-9.55
AABR07030598.1	-9.55
Marc1	-9.94
Nptx1	-10.03
Fscn1	-10.03
Hpgd	-10.08
Gjc1	-10.19
AABR07034991.1	-10.22
Shtn1	-10.35
Shank3	-10.87
Cpa3	-11.01
C1qc	-11.82
Folr2	-12.03
Ccl2	-12.27
C1qa	-12.44
Duoxa1	-12.48
Lyve1	-12.68
Mamstr	-13.00
Cd34	-13.02
Selp	-13.16
Gas6	-13.27
Ltc4s	-13.27
Timd4	-13.69
Psca	-13.71
Sost	-13.83
Cacna1c	-13.96
Aox3	-13.96
Pdlim4	-14.04
Fam20a	-14.24
Slc9a3r2	-14.24
Tgfb2	-14.79
Angptl8	-14.79
Fam13a	-14.81
Prox1	-14.81
Pmp22	-15.26
Asgr2	-15.36
Sgsm1	-15.64
Nrxn2	-16.06

Tcam1	-16.11
Bcar1	-16.19
Slc2a10	-16.98
C1qb	-17.35
Tpsb2	-17.56
Adam23	-17.96
Tpsab1	-18.68
Cxcl1	-18.82
Mlxipl	-19.06
Ptrf	-20.02
Dchs1	-22.01
Cndp1	-23.82
Igfbp6	-26.51
Tnxb	-27.29
Ptgis	-27.46
Ntm	-27.55
Lpo	-27.90
Olfml2a	-29.03
Axl	-29.54
Vil1	-31.79
PCOLCE2	-32.81
Cyp26a1	-33.32
Ms4a8	-36.27
Cxcl14	-37.29
Gpr149	-37.32
Inhbb	-40.55
Fxyd6	-40.81
Ednrb	-41.18
Gata6	-42.06
Sptbn2	-42.07
Wnt2	-42.91
C6	-42.96
Grik3	-48.53
LOC361016	-49.08
Ccl24	-54.73
Ltbp3	-55.39
Gpr39	-56.94
LOC103691013	-57.38
Ccl7	-57.63
Cd163	-63.04
Arhgef5	-64.59
C2cd4b	-68.81
Srgap1	-72.60
LOC100910163	-77.34
Ntsr1	-79.06
Asz1	-79.10
AABR07020815.1	-80.40
Mamdc2	-96.69

Rps4x_1		-111.90
Mmp2		-287.50
Rpl35al1		-350.77
Dgka	-1.50	
Hip1r	-1.51	
Kcnn4	-1.51	
Slc22a17	-1.51	
Mif4gd	-1.51	
Icoslg	-1.52	
Gstm1	-1.52	
Lmbr1l	-1.52	
Rhoh	-1.53	
Cd3d	-1.53	
Ifi27l2b	-1.53	
Siglec10	-1.54	
Tmem80	-1.54	
LOC102555672	-1.55	
Cyp4f17	-1.55	
Neil1	-1.56	
Lax1	-1.56	
Tnfrsf26	-1.56	
As3mt	-1.56	
Snx8	-1.57	
Polg2	-1.57	
LOC100361706_1	-1.58	
ENSRNOG000000341	-1.59	
S1pr4	-1.59	
LOC100911585	-1.60	
Foxp3	-1.60	
Blnk	-1.60	
Pot1b	-1.61	
Dus2	-1.61	
Tnfrsf25	-1.62	
Pou2af1	-1.62	
Cirbp	-1.62	
Ypel3	-1.64	
AABR07060872.1	-1.65	
Phospho1	-1.66	
Col20a1	-1.66	
Zc3h12d	-1.66	
Rnf144a	-1.66	
Hmgn3	-1.66	
Cxcr5	-1.67	
Kifc2	-1.68	
Rpusd3	-1.68	
Trim46	-1.69	
Lbp	-1.69	
Zfp296	-1.69	

Blk	-1.70
Phf1	-1.72
ENSRNOG000000480	-1.73
RT1-DOa	-1.76
LOC100361706_2	-1.77
AABR07051551.1	-1.79
Adam8	-1.80
Cd19	-1.83
Mterf2	-1.83
Ptp4a3	-1.84
LOC103690163	-1.84
Fcrla	-1.86
Pax5	-1.87
Chst10	-1.88
Fcrl1	-1.89
Bend5	-1.91
Vps37d	-1.94
RGD1559588_1	-1.95
LOC688553	-1.96
AC134759.1	-1.96
Rn60_2_1868.2	-1.97
Ppdpf	-1.97
Tmem38a	-1.98
Khdrbs3	-1.99
Fcmr	-2.00
Ell3	-2.01
Srpk3	-2.03
Pde6h	-2.03
Cd79b	-2.04
Olah	-2.05
Cd79al	-2.05
Sult2b1	-2.06
Pigr	-2.06
Ddx25	-2.06
AABR07041096.1	-2.07
Stfa2l2	-2.08
Pxdc1	-2.09
Cd79a	-2.10
Ramp3	-2.12
AABR07017825.5	-2.12
Nsg1	-2.13
Hes5	-2.15
AC141997.1	-2.17
AABR07061052.1	-2.21
Coro6	-2.22
Sox15	-2.25
RT1-Ha	-2.25
Npff	-2.27

Fam212a	-2.29
Prok2	-2.33
Rbpjl	-2.39
AABR07051733.1	-2.45
Card10	-2.48
Gpr4	-2.48
Il22ra2	-2.51
Rpl13	-2.53
Stra6	-2.58
Smtnl2	-2.59
AABR07073391.1	-2.63
Stfa2	-2.64
Htr2a	-2.67
Adgrv1	-2.68
LOC102550441	-2.74
Klhl41	-2.81
Gjc2	-2.97
AABR07022072.2	-2.99
Pglyrp4	-3.18
AABR07035650.1	-3.19
Il17rc	-3.20
LOC689230	-3.45
AABR07021996.1	-3.50
Cnih3	-3.51
Lrrc66	-3.52
Bpi	-3.67
LOC103690326	-3.69
LOC100360573	-3.80
Scgb3a1	-4.04
Lpar4	-4.07
AABR07051565.1	-4.20
Pgam2	-5.32
Cetn3	-5.57
LOC102548472	-5.74
Klhdc8a	-6.07
Enpep	-6.72
Dio3	-9.48
Dcun1d2	-144.55

RNAseq gene expression. Day 7
Fold change: < 1.5 or >1.5
FDR p-value: < 0.05

	LVS / PBS	ΔclpB / PBS
	Day 7	Day 7
Spink2	135.8	976.2
Gzmc	175.4	250.9
Selenbp1	57.3	61.8
LOC100911956	72.5	55.0
AABR07034729.1	25.8	50.6
Hprt1	61.9	36.3
Gng3	8.3	28.1
LOC257642_3	27.1	19.0
Prss3	9.5	18.4
Il34	3.6	15.8
Rn60_7_0127.2	10.4	14.1
Msc	6.1	13.0
ENSRNOG000000588	8.1	12.5
Mt2A	4.0	12.4
Dclk1	3.1	11.5
Try5	4.1	10.6
Rn60_10_0910.5	16.5	10.3
AABR07072400.1	12.9	10.0
RGD1304810	4.2	8.5
Ahsp	2.9	6.9
Cdkn2a	3.1	6.7
Rn60_19_0375.6	10.3	6.7
Gsta4	3.8	6.7
Cdkn3	2.4	6.5
LOC103689947	3.5	6.4
Chchd2	3.2	6.4
Tp73	3.9	6.2
Slc4a1	3.0	6.1
Prkcdbp	5.0	6.0
Ramp2	3.2	5.9
LOC257642_1	10.7	5.6
Aqp1	2.9	5.6
LOC100910138	3.8	5.4
Cd52	3.7	5.4
Pttg1	2.5	5.4
RGD1308065	2.8	5.3
Plekhb1	3.1	5.2
Lgals5	2.3	5.1
Rhd	2.9	5.0
Ank1	3.3	5.0
Csrp2	4.0	4.9

	Up-regulated genes	
	Count	
	**LVS**	**ΔclpB**
**Both vaccines**	157	157
**LVS only**	162	
**ΔclpB only**		522
**Total Up-regulated**	319	679

	Down-regulated genes	
	Count	
	**LVS**	**ΔclpB**
**Both vaccines**	175	175
**LVS only**	719	
**ΔclpB only**		286
**Total down-regulated**	894	461
**Total genes**	1213	1140

Akr7a3	3.0	4.9
Capn9	5.0	4.9
Ube2c	2.1	4.8
Kel	2.7	4.8
Pianp	3.7	4.6
Tmod1	3.6	4.6
Mt-nd5	2.2	4.5
Crybb3	4.6	4.2
AABR07008940.1	4.6	4.1
Snca	3.4	4.1
Tead3	4.3	4.1
Add2	2.6	4.1
Hba-a2_1	3.5	4.1
Fn3k	4.1	4.0
Rec114	3.5	4.0
LOC100361706_1	3.2	4.0
LOC103694857	3.5	4.0
LOC103694855	3.3	3.9
Best1	3.6	3.9
Gzmm	2.1	3.7
Hbb	3.4	3.7
Hba-a2_2	3.4	3.6
Klrg1	2.5	3.6
Birc5	2.1	3.6
RGD1310507	2.1	3.6
Nova2	3.1	3.6
RGD1559972	3.1	3.6
Mt1	2.4	3.6
Knstrn	2.0	3.6
Alas2	3.1	3.5
Fech	2.6	3.5
Ly49s4	3.1	3.5
Rpl26-ps1	3.5	3.5
AABR07015078.2	6.9	3.5
Sptb	2.5	3.5
Nkg7	2.2	3.4
Slc38a5	3.4	3.4
Tfdp2	2.1	3.4
Klra1	2.6	3.4
RGD1309350	1.9	3.3
Bpgm	2.8	3.3
Tlcd1	3.7	3.2
Gzmk	1.9	3.1
Ccl5	2.0	3.0
Stmn1	1.8	3.0
Mt-cyb	2.0	3.0
Mt-nd2	1.8	2.9

Ly49s6	2.4	2.9
Kifc1	1.8	2.8
Mt-co1	1.6	2.8
Tbx21	2.4	2.8
Hmgb2	1.8	2.7
Cenpm	2.1	2.7
Igh-6	2.5	2.7
Mt-cox3	1.7	2.6
Mt-nd1	1.9	2.6
Ybx3	2.2	2.6
Cdca7	1.8	2.6
Cd2	1.8	2.6
Klre1	1.7	2.6
Mt-atp6	1.8	2.5
Mt-nd4	1.8	2.5
AC120310.1	3.4	2.5
Tmem86b	2.0	2.5
Tmcc2	1.9	2.5
Tspan33	2.2	2.5
Hagh	2.0	2.5
Lag3	2.1	2.5
Sub1	1.6	2.4
Arhgef39	2.4	2.4
AABR07034641.1	2.5	2.4
Cdc25b	1.8	2.4
Cdr2	2.1	2.3
Glrx5	2.1	2.3
Klra5_4	2.5	2.3
Rsad2	2.0	2.3
Car2	1.8	2.3
Dusp2	1.7	2.2
Sh2d2a	1.8	2.2
Sec61g	1.6	2.2
Prdx2	1.6	2.2
AABR07030901.1	2.2	2.2
Cfap126	2.6	2.2
Slc25a39	2.0	2.2
Mt-co2	1.6	2.1
Hist3h2ba	1.9	2.1
Etfb	1.8	2.1
Rpl31l4	2.6	2.1
Pcgf2	2.2	2.1
Fam213a	2.0	2.1
Mzb1	1.8	2.1
Cercam	1.9	2.0
Haus4	1.7	2.0
Dhrs11	1.7	2.0

Phgdh	1.6	2.0
LOC108350502	1.8	1.9
Ly49i5	2.2	1.9
Serpinb6b	1.7	1.8
Cisd3	1.8	1.8
Gfi1	1.6	1.8
H1fx	2.0	1.8
Ptprcap	1.7	1.8
B4galnt1	1.6	1.8
RGD1311739	1.7	1.8
Fis1	1.8	1.8
Pkmyt1	1.7	1.7
Cdhr1	1.6	1.7
Ubac1	1.6	1.7
ENSRNOG000000143	1.7	1.7
Bola2	1.6	1.7
Lsm4	1.7	1.7
Pde6d	1.5	1.6
Pfdn1	1.5	1.6
Ccdc12	1.6	1.6
LOC100361706_2	1.8	1.6
Isca1	1.5	1.6
Rpl32_2		7097.3
Camk1g		206.3
RGD1561252		192.8
RGD1305184		185.7
Slc17a7		57.6
Xirp1		23.9
Cacna1b		18.5
Slc29a2		15.5
Stra8		15.1
Orm1		12.5
Batf2		12.1
Cldn3		11.3
Pdzd9		10.7
C1qtnf9		10.4
Slc40a1_1		9.9
Katnal2		9.7
Btnl10		9.2
Acod1		9.1
Rmi2		7.7
Cd177		7.7
Gpx7		7.6
Rn60_7_0127.5		7.6
Gfap		7.5
Car4		7.4
LOC100910792		6.8

Ifitm6	6.8
Lctl	6.6
Col22a1	6.5
Bace2	6.5
Gpc1	6.5
AABR07065821.1	6.2
LOC100909700	6.1
Kctd17	5.8
Ubd	5.7
Mybl2	5.6
Arl14epl	5.6
Ns5atp9	5.5
Cdk1	5.4
Fkbp1b	5.2
AC135026.1	5.2
Ccne1	5.1
AABR07028113.1	5.0
Hist1h3a	5.0
RGD1559962	4.9
Cdc6	4.9
LOC100912611	4.7
Ankrd22	4.7
Prss34	4.7
Ccnb1	4.7
Ska1	4.6
E2f8	4.5
Cenpw	4.5
Asf1b	4.5
Sapcd2	4.5
Cdca3	4.5
Mlf1	4.5
Eme1	4.4
Ifng	4.4
ENSRNOG00000056226	4.3
Plk1	4.3
Hbq1b	4.3
Myo16	4.2
Kif2c	4.2
Ccna2	4.2
Exo1	4.2
Ngp	4.2
AABR07058511.1	4.2
MGC105567	4.1
Mki67	4.1
Depdc1	4.1
Hist1h2bk	4.0
Mmp14	4.0

Prg2	4.0
Ccnf	4.0
Gins1	4.0
Lrr1	3.9
Kif20a	3.9
Tpx2	3.9
Orc1	3.9
Sectm1a	3.9
Nusap1	3.9
Slfn14	3.9
Fam111a	3.8
Lrrc3	3.8
Arhgef37	3.8
Cryba4	3.8
Sox6	3.8
Cdc20	3.8
Nuf2	3.8
Kif4a	3.8
Ncaph	3.8
Klrc1	3.8
Bpifa6	3.8
Atp1b2	3.8
Epb42	3.7
Ggt1	3.7
RGD1308564	3.7
Parpbp	3.7
Kif22	3.7
Mylk2	3.7
Hdc	3.6
Lyzl1	3.6
Tmem79	3.6
AABR07069282.1	3.6
Prr11	3.6
Cdca2	3.6
Kif18b	3.6
Arg2	3.5
Serping1	3.5
Mt-atp8	3.5
Traip	3.5
Dnase2b	3.5
Car12	3.5
Depdc1b	3.5
Cenpt	3.5
Tmie	3.5
Zbtb32	3.5
Cdca8	3.5
Rab3il1	3.4

Tyms	3.4
Aunip	3.4
Cenpk	3.4
Cenpf	3.4
Spta1	3.4
Epx	3.4
Espl1	3.4
Ska3	3.4
Uhrf1	3.4
Fcer1a	3.4
Car8	3.4
Psat1	3.4
Hemgn	3.4
Troap	3.4
AABR07051675.1	3.3
Timd2	3.3
T	3.3
Gbp2	3.3
Fgf13	3.3
AC128901.1	3.3
AABR07053188.1	3.3
Gtse1	3.3
Mt-nd4l	3.3
Shcbp1	3.3
Melk	3.3
Spag5_2	3.3
Metrn	3.3
NOS2	3.3
Cck	3.3
LOC497963	3.3
Cenph	3.3
Thsd7b	3.3
Ninj2	3.2
LOC100910252	3.2
AABR07073039.1	3.2
Cpa2	3.2
Racgap1	3.2
Trim10	3.2
Bub1	3.2
Spc24	3.1
Ube2t	3.1
Slc22a3	3.1
B3gnt9	3.1
Ncapg	3.1
Spc25	3.1
Fabp5	3.1
AABR07017818.1	3.1

Lim2	3.1
Mis18a	3.1
Sectm1b	3.1
Dtl	3.0
Muc13	3.0
Camp	3.0
Nnmt	3.0
Hbe1	3.0
Palm3	3.0
Sgo2	3.0
Chaf1b	3.0
Myo5c	3.0
Rn60_10_0648.2	3.0
Aurkb	3.0
Ddias	3.0
Il18r1	3.0
Cenpe	3.0
LOC103692984	3.0
Top2a	3.0
S100a3	3.0
Kcnk7	3.0
LOC100359539	2.9
Gmnn	2.9
Atp6ap1l	2.9
Ckap2l	2.9
Stil	2.9
Il12rb1	2.9
Dnaaf1	2.9
Cldn1	2.9
Cks2	2.8
Mfsd2a	2.8
Cep55	2.8
Rn60_20_0141.4	2.8
Asns	2.8
Tk1	2.8
RGD1559662	2.8
Hk3	2.8
Rad51	2.8
Hjurp	2.8
Cenpa	2.8
Kcnk5	2.8
Ndc80	2.8
Kif20b	2.8
Dnah8	2.8
Ect2	2.8
Mcpt8	2.8
Kif23	2.8

Mcm5	2.8
Cks1b	2.8
Cxcr3	2.7
Ttk	2.7
C4b	2.7
Clspn	2.7
LOC100361087	2.7
Fam83d	2.7
Poc1a	2.7
Kif11	2.7
Tinagl1	2.7
Il12rb2	2.7
Csf1	2.7
RGD1310335	2.7
RGD1307461	2.7
Cenpu	2.6
Fignl1	2.6
Pbk	2.6
Dctpp1	2.6
Il18rap	2.6
Tnfrsf17	2.6
LOC303566	2.6
C4a_1	2.6
Serpinb9	2.6
Fam72a	2.6
Fancd2	2.5
AABR07000658.1	2.5
LOC100912604	2.5
Irgm	2.5
Specc1	2.5
Ticrr	2.5
Lgals1	2.5
Klf1	2.5
Bard1	2.5
Adgrg3	2.5
Il18bp	2.5
Rps4y2	2.5
Dmtn	2.5
Cdc45	2.4
Esco2	2.4
Hmmr	2.4
Tmem97	2.4
Car1	2.4
Mcm10	2.4
Igtp	2.4
Arhgap19	2.4
Gpsm2	2.4

Gins2	2.4
Mad2l1	2.4
Ckap2	2.4
Sgo1	2.4
AABR07001512.1	2.4
Aspm	2.4
Rad54l	2.4
Ncapd2	2.4
Foxm1	2.3
Ifi47	2.3
Bub1b	2.3
Creg1	2.3
Mbnl3	2.3
Erbb3	2.3
Ifitm1	2.3
Pdcd1	2.3
RGD1564606	2.3
Cxcr6	2.3
Incenp	2.3
Dut	2.3
Fbxo5	2.3
Kif15	2.3
Cdt1	2.2
Kif18a	2.2
Ly49i2	2.2
Ccdc167	2.2
Gbp5	2.2
Psmb9	2.2
Gpr25	2.2
Gata2	2.2
Pole2	2.2
Phospho1	2.2
Tonsl	2.2
NEWGENE_1306714	2.2
Ptger3	2.1
Rorc	2.1
Svip	2.1
Ucp2	2.1
H2afx	2.1
Sdc1	2.1
Mpst	2.1
Mt-nd6	2.1
Prdm1	2.1
Dhfr	2.1
Sytl3	2.1
Kpna2	2.1
Irf1	2.1

Dscc1	2.0
Dbi	2.0
Ptrhd1	2.0
Psmf1	2.0
Cdkn2c	2.0
Tcf19	2.0
Snrpa	2.0
Dlgap5	2.0
Apol11a	2.0
Amt	2.0
Pmf1	2.0
Mypop	2.0
Rpa3	2.0
Bak1	2.0
Jchain	2.0
Trip13	2.0
Lsm2	2.0
Ctsw	2.0
AABR07016572.1	2.0
Psme2	2.0
Cox7b	2.0
Fen1	2.0
Cdc34	2.0
Map6	1.9
Klri2	1.9
Lrrc75b	1.9
Tigit	1.9
Cst7	1.9
Tap1	1.9
Socs1	1.9
Mcm2	1.9
Gclm	1.9
LOC108351137	1.9
Dhcr7	1.9
Batf	1.9
Rtp4	1.9
C1qtnf6	1.9
Gins3	1.9
Eef1akmt1	1.9
Prc1	1.9
Eomes	1.9
Slirp	1.9
Tubb5	1.9
Entpd2	1.9
Ska2	1.9
Emp1	1.8
Dhcr24	1.8

Pfdn4	1.8
ENSRNOG00000059750	1.8
Klrb1a	1.8
Fam89b	1.8
Mrps11	1.8
LOC687679	1.8
Snrpd1	1.8
Grina	1.8
Slamf1	1.8
Rnf10	1.8
Cat	1.8
Rps27l	1.8
Magohb	1.8
Ifitm2	1.8
Ppih	1.8
Hmgb2l1	1.8
Creld2	1.8
Wars	1.8
RGD1562667	1.8
Ak2	1.8
Lig1	1.8
Tpst2	1.8
Slc2a3	1.8
Aurka	1.8
Isg20	1.8
LOC681292	1.8
Gapdh-ps2	1.8
Uqcrb_2	1.8
Slc25a4	1.8
Sytl2_1	1.8
Ndufa12	1.8
Klra5_3	1.8
Prr5	1.8
Fkbp2	1.8
Gapdh	1.8
Lime1	1.8
LOC100911840	1.8
Il2rb	1.8
Cd8a	1.7
B3gnt7	1.7
Cox5b	1.7
Mcm6	1.7
Plxnd1	1.7
Psmb8	1.7
Usmg5	1.7
Mkrn1	1.7
Sh2d1a	1.7

RT1-Ba	1.7
Elof1	1.7
Ran	1.7
Chn2	1.7
Ubl5	1.7
Psma2	1.7
Cyb561d2	1.7
Txn1	1.7
Tubb4b	1.7
Lmnb1	1.7
Pla2g16	1.7
Naa38	1.7
Hopx	1.7
Prim1	1.7
Fzr1	1.7
MGC105649	1.7
LOC100910979_2	1.7
Acot7	1.7
RT1-Da	1.7
Ripk3	1.7
Rab19	1.7
Znhit1	1.7
Sirt2	1.7
RGD1565641	1.7
Ccnd3	1.7
Rfc3	1.7
Atp5l	1.7
Elob	1.7
Tpi1	1.7
Klrc3	1.7
Polr2a	1.7
Atp5i	1.7
Atp5j2	1.7
Gpr171	1.7
Anxa6	1.7
Sit1	1.7
LOC100362149	1.7
Mt-nd3	1.7
Klrd1	1.7
Psmb10	1.7
Tmem181	1.7
Hmbs	1.7
Ndufb7	1.6
Plp2	1.6
Ube2o	1.6
Rfxap	1.6
Emp3	1.6

Trappc1	1.6
Cox7a2	1.6
Psmb3	1.6
Atpif1	1.6
Slc9a3r1	1.6
Spr	1.6
Anapc13	1.6
Cox6b1_1	1.6
Faslg	1.6
Cox6a1	1.6
Mrps21	1.6
Pole	1.6
Cfl1	1.6
Apol3	1.6
H2afz	1.6
Natd1	1.6
Stat1	1.6
Nudt2	1.6
Psme1	1.6
ENSRNOG00000047185	1.6
Dynlt1	1.6
Mrpl41	1.6
Esd	1.6
Fxyd5	1.6
Slbp	1.6
Sh3bgrl3	1.6
Dad1	1.6
Psmb2	1.6
Snrpc	1.6
Abhd4	1.6
Pcna	1.6
Gnpda1	1.6
Rinl	1.6
Mrpl13	1.6
Eif4e2	1.6
Arsb	1.6
Hcst	1.6
Chaf1a	1.6
Psma5	1.6
Fam26f	1.6
Ap2s1	1.6
Ube2s	1.6
Dpm2	1.6
Rcc1	1.6
Lck	1.6
Nup210	1.6
Cdca4	1.6

Magoh		1.6
Ssr2		1.6
Pigx		1.6
Sema7a		1.6
Napa		1.6
Tmem9		1.6
LOC100911483		1.5
Csnk2b		1.5
Psmb4		1.5
Ppil1		1.5
Ndufb10		1.5
Ddt		1.5
Selenoh		1.5
Ahcy		1.5
Dynlrb1		1.5
Psmb1		1.5
Mcm4		1.5
Lmf2		1.5
Mcm7		1.5
Ap1s1		1.5
Klrb1c		1.5
Ppp1r11		1.5
Rrm1		1.5
Arl6ip1		1.5
Grcc10	2208.0	
Hsp90aa1	1173.4	
LOC108348140	873.8	
Syt6	315.4	
LOC100911625	99.6	
LOC100362298	76.6	
LOC102549115	70.4	
AABR07055805.1	64.3	
Creb3l4	61.7	
Rag1	34.1	
Gucy1b2	28.4	
Defa7	19.5	
Defa5	16.7	
AC118414.1	15.4	
Mgat5b	14.6	
Rag2	14.4	
Fgfbp1	12.2	
Casq1	11.8	
AABR07065772.2	9.8	
Mk1	8.9	
RatNP-3b	8.8	
AABR07012775.1	8.7	
Insrr	7.8	

LOC103692170	7.6
Rn50_10_0890.5	7.3
LOC688684	7.1
Tril	7.1
Lix1	7.1
Tcte1	7.0
AABR07002906.1	6.4
Ptcra	5.8
Ptprn	5.8
LOC100910446	5.6
LOC100911615	5.6
AABR07000398.1	5.0
AABR07017208.2	4.7
Crb3	4.1
Hba-a3	4.0
Olfml3	4.0
AABR07044414.1	3.7
Cyp2d2	3.5
AABR07063424.1	3.5
AABR07015078.1	3.5
AABR07015055.1	3.4
Lmtk3	3.4
AABR07015066.1	3.4
Paqr9	3.3
AABR07015080.2	3.3
Crygs	3.2
Plppr3	3.0
Ewsr1	3.0
Sult1a1	2.9
Trpt1	2.9
Hnf1a	2.7
Pde6h	2.6
Pax5	2.6
AABR07000382.1	2.5
Col26a1	2.3
Haao	2.3
Mapk15	2.3
Thy1	2.2
LOC102550456	2.2
NEWGENE_1304700	2.2
Samd3	2.2
Wbp11	2.2
Pla2g2f	2.1
Prss39	2.1
Jsrp1	2.1
Smpd5	2.1
Insl3	2.1

Mss51	2.1
Vps37d	2.0
LOC100912578	2.0
Nfatc4	2.0
AC141521.1	2.0
Begain	2.0
Dusp11	2.0
Oplah	1.9
Zfp579	1.9
Gem	1.9
LOC100911575	1.9
RT1-DOa	1.9
Rplp2	1.8
LOC501110	1.8
Zfp958	1.8
Cd79a	1.8
AABR07060872.1	1.8
Stard10	1.8
Sap25	1.8
Emid1	1.8
Lax1	1.7
Cacng4	1.7
Rdm1	1.7
Zfp821	1.7
Dtx3	1.7
Flt3lg	1.7
Zfyve19	1.7
Nsmce1	1.7
Rpl34-ps1	1.7
ENSRNOG000000341	1.7
Adamts14	1.7
Neil1	1.7
Tex9	1.7
Cd19	1.7
Deaf1	1.7
Cd72	1.7
Rps10l1	1.7
Dnajc17	1.7
Klkb1	1.7
Bcl7c	1.6
Tmem156	1.6
Siglec10	1.6
Tnfrsf25	1.6
Ptp4a3	1.6
Pym1	1.6
Pxdc1	1.6
Cd79b	1.6

Mier2	1.6	
LOC108348287	1.6	
Tnfrsf14	1.6	
Snrnp25	1.6	
Nt5c	1.6	
Rpl28	1.6	
AC142181.1	1.6	
Snx8	1.6	
Fcrl1	1.6	
Pim2	1.6	
Rps15	1.6	
Rpp21	1.6	
Sstr3	1.6	
NEWGENE_68440	1.6	
Bin1	1.6	
Pla2g6	1.6	
Cyp2s1	1.6	
Fcer2	1.6	
Rpl6-ps1	1.6	
Snrpd2	1.6	
Pced1b	1.6	
AABR07007121.1	1.6	
Kmt5c	1.6	
Tssc4	1.6	
Sigirr	1.6	
Fcrla	1.6	
Mfsd10	1.6	
Tmem206	1.5	
Dnajc1	1.5	
Dapk2	1.5	
Rab24	1.5	
Fcmr	1.5	
Ncr1	1.5	
Grap	1.5	
Ada	1.5	
Fcho1	1.5	
Arid3b	1.5	
Nuak1	1.5	
Nat9	1.5	
Mtg2	1.5	
Bcas2	1.5	
Phkg2	1.5	
Cd27	1.5	
LOC100364500	1.5	
Cxcl9	-2.6	8.2
Cxcl10	-3.5	3.0
Cxcl11	-4.7	6.9

Dntt	3.4	-2.6
Pnpla8	-1.5	-1.6
Slc28a2	-1.5	-1.7
Naaa	-1.5	-1.9
Cnot6l	-1.6	-1.6
P2ry10	-1.6	-1.7
Map1lc3a	-1.6	-1.6
Rell1	-1.6	-1.6
Ranbp9	-1.6	-1.6
Lilrb4	-1.6	-1.9
Fnip1	-1.6	-1.7
Arsa	-1.7	-2.0
Csgalnact2	-1.7	-1.6
Lmo2	-1.7	-1.8
Secisbp2l	-1.7	-1.7
Slfn4	-1.7	-1.8
Atp1a1	-1.7	-1.6
Rps6ka2	-1.7	-1.7
Dusp5	-1.7	-2.5
Spg20	-1.8	-1.7
Msl2	-1.8	-1.7
Ctsl	-1.8	-2.0
Rhoq	-1.8	-1.8
Itga6	-1.8	-2.2
Mfsd6	-1.8	-1.9
Tcf7l2	-1.8	-2.0
Dram1	-1.8	-1.8
Dhx58	-1.8	-1.6
Serpinb6	-1.9	-2.8
Mras	-1.9	-2.1
Agl	-1.9	-2.3
Cnksr3	-1.9	-2.0
Asph	-1.9	-1.9
Nr4a1	-1.9	-2.4
Flnb	-1.9	-3.8
Amz1	-1.9	-2.3
Metrnl	-1.9	-1.7
March7	-1.9	-1.9
Phf20l1_1	-1.9	-2.0
Bhlhe40	-1.9	-1.8
Lgmn	-1.9	-1.9
Herc6	-1.9	-1.8
Dusp3	-1.9	-1.7
Cd86	-1.9	-2.0
Dpy19l1	-2.0	-1.6
Cd4	-2.0	-1.9
Lipa	-2.0	-1.7

Ctnna1	-2.0	-1.7
Nfkbiz	-2.0	-3.3
Tnfaip3	-2.0	-2.1
Krt75	-2.0	-2.0
Ppp1cb	-2.0	-1.6
Cebpb	-2.0	-2.0
Fcho2	-2.0	-1.8
RGD1304694	-2.0	-1.7
Tal1	-2.0	-2.1
Snx18	-2.1	-1.8
B4galt6	-2.1	-2.7
Capn3	-2.1	-7.1
Mx2	-2.1	-2.6
Sh3bp4	-2.1	-2.1
Plp1	-2.1	-3.0
Timp2	-2.1	-2.1
Cebpe	-2.1	-2.0
Dse	-2.1	-2.1
Laptm4b	-2.2	-2.9
Tmcc3	-2.2	-3.4
Selenop	-2.2	-2.2
Ccrl2	-2.2	-2.2
Prodh1	-2.2	-2.0
Tubb2a	-2.3	-1.9
Gcnt2	-2.3	-2.2
Dapk1	-2.3	-2.6
G0s2	-2.4	-2.8
Pard3	-2.4	-2.7
Ccl9	-2.4	-2.6
Slc27a3	-2.4	-2.5
LOC681341	-2.4	-3.6
Fpr3	-2.5	-3.0
Fgd4	-2.5	-2.3
AABR07036301.1	-2.5	-7.1
Dennd2c	-2.5	-2.4
Pon1	-2.5	-2.5
Klf6	-2.5	-2.0
Gzmbl3	-2.5	-2.7
Zcchc24	-2.5	-2.0
Abcg2	-2.5	-2.4
Fcna	-2.5	-2.6
Klf4	-2.5	-2.7
Acvr1	-2.5	-2.9
Fos	-2.6	-2.3
Mrc2	-2.6	-4.8
Lpl	-2.6	-4.4
Adgrg6	-2.6	-3.0

Serpinb11	-2.6	-2.5
Tspan12	-2.6	-3.0
Lpar1	-2.6	-3.4
Gprc5d	-2.6	-2.4
Nrp1	-2.6	-2.5
Spred1	-2.7	-2.6
Serpinb8	-2.7	-2.6
RGD1563354	-2.7	-3.3
Pon3	-2.8	-3.0
Map1a	-2.8	-3.9
RGD1560281	-2.8	-2.5
Gucy1b3	-2.8	-2.5
Dusp6	-2.8	-2.2
Pparg	-2.8	-4.5
Gjb2	-2.8	-3.7
Adgre1	-2.8	-2.6
Magi1	-2.9	-3.9
Zfp503	-2.9	-3.4
LOC103692471	-2.9	-5.8
Hes1	-2.9	-7.6
Jag1	-2.9	-3.0
Ldlrad3	-3.0	-3.4
F13a1	-3.0	-2.6
Mylk	-3.0	-2.7
Rfx2	-3.0	-3.1
Maob	-3.0	-4.3
Tek	-3.0	-3.2
Arhgef10l	-3.1	-2.1
Mafb	-3.1	-3.4
Nrxn2	-3.1	-8.7
Gpr137b	-3.1	-2.7
Npl	-3.1	-2.7
Tbc1d9	-3.1	-2.7
Qrfpr	-3.1	-5.4
Rnf149	-3.1	-2.2
Morc4	-3.1	-2.9
RGD1561662	-3.2	-4.0
Lamb3	-3.2	-6.7
Fam184a	-3.2	-2.5
Gp6	-3.2	-2.5
Palld_2	-3.3	-4.1
Adam15	-3.3	-2.1
Cadm3	-3.4	-2.5
Ascl2	-3.4	-2.8
Kcnab1	-3.4	-2.5
Fam198b	-3.4	-2.9
Akr1b10	-3.4	-3.2

Pid1	-3.4	-2.6
Vldlr	-3.5	-2.8
RGD1311575	-3.5	-2.7
Tgfa	-3.6	-3.1
Crim1	-3.6	-2.9
Pir	-3.7	-3.0
Fpr2	-3.7	-2.6
Fam46a	-3.8	-2.8
Npy	-3.8	-4.6
Cecr6	-3.8	-5.2
Tubb1	-3.8	-2.3
Thbd	-3.9	-3.9
Angpt1	-3.9	-3.3
Cfh	-3.9	-2.9
Tgfb3	-3.9	-4.9
LOC100910801	-3.9	-4.3
NEWGENE_2319083	-4.0	-3.0
Kdr	-4.0	-3.0
Zfyve9	-4.1	-2.6
Zfp111	-4.3	-8.4
AABR07035650.1	-4.4	-3.9
Adgra2	-4.5	-3.2
Cd36	-4.6	-4.2
Alpk2	-4.7	-7.9
Gapt	-4.8	-3.1
Oas2	-4.8	-3.6
Fabp4	-5.0	-6.1
AABR07021717.1	-5.1	-4.1
Rps6_1	-10.4	-63.2
Timp3	-10.5	-4.1
RGD1563818	-17.1	-13.1
LOC100912498	-17.6	-24.6
Fam71a	-19.9	-21.5
Polr2l	-348.1	-313.8
Rpl35al1	-358.1	-34.4
Lemd3		-1.5
LOC100912163		-1.5
Etnk1		-1.5
Pcf11		-1.5
Cnnm4		-1.5
Dync1li2		-1.5
Pank3		-1.5
Zfx		-1.6
Rbm26		-1.6
Ash1l		-1.6
Cr2		-1.6
Luc7l3		-1.6

Ubqln2	-1.6
Fam43a	-1.6
Slc30a9	-1.6
Cyb5r1	-1.6
Parg	-1.6
Rnf103	-1.6
Abcc5	-1.6
Ttc3	-1.6
Scarb2	-1.6
L3mbtl3	-1.6
Lrig1	-1.6
Pikfyve	-1.6
ENSRNOG00000051592	-1.6
Zc3h11a	-1.6
Irf6	-1.6
Sik2	-1.6
Acer3	-1.6
Smad4	-1.6
Rps19	-1.6
Gxylt1	-1.6
Map3k8	-1.6
Mllt10	-1.7
Inpp5f	-1.7
Ubp1	-1.7
Ddx17	-1.7
Ulk2	-1.7
Usp33	-1.7
Ccr9	-1.7
Nr1d2	-1.7
Rsbn1	-1.7
Gigyf1	-1.7
Pnisr	-1.7
Heca	-1.7
Zfp869	-1.7
Camk2d	-1.7
Prkag2	-1.7
Prpf39	-1.7
Ralgps2	-1.7
Chd7	-1.7
Wdfy2	-1.7
Rhoc	-1.7
Ggta1	-1.7
Cdkn1b	-1.7
Ano8	-1.7
Pcmtd2	-1.7
Ints6l	-1.7
Prpf4b	-1.7

Zbtb44	-1.7
Smarca2	-1.7
Taf1	-1.8
Setx	-1.8
Dzf17	-1.8
Ogt	-1.8
Zscan26	-1.8
Ceacam1	-1.8
Adcy6	-1.8
Mllt6	-1.8
Pde3b	-1.8
Nedd4	-1.8
Rgs2	-1.8
Dnajb9	-1.8
L1cam	-1.8
Sgms1	-1.8
Zfp238	-1.8
Ppm1h	-1.9
Dstyk	-1.9
Zfc3h1	-1.9
Crebzf	-1.9
Eno3	-1.9
Frat2	-1.9
Ldlrap1	-1.9
Epm2aip1	-1.9
Mef2b	-1.9
Hs3st3b1	-1.9
AABR07058985.2	-1.9
Mtss1	-1.9
Mpp5	-1.9
Klf9	-1.9
Ankrd33b	-1.9
F8	-1.9
Itgb5	-2.0
Myb	-2.0
Mgat4a	-2.0
Fyttd1	-2.0
Fmr1	-2.0
Efnb2	-2.0
LOC100911851	-2.0
Ago4	-2.0
Col17a1	-2.0
Ier5l	-2.0
RGD1309995	-2.0
Tob1	-2.0
Slc25a36	-2.1
Wdr13	-2.1

Thrb	-2.1
She	-2.1
Etv5	-2.1
Ccdc142	-2.2
Ptprs	-2.2
Gucy1a3	-2.2
Kbtbd11	-2.2
Kif13a	-2.2
F11r	-2.2
Skil	-2.2
LOC100911319	-2.3
Il6st	-2.3
Rhob	-2.3
Ahr	-2.3
LOC102550455	-2.3
AABR07012475.2	-2.3
Porcn	-2.3
Sspn	-2.4
RGD1565989	-2.4
Pros1	-2.4
Epha2	-2.4
Srcin1	-2.4
AC127076.2	-2.5
Rhbdf1	-2.5
Kifc3	-2.5
Fam8a1	-2.5
Otud1	-2.5
AABR07035273.1	-2.5
AABR07065448.1	-2.5
Carmil1	-2.6
Glis2	-2.6
Cd163l1	-2.6
Rn50_10_0892.1	-2.6
Atxn7	-2.7
Mcf2l	-2.7
Sik1	-2.7
Sdk2	-2.7
Acer2	-2.7
Zdhhc1	-2.7
Eng	-2.7
Tppp3	-2.8
Cmtm4	-2.8
LOC108348155	-2.8
Ptk2	-2.8
RGD1310081	-2.9
Clec12b	-2.9
Ngfr	-2.9

Popdc2	-2.9
RGD1561149	-2.9
Ecm1	-2.9
Cfb_1	-3.0
Coro6	-3.0
Acadsb	-3.1
Prrt2	-3.1
Hsf4	-3.2
Thbs3	-3.2
Sema6d	-3.2
Ca5b	-3.2
B3gnt3	-3.3
Fst	-3.3
LOC691695	-3.3
Adgra3	-3.3
Myo1b	-3.3
Ier3	-3.3
Fosb	-3.3
LOC100912034	-3.3
Syt15	-3.4
Rn50_X_0752.3	-3.4
Rgl1	-3.5
Cav1	-3.5
Tubb6	-3.5
Irs1	-3.6
Gdf3	-3.6
Snta1	-3.6
Fat1	-3.6
Scn1b	-3.6
Tppp	-3.7
Wwc2	-3.7
RGD1566138	-3.7
Bhlhe22	-3.7
Il5ra	-3.8
AABR07065656.7	-3.8
Tspan18	-3.8
Pcsk1	-3.8
Psca	-3.9
Tpbg	-3.9
Cd109	-3.9
AABR07035955.1	-4.0
Sash1	-4.0
Fam199x	-4.1
Spock2	-4.1
P4ha2	-4.1
Zbtb16	-4.1
Etv1	-4.1

Ccnd1	-4.1
Fam57a	-4.2
Ephb4	-4.2
Dsp	-4.3
Heph	-4.3
Selp	-4.3
Gas6	-4.3
AABR07072639.2	-4.4
Bmp2	-4.4
Igfbp6	-4.4
Adamts12	-4.4
RGD1310819	-4.5
Stab1	-4.5
Gprc5a	-4.6
Slc9a3r2	-4.6
AABR07013729.1	-4.6
Cndp1	-4.7
Adgrb2	-4.7
Folr2	-4.7
Pltp	-4.7
Pld6	-4.8
Gjc1	-4.8
AABR07051326.1	-4.9
Caskin1	-4.9
Pmp22	-4.9
Nov	-4.9
Ltbp1	-5.0
Tgfb2	-5.1
LOC100912210	-5.1
Ltc4s	-5.2
Semg1	-5.3
Msr1	-5.4
LOC100360573	-5.6
Olfml2a	-5.6
AABR07062068.1	-5.8
Tcam1	-5.8
Lhfp	-5.8
Fscn1	-5.9
Arhgap32	-5.9
Pdlim4	-5.9
Fgfr1	-6.0
Vil1	-6.1
LOC688442	-6.1
Frk	-6.1
Fam3b	-6.1
Lyve1	-6.2
Ptrf	-6.2

Enho		-6.3
Mlxipl		-6.4
Plk2		-6.4
Dock3		-6.4
Cyp26a1		-6.8
Arhgef5		-6.9
Asgr2		-6.9
Ntm		-6.9
Sgpp2		-6.9
Axl		-7.0
Prr16		-7.2
Ms4a8		-7.2
Shank3		-7.3
Wnt4		-7.3
Defb14		-7.5
Ltbp3		-7.8
Slc2a10		-8.0
Soat2		-8.3
Sspo		-8.6
Myo5b		-8.6
Ccl2		-9.2
Cd163		-9.4
Lif		-9.6
Slc36a2		-10.1
RGD1566368		-10.2
Mamdc2		-11.0
Cd300lg		-11.3
Mmp2		-12.3
Prox1		-13.5
Ccl7		-14.8
Gpr39		-14.9
Lipm		-21.6
Duoxa1		-22.9
LOC100911951		-55.9
LOC100910163		-72.0
LOC103694380		-73.7
Sccpdh		-268.4
Oas1i		-332.6
Spi1	-1.5	
Fam168b	-1.5	
Ehd4	-1.5	
Vps35	-1.5	
Stx3	-1.5	
Ctnnb1	-1.5	
Nptn	-1.5	
Canx	-1.5	
Sppl2a	-1.5	

Ap2a2	-1.5
Lta4h	-1.5
Cmpk1	-1.5
Myo1f	-1.5
Prdx5	-1.5
Rab31	-1.5
Sdcbp	-1.5
Rpe	-1.5
Mapk1	-1.6
Tkt	-1.6
Naga	-1.6
Cbfb	-1.6
Cndp2	-1.6
LOC108348078	-1.6
Rnf13	-1.6
Fgl2	-1.6
Hps3	-1.6
Abi1	-1.6
AABR07011951.1	-1.6
Lrrc8d	-1.6
Rock1	-1.6
Rrbp1	-1.6
Mgat2	-1.6
Cd44	-1.6
Slc16a3	-1.6
Cxcl16	-1.6
Ptpre	-1.6
Calhm2	-1.6
Gngt2	-1.6
Eif2ak2	-1.6
Zfp385a	-1.6
Lyn	-1.6
Erlin1	-1.6
Nampt	-1.6
Brcc3	-1.6
Apoe	-1.6
Ssfa2	-1.6
Glrx	-1.6
Hk2	-1.6
Apaf1	-1.6
Tmx1	-1.6
Vps41	-1.6
Fam49a	-1.6
Amfr	-1.6
Fcgr2b	-1.6
Plbd1	-1.6
Ube2d3	-1.6

Ckb	-1.6
Rarg	-1.6
Net1	-1.6
Rps6ka3	-1.6
Itgb1	-1.6
Far1	-1.6
Napsa	-1.6
Baz1a	-1.6
Tgfbr1	-1.6
Reep3	-1.6
Chst12	-1.6
Plekhb2	-1.6
Bnip2	-1.6
Ern1	-1.6
Wapl	-1.6
Hspa13	-1.6
Mgrn1	-1.6
Atp13a2	-1.7
Ipo7	-1.7
Cklf	-1.7
Srgap2	-1.7
Bcat1	-1.7
Chi3l1	-1.7
Tspan2	-1.7
Rhov	-1.7
Ptpn12	-1.7
Nipa2	-1.7
Ddx3x	-1.7
Rap1b	-1.7
Prcp	-1.7
Eif4e3	-1.7
Hif1a	-1.7
RGD1561143	-1.7
Atp6v1a	-1.7
Vim	-1.7
Unc93b1	-1.7
Arid3a	-1.7
Pi16	-1.7
Ly49si1	-1.7
Skap2	-1.7
Pik3ap1	-1.7
Mapkapk3	-1.7
Ndufaf7	-1.7
Fgr	-1.7
Stom	-1.7
Cyld	-1.7
Slc2a6	-1.7

Aif1	-1.7
Icam1	-1.7
Rap2a	-1.7
Ctsf	-1.7
Ptp4a1	-1.7
Ccl6	-1.7
RGD1309079	-1.7
Atp6ap2	-1.7
Ctsz	-1.7
Nceh1	-1.7
Aplp2	-1.7
Purb	-1.7
Sod2	-1.7
Lbp	-1.7
Agpat4	-1.7
Atp2a2	-1.7
Zbtb7b	-1.7
Ralb	-1.7
Nadk	-1.7
Tob2	-1.7
Pten	-1.7
Clip2	-1.7
Gm2a	-1.7
Cd84	-1.7
Serinc3	-1.7
Pstpip2	-1.8
Ogfrl1	-1.8
Plcb3	-1.8
Cd1d1	-1.8
Gchfr	-1.8
Nfe2l1	-1.8
B4galt5	-1.8
Rtn4	-1.8
Nbn	-1.8
Ccr5	-1.8
LOC680910	-1.8
Slc15a3	-1.8
Pmaip1	-1.8
Pla2g7	-1.8
Tnfrsf1a	-1.8
Plaur	-1.8
Cd244	-1.8
Themis2	-1.8
Pgap2	-1.8
Cln8	-1.8
Tpd52	-1.8
Tm9sf3	-1.8

Bst2	-1.8
Ifih1	-1.8
Fes	-1.8
Mfsd1	-1.8
Pik3c2a	-1.8
Lrpap1	-1.8
Grn	-1.8
Stard4	-1.8
Hlx	-1.8
Exo5	-1.8
Scamp1	-1.8
Cd59	-1.8
Nucb2	-1.8
Pkm	-1.8
Slc35e3	-1.8
Anxa1	-1.8
Tp53inp2	-1.8
Ap3s1	-1.8
Arhgap18	-1.8
Ostm1	-1.8
Tifa	-1.8
Rbpj_1	-1.8
Id2	-1.8
Fcgr1a	-1.8
Nfe2l2	-1.8
Capn2	-1.8
Serinc1	-1.8
Oat	-1.8
Rnf144b	-1.8
Mfsd14b	-1.8
Alox5ap	-1.9
Sgpp1	-1.9
Idh1	-1.9
Mdfic	-1.9
Klhl9	-1.9
Il1rn	-1.9
Smagp	-1.9
Slc25a16	-1.9
Tspan31	-1.9
Il10ra	-1.9
Ncf2	-1.9
Map3k9	-1.9
Crk	-1.9
Klra2	-1.9
Nfe2	-1.9
Tmem30a	-1.9
Inf2	-1.9

Tcn2	-1.9
Irf5	-1.9
Tsc22d1	-1.9
Ctbs	-1.9
Xbp1	-1.9
Sort1	-1.9
Tbc1d2	-1.9
Naip6	-1.9
Parp12	-1.9
Hmox1	-1.9
Cybrd1	-1.9
Nfil3	-1.9
Grk5	-1.9
Mef2a	-1.9
Cpne2	-1.9
Blvra	-1.9
Pigm	-1.9
Suz12	-1.9
Sipa1l2	-1.9
Lpcat2	-1.9
Plekhg3	-1.9
Sirpa	-1.9
Ptprj	-1.9
Zcchc6	-1.9
Atp2b1	-1.9
Nlrp3	-1.9
Calcrl	-1.9
S100a6	-1.9
Tmem50b	-1.9
Gng10	-2.0
Ppp1r3d	-2.0
XAF1	-2.0
Nod2	-2.0
Slc11a1	-2.0
Itga5	-2.0
Fam120a	-2.0
Nqo1	-2.0
Cast	-2.0
Camkk2	-2.0
C1galt1	-2.0
Tlr2	-2.0
Oaf	-2.0
Tns3	-2.0
Slc37a2	-2.0
Tmem176a	-2.0
Tlr3	-2.0
Cd226	-2.0

Tnfaip2	-2.0
Tgm2	-2.0
LOC108348047	-2.0
Trem3	-2.0
Mgst1	-2.0
Cfp	-2.0
Actr2	-2.0
Serpinb1a	-2.0
Mocs1	-2.0
Atp1a3	-2.0
Ptafr	-2.0
LOC100911825	-2.0
Ets2	-2.0
Tmed7	-2.1
Snx10	-2.1
Mafg	-2.1
Nfam1	-2.1
C5ar1	-2.1
Lrp1	-2.1
Ggh	-2.1
Cdc42bpb	-2.1
Kpna4	-2.1
Marcks	-2.1
Arhgap22	-2.1
Ndrg1	-2.1
S100a4	-2.1
Adssl1	-2.1
Gsr	-2.1
Pqlc3	-2.1
Cybb	-2.1
Nlrx1	-2.1
Slc25a24	-2.1
Siglec8	-2.1
Kif1b	-2.1
Zfp703	-2.1
Isg15	-2.1
Sgk3	-2.1
Tmx3	-2.1
Tfe3	-2.1
Rp2	-2.1
Aldh3b1	-2.1
Ldlr	-2.1
Sox13	-2.1
LOC102546864	-2.1
Spns3	-2.1
Ccr1	-2.1
Lima1	-2.1

Klf10	-2.1
Usp18	-2.1
Tnfrsf21	-2.1
Zbtb42	-2.1
Bmx	-2.1
Sgcb	-2.2
Mfsd12	-2.2
Arl11	-2.2
Acap2	-2.2
Sqrdl	-2.2
Lrrc25	-2.2
Atp2c1	-2.2
AABR07006142.1	-2.2
Cx3cr1	-2.2
Cd300lb	-2.2
Myof	-2.2
Ninj1	-2.2
Tlr10	-2.2
Asah1	-2.2
Dpysl2	-2.2
Ticam2	-2.2
Hip1	-2.2
Lpgat1	-2.2
Adipor2	-2.2
LOC681325	-2.2
RGD1563091	-2.2
Hck	-2.2
Mtus1	-2.2
Oasl	-2.2
Rnd3	-2.2
Filip1l	-2.2
LOC685157	-2.2
C3	-2.2
Clec4d	-2.2
Anxa5	-2.2
Scarb1	-2.2
Tiam1	-2.2
Gyg1	-2.2
Ptprm	-2.2
Nilr1	-2.3
Csf2ra	-2.3
Plin2	-2.3
Rassf4	-2.3
Trim13	-2.3
P4ha1	-2.3
Lamp2	-2.3
Hsd11b1	-2.3

Lgals3	-2.3
Pfkfb4	-2.3
rnf141	-2.3
Ltbr	-2.3
Tlr4	-2.3
Samsn1	-2.3
Hp	-2.3
Dnajc16	-2.3
Atp11a	-2.3
Lrrc49	-2.3
Padi2	-2.3
Slc44a1	-2.3
Lilrb3a	-2.3
Psap	-2.3
C1qtnf1	-2.3
Tnfrsf8	-2.3
LOC100910669	-2.4
Tbc1d8	-2.4
Igsf6	-2.4
Ptgir	-2.4
Havcr2	-2.4
Mefv	-2.4
Sbf2	-2.4
Zfp710	-2.4
Anpep	-2.4
Cpped1	-2.4
AABR07035722.1	-2.4
Fxyd2	-2.4
Gpnmb	-2.4
Csf1r	-2.4
Anxa4	-2.4
Oas3	-2.4
RGD1561730	-2.4
Arpin	-2.4
Pld4	-2.4
Cd302	-2.4
Arrb1	-2.4
Retnlg	-2.4
Hcar2	-2.4
Tmem37	-2.4
NEWGENE_2724	-2.5
Slc40a1_2	-2.5
Fam129b	-2.5
Lacc1	-2.5
Pak1	-2.5
Ucp3	-2.5
Rusc2	-2.5

F5	-2.5
Irf7	-2.5
Ifit3	-2.5
Clec7a	-2.5
Prkd1	-2.5
Tnfrsf1b	-2.5
Smpdl3b	-2.5
Abcc3	-2.5
Large1	-2.5
Cd68	-2.5
Gramd3	-2.5
Cd200r1	-2.5
Clec12a	-2.5
Card9	-2.5
Plxdc2	-2.5
Maf	-2.5
Plxnb2	-2.6
Tmtc3	-2.6
Lyc2	-2.6
Fhl1	-2.6
Ctgf	-2.6
Nlrc4	-2.6
Nr1h3	-2.6
Xdh	-2.6
Plvap	-2.6
Mboat1	-2.6
Catip	-2.6
Tmem176b	-2.6
Tf	-2.6
Enkur	-2.6
Lmna	-2.6
Sh2d1b	-2.6
St14	-2.6
Alox12	-2.6
Slc6a6	-2.6
Cdkn1a	-2.6
Ctdspl	-2.6
Frrs1	-2.6
Nostrin	-2.6
Rasgrp4	-2.6
Cdc42ep4	-2.6
Dok4	-2.7
Numbl	-2.7
Cd80	-2.7
Clec9a	-2.7
Cpne3	-2.7
Gas2l1	-2.7

Sulf2	-2.7
Ptpro	-2.7
Olr1	-2.7
Tlr13	-2.7
Rab32	-2.7
Csf2rb	-2.7
Tbxas1	-2.7
Il36b	-2.7
Tmem65	-2.7
Me1	-2.7
Cd300a	-2.7
Clec4a1	-2.7
Fn1	-2.7
Inpp4a	-2.7
Slk	-2.7
Clec4b2	-2.7
Pwwp2b	-2.7
Samhd1	-2.7
Ap1s2	-2.7
Lilrc2	-2.8
Nf1x	-2.8
Papss2	-2.8
Alcam	-2.8
Npr1	-2.8
Smpdl3a	-2.8
Tifab	-2.8
Ypel2	-2.8
AABR07030086.2	-2.8
AABR07006311.1	-2.8
Ly49si2	-2.8
Cd300le	-2.8
Hgf	-2.8
Tnfsf13	-2.8
Gda	-2.8
Trem1	-2.8
Cebpa	-2.8
Gramd1c	-2.8
Dock1	-2.8
Atf3	-2.8
Pcyox1	-2.8
Mmrn1	-2.8
Sgms2	-2.8
Anxa3	-2.8
Il13ra1	-2.8
Zc2hc1a	-2.8
Slc25a13	-2.9
Gp1ba	-2.9

Pla2g4a	-2.9
Cyp2d1	-2.9
Lilrb3	-2.9
Slc36a3	-2.9
Clca2	-2.9
Olfm1	-2.9
Slc7a8	-2.9
Asrgl1	-2.9
Gpr157	-2.9
Atrnl1	-2.9
Adam9	-2.9
Mpl	-3.0
Ly49si3	-3.0
Stard8	-3.0
Ms4a6a	-3.0
Ugt1a5	-3.0
Tfec	-3.0
Marveld1	-3.0
Gpt2	-3.0
Rpl5l1	-3.0
LOC685933	-3.0
Maoa	-3.0
Tlr11	-3.0
Tlr12	-3.0
Serpinb10	-3.1
Galntl5	-3.1
P2ry13	-3.1
Rgs18	-3.1
Smad9	-3.1
Plxna3	-3.1
Clec4a2	-3.1
Lilra5	-3.1
LOC102556085	-3.1
Kcnk13	-3.1
Car13	-3.1
Gatm	-3.1
Slc24a3	-3.1
Kank1	-3.1
RGD1566226	-3.2
Smim5	-3.2
Megf9	-3.2
AABR07019083.1	-3.2
Rab11fip5	-3.2
Cttnbp2nl	-3.2
LOC100360575_1	-3.2
G6b	-3.2
Myct1	-3.2

Rtp3	-3.2
Pld1	-3.2
Kcna3	-3.2
Snx24	-3.2
Rxra	-3.3
Rin2	-3.3
Lyz2	-3.3
Ptgr1	-3.3
Crybg3	-3.3
C1r	-3.3
LOC680254	-3.3
Tm6sf1	-3.3
Fam20c	-3.3
Slc18a2	-3.3
Aoah	-3.3
Flt3	-3.3
Dab2	-3.3
RGD1304884	-3.3
Rab13	-3.3
Rbm47	-3.3
Rab7b	-3.4
Angptl4	-3.4
Trpc6	-3.4
Cers6	-3.4
Adamts2	-3.4
Slamf8	-3.4
LOC685048	-3.4
Soat1	-3.4
Gas7	-3.4
AABR07021221.1	-3.4
Lrp4	-3.4
Zbtb46	-3.4
LOC24906	-3.5
Cdc42bpa	-3.5
Acvrl1	-3.5
Abcd2	-3.5
Efna2	-3.5
Tac4	-3.5
Gpr27	-3.6
Tlr7	-3.6
ENSRNOG000000517	-3.6
Tnfaip8l3	-3.6
Clec4a3	-3.6
RGD1559482	-3.6
Cyfip1	-3.6
Mal	-3.6
Dmxl2	-3.6

AABR07058124.4	-3.6
AABR07030773.1	-3.6
LOC100909879	-3.7
RGD1561778	-3.7
Slc8a1	-3.7
Fndc3b	-3.7
Cxxc4	-3.7
Apopt1_2	-3.7
Klhl41	-3.7
Ctbp2	-3.7
Trim55	-3.7
Papln	-3.7
Chst13	-3.8
Tlr8	-3.8
Cd34	-3.8
Itgb3	-3.8
Fbln1	-3.8
AABR07002001.1	-3.8
Rnf128	-3.8
Acvr2b	-3.8
AABR07062183.1	-3.8
Clec10a	-3.8
Cadm1	-3.8
Ankdd1a	-3.8
Dfna5	-3.8
Dsg3	-3.8
Rab38	-3.9
NEWGENE_1559832	-3.9
Grk3	-3.9
Fam84b	-3.9
Ass1	-3.9
Aox3	-3.9
Slamf9	-3.9
Wtip	-4.0
Emilin2	-4.0
Liph	-4.1
AABR07060995.1	-4.1
Peli2	-4.1
Mest	-4.1
Ccr2	-4.1
LOC100360940	-4.1
Chrdl1	-4.1
Mctp1	-4.1
Ifit2	-4.1
LOC691418	-4.2
Ndst1	-4.2
Spic	-4.2

Ccdc88a	-4.2
Vash1	-4.2
AABR07053483.1	-4.2
Cables1	-4.3
P2ry12	-4.3
Sept10	-4.3
Gab1	-4.3
Afdn	-4.4
Kcns3	-4.4
AABR07024972.1	-4.4
Lilrb3l	-4.4
AABR07010563.1	-4.5
P2ry2	-4.5
Colec12	-4.5
Ak4	-4.5
Siglech	-4.5
Mnda	-4.5
Adgre4	-4.6
Cd14	-4.6
Mrc1	-4.6
Tgfbi	-4.7
Gpr141	-4.7
Trib1	-4.8
Filip1	-4.9
Rn60_20_0069.2	-4.9
Arhgap28	-4.9
LOC679149	-4.9
Mitf	-4.9
Spata18	-4.9
RGD1311756	-5.0
Gpr34	-5.1
Nipa1	-5.1
AABR07027866.1	-5.2
Angptl2	-5.2
Smpd3	-5.2
Dixdc1	-5.2
Trim36	-5.3
Sowahc	-5.3
Lonrf3	-5.3
P2ry1	-5.4
Hrh3	-5.5
Edil3	-5.5
Fam135a	-5.5
Cds1	-5.6
Vsig2	-5.6
Srxn1	-5.7
Lipg	-5.7

Alox12e	-5.7
Ccl22	-5.7
Mmp19	-5.9
Asic1	-5.9
Strip2	-6.0
Thbs1	-6.0
AABR07006310.1	-6.3
Trim16	-6.3
Lhfpl2	-6.3
Cd300e	-6.3
Adap2	-6.5
Myo7a	-6.5
Sec14l5	-6.6
Bean1	-6.8
AABR07031489.1	-6.9
Ak7	-6.9
Pigz_2	-6.9
LOC681355	-7.0
Sstr4	-7.2
Apba1	-7.5
Clec4a	-7.5
Fcnb	-7.6
Lefty2	-7.6
Gprc5c	-7.8
Igf1	-8.0
Plcb1	-8.0
Rn50_4_1360.2	-8.4
F10	-8.6
Prrg1	-8.9
Tmtc2	-9.0
Pde3a	-9.1
Pde5a	-9.2
Rnase2	-9.3
Kcne3	-9.3
Vcan	-9.4
Egfr	-9.5
Fndc7	-9.7
AABR07006278.1	-9.8
Kcng2	-10.2
Sema3c	-10.6
Siglec1	-11.6
Hs3st3a1	-11.6
Dsc1	-13.0
AABR07030091.1	-13.8
Pik3c2g	-15.3
Sh3pxd2b	-17.3
Pole3	-17.9

Dact1	-19.6
Faim_1	-186.5

RNAseq gene expression. Day 10
Fold change: < 1.5 or >1.5
FDR p-value: < 0.05

	LVS / PBS	ΔclpB / PBS
	Day 10	Day 10
Spink2	166.0	654.8
Camk1g	78.9	215.7
Grcc10	169.0	194.4
Gzmc	118.7	31.7
Xirp1	19.8	22.4
Il34	6.7	14.5
Msc	7.8	10.7
Pianp	6.1	9.4
Dclk1	5.0	9.2
Arl14epl	3.5	7.5
Acod1	2.7	6.9
LOC100910138	5.9	6.9
AABR07029202.1	4.6	6.4
Gng3	5.5	6.0
AABR07058511.1	4.6	4.8
Nova2	3.2	4.8
Ly49s6	3.8	4.3
Heatr9	3.4	4.3
Nkpd1	3.7	4.2
Ly49s4	3.6	4.0
Ccl5	2.3	3.5
AABR07044414.1	3.4	3.4
Ltbp4	2.1	3.3
Tbx21	2.8	3.3
RGD1307461	2.1	3.2
Klrg1	2.3	3.1
Klra1	3.2	3.0
Atcay	2.5	3.0
Gzmm	2.2	2.9
Il12rb1	2.1	2.9
Sptbn4	2.8	2.9
Exoc3l2	2.5	2.9
C4a_1	2.4	2.8
Tnni3k	2.4	2.8
Nkg7	2.2	2.7
Emp1	1.8	2.7
Kcnk5	2.1	2.7
Prdm1	2.0	2.6
Il18r1	1.8	2.6
Ramp2	2.9	2.5
Hnf1a	2.0	2.5

	Up-regulated genes	
	Count	
	LVS	ΔclpB
Both vaccines	84	84
LVS only	41	
ΔclpB only		124
Total Up-regulated	125	208

	Down-regulated genes	
	Count	
	LVS	ΔclpB
Both vaccines	240	240
LVS only	81	
ΔclpB only		924
Total down-regulated	321	1164
Total genes	446	1372

Klrc3	2.3	2.4
Tnfsf9	2.1	2.4
Sult1a1	2.7	2.4
Gzmk	1.9	2.4
Il18rap	1.8	2.4
Cdhr1	1.7	2.3
LOC691995	2.3	2.3
Samd3	2.2	2.2
Faslg	1.6	2.2
Csf1	1.8	2.2
Cd2	1.8	2.2
Klra5_4	2.5	2.2
Cacna1a	2.0	2.1
Sytl3	1.7	2.1
Klre1	1.8	2.1
Ly49i5	2.2	2.1
LOC681292	1.6	2.1
Sema7a	1.6	2.1
AABR07015078.1	1.7	2.1
Pla2g2f	2.1	2.1
AABR07063424.1	1.7	2.0
AABR07015066.1	1.7	2.0
Cpne7	1.8	2.0
AABR07015055.1	1.7	2.0
Lag3	1.8	2.0
Gfi1	1.7	2.0
Gpr171	1.7	2.0
Gem	1.8	2.0
Sulf1	1.7	1.9
Pcgf2	2.0	1.8
Mt-nd5	1.8	1.8
Sh2d2a	1.7	1.8
Klra5_3	1.8	1.8
Ncr1	1.7	1.8
B3gnt7	1.6	1.8
Arid3b	1.7	1.8
Cercam	1.8	1.8
Ncald	1.5	1.8
Spint1	1.6	1.7
Ctsw	1.5	1.7
Klrd1	1.5	1.7
Sytl2_1	1.6	1.7
LOC102549506	1.5	1.7
RGD1305184		299.9
LOC108348096		218.7
Zmiz2		215.8
LOC100911956		30.1

Defa5	13.2
LOC257642_3	12.1
Defa7	12.0
Lctl	10.1
Stra8	9.5
Slc44a4	9.0
RatNP-3b	7.2
Fbln5	7.1
Batf2	6.7
AABR07017208.2	6.6
Tcte1	6.4
LOC100911109	6.2
AABR07052608.1	6.1
Katnal2	5.6
Kctd4	5.2
AABR07017604.1	4.9
AABR07017818.1	4.6
Tp73	4.3
Ifng	4.1
Grid2ip	3.7
LOC257642_1	3.6
Crybb3	3.6
Bace2	3.4
Klrc1	3.3
Cdkn2a	3.2
Lim2	3.1
AABR07015078.2	3.0
Fam131b	2.9
T	2.9
Cxcr6	2.8
Ubd	2.7
Fbn1	2.5
LOC690020	2.4
Snca	2.3
Cd52	2.3
Kcnk7	2.3
Neu3	2.3
Haao	2.3
Akr7a3	2.2
LOC100911980	2.2
Tanc1	2.2
Ankmy1	2.2
Erbb3	2.2
Gbp2	2.2
Slc4a1	2.2
MGC105567	2.2
Depdc1b	2.1

Tmem151b	2.1
Map6	2.1
Irgm	2.0
AABR07030901.1	2.0
Serpinb9	2.0
AABR07015080.2	2.0
Socs1	2.0
Cish	2.0
Dusp2	1.9
LOC103694857	1.9
Igtp	1.9
Hbb	1.9
RGD1309350	1.9
Gbp5	1.9
Chn2	1.9
LOC103694855	1.9
Hba-a2_1	1.9
Rnf43	1.9
LOC100910979_2	1.9
Alas2	1.9
Psat1	1.9
Hba-a2_2	1.8
Fech	1.8
Clic5	1.8
Ly49i2	1.8
LOC685067	1.8
Cdr2	1.8
Ifi47	1.8
Mpa2l	1.8
Lmo7	1.8
Eomes	1.7
Ccnb1	1.7
Nlrc5	1.7
Stat1	1.7
Gbp4	1.7
Glcci1	1.7
Aars2	1.7
Zbtb32	1.7
Mark1	1.7
Klri2	1.7
Rnf213	1.7
Dapk2	1.7
Ybx3	1.7
Bpgm	1.7
AABR07058479.1	1.6
Txk	1.6
Prr5	1.6

Syt11		1.6
Irf1		1.6
Tap1		1.6
Rnf19b		1.6
Sacs		1.6
Psmb9		1.6
Ptpn4		1.6
Cst7		1.6
Arap2		1.6
Sidt1		1.6
B4galnt1		1.6
Spata13		1.6
Lime1		1.6
Cd8a		1.5
Tpst2		1.5
Kifc1		1.5
Pxylp1		1.5
Pole		1.5
Agfg2		1.5
Abcg3l1		1.5
Sub1		1.5
Trim59		1.5
Atp2a3		1.5
Rab19		1.5
Zbp1		1.5
Cdc25b		1.5
LOC100911625	136.9	
Hpd	14.8	
LOC100912485	14.6	
Cntfr	8.5	
LOC502908	5.7	
Prkcdbp	5.0	
AABR07012775.1	4.2	
Ctxn1	2.9	
AABR07034729.1	2.7	
Ewsr1	2.2	
Mss51	2.0	
Brms1l_2	2.0	
Nfatc4	1.9	
Cfap126	1.8	
Smpd5	1.8	
Prss39	1.8	
Mapk15	1.8	
Klkb1	1.8	
Zfp958	1.8	
LOC102550456	1.7	
Begain	1.7	

Mt-nd4	1.7	
AABR07000398.1	1.7	
Mt-cyb	1.7	
Sfn	1.6	
Zfp579	1.6	
Sap25	1.6	
Slfn13	1.6	
Plekhg5	1.6	
AC141521.1	1.6	
Mt-atp6	1.6	
Mt-nd1	1.6	
Hnrnpc	1.6	
Tmem156	1.6	
Mt-cox3	1.6	
Adamts14	1.5	
Tnrc6a	1.5	
Gabbr1	1.5	
Tnfrsf25	1.5	
Rab29	1.5	
Tra2a	1.5	
Lrpap1	-1.5	-1.6
Net1	-1.5	-1.6
Xbp1	-1.6	-1.6
Cd84	-1.7	-1.7
Rps19	-1.5	-1.7
Agap1	-1.5	-1.8
Atp11a	-1.6	-1.8
Tnfrsf1b	-1.6	-1.8
Irf2bp2	-1.6	-1.8
Scarb1	-1.6	-1.8
Emb	-1.7	-1.8
Otud1	-1.8	-1.8
Irf5	-1.5	-1.8
Samsn1	-1.5	-1.8
Tfe3	-1.8	-1.8
Zfp710	-1.9	-1.9
Gyg1	-1.8	-1.9
Plbd1	-1.5	-1.9
Pcyox1	-2.1	-1.9
Pkm	-1.6	-1.9
Coro2a	-1.8	-2.0
Rnf149	-1.8	-2.0
Ctnna1	-1.6	-2.0
RGD1304694	-1.5	-2.0
Kif1b	-1.6	-2.0
Sbf2	-1.7	-2.0
Nt5e	-1.9	-2.1

Cd4	-1.8	-2.1
Map3k9	-1.8	-2.1
Anxa4	-1.6	-2.1
Psap	-1.7	-2.1
Nucb2	-1.7	-2.1
Gzmbl3	-1.9	-2.1
Dusp6	-1.5	-2.1
Ndrg1	-1.6	-2.1
Rxra	-2.2	-2.2
Gas7	-2.3	-2.2
Csf1r	-1.6	-2.2
Hist1h1c	-1.7	-2.2
Selenop	-1.8	-2.2
Clec4a1	-1.5	-2.2
Maf	-1.8	-2.2
P2ry1	-2.1	-2.2
Klf6	-2.3	-2.2
Pld4	-1.6	-2.2
Ypel2	-2.0	-2.2
Sqrdl	-1.5	-2.2
Frrs1	-1.8	-2.2
Sh2d1b	-2.0	-2.2
Tlr5	-2.1	-2.2
Cd86	-1.6	-2.3
Tlr7	-1.8	-2.3
Tnfrsf21	-1.5	-2.3
Ccdc88a	-2.3	-2.3
Tob1	-2.0	-2.3
Arhgef10l	-1.6	-2.3
Cebpa	-1.6	-2.3
Grk3	-2.2	-2.3
Cyfip1	-1.9	-2.3
Lilra5	-1.9	-2.3
Megf9	-2.6	-2.3
Padi2	-1.9	-2.3
Lgmn	-1.6	-2.4
Cxcr4	-1.9	-2.4
Soat1	-1.8	-2.4
Cdc42ep4	-1.6	-2.4
Atrnl1	-2.4	-2.4
Gpr137b	-2.0	-2.4
Ccl9	-1.8	-2.4
Adam9	-2.0	-2.5
Fam129b	-1.9	-2.5
Selenon	-2.5	-2.5
Rgs18	-1.9	-2.5
Fgd4	-1.8	-2.5

Aoah	-2.0	-2.5
Zbtb46	-2.1	-2.5
Ccrl2	-1.7	-2.6
Angpt1	-2.4	-2.6
Fpr2	-2.0	-2.6
Pld1	-1.9	-2.6
AABR07035722.1	-1.5	-2.6
Clec4a3	-1.9	-2.6
Mal	-1.8	-2.6
Cd300a	-1.7	-2.6
Card9	-1.5	-2.6
Plvap	-1.8	-2.6
Plxnb2	-1.8	-2.6
Plxdc2	-1.9	-2.6
Ppef1	-2.6	-2.6
Cd14	-2.1	-2.7
Tlr13	-1.6	-2.7
Tbc1d9	-1.8	-2.7
Colec12	-2.1	-2.7
Klf4	-1.9	-2.7
AABR07021221.1	-2.3	-2.7
LOC100910669	-1.6	-2.7
Smpdl3a	-1.9	-2.7
Itgb3	-2.2	-2.7
Ppp1r15a	-1.9	-2.8
Tubb2b	-2.0	-2.8
Adgre4	-2.6	-2.8
Zfp503	-3.0	-2.8
Cd93	-3.9	-2.8
LOC102556085	-3.1	-2.8
LOC681341	-1.6	-2.8
Ctsl	-1.9	-2.8
Ctla4	-2.1	-2.8
Lyz2	-1.8	-2.8
Nfkbiz	-2.1	-2.9
Cadm1	-2.7	-2.9
AABR07030086.2	-2.5	-2.9
Adgre1	-1.9	-2.9
Smpd3	-3.1	-2.9
Abcg2	-1.9	-2.9
Cd300le	-1.7	-2.9
Nr4a1	-1.9	-2.9
Olr292_1	-2.0	-2.9
Mctp1	-2.2	-2.9
Dusp5	-1.8	-3.0
Vcan	-2.3	-3.0
RGD1561778	-2.0	-3.0

Tlr8	-2.4	-3.0
Smpdl3b	-1.9	-3.0
AABR07062183.1	-1.8	-3.0
RGD1304884	-2.2	-3.1
Ndst1	-3.4	-3.1
Zfyve9	-2.2	-3.1
Fndc3b	-2.4	-3.1
Rin2	-1.6	-3.1
Magi1	-2.2	-3.1
AABR07006310.1	-3.4	-3.1
Fam184a	-2.5	-3.2
Trem1	-2.0	-3.2
LOC691695	-2.8	-3.2
F5	-2.1	-3.2
Rab7b	-2.1	-3.2
Ier5l	-2.0	-3.2
Fam20c	-2.2	-3.3
Ptgs2	-3.2	-3.3
Gjb2	-2.0	-3.3
Emilin2	-2.4	-3.4
Flt3	-2.4	-3.4
Dock1	-2.2	-3.4
Marveld1	-2.1	-3.4
St14	-2.2	-3.5
Dfna5	-2.4	-3.5
Kcne3	-2.7	-3.5
LOC100909879	-2.1	-3.5
Vash1	-2.7	-3.5
Lmna	-2.0	-3.5
Apba1	-3.3	-3.5
Tgfbi	-2.2	-3.5
Cadm3	-1.8	-3.5
Socs3	-2.7	-3.6
Rusc2	-2.7	-3.6
P2ry2	-2.3	-3.6
Sik1	-2.5	-3.6
Atf3	-2.6	-3.6
Trim16	-2.7	-3.6
Afdn	-3.1	-3.6
Cd300e	-2.1	-3.6
Vldlr	-3.8	-3.7
Sh3pxd2b	-9.1	-3.7
Adap2	-2.7	-3.7
RGD1560281	-1.7	-3.7
Mmp19	-2.8	-3.7
Peli2	-3.4	-3.8
Osm	-2.5	-3.8

Smim5	-2.5	-3.8
Kcns3	-2.3	-3.8
Angptl4	-2.3	-3.8
Rgs2	-2.6	-3.8
Maob	-1.8	-3.9
Tubb2a	-1.8	-3.9
Filip1	-3.3	-3.9
Trim55	-2.5	-3.9
Maoa	-2.2	-3.9
AABR07002001.1	-2.7	-4.0
RGD1559482	-2.0	-4.0
Adam15	-2.1	-4.0
Rhob	-2.2	-4.1
Rab11fip5	-3.0	-4.1
Junb	-2.1	-4.1
Alpk2	-2.7	-4.1
Serpinb6	-1.7	-4.2
Qrfpr	-1.8	-4.2
Fbln1	-2.2	-4.2
Clec10a	-2.2	-4.2
Adgra2	-2.7	-4.3
Mafb	-2.4	-4.3
Lilrb3l	-3.4	-4.3
Hs3st3a1	-7.2	-4.3
RGD1561730	-1.9	-4.3
Dlg5	-4.0	-4.4
Lrp4	-2.3	-4.4
Sowahc	-2.9	-4.4
Tf	-2.1	-4.4
Clec4a	-2.6	-4.4
Dsc1	-4.5	-4.6
Trib1	-3.7	-4.6
Cdkn1a	-2.3	-4.6
Bahcc1	-4.5	-4.7
Plcb1	-4.1	-4.7
Jun	-2.5	-4.7
Cfh	-2.7	-4.8
G0s2	-2.3	-4.9
Cd36	-2.2	-5.0
Fn1	-2.6	-5.0
Bean1	-5.5	-5.1
Thbs1	-2.6	-5.1
LOC103692471	-3.5	-5.3
Tubb1	-2.3	-5.3
Tmtc2	-3.7	-5.3
Kdr	-3.1	-5.5
Tgfa	-2.1	-5.6

Lamb3	-2.4	-5.7
Liph	-3.0	-6.1
Myo7a	-3.9	-6.2
AABR07024972.1	-5.8	-6.3
LOC679149	-2.6	-6.4
Gapt	-2.4	-6.4
Ephb2	-3.4	-6.6
Hes1	-2.6	-6.7
Mrc1	-2.5	-6.8
Lhfpl2	-2.6	-7.1
Fosb	-3.5	-7.3
Igf1	-5.7	-7.4
Egr1	-6.8	-7.7
RGD1566368	-3.6	-7.8
Thbd	-2.8	-7.8
Siglech	-4.6	-8.8
Hspa1a	-19.3	-9.4
Rps6_1	-78.0	-10.9
Angptl2	-9.4	-11.2
LOC681355	-3.8	-12.2
Fos	-3.8	-13.1
LOC100912498	-23.0	-26.9
Lif	-10.2	-27.5
Rpl35al1	-403.3	-51.5
Dcun1d2	-135.8	-145.4
Ccl2		-127.9
Ccl7		-77.2
Fam71a		-71.5
Nfs1_2		-65.5
Cxcl2		-46.2
Cxcl1		-27.4
LOC100910163		-23.1
Fam46b		-21.3
Angptl8		-19.0
Cxcl3		-17.0
Sost		-16.6
LOC100360169		-15.6
Hspb1		-14.6
Dio3		-14.4
Ceacam6		-14.3
Col3a1		-14.2
Slc36a2		-13.4
Plk2		-12.7
Tgfb3		-12.7
Sptbn2		-12.6
Fndc4		-12.5
Cd163		-11.3

Enpep	-11.2
Klf8	-11.2
Cyp26a1	-11.2
Duoxa1	-11.1
NEWGENE_621351_1	-11.0
LOC690483	-10.8
C1qa	-10.8
Frk	-10.5
Nrn1	-10.4
Ccl24	-10.2
C1qc	-10.1
Lair1	-9.6
Dact2	-9.6
Tbx4	-9.5
AABR07006033.1	-9.4
C1qb	-9.4
Pnmal2	-9.3
LOC100911548	-9.2
Msln	-8.9
Map1a	-8.8
Mamdc2	-8.7
Folr2	-8.7
Fcrl2	-8.7
Prox1	-8.6
Mast4	-8.5
AABR07053483.1	-8.5
Asic4	-8.4
Gpr39	-8.4
LOC361016	-8.4
AABR07020815.1	-8.3
Olfml2a	-8.0
Asgr2	-8.0
Gdf15	-7.9
Ltbp3	-7.9
Dagla	-7.7
Ms4a8	-7.6
Fxyd6	-7.6
Ltc4s	-7.6
Bcam	-7.4
Mpo	-7.4
Wnt4	-7.4
Vil1	-7.3
Ptgis	-7.2
Spata18	-7.2
Lpar4	-7.1
Gjc1	-7.0
Gprc5a	-7.0

Gnrhr	-7.0
Igfbp6	-6.9
Asz1	-6.9
Arhgef5	-6.9
Fhod3	-6.8
Apoc3	-6.7
Pdlim4	-6.7
Gas6	-6.6
Ier3	-6.6
Chrdl1	-6.6
Tppp3	-6.6
Pigz_2	-6.6
Fabp4	-6.6
Cep295nl	-6.5
Vgf	-6.5
Slc9a3r2	-6.4
Cxcl14	-6.3
AABR07049769.1	-6.3
Ptrf	-6.3
Rasal2	-6.3
Dusp1	-6.2
Ntsr1	-6.2
Mypn	-6.1
AABR07021357.1	-6.1
Mlxipl	-6.0
RGD1561662	-6.0
LOC103694853	-6.0
Nrcam	-6.0
LOC100360573	-6.0
Pltp	-6.0
Sytl4	-5.9
Nrxn2	-5.8
AABR07031489.1	-5.8
Spetex-2F	-5.8
Selp	-5.8
Ltbp1	-5.7
Cfd	-5.6
Axl	-5.6
Tcam1	-5.6
Mcpt4	-5.6
Pmp22	-5.6
Cyr61	-5.6
Shank3	-5.5
Lyve1	-5.5
Fscn1	-5.5
Sdpr	-5.5
Edil3	-5.5

Lonrf3	-5.5
C1ql3	-5.5
Neurl1	-5.4
Trpc6	-5.4
Sftpd	-5.4
Msr1	-5.4
RGD1565844	-5.4
Tubb6	-5.4
Eng	-5.3
Fam3b	-5.3
Trpm1	-5.3
AABR07035650.1	-5.3
Vsig2	-5.3
Klhl30	-5.3
Timd4	-5.3
Col4a1	-5.2
AC111292.2	-5.2
Enho	-5.2
Cfb_1	-5.2
Bmyc	-5.2
Pomgnt2	-5.1
Lefty2	-5.1
P4ha2	-5.1
Pde5a	-5.1
Fgfr1	-5.0
Gpr27	-5.0
Ddn	-5.0
LOC103690326	-5.0
Fbxl2	-5.0
Marc1	-4.9
Dab2	-4.9
Cachd1	-4.9
Sema3c	-4.9
RGD1310819	-4.9
Myrf	-4.9
Tspan12	-4.9
Gprc5d	-4.9
Cndp1	-4.8
LOC102547734	-4.8
Gdf3	-4.8
AABR07021022.1	-4.8
Arhgap28	-4.8
Sstr4	-4.8
Ascl2	-4.8
Adamts12	-4.7
Prg4	-4.7
F13a1	-4.7

Dchs1	-4.7
Plau	-4.7
Ak7	-4.7
Ly6g6c	-4.7
Ly6al	-4.7
Cbln2	-4.7
Nectin4	-4.7
Mpl	-4.6
Mest	-4.6
Itga2b	-4.6
Slc13a5	-4.6
Cables1	-4.6
Sash1	-4.6
NEWGENE_2724	-4.6
Muc20	-4.6
Morc4	-4.6
Pla2g2a	-4.6
AABR07052504.1	-4.5
Rhbdl3	-4.5
Gp1bb_2	-4.5
Dner	-4.5
LOC100910650	-4.5
Sec14l5	-4.5
Abcd2	-4.5
Pipox	-4.5
Clu	-4.5
Myo5b	-4.5
LOC100910801	-4.4
Fcna	-4.4
Myl9	-4.4
RGD1564129	-4.4
Slpil2	-4.4
Il17rc	-4.4
Ccnd1	-4.4
Fblim1	-4.4
Laptm4b	-4.3
Pid1	-4.3
Obscn	-4.3
Anxa3	-4.3
Treml1	-4.3
Alox12	-4.3
Egfr	-4.3
Fhl1	-4.3
Bgn	-4.3
Ggt5	-4.3
Tek	-4.2
AABR07021998.1	-4.2

Hspa1b	-4.2
Clec1b	-4.2
Myct1	-4.2
Kcng2	-4.2
Gp9	-4.2
Kcnab1	-4.2
Rarres1	-4.2
Pla1a	-4.2
Scn1b	-4.2
Mmrn1	-4.2
Bambi	-4.2
LOC685048	-4.1
G6b	-4.1
Syt5	-4.1
Psd3	-4.1
Maged1	-4.1
Serpinb10	-4.1
LOC100912228	-4.1
Gp1ba	-4.1
Serpinb8	-4.1
Mrgprx3	-4.1
Oas2	-4.1
LOC498368	-4.1
Gp6	-4.1
Ophn1	-4.1
Slc2a4	-4.1
Bcar1	-4.1
Gdpd2	-4.1
Ephb4	-4.1
Asic1	-4.0
Popdc2	-4.0
Heph	-4.0
Npnt	-4.0
F11r	-4.0
Scamp5	-4.0
Tarm1	-4.0
Gpx3	-4.0
Prkd1	-4.0
RGD1311575	-4.0
Pde3a	-4.0
AABR07053483.2	-4.0
Rfx2	-4.0
Serpinb11	-3.9
Etv1	-3.9
Tgfb2	-3.9
Ddr1	-3.9
Trim36	-3.9

Lpl	-3.9
AABR07030793.1	-3.9
Duox1	-3.9
Acvr2b	-3.9
Timp2	-3.9
Acer2	-3.9
Tmem117	-3.9
Il1r2	-3.9
Tac4	-3.9
Semg1	-3.8
Lgals2	-3.8
Epb41l1	-3.8
Papln	-3.8
Cxxc4	-3.8
Gng11	-3.8
Rpp25	-3.8
Mylk	-3.8
AABR07044820.1	-3.8
Slc40a1_2	-3.8
Cav1	-3.8
Carmil1	-3.8
Fads1	-3.8
Cux2	-3.8
Tgm1	-3.7
Prok2	-3.7
F2rl2	-3.7
Fam135a	-3.7
LOC100911403	-3.7
Bglap	-3.7
Ly6g6f	-3.7
Zdhhc1	-3.7
Fam198b	-3.7
Rtp3	-3.7
Me1	-3.7
Mb21d2	-3.7
Npy	-3.7
Col4a4	-3.7
Vnn3	-3.7
Zfp111	-3.7
Pf4	-3.6
Slc6a4	-3.6
AABR07006278.1	-3.6
Mrvi1	-3.6
Soat2	-3.6
Kcnk13	-3.6
Pparg	-3.6
Nptxr	-3.6

Lingo3	-3.6
Parvb	-3.6
Fst	-3.6
Ceacam4	-3.6
Eepd1	-3.5
Enkur	-3.5
Ccdc80	-3.5
Acsbg1	-3.5
B3galnt1	-3.5
AABR07036301.1	-3.5
Bco1	-3.5
Adcy10	-3.5
Tmem176b	-3.5
Clec4b2	-3.5
Sema6d	-3.5
Siglec1	-3.5
Zbtb16	-3.4
Hcar2	-3.4
Ston2	-3.4
Abca17	-3.4
Ddah1	-3.4
Flnb	-3.4
Trem2	-3.4
Mcpt2	-3.4
LOC680254	-3.4
Ackr3	-3.4
Ttyh2	-3.4
Adamts2	-3.4
Mfap3l	-3.4
F10	-3.4
Cttn_1	-3.4
Timp3	-3.4
Cd276	-3.4
Akr1b10	-3.3
Fat1	-3.3
Tpd52l1	-3.3
Epha1	-3.3
Slc32a1	-3.3
Cntnap2	-3.3
Il1r1	-3.3
Ca5b	-3.3
Siglec8	-3.3
Gpm6b	-3.3
Slpi	-3.3
Il11ra1	-3.3
Clec4f	-3.3
Bmx	-3.3

Kifc3	-3.2
Hp	-3.2
Reep2	-3.2
Ppbp	-3.2
Plxna3	-3.2
Depdc7	-3.2
Bmp2	-3.2
Sdk2	-3.2
Rab38	-3.2
Alpl	-3.2
Ccdc92	-3.2
Gprc5c	-3.2
Uaca	-3.2
LOC103691261	-3.2
Rhbdf1	-3.2
Fpr1	-3.2
Gnaz	-3.2
Snta1	-3.2
Ccr1	-3.2
Clec7a	-3.2
Cyp2d1	-3.2
Tyro3	-3.2
Nbl1	-3.2
Tspan18	-3.2
Ptpn13	-3.2
P2ry12	-3.2
Lamb2	-3.1
Trim47	-3.1
Capn3	-3.1
Gpr34	-3.1
Cnih2	-3.1
Tbxas1	-3.1
Slc24a3	-3.1
Pglyrp4	-3.1
AABR07035776.1	-3.1
Acvr1	-3.1
Pdgfa	-3.1
Dennd2c	-3.1
Gpnmb	-3.1
AABR07030773.1	-3.1
Slc22a23	-3.1
Defb14	-3.1
Adgrg6	-3.1
Gatm	-3.1
Gucy1b3	-3.1
Dok4	-3.1
Serpinb1a	-3.0

Sh2d1b2	-3.0
Slc18a2	-3.0
Clca2	-3.0
Wwc2	-3.0
Gmpr	-3.0
Cpne8	-3.0
Adgra3	-3.0
Nwd1	-3.0
RGD1559588_1	-3.0
Cfp	-3.0
Slc7a8	-3.0
Scgb3a1	-3.0
Abcc3	-3.0
Zfp532	-3.0
Gsta1	-3.0
Mapk13	-3.0
Rnase4	-3.0
AABR07044454.1	-2.9
Nlrp12	-2.9
Crim1	-2.9
LOC100910838	-2.9
Wfdc15b	-2.9
B3gnt3	-2.9
Tsc22d1	-2.9
Rnf112	-2.9
LOC100912068	-2.9
Ptk7	-2.9
LOC689230	-2.9
Pam	-2.9
Steap3	-2.9
Card10	-2.9
Slamf9	-2.9
Ltb4r	-2.9
Kcns1	-2.9
LOC691418	-2.9
Tcn2	-2.8
Rbm47	-2.8
Capn12	-2.8
Camk1	-2.8
Anxa1	-2.8
Cd300lb	-2.8
Ptgs1	-2.8
Steap4	-2.8
Meis1	-2.8
RGD1311756	-2.8
Retnlg	-2.8
Pon3	-2.8

Id1	-2.8
Vegfa	-2.8
Phldb1	-2.8
Rhag	-2.8
C5ar2	-2.7
Tm6sf1	-2.7
Palld_2	-2.7
Siglec5	-2.7
Zc3h12c	-2.7
Dmxl2	-2.7
LOC108348047	-2.7
Clec4a2	-2.7
Large1	-2.7
LOC100360575_1	-2.7
Gp1bb_1	-2.7
Rgs6	-2.7
Lilrb3a	-2.7
Tgfbr3	-2.7
Tmem37	-2.7
Cald1	-2.7
Cecr6	-2.7
Ifit3	-2.7
RGD1307182	-2.7
Dgat2	-2.7
Porcn	-2.7
Clec4d	-2.7
Syt3	-2.7
Mrc2	-2.7
Glis2	-2.7
Mtus1	-2.7
Ecm1	-2.7
Arl11	-2.7
Pir	-2.7
Ces2c	-2.7
Gsdma	-2.7
Npl	-2.7
Lrrc49	-2.7
Csf3r	-2.6
LOC685157	-2.6
Zfp36	-2.6
C1rl	-2.6
Npr1	-2.6
Ldhc	-2.6
Olr1	-2.6
Tmem176a	-2.6
AABR07058124.4	-2.6
Cd33	-2.6

Clec4e	-2.6
Ifit2	-2.6
RGD1561149	-2.6
Fxyd2	-2.6
Cttnbp2nl	-2.6
LOC100911825	-2.6
Ggn	-2.6
Mnda	-2.6
Dsg3	-2.6
Ctbp2	-2.6
Shc2	-2.6
LOC680910	-2.6
Pla2g7	-2.6
Arhgap18	-2.6
Gchfr	-2.6
Ptafr	-2.6
Ninj1	-2.6
Ifit1	-2.6
Lgals3	-2.6
Apobec1	-2.6
Opn3	-2.5
Gfi1b	-2.5
Lilrc2	-2.5
LOC103690020	-2.5
Gadd45a	-2.5
Plp1	-2.5
Arhgap22	-2.5
Lyc2	-2.5
Oasl	-2.5
Lbp	-2.5
Ifitm3	-2.5
Rasgrp4	-2.5
Numbl	-2.5
Clec5a	-2.5
Slc37a2	-2.5
Sox13	-2.5
Prtfdc1	-2.5
Ccl6	-2.5
Rufy4	-2.5
Rab13	-2.5
Ankrd33b	-2.5
Ccr4	-2.5
Efna2	-2.5
B4galt6	-2.5
Fgf12	-2.5
Cttn_2	-2.5
Nfil3	-2.5

LOC103689942	-2.5
Ctdspl	-2.5
Slc16a3	-2.5
Cebpb	-2.5
Alox5ap	-2.5
Cxcr2	-2.5
S100a4	-2.5
Plaur	-2.5
Gas2l1	-2.5
Dbn1	-2.5
Nrgn	-2.5
Sspo	-2.5
Coro6	-2.5
Cpq	-2.5
Sh3bp4	-2.5
Aqp9	-2.4
Tmcc3	-2.4
Eps8l1	-2.4
Esam	-2.4
RGD1561143	-2.4
Igsf7	-2.4
Il13ra1	-2.4
Zfp703	-2.4
Slc44a1	-2.4
Chst13	-2.4
Cebpe	-2.4
Hsf4	-2.4
Adssl1	-2.4
Sgms2	-2.4
Sort1	-2.4
Gng8	-2.4
Ogfrl1	-2.4
P2ry13	-2.4
Rgl1	-2.4
Panx2	-2.4
Nfe2	-2.4
AABR07041096.1	-2.4
Itga5	-2.4
Anpep	-2.4
Crybg3	-2.4
Atp1a3	-2.4
Csf2ra	-2.4
Smagp	-2.4
Dnajc22	-2.4
Anxa5	-2.4
Aldh3b1	-2.4
Slc22a15	-2.4

Cebpd	-2.4
Pdzd3	-2.4
Fosl2	-2.4
Gpt2	-2.3
Nmnat3	-2.3
Oaf	-2.3
Pwwp2b	-2.3
Oscar	-2.3
Nilr1	-2.3
Klra2	-2.3
RGD1563354	-2.3
Tnfsf13	-2.3
Ms4a6a	-2.3
Ptprm	-2.3
Lgalsl	-2.3
Ly6g6d	-2.3
Mitf	-2.3
AABR07062800.1	-2.3
Fetub	-2.3
Xdh	-2.3
Sipa1l2	-2.3
Tal1	-2.3
Dapk1	-2.3
Lrg1	-2.3
Ptk2	-2.3
C5ar1	-2.3
Stfa2	-2.3
Megf8	-2.3
Il1b	-2.3
Mcemp1	-2.3
Il36b	-2.3
Rn60_1_2212.3	-2.3
AABR07063740.2	-2.3
Slc43a3	-2.3
Kit	-2.3
Stard8	-2.3
Nlrp3	-2.3
Lpar1	-2.3
P2rx1	-2.3
LOC24906	-2.2
Tspan8	-2.2
Pfkfb4	-2.2
Il18	-2.2
Padi4	-2.2
Ptgir	-2.2
Rhoc	-2.2
Tpd52	-2.2

Sfxn5	-2.2
AABR07006142.1	-2.2
Stom	-2.2
Ldlrad3	-2.2
Lrrc25	-2.2
Cd101	-2.2
Chi3l1	-2.2
Metrnl	-2.2
Plin2	-2.2
A3galt2	-2.2
F2rl3	-2.2
Prodh1	-2.2
Pak1	-2.2
Pram1	-2.2
Ugt1a5	-2.2
Ltbr	-2.2
Rab44	-2.2
Arhgef10	-2.2
Nlrx1	-2.2
Lyplal1	-2.2
Arhgef11	-2.2
Csf2rb	-2.2
Pglyrp1	-2.2
Lima1	-2.2
Rnf150	-2.2
Map1lc3a	-2.2
Ctsf	-2.2
Rspo1	-2.2
Tesc	-2.2
Slc7a7	-2.1
Mgst1	-2.1
Fth1	-2.1
Pi16	-2.1
C3ar1	-2.1
S100a6	-2.1
Wdfy3	-2.1
Ncf4	-2.1
Ngfr	-2.1
Hlx	-2.1
Cass4	-2.1
Lpcat2	-2.1
Mras	-2.1
Ptpro	-2.1
Itgb5	-2.1
LOC100911851	-2.1
Ldlr	-2.1
Plk3	-2.1

Stau2	-2.1
Cdc42bpb	-2.1
Naaa	-2.1
Agpat2	-2.1
Rilp	-2.1
Stfa2l2	-2.1
S100a9	-2.1
Nf1x	-2.1
Sirpa	-2.1
Grn	-2.1
Klf10	-2.1
LOC100910636_2	-2.1
Tmem51	-2.1
Igsf6	-2.1
Tspan9	-2.1
Gcnt2	-2.1
LOC100360575_2	-2.1
Hacd4	-2.1
Naprt	-2.1
Ppp1r3d	-2.1
Cd200r1	-2.1
Dgat1	-2.1
Ets2	-2.0
Itga6	-2.0
Fes	-2.0
Gk	-2.0
Cd63	-2.0
RGD1311946	-2.0
Palm	-2.0
Etv5	-2.0
Fcgr2b	-2.0
Clec12a	-2.0
Wipi1	-2.0
Apoe	-2.0
Ncf2	-2.0
Grk5	-2.0
Marcks	-2.0
Btg2	-2.0
Nqo1	-2.0
Amz1	-2.0
Zcchc24	-2.0
Mboat1	-2.0
Ctsh	-2.0
Nqo2	-2.0
S100a8	-2.0
Dusp3	-2.0
Kmo	-2.0

Ier2	-2.0
Dpep2	-2.0
Tex12_1	-2.0
Rarg	-2.0
Rbm20	-2.0
Trim13	-2.0
Mocos	-2.0
Pik3r6	-2.0
Dtx4	-2.0
Lipa	-2.0
Isg15	-2.0
Fhl3	-2.0
Rab32	-2.0
Cd68	-2.0
Cpne2	-2.0
Cd302	-1.9
Plekhg3	-1.9
Tbc1d2	-1.9
Hck	-1.9
Gng10	-1.9
Rab31	-1.9
Abcb6	-1.9
Slc15a3	-1.9
P4ha1	-1.9
Arsa	-1.9
Pc	-1.9
Mpzl2	-1.9
Mafg	-1.9
S100a11	-1.9
Dap	-1.9
Sulf2	-1.9
Lrp1	-1.9
Krt75	-1.9
Pdlim1	-1.9
Il1rn	-1.9
Sema6b	-1.9
Asah1	-1.9
Clip2	-1.9
Hebp1	-1.9
Mmp9	-1.9
Cybrd1	-1.9
Gm2a	-1.9
Rhov	-1.9
Oas1b	-1.9
Alcam	-1.9
Rnf11l1	-1.9
Ethe1	-1.9

Irf7	-1.9
Slc11a1	-1.8
Trem3	-1.8
Mfsd6	-1.8
Mefv	-1.8
Tspan31	-1.8
Blvra	-1.8
Tbc1d8	-1.8
Mboat7l1	-1.8
Rcn3	-1.8
Gsr	-1.8
Pqlc1	-1.8
Pmaip1	-1.8
Cnksr3	-1.8
Spi1	-1.8
Inf2	-1.8
Xcl1	-1.8
Hsd11b1	-1.8
Unc93b1	-1.8
Mrps6	-1.8
Npc2	-1.8
Ddit3	-1.8
Whrn	-1.8
Plekhb2	-1.8
Themis2	-1.8
Dusp22	-1.8
Ralb	-1.8
Ctsz	-1.8
Tnfrsf1a	-1.8
Slc29a3	-1.8
Snx18	-1.8
Arhgap24	-1.8
Plcb2	-1.8
Nlrc4	-1.8
Msrb1	-1.8
Mgst2	-1.8
Ctbs	-1.8
Pim1	-1.8
Jaml	-1.8
Rps6ka2	-1.8
Nudt4	-1.8
Capg	-1.8
Cd244	-1.7
Aplp2	-1.7
Ampd3	-1.7
Hist1h2bcl1	-1.7
Itgb7	-1.7

Osgin1	-1.7
Tax1bp1	-1.7
Fcgr1a	-1.7
Napsa	-1.7
Chst12	-1.7
Ier5	-1.7
Tns3	-1.7
Lyl1	-1.7
Lmo2	-1.7
H3f3b	-1.7
Cklf	-1.7
Ly49si1	-1.7
Tp53inp2	-1.7
Mgrn1	-1.7
Mocs1	-1.7
Ece1	-1.7
Fgr	-1.7
Cndp2	-1.7
Ifngr2	-1.7
Eno3	-1.7
Ehd4	-1.7
Tax1bp3	-1.7
Cybb	-1.7
Scamp1	-1.7
Nfam1	-1.7
Cln8	-1.7
Calhm2	-1.7
Rassf4	-1.7
Hip1	-1.7
Zc3h12a	-1.7
Dhrs3	-1.7
Ckb	-1.7
Tlr2	-1.7
Lamp2	-1.7
Atp13a2	-1.7
Lilrb4	-1.7
Tkt	-1.7
Dram1	-1.7
Ephx1	-1.6
Ubc	-1.6
Exo5	-1.6
Card19	-1.6
Ap1s2	-1.6
Cyp4f18	-1.6
Fry	-1.6
Oat	-1.6
Fcho2	-1.6

Tspo	-1.6
Slc28a2	-1.6
Gamt	-1.6
Glipr1	-1.6
Bst1	-1.6
Asph	-1.6
Nadk	-1.6
Plcd1	-1.6
Cyb5r1	-1.6
Tmem184b	-1.6
Gpx1	-1.6
Cd44	-1.6
Dse	-1.6
Agl	-1.6
Plcb3	-1.6
Naga	-1.6
Skap2	-1.6
Taldo1	-1.6
Atp1a1	-1.6
Prdx5	-1.6
Pgap2	-1.6
Micu1	-1.6
Bcl6	-1.6
Lyn	-1.6
Srgap2	-1.6
Spg20	-1.6
Mfsd13a	-1.6
Ctsb	-1.6
Slc35e3	-1.6
Tie1	-1.6
Daglb	-1.6
Idh1	-1.6
Il17ra	-1.6
Dpy19l1	-1.6
Vim	-1.5
Acer3	-1.5
Ptprj	-1.5
Mx2	-1.5
Dhdds	-1.5
Tspan2	-1.5
Sat1	-1.5
Aldh2	-1.5
Rell1	-1.5
LOC108348064	-1.5
Gaa	-1.5
ENSRNOG00000047276	-1.5
Dok1	-1.5

Pdia5		-1.5
Adam8		-1.5
Ctsd		-1.5
Cd1d1		-1.5
Agpat4		-1.5
LOC100359687		-1.5
Cast		-1.5
Prcp		-1.5
Fam43a		-1.5
Mbp	-1.5	
Atp2c1	-1.5	
Vps13c	-1.6	
Nek9	-1.6	
Zfp106	-1.6	
Pi4k2a	-1.6	
Dpysl2	-1.6	
AABR07043421.1	-1.6	
Samhd1	-1.7	
Olfm1	-1.7	
Adipor2	-1.7	
Tiam1	-1.7	
March7	-1.7	
Atf6	-1.7	
Tcf12	-1.7	
Ptbp3	-1.7	
Slk	-1.7	
Exoc4	-1.8	
Wasf2	-1.8	
Acvrl1	-1.8	
Lims1	-1.8	
Inpp4a	-1.8	
Dnajc16	-1.8	
Abhd2	-1.8	
Dgkg	-1.9	
Scin	-1.9	
Rab43	-1.9	
Dock2	-1.9	
Slc6a6	-1.9	
rnf141	-1.9	
Cpne3	-1.9	
Arrb1	-2.0	
Tnfaip3	-2.0	
AABR07051533.2	-2.0	
Itsn1	-2.0	
Fcnb	-2.1	
Bach1	-2.1	
AABR07065823.1	-2.1	

Ids	-2.1
Gda	-2.1
Vwa8	-2.1
Nr3c1_2	-2.2
Trps1	-2.2
Kank1	-2.2
Clec9a	-2.2
LOC685933	-2.3
Gcsam	-2.3
LOC108348118	-2.3
ENSRNOG000000517	-2.3
Tlr12	-2.3
Myh9l1	-2.4
Kcna3	-2.4
AABR07065676.1	-2.5
Cers6	-2.5
Fnip2	-2.6
Fam102b	-2.6
Slamf7	-2.6
AABR07065821.1	-2.6
Slc8a1	-2.8
Frmd4a	-2.8
Cdh5	-2.9
Arhgap5	-2.9
NEWGENE_1310139	-3.0
Plekhm3	-3.1
Rpl13	-3.3
AABR07027272.1	-3.4
Fam199x	-3.5
AABR07033887.1	-3.7
Notch3	-4.0
Hist1h2an	-4.0
Ccr2	-4.4
Soga1	-4.4
Fmn1	-5.0
LOC102556337	-7.2
Reps2	-16.8
LOC102551606	-19.4
Ncbp2_2	-130.4
Mepce	-186.1
LOC100361457	-335.0
Oas1i	-411.3
Impad1_2	-587.1

**Genes numbers**	446	1372

RNAseq gene expression. Day 24
Fold change: < 1.5 or >1.5
FDR p-value: < 0.05

	LVS / PBS	ΔclpB / PBS
	Day 24	Day 24
Spink2	193.9	698.1
LOC100911625	105.7	193.5
Gzmc	292.7	122.7
LOC100911956	95.6	95.6
Creb3l4	79.8	64.2
Grcc10	341.4	59.6
Hprt1	47.9	58.6
Il34	3.5	39.2
LOC257642_3	29.1	31.1
Pianp	4.2	15.4
Msc	5.4	13.6
AABR07029202.1	3.2	12.9
Rn60_10_0910.5	19.0	12.3
Rn60_7_0127.2	18.8	11.5
Dclk1	2.4	10.2
Arl14epl	4.1	9.6
AC118414.1	15.6	9.6
LOC100911615	6.4	9.4
Mk1	15.1	9.1
Heatr9	2.4	8.7
Tcte1	9.1	8.7
AABR07072400.1	13.8	8.5
LOC100910138	3.5	7.4
Rn60_19_0375.6	8.2	7.2
LOC257642_1	6.7	7.2
AABR07017597.1	3.9	7.0
Ifng	2.7	7.0
Rn60_7_0127.5	10.8	5.7
Klrc1	2.0	5.6
T	2.5	5.4
AABR07008940.1	7.1	5.3
Pp2d1	11.7	5.2
Atcay	2.4	5.1
Sptbn4	2.3	5.0
Ly49s6	2.3	4.5
Cxcr6	1.9	4.4
Chchd2	3.9	4.4
AABR07015078.2	3.4	4.2
RGD1307461	2.6	4.0
Il18r1	1.5	3.5
Cpne7	1.7	3.5

	Up-regulated genes	
	Count	
	LVS	ΔclpB
Both vaccines	75	75
LVS only	19	
ΔclpB only		359
Total Up-regulated	94	434

	Down-regulated genes	
	Count	
	LVS	ΔclpB
Both vaccines	260	260
LVS only	41	
ΔclpB only		901
Total down-regulated	301	1161
Total genes	395	1595

Il12rb1	1.8	3.4
Il18rap	1.8	3.4
Crb3	4.3	3.3
Map6	1.7	3.3
Tead3	4.8	3.2
Ccl5	1.8	3.1
Tnni3k	2.8	3.0
Tlcd1	4.1	3.0
Klra1	3.3	2.9
Tbx21	1.9	2.9
Tmem79	4.2	2.9
Gzmm	1.7	2.9
Prdm1	1.6	2.8
Klrg1	1.6	2.7
Best1	5.6	2.6
LOC102551606	2.3	2.6
Cish	1.6	2.5
Rpl26-ps1	4.3	2.5
Cd2	1.6	2.4
Neu3	1.8	2.4
Sema7a	1.7	2.3
Nkg7	1.7	2.3
Esrp2	3.2	2.3
Mt-nd6	1.9	2.3
LOC100911917	2.2	2.2
Faslg	1.6	2.2
Ly49s4	2.3	2.1
AABR07034641.1	2.7	2.0
Fam19a3	1.6	1.8
Eomes	1.5	1.8
Csf1	1.8	1.7
Susd2	2.0	1.7
Cxcr3	1.6	1.7
LOC100910882	1.6	1.6
Hsp90aa1		2451.6
LOC103690175		171.7
LOC108348096		161.5
Camk1g		154.6
LOC100362298		76.4
Xirp1		57.2
RGD1561252		55.1
RGD1305184		53.1
LOC100911166		51.4
Tprkb		44.9
Clip3		40.5
Fgf2		37.3
Fbln5		16.4

LOC100910979_1	14.7
Lrrc39	12.9
Slc44a4	11.7
Lctl	11.3
Stra8	10.8
Fat4	8.9
Gng3	8.4
Hapln4	7.6
1700066B19Rik	7.5
AABR07030544.1	7.3
AABR07052608.1	7.1
Rhou	6.9
Abca5	6.6
Slc40a1_1	6.5
Katnal2	6.3
Nova2	6.0
AABR07002906.1	5.9
Cntfr	5.9
Fam131b	5.6
Cgn	5.2
Kctd4	5.2
Ptprg	5.1
AABR07064878.1	5.0
Tp73	4.9
LOC100910446	4.8
LOC502908	4.8
AABR07060952.1	4.7
Slc4a8	4.7
Fgf13	4.6
Pcyt1b	4.5
AABR07017669.1	4.5
Ltbp4	4.5
Tnfsf9	4.4
Atxn1	4.3
AABR07017818.1	4.3
Exoc3l2	3.9
AABR07002898.1	3.9
Zfp14	3.8
Nkpd1	3.6
Rora	3.6
AABR07060953.1	3.5
Ccdc177	3.5
Ccl4	3.5
AABR07004993.1	3.5
Muc13	3.5
Emp1	3.4
Ankmy1	3.4

AABR07002677.2	3.3
Clcn1	3.2
En1	3.2
Cdkn2a	3.1
Kmt2a	3.1
AABR07037528.1	3.0
Edaradd	3.0
Kcnk5	2.9
Tanc1	2.9
Il12rb2	2.9
Ptger1	2.9
AABR07017748.2	2.9
Ramp2	2.8
Klrc3	2.8
Pla2g2f	2.8
LOC690020	2.7
Nr4a3	2.7
AABR07017825.4	2.7
A2ml1	2.7
Nfatc2	2.6
Slc27a2	2.6
AABR07067267.1	2.6
Sbspon	2.6
Vat1l	2.5
LOC690288	2.5
AABR07017737.1	2.5
Ikzf3	2.5
Farp1	2.5
Plxnd1	2.5
Rhbdl1	2.5
Clic5	2.5
Ctxn1	2.5
Thsd7b	2.4
AABR07059004.1	2.4
Nlrc5	2.4
Zic3	2.4
Slco1a2	2.4
Peak1	2.4
Syde1	2.4
Rnf43	2.4
Tshr	2.4
Birc6	2.4
Kcnq5	2.3
Mt-nd5	2.3
Ptpn5	2.3
Ppp1r12b	2.3
Slc4a7	2.3

Kcnk7	2.3
Tmem151b	2.3
Mpa2l	2.3
Adam11	2.3
LOC100911980	2.3
Cacna2d2	2.3
Rn50_X_0752.3	2.3
Pcgf2	2.3
AABR07019085.1	2.3
Akap2	2.3
LOC499229	2.3
Amer1	2.3
Ctsw	2.3
Erbb3	2.2
Cep76	2.2
ENSRNOG00000055238	2.2
Dusp2	2.2
Klhl3	2.2
Lrrc75b	2.2
Ino80d	2.2
Cdhr1	2.2
Themis	2.2
Cx3cr1	2.2
Cers4	2.2
Rictor	2.2
LOC108348118	2.2
Brsk1	2.2
Zfp819	2.2
Acod1	2.2
Amt	2.2
Samd3	2.1
AC120712.2	2.1
Epb41l5	2.1
LOC682206	2.1
Arid4b	2.1
Espn	2.1
Hnf1a	2.1
Rn50_13_0834.1	2.1
Serpinb9	2.1
Hormad2	2.1
Cep350	2.0
Vps13a	2.0
Chn2	2.0
AABR07021996.1	2.0
ENSRNOG00000051999	2.0
LOC685067	2.0
Dock5	2.0

Sytl3	2.0
Tnfrsf25	2.0
Zfp575	2.0
Huwe1	2.0
LOC681292	1.9
AABR07058479.1	1.9
Smg1	1.9
Asb13	1.9
Gabbr1	1.9
Zfp382	1.9
Mfsd14a	1.9
Ceacam18	1.9
Srcin1	1.9
Gdf11	1.9
Fam124a	1.9
Aars2	1.9
Aim1l	1.9
Prnp	1.9
Klre1	1.9
Rrp1b	1.9
Mdn1	1.9
Kmt2c	1.9
Klhl11	1.9
C1qtnf1	1.9
Gfi1	1.9
Mdm4	1.9
Akap1	1.9
AABR07030901.1	1.9
Cdc14b	1.9
Sla2	1.8
AABR07015078.1	1.8
AABR07063424.1	1.8
Mga	1.8
Sytl2_1	1.8
RGD1560470	1.8
Dock9	1.8
Ntng2	1.8
Phlpp2	1.8
Abcg3l1	1.8
Tspear	1.8
Cacna1a	1.8
AABR07015066.1	1.8
Rnf213	1.8
Myo19	1.8
Syt11	1.8
Klrk1	1.8
AABR07035796.1	1.8

AABR07015055.1	1.8
LOC102549506	1.8
S1pr5	1.8
AABR07015080.2	1.8
Sh2d2a	1.8
Rnf19b	1.8
Bdp1	1.8
Kmt2d	1.8
Nsd1	1.8
Slamf1	1.8
Itga4	1.8
Yme1l1	1.8
Sacs	1.8
Setd1b	1.8
Ncald	1.8
Hsph1	1.8
Pole	1.8
Gpr171	1.8
Phip	1.7
Selenoi	1.7
Ubr1	1.7
Lime1	1.7
Ipo9	1.7
Macf1	1.7
Prrc2b	1.7
ENSRNOG00000056059	1.7
Tshz1	1.7
ENSRNOG00000000891	1.7
Tnik	1.7
Cdh23	1.7
Cd8a	1.7
Srrm2	1.7
LOC108348151	1.7
Syne2	1.7
Baz2a	1.7
Elf4	1.7
Klhl42	1.7
Phc3	1.7
L1cam	1.7
Zfp398	1.7
Spen	1.7
Dst	1.7
Mt-nd4l	1.7
Gzmk	1.7
Kif21b	1.7
Arid3b	1.7
Thoc2	1.7

Ythdc2	1.7
RGD1307100	1.7
Cd8b	1.7
Bhlhb9	1.7
Gbp5	1.7
Pvt1	1.7
Dhcr24	1.7
Zbtb32	1.7
Tpst2	1.7
ENSRNOG00000043164	1.7
Cercam	1.7
Crmp1	1.7
Lmo7	1.7
Atp8b4	1.6
Trio	1.6
Kat6a	1.6
Runx3	1.6
Zbtb4	1.6
Arid2	1.6
Chst10	1.6
Sidt1	1.6
Ccnd2	1.6
Mllt3	1.6
Vwa5b2	1.6
Emsy	1.6
Otud7b	1.6
Nebl	1.6
Hectd4	1.6
Setx	1.6
Gbp4	1.6
Arap2	1.6
Spata13	1.6
Bod1l1	1.6
Wrn	1.6
Agpat3	1.6
March9	1.6
Bcr	1.6
Wipf3	1.6
Herc1	1.6
B3gnt7	1.6
LOC100911562	1.6
Kmt2e	1.6
Ahnak	1.6
Otud4	1.6
Fryl	1.6
Srcap	1.6
Fam208a	1.6

Ccr5	1.6
Ralgapa1	1.6
Zfp507	1.6
Nktr	1.6
Dnhd1	1.6
Syne1	1.6
Ankrd26	1.6
Heg1	1.6
Dopey1	1.6
Grasp	1.6
Sema4a	1.6
Dapk2	1.6
Med12	1.6
Apc	1.6
Dvl3	1.6
Nlrc3	1.6
Nefh	1.6
Cd28	1.6
Ankrd44	1.6
Dhcr7	1.6
RGD1308117	1.6
Tmem181	1.6
Sos1	1.6
AABR07058985.2	1.6
Tet2	1.5
Clec2d2	1.5
Utrn	1.5
MGC105567	1.5
Atn1	1.5
RGD1309808	1.5
Mt-nd4	1.5
Ago4	1.5
Ankrd12	1.5
Fam208b	1.5
Usp44	1.5
Akap9	1.5
Gon4l	1.5
Prmt2	1.5
Pip4k2b	1.5
Slc39a14	1.5
Spint1	1.5
Herc2	1.5
Cep295	1.5
Gimap8	1.5
Tsc1	1.5
Tnrc6c	1.5
Bcl9l	1.5

Mt-atp8		1.5
Cep162		1.5
Trbv1		1.5
Bptf		1.5
Trrap		1.5
Dok2		1.5
Psat1		1.5
Mt-cyb		1.5
Ubr4		1.5
Lag3		1.5
Plcg1		1.5
RGD1311595		1.5
Polr2a		1.5
Plekhg2		1.5
Larp1		1.5
Klrb1c		1.5
Prkd3		1.5
Snrpg	190.0	
Cers1	105.7	
Akr1c14	19.0	
Fabp1	17.2	
LOC100912485	14.0	
Pdzd9	13.2	
Alb	11.8	
Hpd	9.8	
AABR07028995.2	6.3	
AC129753.1	4.3	
Dcst2	4.2	
AC130391.3	3.9	
Cycs	3.2	
Trpt1	2.3	
Rpl31l4	1.8	
Klra5_4	1.7	
Stfa2l2	1.7	
Klra5_3	1.6	
LOC103691939	1.6	
Lamp2	-1.6	-1.5
Creg1	-1.6	-1.6
Dapk1	-1.6	-1.6
Ggh	-1.6	-1.6
Frrs1	-1.7	-1.7
Ctnna1	-1.6	-1.7
Zcchc24	-1.5	-1.7
Cybb	-1.7	-1.8
Abcc3	-1.7	-1.8
Kif1b	-1.6	-1.8
Klf4	-1.6	-1.8

Plxnb2	-1.6	-1.8
Bpgm	-2.0	-1.8
Mal	-1.6	-1.8
Tnfrsf21	-1.7	-1.8
Flnb	-1.6	-1.8
Arhgef10l	-1.5	-1.8
Tbc1d9	-1.5	-1.8
C2	-2.0	-1.8
Anxa4	-1.5	-1.8
Cyfip1	-1.8	-1.8
Capg	-1.5	-1.8
Cd68	-1.5	-1.9
Ap1s2	-1.6	-1.9
Rpl9	-1.9	-1.9
Clec4a3	-1.5	-1.9
Ly49si3	-1.8	-1.9
Nfkbiz	-2.1	-1.9
RGD1561778	-1.6	-1.9
Itsn1	-2.2	-1.9
Scamp1	-1.5	-1.9
Colec12	-1.9	-1.9
Dusp6	-1.5	-1.9
Pld1	-1.7	-1.9
Ptprm	-1.9	-2.0
Psap	-1.5	-2.0
Ly49si2	-1.8	-2.0
B4galt6	-1.8	-2.0
Ctsz	-1.5	-2.0
Sulf2	-2.1	-2.0
Tnfsf13	-1.7	-2.0
Tlr7	-1.7	-2.0
Fcgr2b	-1.5	-2.0
Gjb2	-1.9	-2.0
Nucb2	-1.6	-2.0
Dpep2	-1.7	-2.1
Adgre1	-2.0	-2.1
Slc7a7	-1.8	-2.1
Gpx1	-1.5	-2.1
Npl	-1.7	-2.1
LOC103694857	-2.4	-2.1
Hbb	-2.3	-2.1
Ahsp	-3.2	-2.1
Alas2	-2.6	-2.1
Card9	-1.5	-2.1
Hba-a2_1	-2.3	-2.1
Dsg3	-1.8	-2.1
Gyg1	-1.8	-2.1

LOC102555453	-1.8	-2.1
AABR07006310.1	-2.3	-2.1
Cd300le	-1.5	-2.1
S100a4	-1.5	-2.2
Slc44a1	-1.9	-2.2
Fam129c	-2.1	-2.2
Ca5b	-2.1	-2.2
Hba-a2_2	-2.3	-2.2
Tmcc2	-2.5	-2.2
Eps8	-2.0	-2.2
Emilin2	-2.0	-2.2
Fam129b	-1.8	-2.2
Plxdc2	-2.1	-2.2
LOC103694855	-2.5	-2.2
Slc7a8	-1.7	-2.3
Rhob	-1.8	-2.3
RGD1311756	-2.2	-2.3
Rfx2	-2.2	-2.3
Csf2ra	-1.5	-2.3
Lpl	-2.5	-2.3
AABR07062183.1	-1.8	-2.3
Rin2	-1.6	-2.3
Popdc2	-2.0	-2.3
AABR07026311.1	-2.6	-2.4
Klf1	-2.3	-2.4
Tm6sf1	-1.7	-2.4
Sdc3	-2.2	-2.4
Cd200r1	-2.1	-2.4
Ugt1a5	-2.1	-2.4
Ass1	-2.2	-2.4
Lyz2	-1.6	-2.4
Oaf	-1.7	-2.4
Capn12	-2.5	-2.4
Ninj1	-1.7	-2.4
Smpdl3a	-1.9	-2.4
Lgals5	-2.7	-2.4
Npr1	-1.9	-2.4
Maoa	-2.7	-2.4
LOC24906	-1.8	-2.5
Lrp4	-2.3	-2.5
LOC103689947	-3.0	-2.5
Tgfbi	-1.8	-2.5
Dock1	-2.4	-2.5
Slc16a7	-3.3	-2.5
Mnda	-2.0	-2.5
Vcan	-2.3	-2.5
Mylk2	-3.7	-2.5

F5	-2.0	-2.5
Cd300e	-2.3	-2.5
Cfp	-1.9	-2.5
Mitf	-2.1	-2.5
Ctsl	-1.9	-2.5
Hid1	-3.0	-2.6
Trem1	-1.8	-2.6
Cd36	-1.8	-2.6
Tgfbr3	-2.6	-2.6
RGD1566138	-3.5	-2.6
Sowahc	-2.4	-2.6
Epb42	-3.8	-2.6
Tmem176a	-2.0	-2.6
Rnf128	-2.3	-2.6
Plcb1	-2.9	-2.6
Sik1	-2.2	-2.6
P2ry2	-2.2	-2.6
Rpl5l1	-2.6	-2.6
Smpdl3b	-1.7	-2.6
Ms4a6a	-1.7	-2.6
Trib1	-2.3	-2.7
RGD1559482	-1.8	-2.7
Gatm	-1.9	-2.7
Col4a4	-2.8	-2.7
Alox15	-3.0	-2.7
Vldlr	-2.2	-2.7
Adgrg6	-2.1	-2.7
Timp2	-2.0	-2.7
Rab11fip5	-2.2	-2.7
Lmna	-1.9	-2.7
Rab13	-2.1	-2.7
Cd14	-2.4	-2.7
Rgs2	-1.8	-2.8
Rnase4	-2.6	-2.8
Adam15	-2.1	-2.8
Serpinb1a	-2.2	-2.8
Ggt5	-1.8	-2.8
Psd3	-3.9	-2.8
Fam20c	-2.2	-2.9
Asrgl1	-2.0	-2.9
Mafb	-2.6	-2.9
Kcne3	-2.2	-2.9
Fcnb	-2.0	-2.9
Myo7a	-2.8	-3.0
Serpinb8	-2.1	-3.0
RGD1565355	-2.7	-3.0
Cd24	-1.9	-3.0

Il18	-2.4	-3.0
Ptgs1	-2.0	-3.0
Tmem176b	-2.3	-3.0
Carmil1	-2.6	-3.0
Il1rl1	-3.6	-3.1
Hes1	-1.9	-3.1
Fam198b	-2.9	-3.1
Tbxas1	-2.5	-3.1
Tf	-2.1	-3.1
Ppial4d	-2.0	-3.1
Kifc3	-2.7	-3.1
Hp	-1.8	-3.1
Ccnd1	-3.4	-3.2
LOC103690020	-2.5	-3.2
Dab2	-2.9	-3.2
Scn1b	-2.6	-3.2
Parvb	-2.4	-3.3
Rarres1	-3.9	-3.3
Cyp2d1	-2.7	-3.3
St14	-2.3	-3.3
Mmp19	-2.5	-3.3
Fn1	-3.5	-3.3
Pid1	-2.1	-3.3
Kdr	-2.1	-3.3
Cpq	-2.4	-3.4
Tac4	-2.4	-3.4
Rnase2	-2.1	-3.4
Tek	-2.5	-3.4
Cdkn1a	-2.1	-3.5
Trim47	-3.3	-3.6
Gprc5c	-3.5	-3.6
LOC103694853	-6.5	-3.6
LOC680254	-2.7	-3.6
Serpinb10	-3.5	-3.7
Etv1	-4.1	-3.8
Pmp22	-2.4	-3.9
Clec10a	-2.8	-3.9
Prg4	-4.1	-3.9
Aox3	-6.3	-3.9
Laptm4b	-2.3	-3.9
Gprc5d	-2.7	-4.0
Enpp3	-3.5	-4.0
Fcna	-3.1	-4.0
Wwc2	-2.9	-4.0
Tgfb2	-3.9	-4.0
Adgra3	-2.8	-4.1
Anxa3	-3.1	-4.1

RGD1310819	-3.6	-4.1
Gapt	-3.3	-4.1
Ltbp1	-4.7	-4.1
Siglech	-3.1	-4.1
Mfsd2b	-4.3	-4.2
Cfd	-2.7	-4.2
Eng	-3.4	-4.2
Atf3	-2.6	-4.2
Slamf9	-2.4	-4.2
Pomgnt2	-5.6	-4.3
Tppp3	-3.6	-4.3
Il11ra1	-4.4	-4.4
Fscn1	-4.5	-4.4
Bmp2	-4.6	-4.6
Bcar1	-4.4	-4.6
RGD1561662	-3.7	-4.6
Tubb6	-4.0	-4.6
Ptrf	-5.0	-4.6
Cfh	-3.4	-4.7
Pdlim4	-5.3	-4.7
Selp	-4.4	-4.8
AABR07024972.1	-6.3	-4.8
Ms4a8	-8.2	-4.8
Cpe	-4.5	-4.8
LOC100360940	-4.7	-4.9
P4ha2	-3.7	-4.9
Fbln1	-2.8	-4.9
Egr1	-4.8	-5.1
Gprc5a	-5.8	-5.2
Thbd	-3.7	-5.5
Pltp	-4.5	-5.6
Fam20a	-6.8	-5.8
Ier3	-3.4	-5.9
Axl	-6.5	-6.1
Cfb_1	-5.4	-6.2
Bmyc	-5.5	-6.4
Msr1	-4.5	-6.5
LOC100912034	-3.6	-6.6
Shank3	-6.5	-6.8
Ntm	-10.5	-7.2
Gjc1	-10.5	-7.5
Vil1	-6.8	-7.9
Plk2	-6.3	-8.2
C2cd4b	-19.3	-8.6
Hspb1	-5.5	-8.6
Mamdc2	-6.0	-8.7
Cd300lg	-13.0	-9.8

Cxcl2	-6.8	-9.8
Plau	-4.3	-9.9
Prox1	-27.8	-12.0
LOC361016	-22.5	-13.4
Cyp26a1	-8.2	-14.6
Slc36a2	-9.8	-15.4
Lif	-13.0	-15.8
Ccl7	-9.0	-16.1
Npy	-4.5	-19.9
Ccl2	-8.8	-20.8
RGD1563818	-22.9	-22.2
LOC100912498	-15.0	-27.8
Fam71a	-26.1	-69.2
Sccpdh	-60.0	-339.8
Cmpk1		-1.5
Tspan2		-1.5
RT1-T24-4		-1.5
Themis2		-1.5
RGD1311739		-1.5
Mras		-1.5
Atf5		-1.5
Cstb		-1.5
Dhx58		-1.5
Ypel5		-1.5
Micu1		-1.5
Mrpl33		-1.5
Rnf149		-1.5
Nfu1		-1.5
Rbpms		-1.5
Adap1		-1.5
Dok1		-1.5
Map3k9		-1.5
LOC100911104		-1.5
Clstn1		-1.5
LOC100359687		-1.5
Nilr1		-1.5
Cd99		-1.5
Pink1		-1.5
Hexb		-1.5
Cnksr3		-1.5
Naga		-1.5
Plcb2		-1.5
Soat1		-1.5
Hmox1		-1.5
Ctsd		-1.5
March2		-1.5
Csf1r		-1.5

Asah1	-1.5
Sgpp1	-1.5
RGD1561143	-1.5
Begain	-1.5
Hagh	-1.5
Trem3	-1.5
LOC691141	-1.5
Mboat7l1	-1.6
Nfam1	-1.6
Pkib	-1.6
Aldh2	-1.6
Tmem50b	-1.6
LOC102546864	-1.6
Card19	-1.6
Jam2	-1.6
Plekho1	-1.6
Tlr2	-1.6
Vim	-1.6
Sort1	-1.6
Sat1	-1.6
Dusp3	-1.6
Rasgrp3	-1.6
Kdelc2	-1.6
Aplp2	-1.6
Ticam2	-1.6
AABR07035722.1	-1.6
Ly86	-1.6
Sirpa	-1.6
LOC108348064	-1.6
Bst2	-1.6
Tkt	-1.6
Il1rn	-1.6
Csrp1	-1.6
Clip2	-1.6
LOC100910270	-1.6
Klf10	-1.6
Cklf	-1.6
Ap3s1	-1.6
Igsf6	-1.6
Otud1	-1.6
Atox1	-1.6
Zfp385a	-1.6
Tyrobp	-1.6
Lst1	-1.6
Sod2	-1.6
Gys1	-1.6
Cd300lb	-1.6

Mmp9	-1.6
Lyn	-1.6
LOC681325	-1.6
Lta4h	-1.6
Ets2	-1.6
Agap1	-1.6
Tnfrsf1a	-1.6
Cd244	-1.6
Tbc1d2	-1.6
Acer3	-1.6
Nlrp3	-1.6
Slc2a6	-1.6
Slc37a2	-1.6
Idh1	-1.6
Taldo1	-1.6
ENSRNOG00000047276	-1.6
Ifngr2	-1.6
RGD1304694	-1.6
Hhex	-1.6
Gna12	-1.6
Adcy4	-1.6
Crat	-1.6
Isoc2b	-1.6
Net1	-1.6
Gsr	-1.6
Lmo2	-1.6
Ctbs	-1.6
Lima1	-1.6
Mkrn1	-1.6
Dgat1	-1.6
Nipsnap3b	-1.6
Slc29a3	-1.6
Cxcr4	-1.6
Pqlc1	-1.6
Ephx1	-1.6
Mcub	-1.6
Trex1	-1.6
Ppp1r15a	-1.6
H1fx	-1.6
AABR07051639.1	-1.6
Ddit3	-1.6
LOC498154	-1.6
Ptgir	-1.6
Cln8	-1.6
Scarb1	-1.6
Arhgap24	-1.6
Agpat2	-1.6

Atp1a3	-1.6
Dok3	-1.6
Itga5	-1.6
Slc11a1	-1.6
Armcx3	-1.6
Pkm	-1.6
LOC100910669	-1.6
Rnf166	-1.7
Rcn3	-1.7
Mefv	-1.7
Lpcat2	-1.7
Ethe1	-1.7
Nr1h3	-1.7
Tuba1c	-1.7
Abcb1a	-1.7
S100a11	-1.7
Ehd4	-1.7
Pla2g4a	-1.7
AABR07002969.1	-1.7
Pls1	-1.7
Tie1	-1.7
Abhd14b	-1.7
Tlr13	-1.7
Npc2	-1.7
H3f3c	-1.7
Zfp36	-1.7
Mrps6	-1.7
RGD1560281	-1.7
Snx18	-1.7
Tifab	-1.7
Abcb6	-1.7
Glrx	-1.7
Ncf2	-1.7
Plin2	-1.7
Cndp2	-1.7
Ckb	-1.7
Lipa	-1.7
Lilrc2	-1.7
Cd7	-1.7
Ftl1	-1.7
Itgb7	-1.7
Hist1h2bcl1	-1.7
Klri1	-1.7
Fcgr1a	-1.7
Itgad	-1.7
Tspan31	-1.7
Irf5	-1.7

Ldlrad3	-1.7
Napsa	-1.7
Ptpro	-1.7
Oas3	-1.7
Lbp	-1.7
Hck	-1.7
Dpy19l1	-1.7
Oas1b	-1.7
Hyal2	-1.7
Mef2b	-1.7
Ndrg1	-1.7
Fes	-1.7
Cyp4f18	-1.7
Gpr137b	-1.7
Xcl1	-1.7
Upp1	-1.7
Tmcc3	-1.7
Lrrc25	-1.7
Tp53inp2	-1.7
Snx21	-1.7
Gamt	-1.7
Chi3l1	-1.7
Fam43a	-1.7
Chst12	-1.7
RT1-DMb_1	-1.7
Mocos	-1.7
Smagp	-1.7
Tspan5	-1.7
Tspo	-1.7
LOC680910	-1.7
Pon3	-1.7
Rnf11l1	-1.7
Rassf4	-1.7
Scimp	-1.7
Zfyve9	-1.7
Ncf1	-1.7
Blvra	-1.7
Clec12a	-1.7
Slc15a3	-1.7
P2ry6	-1.7
Clec4d	-1.7
Spi1	-1.7
Gm2a	-1.7
Nqo1	-1.8
Ogfrl1	-1.8
Msrb1	-1.8
Aif1	-1.8

Kcnk1	-1.8
Mcemp1	-1.8
Scin	-1.8
Dusp5	-1.8
RT1-DMb_2	-1.8
Ppp1r3d	-1.8
Plaur	-1.8
Slamf8	-1.8
Rilp	-1.8
Fcgrt	-1.8
Rgl1	-1.8
Pwwp2b	-1.8
B3gnt3	-1.8
Padi4	-1.8
Tax1bp1	-1.8
Prdx5	-1.8
Slc16a3	-1.8
Bst1	-1.8
Rab31	-1.8
Fabp5	-1.8
Zfp703	-1.8
Ctsb	-1.8
Ly49si1	-1.8
Sipa1l2	-1.8
Bmx	-1.8
Klra2	-1.8
Rhov	-1.8
Lamb3	-1.8
Siglec5	-1.8
Spns3	-1.8
Xdh	-1.8
Znf768	-1.8
Pik3r6	-1.8
Itga6	-1.8
Csf3r	-1.8
Ifi30	-1.8
Dusp22	-1.8
Dap	-1.8
LOC100360087	-1.8
Rspo1	-1.8
RGD1561730	-1.8
Pmaip1	-1.8
Fuca2	-1.8
Lrp12	-1.8
Fbxw8	-1.8
S100a8	-1.8
Kank2	-1.8

Fhl3	-1.8
Gadd45b	-1.8
Hsd3b7	-1.8
S100a9	-1.8
Lrg1	-1.8
C1qtnf6	-1.8
AABR07002001.1	-1.8
Nlrx1	-1.8
Ptafr	-1.8
Ctbp2	-1.8
Tnfsf12	-1.8
Slc22a15	-1.8
LOC497796	-1.8
Selenop	-1.8
Tmem86a	-1.9
RGD1304884	-1.9
Cdc42ep4	-1.9
Cpped1	-1.9
Arl11	-1.9
Acvrl1	-1.9
Gpat3	-1.9
Tifa	-1.9
Anpep	-1.9
Pglyrp1	-1.9
Ptk2	-1.9
Tgfa	-1.9
St3gal2	-1.9
C5ar1	-1.9
Cebpe	-1.9
Rgs18	-1.9
Sh3yl1	-1.9
Clec5a	-1.9
Siglec8	-1.9
Cd9	-1.9
Ube2l6	-1.9
Ccl9	-1.9
Mx2	-1.9
Pak1	-1.9
LOC108348047	-1.9
Sh2d5	-1.9
Gpnmb	-1.9
Sqrdl	-1.9
Dhrs3	-1.9
Marcks	-1.9
Ptgfrn	-1.9
Tesc	-1.9
Gramd3	-1.9

Naaa	-1.9
Maob	-1.9
Pld4	-1.9
Stau2	-1.9
LOC100360575_1	-1.9
Cd300a	-1.9
Lyl1	-1.9
LOC100911825	-1.9
Ncf4	-1.9
LOC100909879	-1.9
Unc93b1	-1.9
Ctla4	-1.9
Alox5	-1.9
Arhgap22	-1.9
C1rl	-1.9
Clec4a1	-1.9
Nr4a1	-1.9
Usp18	-1.9
Sprydd7	-1.9
Acy3	-1.9
Aqp9	-1.9
Lipg	-1.9
Olfm1	-1.9
Stom	-1.9
Hgf	-1.9
P2rx1	-1.9
Stard8	-1.9
Cadm3	-1.9
Hebp1	-1.9
Ccl6	-1.9
Lgmn	-1.9
Kmo	-1.9
Clec7a	-1.9
Dmxl2	-1.9
Gng10	-1.9
Gch1	-1.9
Junb	-1.9
Mcpt8	-1.9
LOC108348072	-2.0
S100a6	-2.0
Grn	-2.0
Tpd52	-2.0
Cdc42bpb	-2.0
Csf2rb	-2.0
Pdlim1	-2.0
Naprt	-2.0
Ms4a2	-2.0

Ccr1	-2.0
Hsd17b1	-2.0
Nrg2	-2.0
Lilrb3l	-2.0
Tspan8	-2.0
Lilrb3a	-2.0
Akr1b10	-2.0
Crip2	-2.0
Il13ra1	-2.0
Dgat2	-2.0
Snta1	-2.0
Zc2hc1a	-2.0
Apobec1	-2.0
Rflna	-2.0
Plbd1	-2.0
Rhoc	-2.0
Ctsh	-2.0
Ltbr	-2.0
Qrfpr	-2.0
Metrnl	-2.0
Mboat1	-2.0
Anxa5	-2.0
Pcsk1	-2.0
Morc1	-2.0
Mtus1	-2.0
Dnajc22	-2.0
Nfil3	-2.0
Fth1	-2.0
Wipi1	-2.0
Itgb5	-2.0
Mcpt1l4	-2.0
Spic	-2.0
Tlr3	-2.0
Ctnnbip1	-2.0
LOC100911851	-2.0
Prodh1	-2.0
Palm	-2.0
Plp1	-2.0
Megf8	-2.0
Gadd45a	-2.0
Emb	-2.0
Mcpt8l2	-2.0
Olr292_1	-2.0
Hlx	-2.0
P3h3	-2.0
Ube2e2	-2.0
Hacd4	-2.0

Pla2g7	-2.0
Cebpd	-2.0
Tcn2	-2.0
Ifit2	-2.1
LOC100911516	-2.1
Card10	-2.1
Il3ra	-2.1
Hbe1	-2.1
Jun	-2.1
C1r	-2.1
Gcsam	-2.1
Il36b	-2.1
Sox13	-2.1
Cd86	-2.1
Tnfaip8l3	-2.1
Mctp1	-2.1
Pkdcc	-2.1
Rab32	-2.1
Ptpn13	-2.1
Cd302	-2.1
Adam9	-2.1
Olr1	-2.1
Rsad2	-2.1
Gchfr	-2.1
Pram1	-2.1
Nt5e	-2.1
RGD1559662	-2.1
Cd63	-2.1
Mapk15	-2.1
Lyplal1	-2.1
Map1lc3a	-2.1
Car1	-2.1
Tmem51	-2.1
Thy1	-2.1
Cadm1	-2.1
Arhgap18	-2.1
Cd33	-2.1
Clec4a2	-2.1
Il1r2	-2.1
Apoe	-2.1
Oscar	-2.1
Mgst1	-2.1
Sh3bp4	-2.1
Gas2l1	-2.1
Degs2	-2.1
Aldh3b1	-2.1
P2ry1	-2.1

Paqr6	-2.2
Oasl	-2.2
Nr0b2	-2.2
LOC56764	-2.2
Irf7	-2.2
LOC103689942	-2.2
Ddah1	-2.2
Cebpb	-2.2
Id1	-2.2
Golga7b	-2.2
Dok4	-2.2
Ccrl2	-2.2
Cebpa	-2.2
Smpd3	-2.2
Pparg	-2.2
LOC102556085	-2.2
Isg15	-2.2
Kcne5	-2.2
Cttn_2	-2.2
Ctdspl	-2.2
Ggn	-2.2
Cldn4	-2.2
Car8	-2.2
AABR07052529.1	-2.2
Kank1	-2.2
Fosb	-2.2
Marveld1	-2.2
Anxa1	-2.2
Rusc2	-2.2
Nfe2	-2.2
Adssl1	-2.2
Gpr141	-2.2
Arhgef10	-2.2
Abcg2	-2.2
Tsc22d1	-2.3
Rpl3l	-2.3
Fxyd2	-2.3
Fam149a	-2.3
Hcar2	-2.3
Lgals3	-2.3
Tyro3	-2.3
Rab44	-2.3
AABR07062800.1	-2.3
Ccdc92	-2.3
Ifit3	-2.3
Tal1	-2.3
Amigo2	-2.3

Alox5ap	-2.3
Crim1	-2.3
Adgra2	-2.3
Opn3	-2.3
Clic2	-2.3
Ccdc80	-2.3
LOC100910801	-2.3
Mrvi1	-2.3
Gzmbl3	-2.3
Mgst2	-2.3
G0s2	-2.4
Steap3	-2.4
RGD1311575	-2.4
Numbl	-2.4
Lrrc49	-2.4
Gp1bb_1	-2.4
Scarf1	-2.4
Nacc2	-2.4
Gucy1b3	-2.4
Large1	-2.4
Osm	-2.4
Apcdd1	-2.4
Acer2	-2.4
Ces2c	-2.4
Bfsp1	-2.4
Slc27a3	-2.4
Gfi1b	-2.4
B3gnt8	-2.4
Rassf6	-2.4
Arpin	-2.4
Socs3	-2.4
Loxl2	-2.4
Rab7b	-2.4
Kit	-2.4
Dab2ip	-2.4
Col16a1	-2.5
Spry4	-2.5
Slc40a1_2	-2.5
Slfn14	-2.5
RGD1307182	-2.5
Dusp1	-2.5
Trim55	-2.5
LOC679149	-2.5
AC117058.1	-2.5
LOC103691261	-2.5
Padi2	-2.5
Cpne4	-2.5

Gpr34	-2.5
Enkur	-2.5
Dennd2c	-2.5
Peli2	-2.5
Adap2	-2.5
Ascl2	-2.5
Adcy5	-2.5
Adora3	-2.5
Aatk	-2.5
F2rl2	-2.5
Pald1	-2.5
Nmnat3	-2.5
Tspan9	-2.5
Thbs1	-2.5
Plvap	-2.6
RGD1561149	-2.6
Esam	-2.6
Ceacam4	-2.6
Arsi	-2.6
Batf3	-2.6
Epha2	-2.6
Tmem246	-2.6
Lpar1	-2.6
Dbn1	-2.6
Glis2	-2.6
Lpar3	-2.6
Fam184a	-2.6
Bgn	-2.6
LOC689574	-2.7
Cttnbp2nl	-2.7
Fam131c	-2.7
Cst3	-2.7
Ap3b2	-2.7
G6b	-2.7
Eepd1	-2.7
LOC103689927	-2.7
Gsdma	-2.7
Snx24	-2.7
Trim2	-2.7
AABR07071392.1	-2.7
Rarb	-2.7
Cela1	-2.7
Tubb2b	-2.7
AABR07053483.1	-2.7
Kcng2	-2.7
Plod2	-2.7
AABR07016640.1	-2.7

Sema4c	-2.7
Mmrn1	-2.7
Retnlg	-2.7
Mpl	-2.7
Procr	-2.7
Heph	-2.7
P2ry12	-2.7
Gmpr	-2.8
Prtfdc1	-2.8
Gp1ba	-2.8
Chst13	-2.8
Ccser1	-2.8
Gdf3	-2.8
Gdf10	-2.8
Kcnab1	-2.8
AABR07035955.1	-2.8
Mylk	-2.8
Pde3a	-2.8
Serpinb6	-2.8
Slc18a2	-2.8
Ddr1	-2.8
Ly6al	-2.8
Tlr11	-2.8
Me1	-2.9
Cpne8	-2.9
Clec4b2	-2.9
RGD1564129	-2.9
Alox12e	-2.9
Rhag	-2.9
Gsta1	-2.9
Fads1	-2.9
Tubb1	-2.9
Efhd1	-2.9
Rhpn2	-2.9
Angptl4	-2.9
Msh5	-2.9
Gpx3	-2.9
Slc6a4	-2.9
Trpc6	-2.9
AABR07061068.2	-2.9
Tubb2a	-2.9
Angpt2	-2.9
Micall2	-2.9
LOC691695	-2.9
Zfp503	-2.9
Smim5	-3.0
Gpr157	-3.0

Plxna2	-3.0
Sardh	-3.0
Flt3	-3.0
Slc22a23	-3.0
Cald1	-3.0
Ltb4r	-3.0
Htra3	-3.0
Nxph3	-3.0
Lonrf3	-3.0
Myh2	-3.0
Cbr3	-3.0
Dydc1	-3.0
Gdf1	-3.0
Rpp25	-3.0
Bco1	-3.0
Nbl1	-3.0
Pam	-3.0
Hs3st3a1	-3.0
Nxph4	-3.0
Ptgr1	-3.0
Clec4f	-3.0
Fos	-3.0
Alpl	-3.0
Nrgn	-3.0
Tmem37	-3.1
Zdhhc1	-3.1
RGD1566368	-3.1
Scamp5	-3.1
Myct1	-3.1
Gdpd2	-3.1
LOC108348091	-3.1
Tspan4	-3.1
Defb14	-3.1
Tgm1	-3.1
Ptgs2	-3.1
Ifitm3	-3.1
Clec4a	-3.1
Tpd52l1	-3.1
AABR07003304.2	-3.2
RGD1305807	-3.2
Clec2l	-3.2
Cnih2	-3.2
Cdh5	-3.2
Rab38	-3.2
Tmem117	-3.2
LOC691716	-3.2
Muc20	-3.2

Myl9	-3.2
Camk1	-3.2
Xcr1	-3.2
Liph	-3.2
Dixdc1	-3.2
Wtip	-3.2
Gpm6b	-3.2
Rn50_20_0026.4	-3.2
Clu	-3.2
Vegfc	-3.2
Oas2	-3.2
Sash1	-3.3
LOC100912228	-3.3
Filip1	-3.3
Mrc1	-3.3
Calml4	-3.3
Pdgfa	-3.3
Gab1	-3.3
Cxxc4	-3.3
Abcd2	-3.3
AABR07053483.2	-3.3
Adamts12	-3.3
Lgalsl	-3.3
Car13	-3.3
Rhbdl3	-3.3
Alox12	-3.4
Reep2	-3.4
Bglap	-3.4
NEWGENE_2724	-3.4
Ccl17	-3.4
Gpr4	-3.4
Pla2g2a	-3.4
Pla1a	-3.4
Ephb2	-3.4
Dynlrb2	-3.4
LOC100910650	-3.4
Ophn1	-3.4
Sestd1	-3.4
Neurl1	-3.4
Agrp	-3.4
Tlr12	-3.4
Fam135a	-3.4
Lamb2	-3.4
Dnase1l3	-3.4
Clec9a	-3.4
Ms4a4a	-3.4
Itga2b	-3.4

Cldn7	-3.4
Tnni1	-3.4
LOC100912210	-3.4
LOC498368	-3.4
B3galnt1	-3.5
Ak9	-3.5
Afdn	-3.5
Bambi	-3.5
F11r	-3.5
Lhfpl2	-3.5
Matn2	-3.5
Sertm1	-3.5
Ppbp	-3.6
Tspan12	-3.6
Slc2a4	-3.6
RGD1565048	-3.6
AABR07006278.1	-3.6
Siglec1	-3.6
AABR07016856.1	-3.6
Kcns3	-3.6
Syt5	-3.6
Ly6g6f	-3.6
Adamts2	-3.6
Ephb4	-3.6
Dnah8	-3.7
Kcnk13	-3.7
Arhgap29	-3.7
Cd34	-3.7
Slpi	-3.7
Fhl1	-3.7
Pf4	-3.7
F13a1	-3.8
Serpinb11	-3.8
Morc4	-3.8
Gp6	-3.8
Msantd3	-3.8
Cbln2	-3.8
Sdk2	-3.8
Gng11	-3.8
Nrcam	-3.8
Ccl22	-3.9
Cyr61	-3.9
Gp9	-3.9
Cntnap2	-3.9
Ier5l	-3.9
Clec1b	-3.9
Trim16	-3.9

Ly6g6c	-4.0
Treml1	-4.0
Cndp1	-4.0
Lrig3	-4.0
Abo	-4.0
Wwtr1	-4.0
Shtn1	-4.0
Clca2	-4.0
AABR07044820.1	-4.1
Slpil2	-4.1
Abca17	-4.1
Marc1	-4.1
Timp3	-4.2
Ccdc149	-4.2
Bean1	-4.2
Dner	-4.2
Duox1	-4.2
Mb21d2	-4.2
Gp1bb_2	-4.2
RGD1565536	-4.3
Myo5b	-4.3
Sftpd	-4.3
Edar	-4.3
Ppp1r14a	-4.4
Adam23	-4.4
Sdpr	-4.4
Grem1	-4.5
Vgf	-4.5
Lhfp	-4.5
Fgfr1	-4.5
AABR07049769.1	-4.5
AABR07058788.4	-4.5
Slc24a4	-4.6
Nrxn2	-4.6
Gpr31	-4.6
Bcam	-4.6
Lgals2	-4.6
Far2	-4.6
Fabp4	-4.7
Dchs1	-4.7
Pigz_2	-4.7
Hgd	-4.8
AABR07052504.1	-4.8
Fam13a	-4.8
Tgfb3	-4.8
Igf1	-4.9
Igfbp5	-4.9

Folr2	-5.0
Homer1	-5.0
Rn60_20_0030.4	-5.1
Pigz_1	-5.1
Ak7	-5.1
Spetex-2F	-5.2
Elane	-5.3
Asgr2	-5.3
Sh2d4a	-5.4
Gpr39	-5.4
Lyve1	-5.5
Npnt	-5.5
F10	-5.5
Gprc5b	-5.6
Lpar4	-5.6
Bpifa6	-5.6
Fam3b	-5.6
Col4a1	-5.6
Klhl13	-5.6
Sema3c	-5.7
Timd4	-5.7
Tnni2	-5.7
Gas6	-5.9
Arhgef5	-5.9
Nrn1	-6.0
Olfml2a	-6.0
Dagla	-6.1
Nectin2	-6.2
Erg	-6.2
Tcam1	-6.5
Duoxa1	-6.6
Ltc4s	-6.7
Pik3c2g	-6.8
Cacna1c	-6.8
Atoh8	-6.9
Tcf15	-7.0
C1qb	-7.1
Cxcl3	-7.3
Ltbp3	-7.4
C1qc	-7.5
Frk	-7.5
C1qa	-7.5
AABR07020815.1	-7.6
Atp6v1g2	-7.6
Ednrb	-7.6
Apoc3	-7.8
Fcrl2	-8.0

Mamstr		-8.8
Efs		-8.8
Grik3		-9.6
LOC100910163		-9.8
Cntn1		-9.9
Cfap57		-10.0
Gdf15		-10.3
Aldh1b1		-11.0
Sptbn2		-11.4
Cxcl1		-12.5
Igfbp6		-12.6
Ceacam6		-12.9
Wnt4		-13.7
Cd163		-14.2
Sost		-15.4
Fam46b		-19.6
Mmp2		-57.0
Nfs1_2		-63.8
Cracr2b		-69.0
Rps4x_1		-133.6
Dcun1d2		-141.3
Rpl35al1		-410.7
Slc25a24	-1.5	
Calcrl	-1.5	
Pqlc3	-1.6	
Fpr2	-1.7	
Ybx3	-1.7	
Fam46a	-1.8	
Faxdc2	-1.9	
Nup210l	-1.9	
Fech	-2.0	
Gas7	-2.0	
Hrh3	-2.1	
Adgre4	-2.1	
AABR07000658.1	-2.1	
Ndst1	-2.3	
RGD1310507	-2.4	
Sptb	-2.5	
Prrg1	-2.7	
Add2	-2.7	
Apba1	-2.7	
Slc38a5	-2.7	
Rhd	-2.8	
Plekhb1	-2.8	
Depdc7	-2.8	
Hbq1b	-2.9	
Hemgn	-2.9	

Aqp1	-2.9
Kel	-3.0
Slc4a1	-3.0
Cav1	-3.4
Arhgef37	-3.5
Angptl2	-3.8
Cysltr1	-4.1
Egfr	-4.3
Map2k3	-4.5
LOC108348079	-5.4
Mmp14	-7.4
Ptgis	-7.6
Asz1	-8.6
Mast4	-99.4
Faim_1	-216.2
Polr2l	-401.3
