# Supplementary material for: Transcriptional analyses of peripheral blood lymphocytes obtained shortly after primary immunization discriminate vaccines against Francisella tularensis
Source: Infect Immun. 2026 Aug 14;94(9):e00208-26. doi: 10.1128/iai.00208-26 (PMC13552110; doi:10.1128/iai.00208-26)
Supplement: Supplementary file 5 [file iai.00208-26-s0005.pdf]

Table S3. Pathways differentially activated after vaccination by Ingenuity pathway analyses. Canonical pathways that may be activated or inhibited after vaccination were filtered as described in Fig. 5, and at each time point and vaccine group pathways were sorted by z-score. Shown also are the ratios, indicating the number of genes that overlap the pathway (1 = 100%), and the -log p-value.

Day 3: LVS vs PBS

Ingenuity Canonical Pathways	-log(p-value)	Ratio	z-score
Cell Cycle Checkpoints	39.3	0.28	8.72
Mitotic Metaphase and Anaphase	27.7	0.25	7.68
Mitotic Prometaphase	29.4	0.28	7.55
RHO GTPases Activate Formins	28.4	0.34	6.56
Neutrophil degranulation	11.2	0.12	5.70
Synthesis of DNA	15.1	0.25	5.57
Mitotic G2-G2/M phases	9.08	0.16	5.57
Class I MHC mediated antigen processing and presentation	4.53	0.09	5.24
HDR through Homologous Recombination (HRR) or Single Strand Annealing (SSA)	9.95	0.21	4.90
RHO GTPase cycle	6.25	0.10	4.82
IL-27 Signaling Pathway	9.89	0.19	4.60
Activation of the pre-replicative complex	16.6	0.54	4.36
Cell Cycle Control of Chromosomal Replication	12.9	0.35	4.36
Nuclear Cytoskeleton Signaling Pathway	7.45	0.14	4.27
Deubiquitination	2.52	0.08	4.26
Regulation of mitotic cell cycle	7.43	0.21	4.24
DNA Replication Pre-Initiation	5.6	0.16	4.12
Intra-Golgi and retrograde Golgi-to-ER traffic	4.51	0.11	3.96
Kinesins	11.1	0.31	3.90
Nucleosome assembly	8.86	0.31	3.87
Pathogen Induced Cytokine Storm Signaling Pathway	16	0.15	3.78
Kinetochore Metaphase Signaling Pathway	28.2	0.40	3.65
TP53 Regulates Transcription of Cell Cycle Genes	6.94	0.27	3.61
S Phase	3.96	0.14	3.61
Colorectal Cancer Metastasis Signaling	2.73	0.08	3.58
Telomere Maintenance	3.32	0.14	3.46
Mitotic Prophase	2.72	0.12	3.46
NOD1/2 Signaling Pathway	3.09	0.10	3.44
Cohesin Chromatin Regulation Pathway	4.04	0.10	3.41
Interferon alpha/beta signaling	6.08	0.20	3.36
Salvage Pathways of Pyrimidine Ribonucleotides	2.87	0.12	3.32
Neuroinflammation Signaling Pathway	8.76	0.12	3.29
Cachexia Signaling Pathway	2.49	0.08	3.27
Immunogenic Cell Death Signaling Pathway	6.17	0.18	3.21
MHC class II antigen presentation	8.15	0.18	3.13
IL-12 Signaling and Production in Macrophages	4.21	0.10	3.13
Interleukin-1 family signaling	2.38	0.10	3.05
Interconversion of nucleotide di- and triphosphates	5.6	0.31	3.00
DNA Damage Bypass	3.7	0.19	3.00
Chronic Myeloid Leukemia Signaling	3.21	0.09	3.00
NLR signaling pathways	3.18	0.16	3.00
Interferon gamma signaling	7.4	0.19	2.98

Regulation of TP53 Activity through Phosphorylation	3.15	0.13	2.89
TREM1 Signaling	6.61	0.20	2.84
ID1 Signaling Pathway	3.67	0.10	2.84
TAK1-dependent IKK and NF-kappa-B activation	3.26	0.18	2.83
Pyridoxal 5'-phosphate Salvage Pathway	2.66	0.14	2.83
Cyclins and Cell Cycle Regulation	4.7	0.16	2.71
ISGylation Signaling Pathway	9.59	0.21	2.71
L1CAM interactions	2.99	0.11	2.67
Ribonucleotide Reductase Signaling Pathway	2.79	0.10	2.67
NER (Nucleotide Excision Repair, Enhanced Pathway)	2.7	0.12	2.65
SUMOylation of DNA replication proteins	2.45	0.15	2.65
Meiotic recombination	2.25	0.14	2.65
Activin Inhibin Signaling Pathway	2.61	0.09	2.52
Role of Pattern Recognition Receptors in Recognition of Bacteria and Viruses	3.86	0.12	2.50
Multiple Sclerosis Signaling Pathway	11.2	0.16	2.48
Pyrimidine Deoxyribonucleotides De Novo Biosynthesis I	3.13	0.23	2.45
RHO GTPases activate IQGAPs	2.64	0.19	2.45
Toll-like Receptor Signaling	2.21	0.12	2.45
Estrogen-mediated S-phase Entry	7.18	0.39	2.33
Interferon Signaling	4.65	0.24	2.33
Apoptotic execution phase	3.43	0.17	2.33
ISG15 antiviral mechanism	2.32	0.12	2.33
Pyroptosis Signaling Pathway	2.31	0.11	2.33
KEAP1-NFE2L2 pathway	2.57	0.10	2.32
Natural Killer Cell Signaling	2.11	0.08	2.32
PKR-mediated signaling	3.87	0.15	2.31
iNOS Signaling	2.29	0.14	2.24
Transcriptional regulation by RUNX2	2.2	0.14	2.24
Signaling by VEGF	3.64	0.13	2.14
Role of PKR in Interferon Induction and Antiviral Response	2.99	0.11	2.14
G alpha (i) signalling events	6.96	0.12	2.12
Toll-like Receptor Cascades	7.25	0.34	2.11
Interleukin-4 and Interleukin-13 signaling	6.51	0.17	2.07
Signaling by Rho Family GTPases	2.81	0.09	2.07
Tumoricidal Function of Hepatic Natural Killer Cells	2.48	0.21	2.00
Caspase activation via Death Receptors in the presence of ligand	2.37	0.25	2.00
Mismatch Repair	2.37	0.25	2.00
Superpathway of Citrulline Metabolism	2.37	0.25	2.00
Mismatch Repair in Eukaryotes	2.09	0.21	2.00
Signaling by Hippo	2	0.20	2.00
Serotonin Receptor Signaling	3.09	0.08	1.92
S100 Family Signaling Pathway	4.49	0.08	1.91
Death Receptor Signaling	2.01	0.10	1.90
Pancreatic Adenocarcinoma Signaling	3.96	0.13	1.89
Resolution of Abasic Sites (AP sites)	2.95	0.18	1.89
Gap junction trafficking and regulation	2.2	0.14	1.89
Glycosaminoglycan metabolism	3.33	0.12	1.81
Antigen Presentation Pathway	12	0.40	1.73
Post-translational protein phosphorylation	2.62	0.11	1.73

Regulation of Insulin-like Growth Factor (IGF) transport and uptake by IGFBPs	2.1	0.10	1.73
Irritable Bowel Syndrome Signaling Pathway	4.55	0.10	1.67
Crosstalk between Dendritic Cells and Natural Killer Cells	5.3	0.16	1.67
Signaling by PTK6	3.24	0.16	1.67
Ribavirin ADME	2.62	0.13	1.67
Zn Homeostasis Signaling Pathway	2.2	0.06	1.64
Macrophage Classical Activation Signaling Pathway	15.5	0.21	1.62
Signaling by NOTCH4	2.43	0.10	1.60
Mitotic Roles of Polo-Like Kinase	7.68	0.24	1.51
Role of JAK family kinases in IL-6-type Cytokine Signaling	3.12	0.14	1.51
Hepatitis B Chronic Liver Pathogenesis Signaling Pathway	2.82	0.10	1.50
Cell Cycle: G1/S Checkpoint Regulation	4.41	0.17	-1.51
Tuberculosis Active Signaling Pathway	5.95	0.12	-1.51
Mitotic G1 phase and G1/S transition	15.8	0.25	-1.67
Coronavirus Pathogenesis Pathway	3.42	0.10	-1.79
Transcriptional Regulation by E2F6	3.25	0.21	-1.89
Th2 Pathway	8.68	0.18	-2.36

Day 3: ΔclpB vs. PBS

Ingenuity Canonical Pathways	-log(p-value)	Ratio	z-score
Neutrophil degranulation	34.9	0.26	9.03
Mitotic Metaphase and Anaphase	12.7	0.23	7.08
Cell Cycle Checkpoints	13.9	0.22	7.04
Class I MHC mediated antigen processing and presentation	8.76	0.17	6.25
Mitotic Prometaphase	9.03	0.21	5.95
Mitotic G2-G2/M phases	3.92	0.16	5.57
RHO GTPases Activate Formins	10.9	0.26	5.10
Interferon alpha/beta signaling	10.6	0.34	4.71
Synthesis of DNA	5.34	0.21	4.60
Regulation of mitotic cell cycle	5.69	0.24	4.58
DNA Replication Pre-Initiation	3.53	0.18	4.36
Kinesins	6.3	0.29	4.24
Nuclear Cytoskeleton Signaling Pathway	3.81	0.15	4.24
Deubiquitination	2.99	0.13	4.23
Interleukin-1 family signaling	6.99	0.23	4.16
Interferon gamma signaling	8.57	0.28	4.04
Oxidative Phosphorylation	2.12	0.15	4.00
Detoxification of Reactive Oxygen Species	7.52	0.41	3.87
Respiratory electron transport	2.6	0.17	3.87
Metabolism of polyamines	3.97	0.24	3.74
Cellular response to hypoxia	2.79	0.18	3.74
S Phase	5.3	0.22	3.71
IL-27 Signaling Pathway	16.6	0.33	3.70
Protein folding	2.9	0.17	3.64
Cytoprotection by HMOX1	4.06	0.24	3.61
Hedgehog ligand biogenesis	2.97	0.20	3.61
Protein Ubiquitination Pathway	2.1	0.12	3.53
Regulation of Apoptosis	3.28	0.23	3.46
Regulation of RUNX2 expression and activity	2.55	0.18	3.46
Nucleosome assembly	3.02	0.22	3.32
NIK-->noncanonical NF-kB signaling	2.29	0.18	3.32
Neutrophil Extracellular Trap Signaling Pathway	6.12	0.15	3.31
Cachexia Signaling Pathway	14.5	0.20	3.30
Role of Hypercytokinemia/hyperchemokinemia in the Pathogenesis of Influenza	5.95	0.24	3.30
TNFR2 non-canonical NF-kB pathway	5.66	0.27	3.30
Death Receptor Signaling	5.53	0.23	3.27
iNOS Signaling	3.02	0.22	3.16
TAK1-dependent IKK and NF-kappa-B activation	2.84	0.23	3.16
ISGylation Signaling Pathway	8.76	0.27	3.16
MHC class II antigen presentation	7.07	0.23	3.16
Immunogenic Cell Death Signaling Pathway	6.91	0.25	3.13
Interferon Signaling	6.62	0.38	3.05
Hedgehog 'off' state	3.89	0.18	3.05
Glucose metabolism	3.15	0.19	3.00
Somitogenesis	3.13	0.22	2.89
KEAP1-NFE2L2 pathway	3.29	0.16	2.86
Antigen Presentation Pathway	11.2	0.47	2.84

TP53 Regulates Metabolic Genes	2.75	0.18	2.84
Transcriptional regulation by RUNX1	2.24	0.14	2.84
Pyroptosis	3.18	0.30	2.83
Interconversion of nucleotide di- and triphosphates	2.95	0.28	2.83
Signaling by CSF3 (G-CSF)	2.84	0.27	2.83
RIPK1-mediated regulated necrosis	2.65	0.25	2.83
RHO GTPases activate IQGAPs	2.65	0.25	2.83
PTEN Regulation	2.64	0.15	2.83
TREM1 Signaling	11.8	0.35	2.75
Retinoic acid Mediated Apoptosis Signaling	2.23	0.18	2.71
Signaling by NOTCH4	3.34	0.16	2.71
PKR-mediated signaling	2.67	0.18	2.67
Iron uptake and transport	5.82	0.28	2.67
Microautophagy Signaling Pathway	2.33	0.14	2.67
Role of Chondrocytes in Rheumatoid Arthritis Signaling Pathway	5.93	0.20	2.65
NFE2L2 regulating anti-oxidant/detoxification enzymes	2.92	0.30	2.65
RHO GTPases Activate NADPH Oxidases	2.8	0.29	2.65
Neuroinflammation Signaling Pathway	17.8	0.23	2.63
Pathogen Induced Cytokine Storm Signaling Pathway	25.4	0.25	2.56
Irritable Bowel Syndrome Signaling Pathway	7.41	0.17	2.55
IL-12 Signaling and Production in Macrophages	9.12	0.20	2.53
Toll-like Receptor Cascades	4.06	0.31	2.53
Activation of the pre-replicative complex	3.7	0.29	2.53
Degradation of beta-catenin by the destruction complex	3.82	0.20	2.50
NLR signaling pathways	3.63	0.23	2.50
Production of Nitric Oxide and Reactive Oxygen Species in Macrophages	5.49	0.18	2.48
Pyrimidine Deoxyribonucleotides De Novo Biosynthesis I	2.57	0.27	2.45
Regulation of TLR by endogenous ligand	2.3	0.27	2.45
Chaperone Mediated Autophagy	2.3	0.27	2.45
Nucleotide salvage	2.2	0.26	2.45
Role of PKR in Interferon Induction and Antiviral Response	3.12	0.16	2.40
IL-17A Signaling in Fibroblasts	4.59	0.23	2.36
Fcγ Receptor-mediated Phagocytosis in Macrophages and Monocytes	4.2	0.20	2.36
HEY1 Signaling Pathway	3.67	0.16	2.35
Growth hormone receptor signaling	4.25	0.36	2.33
Metabolism of porphyrins	4.1	0.35	2.33
MIF Regulation of Innate Immunity	2.68	0.22	2.33
Signaling by Insulin receptor	2.21	0.20	2.33
Regulation of mRNA stability by proteins that bind AU-rich elements	2.55	0.17	2.32
Toll-like Receptor Signaling	3.72	0.21	2.31
Cyclins and Cell Cycle Regulation	3.64	0.20	2.31
Kinetochore Metaphase Signaling Pathway	11.6	0.31	2.29
Hedgehog 'on' state	3.25	0.16	2.29
Sertoli Cell-Sertoli Cell Junction Signaling	2.94	0.13	2.26
Warburg Effect Signaling Pathway	3.92	0.18	2.24
Heme Biosynthesis II	3.61	0.56	2.24
Caspase activation via Death Receptors in the presence of ligand	2.26	0.31	2.24
NOD1/2 Signaling Pathway	6.8	0.19	2.20
Orexin Signaling Pathway	4.42	0.15	2.20

Role of MAPK Signaling in Promoting the Pathogenesis of Influenza	3.89	0.18	2.18
Pyroptosis Signaling Pathway	3.18	0.18	2.14
RHO GTPase cycle	8.81	0.16	2.12
Glycolysis I	4.48	0.35	2.12
Surfactant metabolism	2.65	0.25	2.12
RHO GTPases Activate WASPs and WAVEs	3.54	0.26	2.11
Colorectal Cancer Metastasis Signaling	7.04	0.17	2.08
Fcgamma receptor (FCGR) dependent phagocytosis	2.48	0.14	2.07
CGAS-STING Signaling Pathway	9.8	0.25	2.06
Tumor Microenvironment Pathway	4	0.16	2.04
Role of Macrophages, Fibroblasts and Endothelial Cells in Rheumatoid Arthritis	11.1	0.19	2.00
FAT10 Signaling Pathway	3.63	0.23	2.00
Salvage Pathways of Pyrimidine Ribonucleotides	3.29	0.18	2.00
Erythrocytes take up oxygen and release carbon dioxide	3.01	0.57	2.00
ATF6 (ATF6-alpha) activates chaperone genes	2.31	0.40	2.00
Superpathway of Citrulline Metabolism	2.26	0.31	2.00
Erythrocytes take up carbon dioxide and release oxygen	2.14	0.36	2.00
Tumoricidal Function of Hepatic Natural Killer Cells	2.1	0.25	2.00
γ-glutamyl Cycle	2	0.33	2.00
Multiple Sclerosis Signaling Pathway	19.4	0.28	1.99
Ephrin A Signaling	2.67	0.15	1.96
Preeclampsia Signaling Pathway	3.67	0.16	1.96
Agrin Interactions at Neuromuscular Junction	3.28	0.21	1.94
Unfolded protein response	2.41	0.16	1.94
Epithelial Adherens Junction Signaling	5.09	0.18	1.89
MIF-mediated Glucocorticoid Regulation	2.16	0.21	1.89
Pulmonary Healing Signaling Pathway	7.76	0.20	1.81
MITF-M-dependent gene expression	7.55	0.26	1.80
L1CAM interactions	5.21	0.20	1.80
Role of Pattern Recognition Receptors in Recognition of Bacteria and Viruses	10.8	0.25	1.79
Acute Phase Response Signaling	5.54	0.18	1.79
GM-CSF Signaling	3.68	0.21	1.73
Ribonucleotide Reductase Signaling Pathway	3.5	0.16	1.73
Activation of IRF by Cytosolic Pattern Recognition Receptors	2.42	0.18	1.73
G alpha (i) signalling events	5.62	0.16	1.72
Interleukin-10 signaling	7.89	0.38	1.70
Signaling by PTK6	6.42	0.31	1.70
PI3K/AKT Signaling	5.51	0.18	1.70
Binding and Uptake of Ligands by Scavenger Receptors	2.24	0.15	1.70
Macrophage Classical Activation Signaling Pathway	19.5	0.30	1.70
Ion channel transport	3.88	0.16	1.67
Estrogen-mediated S-phase Entry	4.1	0.35	1.67
Nucleotide catabolism	3.01	0.26	1.67
Necroptosis Signaling Pathway	3.98	0.17	1.63
Airway Pathology in Chronic Obstructive Pulmonary Disease	3.44	0.18	1.63
Endoplasmic Reticulum Stress Pathway	3.18	0.33	1.63
Gluconeogenesis I	2.84	0.27	1.63
Pancreatic Adenocarcinoma Signaling	9.14	0.26	1.61
EPH-Ephrin signaling	4.2	0.20	1.61

Induction of Apoptosis by HIV1	3.98	0.23	1.60
Platelet homeostasis	2.27	0.16	1.60
Role of Osteoclasts in Rheumatoid Arthritis Signaling Pathway	5.41	0.15	1.58
Glycerophospholipid biosynthesis	3.18	0.16	1.53
Response to elevated platelet cytosolic Ca2+	6.22	0.21	1.51
Nicotinate metabolism	5.01	0.36	1.51
Signaling by SCF-KIT	3.54	0.26	1.51
TNFR1 Signaling	3.36	0.23	1.51
Folate Signaling Pathway	2.46	0.19	1.51
Type II Diabetes Mellitus Signaling	2.24	0.14	1.51
Antioxidant Action of Vitamin C	6.24	0.22	-1.50
Cell Cycle: G1/S Checkpoint Regulation	5.56	0.26	-1.50
Extra-nuclear estrogen signaling	3.86	0.21	-1.50
BEX2 Signaling Pathway	3.39	0.20	-1.50
Acute Myeloid Leukemia Signaling	4.8	0.22	-1.51
Cardiac β-adrenergic Signaling	2.29	0.14	-1.51
Acetylcholine Receptor Signaling Pathway	3.63	0.15	-1.51
Role of NFAT in Cardiac Hypertrophy	3.2	0.14	-1.57
Myelination Signaling Pathway	2.66	0.12	-1.58
Hereditary Breast Cancer Signaling	2.4	0.15	-1.61
Factors Promoting Cardiogenesis in Vertebrates	4.14	0.17	-1.63
Sperm Motility	5.28	0.16	-1.81
Breast Cancer Regulation by Stathmin1	14.5	0.17	-1.84
Th2 Pathway	10.2	0.26	-1.89
FOXO-mediated transcription of cell cycle genes	3.84	0.41	-1.89
Cell Cycle: G2/M DNA Damage Checkpoint Regulation	2.34	0.20	-1.89
CREB Signaling in Neurons	11.6	0.16	-1.92
LXR/RXR Activation	4.84	0.19	-1.96
PPAR Signaling	2.89	0.17	-2.36
Tuberculosis Active Signaling Pathway	16.8	0.25	-2.58
Parkinson's Signaling Pathway	13.9	0.21	-2.79

Day 7: LVS vs. PBS

Ingenuity Canonical Pathways	-log(p-value)	Ratio	z-score
Mitochondrial RNA degradation	6.5	0.36	3.00
rRNA processing	5.5	0.28	3.00
tRNA processing in the mitochondrion	4.5	0.22	3.00
Respiratory electron transport	2.1	0.10	3.00
Cellular Effects of Sildenafil (Viagra)	9.4	0.09	2.99
PTEN Signaling	3.4	0.11	2.67
CLEAR Signaling Pathway	9.5	0.13	2.33
PPAR Signaling	4.0	0.13	2.14
Antioxidant Action of Vitamin C	3.6	0.12	2.11
Erythrocytes take up oxygen and release carbon dioxide	4.1	0.57	2.00
Erythrocytes take up carbon dioxide and release oxygen	3.2	0.36	2.00
PI3K/AKT Signaling	2.5	0.09	-1.51
Sheddase Signaling Pathway	7.8	0.14	-1.51
Docosahexaenoic Acid (DHA) Signaling	5.0	0.10	-1.57
Immunogenic Cell Death Signaling Pathway	5.9	0.17	-1.60
MSP-RON Signaling in Macrophages Pathway	3.5	0.12	-1.60
EIF2 Signaling	2.1	0.08	-1.60
Cerebral Malformation Signaling Pathway	5.6	0.14	-1.61
G-protein beta:gamma signalling	2.8	0.19	-1.63
Signal amplification	2.7	0.18	-1.63
Coagulation System	2.6	0.17	-1.63
Detoxification of Reactive Oxygen Species	2.5	0.16	-1.63
Transcriptional regulation by RUNX2	2.4	0.14	-1.63
Semaphorin interactions	3.0	0.14	-1.67
Ceramide Signaling	3.0	0.12	-1.67
G alpha (12/13) signalling events	2.3	0.11	-1.67
Sperm Motility	4.8	0.10	-1.73
Th1 Pathway	2.3	0.10	-1.73
Myelination Signaling Pathway	5.4	0.10	-1.77
Erythropoietin Signaling Pathway	3.9	0.11	-1.81
Adipogenesis pathway	3.4	0.11	-1.81
Osteoarthritis Pathway	6.4	0.12	-1.88
Oxytocin in Brain Signaling Pathway	5.2	0.11	-1.88
Opioid Signaling Pathway	5.0	0.10	-1.88
IL-27 Signaling Pathway	5.1	0.13	-1.89
MicroRNA Biogenesis Signaling Pathway	3.3	0.10	-1.89
Immunoregulatory interactions between a Lymphoid and a non-Lymphoid cell	2.6	0.08	-1.89
PFKFB4 Signaling Pathway	2.5	0.14	-1.89
Apoptotic execution phase	2.3	0.14	-1.89
Transcriptional regulation of granulopoiesis	4.5	0.20	-1.90
Regulation of Actin-based Motility by Rho	2.8	0.11	-1.90
Endocannabinoid Developing Neuron Pathway	2.7	0.10	-1.90
p38 MAPK Signaling	4.4	0.13	-1.94
Ferroptosis Signaling Pathway	3.4	0.11	-1.94
NAD Signaling Pathway	2.9	0.10	-1.94
Hereditary Breast Cancer Signaling	2.4	0.09	-1.94
White Adipose Tissue Browning Pathway	2.4	0.09	-1.94

Calcium Transport I	3.4	0.40	-2.00
Necroptosis Signaling Pathway	3.0	0.10	-2.00
Inhibition of Angiogenesis by TSP1	2.7	0.18	-2.00
Gastrin-CREB signalling pathway via PKC and MAPK	2.3	0.22	-2.00
Oxytocin Signaling Pathway	7.8	0.12	-2.06
Sirtuin Signaling Pathway	2.3	0.07	-2.07
Phospholipases	4.8	0.18	-2.11
Chemokine Signaling	3.4	0.14	-2.11
Prostate Cancer Signaling	2.6	0.10	-2.11
IL-15 Production	2.4	0.10	-2.11
Antiproliferative Role of Somatostatin Receptor 2	5.7	0.18	-2.12
Melanoma Signaling	4.6	0.20	-2.12
Formation of Fibrin Clot (Clotting Cascade)	3.9	0.21	-2.12
Oncostatin M Signaling	3.6	0.19	-2.12
ERB2-ERBB3 Signaling	2.4	0.13	-2.12
α-Adrenergic Signaling	2.3	0.10	-2.12
Cardiac conduction	2.2	0.09	-2.12
ERK/MAPK Signaling	5.5	0.11	-2.13
Atherosclerosis Signaling	4.5	0.13	-2.14
Huntington's Disease Signaling	2.2	0.07	-2.14
HOTAIR Regulatory Pathway	2.2	0.08	-2.14
Mitochondrial Division Signaling Pathway	2.1	0.08	-2.14
Proteasomal PSMD10 Signaling Pathway	5.1	0.14	-2.18
Glycerophospholipid biosynthesis	4.8	0.13	-2.18
Chronic Myeloid Leukemia Signaling	5.9	0.11	-2.19
Autophagy	6.1	0.12	-2.20
Leukocyte Extravasation Signaling	4.4	0.11	-2.24
Trafficking and processing of endosomal TLR	4.0	0.39	-2.24
Platelet Adhesion to exposed collagen	3.5	0.31	-2.24
NFE2L2 regulating anti-oxidant/detoxification enzymes	2.7	0.22	-2.24
Syndecan interactions	2.4	0.19	-2.24
CDP-diacylglycerol Biosynthesis I	2.3	0.18	-2.24
Interconversion of nucleotide di- and triphosphates	2.3	0.17	-2.24
Phosphatidylglycerol Biosynthesis II (Non-plastidic)	2.2	0.17	-2.24
Signaling by BMP	2.1	0.16	-2.24
Cancer Drug Resistance by Drug Efflux	2.1	0.12	-2.24
Cachexia Signaling Pathway	4.8	0.09	-2.26
Sertoli Cell-Germ Cell Junction Signaling Pathway (Enhanced)	4.1	0.10	-2.29
Regulation of Cellular Mechanics by Calpain Protease	4.9	0.16	-2.31
FLT3 Signaling in Hematopoietic Progenitor Cells	4.0	0.15	-2.31
Role of MAPK Signaling in Promoting the Pathogenesis of Influenza	3.2	0.11	-2.31
Natural Killer Cell Signaling	2.5	0.08	-2.32
Relaxin Signaling	4.2	0.12	-2.33
Endometrial Cancer Signaling	3.9	0.17	-2.33
Angiopoietin Signaling	3.6	0.14	-2.33
Degradation of the extracellular matrix	2.3	0.11	-2.33
DDX58/IFIH1-mediated induction of interferon-alpha/beta	2.3	0.11	-2.33
Mitochondrial Dysfunction	3.1	0.08	-2.35
Autism Signaling Pathway	3.1	0.08	-2.40

Regulation of TLR by endogenous ligand	3.8	0.27	-2.45
MyD88 dependent cascade initiated on endosome	2.9	0.19	-2.45
Ephrin B Signaling	2.1	0.11	-2.45
Role of Hypercytokinemia/hyperchemokinemia in the Pathogenesis of Influenza	6.1	0.17	-2.50
VEGF Family Ligand-Receptor Interactions	5.1	0.16	-2.50
Thyroid Cancer Signaling	4.9	0.17	-2.50
NRF2-mediated Oxidative Stress Response	4.8	0.10	-2.50
Fc Epsilon RI Signaling	3.0	0.11	-2.50
Factors Promoting Cardiogenesis in Vertebrates	2.9	0.10	-2.50
Synaptic Long Term Depression	3.8	0.10	-2.52
Neuregulin Signaling	4.7	0.14	-2.53
Neurotrophin/TRK Signaling	3.0	0.13	-2.53
JAK/STAT Signaling	2.8	0.12	-2.53
Cell junction organization	2.4	0.11	-2.53
Telomerase Signaling	2.4	0.10	-2.53
Pancreatic Adenocarcinoma Signaling	2.3	0.10	-2.53
Macrophage Classical Activation Signaling Pathway	6.2	0.13	-2.56
MAPK targets/ Nuclear events mediated by MAP kinases	3.6	0.21	-2.65
FAT10 Cancer Signaling Pathway	3.0	0.16	-2.65
RHO GTPases Activate WASPs and WAVEs	2.8	0.16	-2.65
Elastic fibre formation	2.8	0.16	-2.65
Arachidonic acid metabolism	2.7	0.16	-2.65
Signaling by TGF-beta Receptor Complex	2.3	0.13	-2.65
UVB-Induced MAPK Signaling	2.3	0.13	-2.65
ILK Signaling	3.0	0.09	-2.67
Interleukin-10 signaling	8.9	0.31	-2.67
CCR3 Signaling in Eosinophils	5.7	0.14	-2.67
PIP3 activates AKT signaling	2.7	0.10	-2.67
ISGylation Signaling Pathway	10.3	0.21	-2.71
Estrogen-Dependent Breast Cancer Signaling	4.6	0.16	-2.71
ERK5 Signaling	4.4	0.16	-2.71
Gαi Signaling	3.1	0.10	-2.71
PAK Signaling	3.0	0.11	-2.71
VEGF Signaling	2.8	0.11	-2.71
Interferon gamma signaling	2.7	0.11	-2.71
Apelin Cardiomyocyte Signaling Pathway	2.7	0.11	-2.71
Cargo recognition for clathrin-mediated endocytosis	2.5	0.11	-2.71
Renin-Angiotensin Signaling	2.4	0.10	-2.71
Macrophage Alternative Activation Signaling Pathway	8.4	0.15	-2.75
Hepatitis B Chronic Liver Pathogenesis Signaling Pathway	7.4	0.14	-2.75
Estrogen Receptor Signaling	4.6	0.09	-2.75
ID1 Signaling Pathway	10.5	0.16	-2.83
Parkinson's Signaling Pathway	7.2	0.11	-2.83
Toll-like Receptor Cascades	4.5	0.25	-2.83
NLR signaling pathways	2.8	0.14	-2.83
Growth Hormone Signaling	2.6	0.12	-2.83
IL-2 Signaling	2.4	0.13	-2.83
ERBB4 Signaling	2.3	0.12	-2.83
Agrin Interactions at Neuromuscular Junction	2.2	0.12	-2.83

Role of JAK1 and JAK3 in γc Cytokine Signaling	2.2	0.12	-2.83
Orexin Signaling Pathway	3.1	0.09	-2.84
Glioma Invasiveness Signaling	6.9	0.21	-2.84
P2Y Purinergic Receptor Signaling Pathway	5.8	0.14	-2.84
Warburg Effect Signaling Pathway	4.7	0.13	-2.84
Clathrin-mediated endocytosis	3.7	0.12	-2.84
Endothelin-1 Signaling	6.5	0.13	-2.86
Toll-like Receptor Signaling	7.3	0.21	-2.89
TGF-β Signaling	4.5	0.14	-2.89
Platelet homeostasis	3.8	0.14	-2.89
Melanocyte Development and Pigmentation Signaling	3.8	0.13	-2.89
Glioma Signaling	3.7	0.12	-2.89
Mouse Embryonic Stem Cell Pluripotency	3.1	0.12	-2.89
Fcgamma receptor (FCGR) dependent phagocytosis	2.6	0.09	-2.89
RAR Activation	2.8	0.07	-2.92
Interleukin-4 and Interleukin-13 signaling	7.1	0.17	-2.98
Signaling by VEGF	5.2	0.15	-3.00
Gαq Signaling	3.8	0.11	-3.00
UVC-Induced MAPK Signaling	3.8	0.18	-3.00
Unfolded protein response	3.5	0.13	-3.00
CNTF Signaling	3.4	0.16	-3.00
GM-CSF Signaling	3.3	0.14	-3.00
Role of NANOG in Mammalian Embryonic Stem Cell Pluripotency	2.9	0.11	-3.00
Ovarian Cancer Signaling	2.3	0.09	-3.00
Prolactin Signaling	2.3	0.10	-3.00
Non-Small Cell Lung Cancer Signaling	2.3	0.10	-3.00
Integrin cell surface interactions	2.2	0.11	-3.00
IGF-1 Signaling	2.0	0.10	-3.00
Senescence Pathway	5.3	0.10	-3.02
Paxillin Signaling	4.7	0.14	-3.05
GNRH Signaling	2.7	0.09	-3.05
Insulin Receptor Signaling	2.7	0.10	-3.05
Neutrophil Extracellular Trap Signaling Pathway	5.4	0.09	-3.12
MSP-RON Signaling in Cancer Cells Pathway	8.6	0.17	-3.13
Adrenomedullin signaling pathway	6.2	0.12	-3.13
NAFLD Signaling Pathway	4.4	0.10	-3.13
L1CAM interactions	5.0	0.14	-3.15
Ephrin Receptor Signaling	4.2	0.10	-3.15
Role of NFAT in Cardiac Hypertrophy	3.0	0.09	-3.15
SPINK1 General Cancer Pathway	4.2	0.16	-3.16
Thrombopoietin Signaling	3.7	0.16	-3.16
IL-3 Signaling	3.4	0.14	-3.16
GDNF Family Ligand-Receptor Interactions	3.1	0.13	-3.16
Sphingosine-1-phosphate Signaling	3.0	0.11	-3.16
Signaling by MET	2.9	0.13	-3.16
Signaling by NTRK1 (TRKA)	2.8	0.12	-3.16
PDGF Signaling	2.6	0.12	-3.16
Regulation of eIF4 and p70S6K Signaling	2.0	0.08	-3.16
Interferon alpha/beta signaling	5.8	0.18	-3.21

NF-κB Activation by Viruses	5.6	0.18	-3.21
fMLP Signaling in Neutrophils	4.1	0.12	-3.21
NGF Signaling	4.0	0.12	-3.21
TR/RXR Activation	3.6	0.12	-3.21
Ion channel transport	3.4	0.10	-3.30
Actin Nucleation by ARP-WASP Complex	4.2	0.14	-3.32
Role of PKR in Interferon Induction and Antiviral Response	2.4	0.09	-3.32
HEY1 Signaling Pathway	2.7	0.09	-3.36
Transcriptional Regulatory Network in Embryonic Stem Cells	2.6	0.09	-3.36
Wound Healing Signaling Pathway	4.6	0.10	-3.40
RAF/MAP kinase cascade	3.0	0.08	-3.41
Macropinocytosis Signaling	7.3	0.21	-3.46
Renal Cell Carcinoma Signaling	5.6	0.18	-3.46
UVA-Induced MAPK Signaling	5.1	0.15	-3.46
LPS-stimulated MAPK Signaling	4.5	0.15	-3.46
Plasma lipoprotein assembly, remodeling, and clearance	4.3	0.16	-3.46
STAT3 Pathway	3.8	0.12	-3.46
ERBB Signaling	3.5	0.13	-3.46
Fcγ Receptor-mediated Phagocytosis in Macrophages and Monocytes	6.1	0.17	-3.50
Glioblastoma Multiforme Signaling	3.3	0.10	-3.50
Protein Sorting Signaling Pathway	3.0	0.09	-3.50
Multiple Sclerosis Signaling Pathway	7.7	0.13	-3.53
Glycation Signaling Pathway	7.5	0.13	-3.53
Epithelial Membrane Protein Signaling Pathway	8.2	0.15	-3.53
Sertoli Cell-Sertoli Cell Junction Signaling	5.6	0.11	-3.53
G alpha (i) signalling events	3.5	0.09	-3.55
Production of Nitric Oxide and Reactive Oxygen Species in Macrophages	6.1	0.13	-3.58
IL-6 Signaling	3.7	0.12	-3.61
IL-13 Signaling Pathway	2.9	0.11	-3.61
HGF Signaling	6.5	0.15	-3.64
Actin Cytoskeleton Signaling	3.9	0.10	-3.64
Class A/1 (Rhodopsin-like receptors)	4.5	0.09	-3.65
Activin Inhibin Signaling Pathway	10.6	0.16	-3.66
Pancreatic Secretion Signaling Pathway	3.5	0.09	-3.71
Reelin Signaling in Neurons	4.4	0.12	-3.74
Virus Entry via Endocytic Pathways	3.5	0.12	-3.74
Role of Pattern Recognition Receptors in Recognition of Bacteria and Viruses	8.6	0.16	-3.77
Human Embryonic Stem Cell Pluripotency	2.9	0.09	-3.77
Synaptogenesis Signaling Pathway	4.5	0.09	-3.78
14-3-3-mediated Signaling	4.7	0.13	-3.87
HMGB1 Signaling	4.1	0.11	-3.87
Thrombin Signaling	4.0	0.10	-3.87
Role of Chondrocytes in Rheumatoid Arthritis Signaling Pathway	5.3	0.13	-3.90
Regulation of the Epithelial Mesenchymal Transition by Growth Factors Pathway	3.5	0.10	-3.90
Cell surface interactions at the vascular wall	3.0	0.09	-3.90
Role of Osteoclasts in Rheumatoid Arthritis Signaling Pathway	4.9	0.09	-3.92
NOD1/2 Signaling Pathway	5.5	0.12	-3.96
Pyroptosis Signaling Pathway	5.5	0.16	-4.00
Apelin Endothelial Signaling Pathway	5.2	0.13	-4.00

Acute Phase Response Signaling	4.5	0.11	-4.00
Eicosanoid Signaling	9.0	0.13	-4.00
Cardiac Hypertrophy Signaling	3.8	0.09	-4.03
Integrin Signaling	6.4	0.12	-4.08
RAC Signaling	6.9	0.15	-4.12
Cholecystokinin/Gastrin-mediated Signaling	5.4	0.15	-4.12
Xenobiotic Metabolism General Signaling Pathway	4.0	0.11	-4.12
G-Protein Coupled Receptor Signaling	9.5	0.09	-4.13
Pulmonary Healing Signaling Pathway	6.2	0.12	-4.20
HER-2 Signaling in Breast Cancer	5.3	0.11	-4.20
Response to elevated platelet cytosolic Ca2+	5.2	0.14	-4.24
DHCR24 Signaling Pathway	4.8	0.13	-4.24
mTOR Signaling	3.4	0.09	-4.24
CXCR4 Signaling	6.5	0.14	-4.36
Signaling by Rho Family GTPases	5.4	0.11	-4.38
Neuroinflammation Signaling Pathway	12.1	0.14	-4.42
Role of Macrophages, Fibroblasts and Endothelial Cells in Rheumatoid Arthritis	9.5	0.12	-4.43
TREM1 Signaling	11.5	0.27	-4.47
Tumor Microenvironment Pathway	4.4	0.11	-4.47
BBSome Signaling Pathway	3.7	0.08	-4.49
HIF1α Signaling	5.4	0.11	-4.49
Colorectal Cancer Metastasis Signaling	8.9	0.13	-4.54
Zn Homeostasis Signaling Pathway	2.6	0.06	-4.65
Pulmonary Fibrosis Idiopathic Signaling Pathway	7.3	0.11	-4.67
Irritable Bowel Syndrome Signaling Pathway	3.9	0.09	-4.71
Role of Tissue Factor in Cancer	12.2	0.17	-4.80
Pathogen Induced Cytokine Storm Signaling Pathway	9.9	0.12	-4.84
IL-8 Signaling	6.5	0.13	-4.90
Breast Cancer Regulation by Stathmin1	8.6	0.09	-5.00
Hepatic Fibrosis Signaling Pathway	9.2	0.11	-5.03
Serotonin Receptor Signaling	8.0	0.10	-5.11
Lung Ionic Balance Signaling Pathway	5.8	0.09	-5.13
Cardiac Hypertrophy Signaling (Enhanced)	6.3	0.09	-5.19
RHO GTPase cycle	4.5	0.08	-5.43
CREB Signaling in Neurons	7.1	0.09	-5.81
S100 Family Signaling Pathway	8.7	0.09	-6.02
FAK Signaling	4.5	0.07	-6.43
Molecular Mechanisms of Cancer	12.8	0.10	-7.18
Phagosome Formation	20.0	0.12	-7.54
Neutrophil degranulation	21.0	0.15	-7.71

Day 7: ΔclpB vs. PBS

Ingenuity Canonical Pathways	-log(p-value)	Ratio	z-score
Cell Cycle Checkpoints	34.7	0.25	7.70
Mitotic Metaphase and Anaphase	30.0	0.25	6.57
Synthesis of DNA	23.9	0.31	6.16
Mitotic Prometaphase	18.6	0.21	5.56
Regulation of mitotic cell cycle	15.9	0.30	5.10
DNA Replication Pre-Initiation	13.0	0.24	5.00
Oxidative Phosphorylation	11.7	0.22	4.90
Respiratory electron transport	11.0	0.24	4.58
Mitotic G2-G2/M phases	10.6	0.16	4.49
RHO GTPases Activate Formins	18.6	0.25	4.23
Class I MHC mediated antigen processing and presentation	5.0	0.09	4.00
Neddylation	2.6	0.08	3.90
Activation of the pre-replicative complex	12.2	0.43	3.87
Cell Cycle Control of Chromosomal Replication	9.7	0.28	3.87
HDR through Homologous Recombination (HRR) or Single Strand Annealing (SSA)	4.0	0.12	3.74
C-type lectin receptors (CLRs)	2.9	0.10	3.74
S Phase	10.7	0.22	3.71
Mitochondrial RNA degradation	12.0	0.52	3.61
Cellular response to hypoxia	5.3	0.17	3.61
KEAP1-NFE2L2 pathway	2.3	0.09	3.61
Protein Ubiquitination Pathway	4.2	0.09	3.55
TCR signaling	4.6	0.13	3.50
Interleukin-1 family signaling	4.5	0.12	3.50
tRNA processing in the mitochondrion	7.6	0.29	3.46
Regulation of Apoptosis	6.3	0.23	3.46
Somitogenesis	6.1	0.22	3.46
Metabolism of polyamines	5.8	0.20	3.46
NIK-->noncanonical NF-kB signaling	5.7	0.20	3.46
Hedgehog ligand biogenesis	5.3	0.19	3.46
TNFR2 non-canonical NF-kB pathway	5.1	0.18	3.46
Hedgehog 'on' state	2.1	0.08	3.46
Neutrophil Extracellular Trap Signaling Pathway	3.0	0.07	3.40
rRNA processing	7.9	0.34	3.32
Nucleosome assembly	5.8	0.22	3.32
Regulation of RUNX2 expression and activity	6.4	0.19	3.21
Degradation of beta-catenin by the destruction complex	5.1	0.15	3.21
Mitochondrial protein degradation	4.7	0.14	3.21
Centrosomal KIAA0586 Signaling Pathway	3.3	0.11	3.21
Complex I biogenesis	3.6	0.15	3.16
Hedgehog 'off' state	5.2	0.14	3.05
Regulation of mRNA stability by proteins that bind AU-rich elements	4.5	0.15	3.05
ABC-family proteins mediated transport	3.9	0.13	3.05
Kinesins	9.1	0.26	3.00
Signaling by ROBO receptors	4.6	0.10	3.00
Cytoprotection by HMOX1	3.5	0.16	3.00
Intra-Golgi and retrograde Golgi-to-ER traffic	3.5	0.09	2.98
PTEN Regulation	4.8	0.12	2.83

SUMOylation of DNA replication proteins	3.5	0.17	2.83
Complex IV assembly	3.4	0.17	2.83
Ribosomal Quality Control Signaling Pathway	2.2	0.07	2.83
Telomere Maintenance	3.3	0.12	2.71
Transcriptional regulation by RUNX1	5.9	0.13	2.68
Signaling by NOTCH4	3.0	0.10	2.67
Kinetochore Metaphase Signaling Pathway	23.5	0.34	2.60
Nuclear Cytoskeleton Signaling Pathway	6.5	0.12	2.60
Deubiquitination	3.3	0.08	2.56
MHC class II antigen presentation	6.4	0.15	2.52
TCF dependent signaling in response to WNT	3.7	0.10	2.52
Microautophagy Signaling Pathway	3.4	0.10	2.50
Erythrocytes take up oxygen and release carbon dioxide	7.7	0.86	2.45
IL-12 Signaling and Production in Macrophages	4.7	0.10	2.45
Interconversion of nucleotide di- and triphosphates	3.2	0.21	2.45
DNA Damage Bypass	2.0	0.13	2.45
Cohesin Chromatin Regulation Pathway	5.5	0.10	2.40
NER (Nucleotide Excision Repair, Enhanced Pathway)	3.8	0.13	2.33
TP53 Regulates Metabolic Genes	4.1	0.14	2.31
Regulation of TP53 Activity through Phosphorylation	3.7	0.13	2.31
Mismatch Repair in Eukaryotes	3.2	0.26	2.24
Natural Killer Cell Signaling	2.3	0.08	2.14
Resolution of Abasic Sites (AP sites)	4.1	0.21	2.12
FAT10 Signaling Pathway	7.8	0.25	2.00
Cachexia Signaling Pathway	7.1	0.10	2.00
Pathogenesis of Multiple Sclerosis	5.1	0.56	2.00
Mismatch Repair	2.6	0.25	2.00
Antigen Presentation Pathway	2.3	0.14	2.00
Formation of ATP by chemiosmotic coupling	2.2	0.20	2.00
Processing of Capped Intron-Containing Pre-mRNA	2.5	0.07	1.96
Cyclins and Cell Cycle Regulation	4.0	0.14	1.90
ISGylation Signaling Pathway	2.1	0.09	1.90
Erythrocytes take up carbon dioxide and release oxygen	7.5	0.64	1.89
Interferon Signaling	3.4	0.19	1.89
Hematoma Resolution Signaling Pathway	7.8	0.12	1.83
PTEN Signaling	2.7	0.09	1.73
Interferon alpha/beta signaling	2.6	0.12	1.67
Estrogen-mediated S-phase Entry	3.5	0.23	1.63
Pathogen Induced Cytokine Storm Signaling Pathway	6.2	0.09	1.62
TP53 Regulates Transcription of Cell Cycle Genes	5.8	0.22	1.51
Th1 Pathway	4.7	0.13	1.51
Interferon gamma signaling	2.9	0.11	1.51
Colorectal Cancer Metastasis Signaling	4.4	0.09	-1.61
CDK5 Signaling	2.2	0.09	-1.63
FAT10 Cancer Signaling Pathway	4.7	0.20	-1.67
GADD45 Signaling	4.1	0.17	-1.67
Synaptogenesis Signaling Pathway	2.0	0.07	-1.70
Cell Cycle: G2/M DNA Damage Checkpoint Regulation	4.7	0.20	-1.89
IGF-1 Signaling	2.2	0.10	-1.89

White Adipose Tissue Browning Pathway	2.6	0.09	-1.94
HEY1 Signaling Pathway	2.1	0.08	-1.94
Estrogen Receptor Signaling	4.0	0.08	-2.00
Sphingosine and Sphingosine-1-phosphate Metabolism	3.7	0.44	-2.00
FOXO-mediated transcription of cell cycle genes	2.5	0.24	-2.00
Breast Cancer Regulation by Stathmin1	5.5	0.08	-2.03
Cell junction organization	3.2	0.12	-2.11
Response to elevated platelet cytosolic Ca2+	5.0	0.13	-2.18
Role of Tissue Factor in Cancer	3.0	0.09	-2.18
RAF/MAP kinase cascade	8.2	0.12	-2.24
Sertoli Cell-Germ Cell Junction Signaling Pathway (Enhanced)	3.2	0.08	-2.24
Parkinson's Signaling Pathway	4.5	0.09	-2.27
Cardiac Hypertrophy Signaling (Enhanced)	3.1	0.07	-2.27
G-Protein Coupled Receptor Signaling	2.5	0.06	-2.27
Extra-nuclear estrogen signaling	2.7	0.12	-2.33
Senescence Pathway	3.3	0.08	-2.36
Sirtuin Signaling Pathway	4.2	0.09	-2.40
HER-2 Signaling in Breast Cancer	5.2	0.11	-2.45
Elastic fibre formation	2.2	0.14	-2.45
Phagosome Formation	2.3	0.06	-2.54
Sperm Motility	2.4	0.07	-2.65
ERK5 Signaling	2.2	0.11	-2.65
Serotonin Receptor Signaling	2.1	0.06	-2.65
Gap Junction Signaling	2.0	0.07	-2.68
Myelination Signaling Pathway	2.4	0.07	-2.71
Hepatic Fibrosis Signaling Pathway	2.9	0.07	-2.75
STAT3 Pathway	3.6	0.11	-2.83
CREB Signaling in Neurons	2.4	0.06	-3.02
RAR Activation	3.2	0.07	-3.05
Signaling by TGF-beta Receptor Complex	4.5	0.19	-3.16
Transcriptional Regulatory Network in Embryonic Stem Cells	2.4	0.09	-3.21
IL-8 Signaling	2.2	0.08	-3.21
Pulmonary Fibrosis Idiopathic Signaling Pathway	3.9	0.08	-3.53
Granzyme A Signaling	6.9	0.20	-3.87
Mitochondrial Dysfunction	10.0	0.12	-3.90
Molecular Mechanisms of Cancer	7.3	0.08	-3.91

Day 10: LVS vs. PBS

Ingenuity Canonical Pathways	-log(p-value)	Ratio	z-score
Mitochondrial RNA degradation	5.9	0.24	2.45
rRNA processing	5.3	0.19	2.45
tRNA processing in the mitochondrion	4.6	0.15	2.45
Oxidative Phosphorylation	2.3	0.06	2.45
Respiratory electron transport	2.1	0.06	2.24
Natural Killer Cell Signaling	3.1	0.05	2.12
IL-12 Signaling and Production in Macrophages	4.3	0.05	1.73
Neuroinflammation Signaling Pathway	5.2	0.05	-1.50
Translocation of SLC2A4 (GLUT4) to the plasma membrane	4.1	0.10	-1.63
L1CAM interactions	2.7	0.06	-1.63
Post-translational protein phosphorylation	2.4	0.06	-1.63
Cardiac Hypertrophy Signaling (Enhanced)	4.4	0.04	-1.70
S100 Family Signaling Pathway	2.7	0.03	-1.79
Parkinson's Signaling Pathway	6.7	0.06	-1.89
Acute Phase Response Signaling	2.7	0.05	-1.89
Regulation of Insulin-like Growth Factor (IGF) transport and uptake by IGFBPs	2.7	0.06	-1.89
p75 NTR receptor-mediated signalling	2.1	0.04	-1.89
Epithelial Adherens Junction Signaling	2.1	0.04	-1.89
Toll-like Receptor Cascades	3.0	0.13	-2.00
Unfolded protein response	2.7	0.07	-2.00
Role of Chondrocytes in Rheumatoid Arthritis Signaling Pathway	5.1	0.08	-2.11
Sertoli Cell-Sertoli Cell Junction Signaling	4.2	0.05	-2.11
Molecular Mechanisms of Cancer	4.7	0.03	-2.12
NOD1/2 Signaling Pathway	6.9	0.08	-2.14
CREB Signaling in Neurons	2.1	0.03	-2.14
Activin Inhibin Signaling Pathway	7.9	0.08	-2.18
NAP1L1 Transcription Regulation Signaling Pathway	2.8	0.07	-2.24
P2Y Purinergic Receptor Signaling Pathway	2.5	0.05	-2.24
ESR-mediated signaling	2.2	0.05	-2.24
Fcgamma receptor (FCGR) dependent phagocytosis	2.0	0.04	-2.24
Role of Tissue Factor in Cancer	4.3	0.06	-2.31
BBSome Signaling Pathway	2.3	0.03	-2.31
Breast Cancer Regulation by Stathmin1	3.3	0.03	-2.32
Signaling by VEGF	4.6	0.08	-2.33
NGF-stimulated transcription	4.7	0.15	-2.45
HMGB1 Signaling	2.7	0.05	-2.45
Extracellular matrix organization	2.4	0.06	-2.45
ISGylation Signaling Pathway	2.3	0.06	-2.45
Actin Cytoskeleton Signaling	2.1	0.04	-2.45
Tumor Microenvironment Pathway	5.7	0.07	-2.50
Response to elevated platelet cytosolic Ca2+	4.7	0.08	-2.53
Th2 Pathway	4.4	0.07	-2.53
HIF1α Signaling	3.6	0.05	-2.53
RAF/MAP kinase cascade	2.3	0.04	-2.53
Pulmonary Fibrosis Idiopathic Signaling Pathway	2.1	0.03	-2.53
TREM1 Signaling	3.9	0.09	-2.65
Pyroptosis Signaling Pathway	3.2	0.07	-2.65

CXCR4 Signaling	3.1	0.05	-2.65
Cargo recognition for clathrin-mediated endocytosis	3.1	0.07	-2.65
Clathrin-mediated endocytosis	2.6	0.05	-2.65
ABRA Signaling Pathway	4.4	0.09	-2.83
Cell junction organization	4.3	0.09	-2.83
Nuclear Cytoskeleton Signaling Pathway	2.3	0.04	-2.83
Colorectal Cancer Metastasis Signaling	4.4	0.05	-2.89
NAFLD Signaling Pathway	6.8	0.07	-3.00
ILK Signaling	3.2	0.05	-3.00
IL-8 Signaling	3.1	0.05	-3.00
Signaling by Rho Family GTPases	2.8	0.04	-3.00
Interleukin-4 and Interleukin-13 signaling	6.2	0.10	-3.32
Phagosome Formation	6.3	0.04	-3.66
RHO GTPase cycle	5.6	0.05	-3.71
Neutrophil degranulation	2.8	0.03	-4.00

Day 10: ΔclpB vs. PBS

Ingenuity Canonical Pathways	-log(p-value)	Ratio	z-score
Cellular Effects of Sildenafil (Viagra)	7.7	0.09	3.65
PTEN Signaling	4.8	0.13	3.36
RHOGDI Signaling	2.6	0.09	3.32
PPAR Signaling	5.8	0.17	2.83
Antioxidant Action of Vitamin C	2.6	0.11	2.53
PPARα/RXRα Activation	4.0	0.11	2.00
IL-12 Signaling and Production in Macrophages	10.0	0.15	1.57
JAK/STAT Signaling	2.9	0.13	-1.51
Interferon alpha/beta signaling	5.1	0.18	-1.60
Role of PKR in Interferon Induction and Antiviral Response	4.8	0.14	-1.60
Th2 Pathway	4.7	0.14	-1.61
PI3K/AKT Signaling	4.4	0.12	-1.63
Sphingolipid metabolism	3.3	0.13	-1.63
Inhibition of Angiogenesis by TSP1	3.2	0.21	-1.63
Thrombin signalling through proteinase activated receptors (PARs)	2.5	0.19	-1.63
Signal amplification	2.5	0.18	-1.63
GADD45 Signaling	3.5	0.17	-1.67
Transcriptional regulation of granulopoiesis	3.4	0.18	-1.67
IL-7 Signaling Pathway	2.5	0.13	-1.67
CDK5 Signaling	2.5	0.11	-1.67
Macrophage Classical Activation Signaling Pathway	8.7	0.16	-1.67
Ferroptosis Signaling Pathway	2.0	0.09	-1.73
Role of Osteoblasts in Rheumatoid Arthritis Signaling Pathway	7.8	0.14	-1.83
ERK/MAPK Signaling	4.6	0.11	-1.89
Protein Kinase A Signaling	4.5	0.09	-1.89
MyD88:MAL(TIRAP) cascade initiated on plasma membrane	2.6	0.17	-1.89
Neuregulin Signaling	2.6	0.11	-1.89
Oncostatin M Signaling	2.5	0.16	-1.89
Regulation of Actin-based Motility by Rho	2.3	0.11	-1.89
Role of Hypercytokinemia/hyperchemokinemia in the Pathogenesis of Influenza	5.4	0.17	-1.94
Ephrin Receptor Signaling	5.2	0.12	-1.94
Proteasomal PSMD10 Signaling Pathway	2.4	0.11	-1.94
Ephrin A Signaling	5.8	0.15	-1.96
Immunoregulatory interactions between a Lymphoid and a non-Lymphoid cell	3.1	0.10	-1.96
Immunogenic Cell Death Signaling Pathway	6.6	0.19	-2.00
Ceramide Degradation	3.9	0.57	-2.00
Sphingosine and Sphingosine-1-phosphate Metabolism	3.4	0.44	-2.00
Prostanoid Biosynthesis	3.0	0.36	-2.00
GP1b-IX-V activation signalling	2.8	0.33	-2.00
Necroptosis Signaling Pathway	2.5	0.10	-2.00
Airway Inflammation in Asthma	2.5	0.18	-2.00
Synthesis of Prostaglandins (PG) and Thromboxanes (TX)	2.4	0.27	-2.00
Response of EIF2AK1 (HRI) to heme deficiency	2.4	0.27	-2.00
Putrescine Degradation III	2.4	0.21	-2.00
Signal regulatory protein family interactions	2.3	0.25	-2.00
Metabolism of Angiotensinogen to Angiotensins	2.2	0.24	-2.00
FOXO-mediated transcription of cell cycle genes	2.2	0.24	-2.00

RHO GTPases Activate ROCKs	2.0	0.21	-2.00
Autism Signaling Pathway	3.0	0.09	-2.04
p38 MAPK Signaling	7.4	0.18	-2.07
Leukocyte Extravasation Signaling	3.7	0.11	-2.07
Macropinocytosis Signaling	5.1	0.18	-2.12
Toll-like Receptor Cascades	4.2	0.25	-2.12
GM-CSF Signaling	2.9	0.14	-2.12
Thrombopoietin Signaling	2.1	0.13	-2.12
Osteoarthritis Pathway	8.5	0.14	-2.13
Hepatitis B Chronic Liver Pathogenesis Signaling Pathway	4.3	0.12	-2.13
Elastic fibre formation	8.3	0.32	-2.14
D-myo-inositol (1,4,5,6)-Tetrakisphosphate Biosynthesis	2.6	0.10	-2.14
D-myo-inositol (3,4,5,6)-tetrakisphosphate Biosynthesis	2.6	0.10	-2.14
3-phosphoinositide Degradation	2.3	0.09	-2.14
Cerebral Malformation Signaling Pathway	5.4	0.15	-2.24
Trafficking and processing of endosomal TLR	3.7	0.39	-2.24
Platelet Aggregation (Plug Formation)	3.7	0.39	-2.24
cAMP-mediated signaling	2.8	0.09	-2.24
RHO GTPases Activate NADPH Oxidases	2.4	0.21	-2.24
Inflammasome pathway	2.4	0.21	-2.24
IL-1 Signaling	2.3	0.11	-2.24
Integrin signaling	2.2	0.19	-2.24
Complement System	2.1	0.15	-2.24
CDP-diacylglycerol Biosynthesis I	2.1	0.18	-2.24
FAT10 Cancer Signaling Pathway	5.6	0.24	-2.31
Platelet homeostasis	3.3	0.14	-2.31
Role of JAK family kinases in IL-6-type Cytokine Signaling	6.2	0.20	-2.32
3-phosphoinositide Biosynthesis	2.3	0.09	-2.32
HEY1 Signaling Pathway	2.2	0.09	-2.32
G-protein beta:gamma signalling	5.1	0.28	-2.33
Signaling by PTK6	3.1	0.16	-2.33
PAK Signaling	3.0	0.12	-2.33
NAP1L1 Transcription Regulation Signaling Pathway	2.1	0.11	-2.33
IGF-1 Signaling	2.1	0.11	-2.33
Ceramide Signaling	2.1	0.11	-2.33
Thyroid Cancer Signaling	2.1	0.12	-2.33
Macrophage Alternative Activation Signaling Pathway	11.0	0.18	-2.34
Docosahexaenoic Acid (DHA) Signaling	6.3	0.12	-2.34
Platelet Adhesion to exposed collagen	4.3	0.38	-2.45
IL-17A Signaling in Gastric Cells	3.9	0.27	-2.45
Syndecan interactions	2.9	0.22	-2.45
Glutathione Redox Reactions I	2.7	0.20	-2.45
Renal Cell Carcinoma Signaling	2.6	0.13	-2.45
Actin Nucleation by ARP-WASP Complex	2.6	0.12	-2.45
Surfactant metabolism	2.5	0.19	-2.45
GPVI-mediated activation cascade	2.3	0.17	-2.45
Coagulation System	2.3	0.17	-2.45
Transcriptional regulation by the AP-2 (TFAP2) family of transcription factors	2.3	0.17	-2.45
Adipogenesis pathway	3.3	0.12	-2.50

Multiple Sclerosis Signaling Pathway	13.3	0.18	-2.53
UVA-Induced MAPK Signaling	3.9	0.14	-2.53
Phospholipases	3.6	0.16	-2.53
TGF-β Signaling	3.4	0.13	-2.53
Semaphorin interactions	3.2	0.16	-2.53
Glioma Signaling	2.7	0.11	-2.53
Signaling by MET	2.5	0.13	-2.53
Transcriptional regulation of white adipocyte differentiation	2.3	0.12	-2.53
Sheddase Signaling Pathway	10.9	0.17	-2.54
Parkinson's Signaling Pathway	7.8	0.12	-2.54
Nicotinate metabolism	3.4	0.23	-2.65
Detoxification of Reactive Oxygen Species	2.9	0.19	-2.65
Ephrin B Signaling	2.8	0.14	-2.65
Retinoid metabolism and transport	2.5	0.16	-2.65
Antimicrobial peptides	2.2	0.14	-2.65
Glycation Signaling Pathway	10.9	0.16	-2.67
Glycerophospholipid biosynthesis	4.1	0.13	-2.67
Signaling by TGF-beta Receptor Complex	7.1	0.26	-2.67
Glioma Invasiveness Signaling	5.5	0.20	-2.67
Chemokine Signaling	4.8	0.17	-2.67
Gαq Signaling	2.7	0.10	-2.67
Degradation of the extracellular matrix	3.0	0.14	-2.71
Renin-Angiotensin Signaling	2.9	0.12	-2.71
Transport of bile salts and organic acids, metal ions and amine compounds	2.8	0.13	-2.71
ERBB Signaling	2.5	0.12	-2.71
Pancreatic Adenocarcinoma Signaling	2.3	0.10	-2.71
Cargo recognition for clathrin-mediated endocytosis	2.1	0.11	-2.71
Cachexia Signaling Pathway	7.8	0.12	-2.78
Regulation of TLR by endogenous ligand	5.5	0.36	-2.83
NGF-stimulated transcription	3.5	0.21	-2.83
D-myo-inositol-5-phosphate Metabolism	3.4	0.10	-2.83
UVC-Induced MAPK Signaling	2.7	0.16	-2.83
Triacylglycerol Biosynthesis	2.3	0.14	-2.83
Iron uptake and transport	2.3	0.13	-2.83
Orexin Signaling Pathway	2.4	0.09	-2.84
Interleukin-10 signaling	9.2	0.33	-2.84
Post-translational protein phosphorylation	4.0	0.14	-2.84
Integration of energy metabolism	4.0	0.14	-2.84
Sphingosine-1-phosphate Signaling	4.6	0.14	-2.89
Gαi Signaling	4.4	0.13	-2.89
Extra-nuclear estrogen signaling	3.9	0.16	-2.89
CCR3 Signaling in Eosinophils	3.8	0.13	-2.89
Apelin Endothelial Signaling Pathway	3.5	0.12	-2.89
Superpathway of Inositol Phosphate Compounds	2.8	0.09	-2.98
Sperm Motility	11.0	0.16	-3.00
Neuroinflammation Signaling Pathway	6.8	0.12	-3.00
Smooth Muscle Contraction	4.0	0.21	-3.00
Arachidonic acid metabolism	3.8	0.20	-3.00
MSP-RON Signaling in Cancer Cells Pathway	3.6	0.12	-3.00

Paxillin Signaling	3.5	0.13	-3.00
Caveolar-mediated Endocytosis Signaling	2.5	0.13	-3.00
LPS-stimulated MAPK Signaling	2.3	0.12	-3.00
Hepatic Cholestasis	5.8	0.12	-3.02
Sertoli Cell-Germ Cell Junction Signaling Pathway (Enhanced)	5.4	0.12	-3.02
HGF Signaling	5.7	0.15	-3.05
G alpha (12/13) signalling events	4.2	0.16	-3.05
IL-17A Signaling in Fibroblasts	4.2	0.16	-3.05
Clathrin-mediated endocytosis	2.2	0.10	-3.05
Regulation of Insulin-like Growth Factor (IGF) transport and uptake by IGFBPs	4.3	0.14	-3.15
Toll-like Receptor Signaling	5.8	0.20	-3.16
Formation of Fibrin Clot (Clotting Cascade)	5.2	0.26	-3.16
Relaxin Signaling	3.1	0.11	-3.16
Actin Cytoskeleton Signaling	2.8	0.09	-3.16
fMLP Signaling in Neutrophils	2.5	0.11	-3.16
EPH-Ephrin signaling	2.0	0.11	-3.16
Role of Pattern Recognition Receptors in Recognition of Bacteria and Viruses	10.2	0.19	-3.21
Integrin cell surface interactions	4.6	0.17	-3.21
Factors Promoting Cardiogenesis in Vertebrates	2.4	0.10	-3.21
Chronic Myeloid Leukemia Signaling	2.6	0.09	-3.27
Role of NFAT in Cardiac Hypertrophy	3.1	0.10	-3.30
ABRA Signaling Pathway	2.7	0.13	-3.32
Cell junction organization	2.5	0.12	-3.32
Activin Inhibin Signaling Pathway	11.1	0.17	-3.33
TREM1 Signaling	7.1	0.22	-3.36
P2Y Purinergic Receptor Signaling Pathway	5.5	0.15	-3.36
L1CAM interactions	3.8	0.13	-3.36
White Adipose Tissue Browning Pathway	2.8	0.11	-3.36
IL-15 Production	10.4	0.21	-3.40
NOD1/2 Signaling Pathway	6.1	0.14	-3.40
Endothelin-1 Signaling	5.1	0.12	-3.41
Signaling by VEGF	6.5	0.18	-3.44
ILK Signaling	4.5	0.12	-3.44
RAC Signaling	3.8	0.12	-3.46
Reelin Signaling in Neurons	2.4	0.10	-3.46
TR/RXR Activation	4.6	0.14	-3.50
Glioblastoma Multiforme Signaling	2.7	0.10	-3.50
Transcriptional Regulatory Network in Embryonic Stem Cells	2.5	0.10	-3.50
Ion channel transport	2.0	0.09	-3.50
Epithelial Membrane Protein Signaling Pathway	9.0	0.17	-3.53
Production of Nitric Oxide and Reactive Oxygen Species in Macrophages	8.5	0.16	-3.53
Oxytocin Signaling Pathway	3.2	0.09	-3.53
RAR Activation	7.4	0.11	-3.54
IL-17 Signaling	5.3	0.13	-3.55
IL-13 Signaling Pathway	6.1	0.16	-3.58
STAT3 Pathway	12.1	0.22	-3.64
Atherosclerosis Signaling	7.3	0.17	-3.64
Nuclear Cytoskeleton Signaling Pathway	2.2	0.09	-3.64
ID1 Signaling Pathway	10.4	0.17	-3.66

Interleukin-4 and Interleukin-13 signaling	12.3	0.24	-3.66
Sertoli Cell-Sertoli Cell Junction Signaling	5.5	0.12	-3.66
Neutrophil Extracellular Trap Signaling Pathway	6.1	0.10	-3.70
IL-6 Signaling	7.7	0.18	-3.71
HMGB1 Signaling	7.2	0.16	-3.71
Thrombin Signaling	7.0	0.14	-3.71
Regulation of the Epithelial Mesenchymal Transition by Growth Factors Pathway	4.6	0.12	-3.71
Plasma lipoprotein assembly, remodeling, and clearance	5.1	0.18	-3.74
14-3-3-mediated Signaling	4.5	0.14	-3.74
Warburg Effect Signaling Pathway	3.1	0.12	-3.74
NAFLD Signaling Pathway	7.4	0.14	-3.77
RAF/MAP kinase cascade	4.2	0.10	-3.80
Synaptogenesis Signaling Pathway	3.2	0.09	-3.80
DHCR24 Signaling Pathway	2.7	0.11	-3.87
Myelination Signaling Pathway	4.3	0.10	-3.89
Colorectal Cancer Metastasis Signaling	7.0	0.13	-3.92
Acute Phase Response Signaling	5.7	0.13	-4.03
Pancreatic Secretion Signaling Pathway	2.4	0.09	-4.03
Pulmonary Healing Signaling Pathway	6.9	0.14	-4.04
HIF1α Signaling	4.9	0.12	-4.08
Irritable Bowel Syndrome Signaling Pathway	7.8	0.13	-4.11
Wound Healing Signaling Pathway	13.4	0.17	-4.12
Pyroptosis Signaling Pathway	6.8	0.19	-4.12
GNRH Signaling	3.7	0.11	-4.12
Cardiac Hypertrophy Signaling	3.4	0.10	-4.15
HER-2 Signaling in Breast Cancer	2.8	0.09	-4.15
G alpha (i) signalling events	4.7	0.11	-4.16
Pathogen Induced Cytokine Storm Signaling Pathway	22.2	0.18	-4.23
Extracellular matrix organization	5.8	0.17	-4.24
Adrenomedullin signaling pathway	4.3	0.11	-4.26
Integrin Signaling	4.0	0.11	-4.26
Estrogen Receptor Signaling	2.4	0.08	-4.32
Role of Osteoclasts in Rheumatoid Arthritis Signaling Pathway	7.7	0.12	-4.35
Cholecystokinin/Gastrin-mediated Signaling	5.9	0.16	-4.36
CXCR4 Signaling	8.6	0.17	-4.38
Signaling by Rho Family GTPases	6.2	0.12	-4.38
G alpha (q) signalling events	5.9	0.14	-4.49
Tumor Microenvironment Pathway	8.5	0.16	-4.54
Eicosanoid Signaling	8.1	0.13	-4.56
Role of Chondrocytes in Rheumatoid Arthritis Signaling Pathway	14.0	0.23	-4.70
Cell surface interactions at the vascular wall	5.3	0.12	-4.71
Role of Macrophages, Fibroblasts and Endothelial Cells in Rheumatoid Arthritis	11.6	0.14	-4.81
Zn Homeostasis Signaling Pathway	2.7	0.06	-5.01
G-Protein Coupled Receptor Signaling	10.0	0.10	-5.02
Response to elevated platelet cytosolic Ca2+	13.5	0.24	-5.21
IL-8 Signaling	8.2	0.15	-5.29
Hepatic Fibrosis Signaling Pathway	11.9	0.13	-5.37
BBSome Signaling Pathway	4.4	0.09	-5.38
Class A/1 (Rhodopsin-like receptors)	6.3	0.11	-5.43

Pulmonary Fibrosis Idiopathic Signaling Pathway	7.4	0.12	-5.52
Cardiac Hypertrophy Signaling (Enhanced)	12.6	0.12	-5.55
Role of Tissue Factor in Cancer	10.7	0.17	-5.58
Lung Ionic Balance Signaling Pathway	7.7	0.10	-5.72
RHO GTPase cycle	7.3	0.11	-5.77
Breast Cancer Regulation by Stathmin1	10.7	0.11	-6.20
Serotonin Receptor Signaling	8.0	0.11	-6.22
CREB Signaling in Neurons	8.7	0.10	-6.61
S100 Family Signaling Pathway	9.4	0.10	-6.93
FAK Signaling	6.7	0.08	-6.93
Phagosome Formation	15.1	0.12	-7.99
Molecular Mechanisms of Cancer	18.1	0.12	-8.08
Neutrophil degranulation	25.5	0.17	-8.89

Day 24: LVS vs. PBS

Ingenuity Canonical Pathways	-log(p-value)	Ratio	z-score
Cellular Effects of Sildenafil (Viagra)	3.3	0.03	2.52
PTEN Signaling	2.5	0.05	1.89
Th1 Pathway	3.0	0.06	1.63
HMGB1 Signaling	3.0	0.05	-1.63
L1CAM interactions	2.3	0.05	-1.63
Post-translational protein phosphorylation	5.0	0.08	-1.67
Regulation of Insulin-like Growth Factor (IGF) transport and uptake by IGFBPs	4.5	0.07	-1.67
Role of Chondrocytes in Rheumatoid Arthritis Signaling Pathway	4.0	0.06	-1.67
NOD1/2 Signaling Pathway	3.1	0.05	-1.67
HEY1 Signaling Pathway	2.3	0.04	-1.89
Sertoli Cell-Sertoli Cell Junction Signaling	2.9	0.04	-1.90
G-Protein Coupled Receptor Signaling	4.3	0.03	-1.96
Caveolar-mediated Endocytosis Signaling	3.3	0.08	-2.00
TREM1 Signaling	3.3	0.08	-2.00
Signaling by TGFBR3	2.4	0.08	-2.00
Gap junction trafficking and regulation	2.4	0.08	-2.00
Signaling by PTK6	2.3	0.07	-2.00
Interleukin-4 and Interleukin-13 signaling	4.1	0.07	-2.12
Sheddase Signaling Pathway	2.3	0.04	-2.12
Role of Tissue Factor in Cancer	2.3	0.04	-2.12
Colorectal Cancer Metastasis Signaling	2.1	0.03	-2.12
Cardiac Hypertrophy Signaling (Enhanced)	4.7	0.04	-2.18
STAT3 Pathway	6.6	0.09	-2.24
Extra-nuclear estrogen signaling	2.6	0.07	-2.24
Toll-like Receptor Signaling	2.5	0.06	-2.24
G alpha (12/13) signalling events	2.5	0.06	-2.24
Degradation of the extracellular matrix	2.4	0.06	-2.24
IL-15 Production	2.3	0.05	-2.24
ID1 Signaling Pathway	4.9	0.06	-2.31
Elastic fibre formation	4.7	0.14	-2.45
Zn Homeostasis Signaling Pathway	2.5	0.02	-2.45
Cholecystokinin/Gastrin-mediated Signaling	2.4	0.05	-2.45
Response to elevated platelet cytosolic Ca2+	5.1	0.08	-2.53
Cell surface interactions at the vascular wall	3.4	0.05	-2.53
Breast Cancer Regulation by Stathmin1	3.1	0.03	-2.67
Hepatic Fibrosis Signaling Pathway	3.0	0.03	-2.67
Lung Ionic Balance Signaling Pathway	2.1	0.03	-2.67
IL-8 Signaling	2.3	0.04	-2.83
HIF1α Signaling	2.3	0.04	-2.83
Signaling by VEGF	5.0	0.08	-3.00
Eicosanoid Signaling	5.4	0.05	-3.05
Pulmonary Fibrosis Idiopathic Signaling Pathway	4.1	0.04	-3.05
FAK Signaling	2.8	0.02	-3.13
CREB Signaling in Neurons	3.9	0.03	-3.15
Serotonin Receptor Signaling	2.5	0.03	-3.21
Molecular Mechanisms of Cancer	6.6	0.04	-3.53
S100 Family Signaling Pathway	4.1	0.03	-3.84

Phagosome Formation	5.3	0.04	-4.38
Neutrophil degranulation	6.6	0.05	-4.69

Day 24: ΔclpB vs. PBS

Ingenuity Canonical Pathways	-log(p-value)	Ratio	z-score
Mitochondrial RNA degradation	2.1	0.20	2.24
PPAR Signaling	4.3	0.16	2.18
Cellular Effects of Sildenafil (Viagra)	8.9	0.11	2.02
PTEN Signaling	3.0	0.12	1.94
Antioxidant Action of Vitamin C	2.5	0.12	1.90
RHOGDI Signaling	3.6	0.11	1.81
CLEAR Signaling Pathway	4.0	0.11	1.62
Sphingosine-1-phosphate Signaling	6.0	0.18	-1.50
Adipogenesis pathway	2.6	0.12	-1.50
PAK Signaling	3.4	0.14	-1.51
Chronic Myeloid Leukemia Signaling	3.4	0.10	-1.51
Thrombin Signaling	5.7	0.14	-1.53
G alpha (12/13) signalling events	4.2	0.18	-1.60
Docosahexaenoic Acid (DHA) Signaling	5.1	0.12	-1.62
Sphingolipid metabolism	4.8	0.16	-1.63
Syndecan interactions	2.6	0.22	-1.63
RHO GTPases activate PKNs	2.5	0.21	-1.63
CDP-diacylglycerol Biosynthesis I	2.5	0.21	-1.63
Phosphatidylglycerol Biosynthesis II (Non-plastidic)	2.4	0.20	-1.63
Signal amplification	2.2	0.18	-1.63
Cardiac β-adrenergic Signaling	2.4	0.11	-1.67
IGF-1 Signaling	2.1	0.11	-1.67
Ferroptosis Signaling Pathway	4.5	0.15	-1.70
Osteoarthritis Pathway	7.5	0.15	-1.71
Leukocyte Extravasation Signaling	5.4	0.14	-1.71
GDNF Family Ligand-Receptor Interactions	3.3	0.16	-1.73
Apelin Endothelial Signaling Pathway	2.8	0.12	-1.73
Th2 Pathway	4.8	0.15	-1.79
Ephrin A Signaling	4.4	0.14	-1.79
PIP3 activates AKT signaling	4.1	0.14	-1.79
O-linked glycosylation	3.1	0.13	-1.81
Role of Osteoblasts in Rheumatoid Arthritis Signaling Pathway	5.9	0.13	-1.83
HGF Signaling	8.4	0.20	-1.89
HEY1 Signaling Pathway	2.7	0.11	-1.89
G-protein beta:gamma signalling	2.9	0.22	-1.89
Transcriptional regulation by RUNX2	2.3	0.16	-1.89
MIF Regulation of Innate Immunity	2.0	0.15	-1.89
Paxillin Signaling	3.4	0.14	-1.90
CCR3 Signaling in Eosinophils	2.7	0.12	-1.90
Sperm Motility	7.6	0.14	-1.94
Role of JAK family kinases in IL-6-type Cytokine Signaling	4.1	0.17	-1.94
Chemokine Signaling	3.5	0.16	-1.94
Orexin Signaling Pathway	3.4	0.11	-1.96
Glycerophospholipid biosynthesis	5.5	0.16	-1.96
Neutrophil Extracellular Trap Signaling Pathway	4.9	0.11	-1.98
MSP-RON Signaling in Cancer Cells Pathway	3.8	0.13	-2.00
Ceramide Degradation	3.6	0.57	-2.00

CGAS-STING Signaling Pathway	2.6	0.12	-2.00
GP1b-IX-V activation signalling	2.6	0.33	-2.00
Tryptophan Degradation X (Mammalian, via Tryptamine)	2.4	0.20	-2.00
Signal regulatory protein family interactions	2.1	0.25	-2.00
Immunoregulatory interactions between a Lymphoid and a non-Lymphoid cell	3.4	0.11	-2.04
Estrogen Receptor Signaling	4.9	0.11	-2.06
p38 MAPK Signaling	4.6	0.15	-2.07
Human Embryonic Stem Cell Pluripotency	2.0	0.10	-2.07
Extra-nuclear estrogen signaling	2.8	0.15	-2.11
fMLP Signaling in Neutrophils	2.0	0.11	-2.11
Signaling by PTK6	2.1	0.15	-2.12
Caveolar-mediated Endocytosis Signaling	2.1	0.13	-2.12
Nuclear Cytoskeleton Signaling Pathway	3.2	0.11	-2.13
Glioma Invasiveness Signaling	4.8	0.20	-2.14
Actin Cytoskeleton Signaling	5.4	0.13	-2.18
P2Y Purinergic Receptor Signaling Pathway	5.2	0.16	-2.18
Superpathway of Inositol Phosphate Compounds	2.1	0.09	-2.18
Glycation Signaling Pathway	8.1	0.16	-2.20
Macrophage Classical Activation Signaling Pathway	5.2	0.14	-2.20
Synthesis of Lipoxins (LX)	5.7	0.83	-2.24
Leukotriene Biosynthesis	5.1	0.44	-2.24
Sphingosine and Sphingosine-1-phosphate Metabolism	4.4	0.56	-2.24
Trafficking and processing of endosomal TLR	3.5	0.39	-2.24
Platelet Aggregation (Plug Formation)	3.5	0.39	-2.24
Response of EIF2AK1 (HRI) to heme deficiency	3.1	0.33	-2.24
Role of NFAT in Cardiac Hypertrophy	3.0	0.11	-2.24
Putrescine Degradation III	2.9	0.25	-2.24
Airway Inflammation in Asthma	2.9	0.21	-2.24
Macrophage Alternative Activation Signaling Pathway	7.6	0.16	-2.27
Hepatic Cholestasis	4.7	0.12	-2.27
RHO GTPase cycle	11.4	0.14	-2.29
STAT3 Pathway	6.0	0.17	-2.31
Interleukin-10 signaling	5.6	0.27	-2.31
Signaling by TGF-beta Receptor Complex	4.7	0.22	-2.31
Phospholipases	4.4	0.19	-2.31
Transport of bile salts and organic acids, metal ions and amine compounds	2.9	0.14	-2.31
Fcγ Receptor-mediated Phagocytosis in Macrophages and Monocytes	2.5	0.13	-2.31
Role of PKR in Interferon Induction and Antiviral Response	2.2	0.11	-2.31
RHOA Signaling	2.7	0.12	-2.32
Renal Cell Carcinoma Signaling	2.1	0.13	-2.33
Endothelin-1 Signaling	5.4	0.14	-2.35
Glioblastoma Multiforme Signaling	2.8	0.11	-2.36
Multiple Sclerosis Signaling Pathway	8.4	0.16	-2.40
RAR Activation	5.3	0.11	-2.41
Cerebral Malformation Signaling Pathway	6.7	0.18	-2.45
Platelet Adhesion to exposed collagen	4.0	0.38	-2.45
RHO GTPases Activate NADPH Oxidases	2.9	0.25	-2.45
Complement System	2.4	0.18	-2.45
Surfactant metabolism	2.2	0.19	-2.45

GPVI-mediated activation cascade	2.1	0.17	-2.45
Coagulation System	2.1	0.17	-2.45
Elastic fibre formation	6.6	0.30	-2.50
RAC Signaling	4.0	0.14	-2.50
14-3-3-mediated Signaling	3.7	0.14	-2.50
Binding and Uptake of Ligands by Scavenger Receptors	2.2	0.11	-2.50
TREM1 Signaling	7.0	0.23	-2.50
IL-6 Signaling	3.9	0.14	-2.50
Integration of energy metabolism	3.8	0.15	-2.50
ISGylation Signaling Pathway	3.8	0.15	-2.50
GNRH Signaling	3.3	0.11	-2.50
Proteasomal PSMD10 Signaling Pathway	3.2	0.13	-2.50
G alpha (i) signalling events	3.3	0.11	-2.50
Synaptogenesis Signaling Pathway	3.2	0.10	-2.50
Signaling by VEGF	5.6	0.18	-2.52
Parkinson's Signaling Pathway	8.5	0.14	-2.53
Neuroinflammation Signaling Pathway	8.3	0.14	-2.53
Production of Nitric Oxide and Reactive Oxygen Species in Macrophages	6.0	0.15	-2.60
Sheddase Signaling Pathway	8.1	0.16	-2.61
Myelination Signaling Pathway	3.6	0.10	-2.61
Regulation of TLR by endogenous ligand	4.0	0.32	-2.65
Glutathione Redox Reactions I	3.1	0.23	-2.65
Toll-like Receptor Cascades	2.9	0.22	-2.65
White Adipose Tissue Browning Pathway	3.0	0.12	-2.67
DHCR24 Signaling Pathway	2.9	0.12	-2.67
Transcriptional Regulatory Network in Embryonic Stem Cells	2.2	0.10	-2.67
Role of Pattern Recognition Receptors in Recognition of Bacteria and Viruses	8.8	0.19	-2.67
Role of Hypercytokinemia/hyperchemokinemia in the Pathogenesis of Influenza	5.3	0.19	-2.67
Integrin Signaling	5.3	0.13	-2.71
ILK Signaling	4.9	0.13	-2.71
HER-2 Signaling in Breast Cancer	2.7	0.10	-2.71
Epithelial Membrane Protein Signaling Pathway	6.0	0.15	-2.75
Activin Inhibin Signaling Pathway	7.2	0.15	-2.83
HMGB1 Signaling	5.0	0.14	-2.83
Oxytocin Signaling Pathway	4.4	0.11	-2.83
NGF-stimulated transcription	3.1	0.21	-2.83
Ephrin B Signaling	2.9	0.15	-2.83
Smooth Muscle Contraction	2.8	0.19	-2.83
Antimicrobial peptides	2.4	0.16	-2.83
Adrenomedullin signaling pathway	3.4	0.11	-2.84
Acute Phase Response Signaling	4.2	0.13	-2.84
Cell junction organization	4.1	0.16	-2.84
NAD Signaling Pathway	2.2	0.11	-2.84
Pulmonary Healing Signaling Pathway	3.8	0.12	-2.86
Interferon gamma signaling	2.3	0.12	-2.89
Role of Osteoclasts in Rheumatoid Arthritis Signaling Pathway	6.1	0.12	-2.96
Cholecystokinin/Gastrin-mediated Signaling	5.0	0.16	-2.98
CXCR4 Signaling	6.7	0.16	-2.99
Wound Healing Signaling Pathway	7.5	0.15	-3.00

L1CAM interactions	3.6	0.14	-3.00
Collagen degradation	2.2	0.14	-3.00
MHC class II antigen presentation	2.1	0.11	-3.05
Warburg Effect Signaling Pathway	2.1	0.11	-3.05
TR/RXR Activation	2.1	0.11	-3.05
Role of Macrophages, Fibroblasts and Endothelial Cells in Rheumatoid Arthritis	8.1	0.13	-3.13
RAF/MAP kinase cascade	2.9	0.10	-3.14
Interleukin-4 and Interleukin-13 signaling	12.4	0.26	-3.16
HIF1α Signaling	5.7	0.14	-3.16
Irritable Bowel Syndrome Signaling Pathway	7.7	0.14	-3.16
Detoxification of Reactive Oxygen Species	4.8	0.27	-3.16
Formation of Fibrin Clot (Clotting Cascade)	4.6	0.26	-3.16
Toll-like Receptor Signaling	3.2	0.16	-3.16
Iron uptake and transport	3.0	0.17	-3.16
NAP1L1 Transcription Regulation Signaling Pathway	2.2	0.12	-3.16
Integrin cell surface interactions	3.9	0.17	-3.21
IL-13 Signaling Pathway	2.7	0.12	-3.21
Hepatic Fibrosis Signaling Pathway	11.9	0.14	-3.22
IL-17 Signaling	4.7	0.13	-3.27
Cardiac Hypertrophy Signaling	3.9	0.11	-3.27
Cardiac Hypertrophy Signaling (Enhanced)	9.9	0.12	-3.29
Cell surface interactions at the vascular wall	6.0	0.14	-3.29
Relaxin Signaling	2.5	0.11	-3.32
EPH-Ephrin signaling	2.0	0.12	-3.32
NOD1/2 Signaling Pathway	4.6	0.13	-3.40
NAFLD Signaling Pathway	6.1	0.14	-3.41
Plasma lipoprotein assembly, remodeling, and clearance	3.3	0.16	-3.46
Post-translational protein phosphorylation	3.9	0.15	-3.50
Signaling by Rho Family GTPases	8.2	0.15	-3.53
Sertoli Cell-Sertoli Cell Junction Signaling	6.6	0.14	-3.54
Regulation of the Epithelial Mesenchymal Transition by Growth Factors Pathway	4.5	0.13	-3.55
Atherosclerosis Signaling	8.1	0.19	-3.58
Arachidonic acid metabolism	6.5	0.29	-3.61
Interferon alpha/beta signaling	3.8	0.17	-3.61
ABRA Signaling Pathway	3.2	0.15	-3.61
Extracellular matrix organization	4.4	0.16	-3.64
G alpha (q) signalling events	4.9	0.14	-3.67
IL-15 Production	6.5	0.18	-3.71
Colorectal Cancer Metastasis Signaling	7.0	0.14	-3.77
Tumor Microenvironment Pathway	6.6	0.16	-3.78
Eicosanoid Signaling	7.5	0.14	-3.78
Zn Homeostasis Signaling Pathway	2.9	0.07	-3.81
Pathogen Induced Cytokine Storm Signaling Pathway	18.9	0.18	-3.84
Class A/1 (Rhodopsin-like receptors)	5.3	0.11	-3.89
Regulation of Insulin-like Growth Factor (IGF) transport and uptake by IGFBPs	4.6	0.15	-3.90
Lung Ionic Balance Signaling Pathway	5.5	0.10	-3.98
ID1 Signaling Pathway	7.1	0.15	-4.02
Pancreatic Secretion Signaling Pathway	3.0	0.10	-4.08
G-Protein Coupled Receptor Signaling	8.8	0.11	-4.10

BBSome Signaling Pathway	3.9	0.09	-4.22
Breast Cancer Regulation by Stathmin1	11.8	0.13	-4.28
Role of Chondrocytes in Rheumatoid Arthritis Signaling Pathway	7.5	0.18	-4.32
IL-8 Signaling	7.9	0.16	-4.38
CREB Signaling in Neurons	9.4	0.12	-4.39
FAK Signaling	4.5	0.08	-4.44
Pulmonary Fibrosis Idiopathic Signaling Pathway	5.4	0.12	-4.67
S100 Family Signaling Pathway	9.8	0.11	-4.90
Role of Tissue Factor in Cancer	5.8	0.14	-5.01
Response to elevated platelet cytosolic Ca2+	11.2	0.23	-5.11
Serotonin Receptor Signaling	9.3	0.13	-5.60
Molecular Mechanisms of Cancer	15.7	0.12	-5.66
Phagosome Formation	16.6	0.14	-6.33
Neutrophil degranulation	24.0	0.18	-8.26
