# Supplementary material for: Transcriptional analyses of peripheral blood lymphocytes obtained shortly after primary immunization discriminate vaccines against Francisella tularensis
Source: Infect Immun. 2026 Aug 14;94(9):e00208-26. doi: 10.1128/iai.00208-26 (PMC13552110; doi:10.1128/iai.00208-26)
Supplement: Supplementary file 6 [file iai.00208-26-s0006.pdf]

Table S4. Differentially regulated genes overlapping across time points. Gene expression results were filtered as described in Fig. 4 and analyzed to identify 24 and 147 features that overlapped across time point withing each vaccine group, which are listed alphabetically. Six genes (in red) were differentially regulated in all time points after both LVS and *ΔclpB* vaccination.

LVS vs. PBS: Overlappinf genes across time points (24 features)

In red, genes overlapping with ΔclpB vs. PBS across time points

	Day 3		Day 7		Day 10		Day 24	
Name	Fold change	FDR p-value	Fold change	FDR p-value	Fold change	FDR p-value	Fold change	FDR p-value
AABR07006310.1	2.3	0.00004	-6.3	0.00060	-3.4	0.00018	-2.3	0.00275
Cd14	2.8	0.00000	-4.6	0.00000	-2.1	0.02174	-2.4	0.00006
Cd36	-3.2	0.00000	-4.6	0.00000	-2.2	0.00000	-1.8	0.00013
Colec12	1.7	0.00599	-4.5	0.00012	-2.1	0.01668	-1.9	0.00703
Cyfip1	1.7	0.00034	-3.6	0.00000	-1.9	0.00490	-1.8	0.00021
Dclk1	3.1	0.00122	3.1	0.01772	5.0	0.00000	2.4	0.02893
Fcnb	2.8	0.00000	-7.6	0.00000	-2.1	0.04894	-2.0	0.00017
Fpr2	-2.9	0.00019	-3.7	0.00027	-2.0	0.03413	-1.7	0.03358
Gas7	1.8	0.00138	-3.4	0.00011	-2.3	0.00114	-2.0	0.00199
Gyg1	2.8	0.00000	-2.2	0.00002	-1.8	0.00527	-1.8	0.00017
Gzmc	45.6	0.00753	175.4	0.00001	118.7	0.00028	292.7	0.00000
Gzmm	1.5	0.00150	2.1	0.00008	2.2	0.00000	1.7	0.00017
Hes1	-3.6	0.00000	-2.9	0.00078	-2.6	0.00036	-1.9	0.04245
Klra1	2.0	0.00824	2.6	0.00173	3.2	0.00000	3.3	0.00000
Klrg1	1.8	0.00002	2.5	0.00001	2.3	0.00000	1.6	0.00751
LOC100912498	-11.6	0.00255	-17.6	0.00317	-23.0	0.00119	-15.0	0.00360
Ly49s4	2.2	0.02425	3.1	0.00775	3.6	0.00013	2.3	0.03851
Lyz2	1.9	0.00001	-3.3	0.00000	-1.8	0.02964	-1.6	0.00771
Ndst1	2.0	0.02950	-4.2	0.01914	-3.4	0.00000	-2.3	0.02197
Nkg7	2.0	0.00000	2.2	0.00002	2.2	0.00000	1.7	0.00072
Sowahc	2.2	0.00127	-5.3	0.00000	-2.9	0.00013	-2.4	0.00609
Tbx21	1.8	0.00000	2.4	0.00000	2.8	0.00000	1.9	0.00000
Tlr7	1.7	0.00093	-3.6	0.00000	-1.8	0.02458	-1.7	0.00268
Vcan	1.7	0.00548	-9.4	0.00000	-2.3	0.03243	-2.3	0.00062

ΔclpB vs. PBS: Overlappinf genes across time points (147 features)

In red, genes overlapping with LVS vs. PBS across time points

	Day 3		Day 7		Day 10		Day 24	
Name	Fold change	FDR p-value	Fold change	FDR p-value	Fold change	FDR p-value	Fold change	FDR p-value
Abcg2	-2.1	0.01034	-2.4	0.01840	-2.9	0.00004	-2.2	0.00043
Acod1	118.5	0.00000	9.1	0.00000	6.9	0.00000	2.2	0.00544
Adamts12	-3.9	0.03173	-4.4	0.04013	-4.7	0.03414	-3.3	0.04223
Adgra2	-2.1	0.02705	-3.2	0.00436	-4.3	0.00001	-2.3	0.00750
Adgra3	-2.7	0.02778	-3.3	0.01819	-3.0	0.01124	-4.1	0.00039
Adgre1	-2.1	0.00087	-2.6	0.00337	-2.9	0.00001	-2.1	0.00000
Akr1b10	-4.1	0.00000	-3.2	0.00112	-3.3	0.00004	-2.0	0.00453
Alas2	2.6	0.02065	3.5	0.00013	1.9	0.04708	-2.1	0.02088
Arhgef5	-64.6	0.00002	-6.9	0.03108	-6.9	0.04987	-5.9	0.03735
Ascl2	-3.2	0.00028	-2.8	0.01648	-4.8	0.00000	-2.5	0.00315
Asgr2	-15.4	0.00000	-6.9	0.00980	-8.0	0.00356	-5.3	0.00985
Axl	-29.5	0.00000	-7.0	0.01587	-5.6	0.03674	-6.1	0.02373
B3gnt3	-8.6	0.00000	-3.3	0.00014	-2.9	0.00036	-1.8	0.01917
Bpgm	2.2	0.02372	3.3	0.00001	1.7	0.03645	-1.8	0.01167
Ca5b	-2.2	0.01340	-3.2	0.00072	-3.3	0.00006	-2.2	0.00914
Cadm3	-2.9	0.00000	-2.5	0.01898	-3.5	0.00000	-1.9	0.00025
Carmil1	-4.1	0.00000	-2.6	0.04442	-3.8	0.00130	-3.0	0.00041
Ccl2	-12.3	0.00000	-9.2	0.00094	-127.9	0.00000	-20.8	0.00000
Ccl5	-1.7	0.00844	3.0	0.00000	3.5	0.00000	3.1	0.00000
Ccl7	-57.6	0.00000	-14.8	0.00253	-77.2	0.00000	-16.1	0.00088
Ccl9	-2.2	0.00856	-2.6	0.00821	-2.4	0.00229	-1.9	0.00708
Ccnd1	-2.4	0.03400	-4.1	0.00103	-4.4	0.00034	-3.2	0.00601
Cd163	-63.0	0.00000	-9.4	0.04081	-11.3	0.01895	-14.2	0.00273
Cd36	-7.1	0.00000	-4.2	0.00000	-5.0	0.00000	-2.6	0.00000
Cd86	-2.0	0.00108	-2.0	0.00350	-2.3	0.00003	-2.1	0.00001
Cdkn2a	3.3	0.00234	6.7	0.00000	3.2	0.00883	3.1	0.00200
Cebpb	2.2	0.00083	-2.0	0.02684	-2.5	0.00030	-2.2	0.00001
Cfb_1	2.6	0.02702	-3.0	0.02117	-5.2	0.00011	-6.2	0.00000
Cfh	-3.1	0.00001	-2.9	0.02048	-4.8	0.00004	-4.7	0.00000
Cndp1	-23.8	0.00000	-4.7	0.02278	-4.8	0.01312	-4.0	0.01765
Csf1	2.9	0.00000	2.7	0.00000	2.2	0.00000	1.7	0.00014
Ctsl	-1.7	0.01544	-2.0	0.00538	-2.8	0.00000	-2.5	0.00000
Cxcr6	-3.6	0.00009	2.3	0.00865	2.8	0.00002	4.4	0.00000
Cyp26a1	-33.3	0.00000	-6.8	0.02728	-11.2	0.00094	-14.6	0.00008
Dapk1	-2.3	0.00004	-2.6	0.00005	-2.3	0.00002	-1.6	0.00858
Dclk1	3.0	0.00394	11.5	0.00000	9.2	0.00000	10.2	0.00000
Defb14	-4.4	0.01166	-7.5	0.00378	-3.1	0.03273	-3.1	0.02158
Duoxa1	-12.5	0.00121	-22.9	0.00295	-11.1	0.00129	-6.6	0.01049

Dusp5	-2.5	0.00002	-2.5	0.00001	-3.0	0.00000	-1.8	0.00069
Emp1	-4.3	0.00000	1.8	0.03776	2.7	0.00001	3.4	0.00000
Eng	-2.5	0.01569	-2.7	0.02306	-5.3	0.00003	-4.2	0.00007
Erbb3	6.9	0.00000	2.3	0.00293	2.2	0.00106	2.2	0.00002
Fabp4	-6.6	0.00604	-6.1	0.02759	-6.6	0.00369	-4.7	0.01658
Fam198b	-2.6	0.00439	-2.9	0.02238	-3.7	0.00011	-3.1	0.00002
Fam3b	-8.7	0.00145	-6.1	0.02511	-5.3	0.02931	-5.6	0.02425
Fgfr1	-9.5	0.00000	-6.0	0.00010	-5.0	0.00128	-4.5	0.00004
Flnb	-6.9	0.00000	-3.8	0.00000	-3.4	0.00000	-1.8	0.00017
Folr2	-12.0	0.00000	-4.7	0.02470	-8.7	0.00051	-5.0	0.02266
Fscn1	-10.0	0.00000	-5.9	0.00259	-5.5	0.01027	-4.4	0.02465
G0s2	-2.4	0.00119	-2.8	0.00121	-4.9	0.00000	-2.4	0.00001
Gas6	-13.3	0.00000	-4.3	0.00349	-6.6	0.00030	-5.9	0.00011
Gbp5	6.3	0.00000	2.2	0.00002	1.9	0.00000	1.7	0.00001
Gdf3	-3.3	0.00149	-3.6	0.00202	-4.8	0.00001	-2.8	0.00280
Gjb2	-3.5	0.00000	-3.7	0.00000	-3.3	0.00000	-2.0	0.00005
Gjc1	-10.2	0.00038	-4.8	0.03584	-7.0	0.00303	-7.5	0.00154
Glis2	-3.5	0.00151	-2.6	0.03935	-2.7	0.01485	-2.6	0.00860
Gng3	6.1	0.00046	28.1	0.00000	6.0	0.00073	8.4	0.00000
Gpr39	-56.9	0.00039	-14.9	0.00252	-8.4	0.02339	-5.4	0.02764
Gprc5a	-9.0	0.00000	-4.6	0.00443	-7.0	0.00027	-5.2	0.00073
Gprc5d	-6.6	0.00000	-2.4	0.04845	-4.9	0.00003	-4.0	0.00001
Gzmc	326.8	0.00000	250.9	0.00000	31.7	0.02104	122.7	0.00000
Hba-a2_1	2.7	0.02273	4.1	0.00001	1.9	0.02215	-2.1	0.00996
Hba-a2_2	2.5	0.03532	3.6	0.00006	1.8	0.02895	-2.2	0.00784
Hbb	2.5	0.03612	3.7	0.00005	1.9	0.02030	-2.1	0.01520
Heph	-9.4	0.00000	-4.3	0.00686	-4.0	0.00448	-2.7	0.02467
Hes1	-6.2	0.00000	-7.6	0.00000	-6.7	0.00000	-3.1	0.00000
Ifng	6.9	0.00000	4.4	0.00013	4.1	0.00016	7.0	0.00000
Igfbp6	-26.5	0.00002	-4.4	0.02657	-6.9	0.00077	-12.6	0.00002
Il18r1	2.0	0.00515	3.0	0.00000	2.6	0.00000	3.5	0.00000
Il18rap	3.0	0.00000	2.6	0.00000	2.4	0.00000	3.4	0.00000
Klf4	-1.8	0.00567	-2.7	0.00127	-2.7	0.00000	-1.8	0.00056
Lamb3	-4.8	0.00000	-6.7	0.00000	-5.7	0.00000	-1.8	0.03694
Laptm4b	-2.0	0.01192	-2.9	0.00010	-4.3	0.00000	-3.9	0.00000
Ldlrad3	-2.9	0.00004	-3.4	0.00021	-2.2	0.00596	-1.7	0.03801
Lgmn	-1.6	0.00383	-1.9	0.00227	-2.4	0.00000	-1.9	0.00000
Lif	-5.0	0.02264	-9.6	0.00511	-27.5	0.00000	-15.8	0.00008
LOC100910163	-77.3	0.01111	-72.0	0.02812	-23.1	0.00134	-9.8	0.00440
LOC100910801	-2.5	0.02812	-4.3	0.00173	-4.4	0.00046	-2.3	0.02416
LOC100912498	-9.6	0.00663	-24.6	0.00050	-26.9	0.00018	-27.8	0.00008
LOC103694855	2.6	0.03191	3.9	0.00002	1.9	0.02751	-2.2	0.00754
LOC103694857	2.6	0.03113	4.0	0.00001	1.9	0.01812	-2.1	0.01380
Lpar1	-2.1	0.01666	-3.4	0.00180	-2.3	0.01458	-2.6	0.00056

Lpl	-4.0	0.00001	-4.4	0.00023	-3.9	0.00044	-2.3	0.00437
Ltbp1	-7.8	0.00001	-5.0	0.00919	-5.7	0.00853	-4.1	0.03063
Ltbp3	-55.4	0.00000	-7.8	0.04357	-7.9	0.02564	-7.4	0.03208
Ltc4s	-13.3	0.00003	-5.2	0.00953	-7.6	0.00153	-6.7	0.00209
Lyve1	-12.7	0.00000	-6.2	0.00645	-5.5	0.01594	-5.5	0.00603
Mamdc2	-96.7	0.00000	-11.0	0.00384	-8.7	0.01016	-8.7	0.00137
Maob	-4.3	0.00000	-4.3	0.00000	-3.9	0.00000	-1.9	0.00063
Metrnl	-1.5	0.02371	-1.7	0.03531	-2.2	0.00001	-2.0	0.00000
MGC105567	10.4	0.00000	4.1	0.00000	2.2	0.00012	1.5	0.01700
Mras	-1.8	0.00099	-2.1	0.00292	-2.1	0.00003	-1.5	0.00904
Ms4a8	-36.3	0.00000	-7.2	0.00337	-7.6	0.00341	-4.8	0.01577
Mt-nd5	1.6	0.01677	4.5	0.00000	1.8	0.02659	2.3	0.00000
Naaa	-2.0	0.00000	-1.9	0.00140	-2.1	0.00000	-1.9	0.00000
Nkg7	1.6	0.00811	3.4	0.00000	2.7	0.00000	2.3	0.00000
Nr4a1	-2.2	0.00726	-2.4	0.00097	-2.9	0.00000	-1.9	0.00070
Nrxn2	-16.1	0.00000	-8.7	0.00000	-5.8	0.00017	-4.6	0.00005
Olfml2a	-29.0	0.00000	-5.6	0.02836	-8.0	0.01108	-6.0	0.03598
Pdlim4	-14.0	0.00000	-5.9	0.01694	-6.7	0.00389	-4.7	0.02368
Plk2	-6.1	0.00026	-6.4	0.00337	-12.7	0.00000	-8.2	0.00007
Pltp	-5.9	0.00004	-4.7	0.00399	-6.0	0.00270	-5.6	0.00228
Pmp22	-15.3	0.00000	-4.9	0.00009	-5.6	0.00007	-3.9	0.00013
Pon3	-4.0	0.00000	-3.0	0.00183	-2.8	0.00044	-1.7	0.01108
Popdc2	-6.4	0.00000	-2.9	0.00121	-4.0	0.00000	-2.3	0.00085
Pparg	-2.5	0.00153	-4.5	0.00003	-3.6	0.00004	-2.2	0.00150
Prox1	-14.8	0.01024	-13.5	0.02511	-8.6	0.03184	-12.0	0.00983
Psat1	1.8	0.01175	3.4	0.00000	1.9	0.00512	1.5	0.04058
Ptk2	-3.2	0.00002	-2.8	0.00061	-2.3	0.00878	-1.9	0.01444
Ptrf	-20.0	0.00000	-6.2	0.00747	-6.3	0.01019	-4.6	0.04405
Qrfpr	-6.9	0.00000	-5.4	0.00000	-4.2	0.00000	-2.0	0.00336
Rfx2	-3.0	0.00034	-3.1	0.00119	-4.0	0.00000	-2.3	0.00057
RGD1305184	2839.4	0.00000	185.7	0.00271	299.9	0.00064	53.1	0.02007
RGD1310819	-4.7	0.00335	-4.5	0.00662	-4.9	0.00207	-4.1	0.00289
RGD1560281	-2.1	0.00004	-2.5	0.00346	-3.7	0.00000	-1.7	0.00025
RGD1561149	-3.0	0.01273	-2.9	0.03959	-2.6	0.03392	-2.6	0.01423
RGD1561662	-3.9	0.00148	-4.0	0.00884	-6.0	0.00067	-4.6	0.00034
RGD1566368	-6.5	0.00045	-10.2	0.00047	-7.8	0.00039	-3.1	0.02544
Rgl1	-4.6	0.00000	-3.5	0.00003	-2.4	0.00414	-1.8	0.02081
Rhoc	-3.1	0.00000	-1.7	0.02801	-2.2	0.00002	-2.0	0.00001
Rpl35al1	-350.8	0.00103	-34.4	0.00340	-51.5	0.00051	-410.7	0.00123
Sash1	-6.0	0.00042	-4.0	0.03745	-4.6	0.01246	-3.3	0.03705
Scn1b	-8.7	0.00000	-3.6	0.00022	-4.2	0.00000	-3.2	0.00008
Sdk2	-7.3	0.00001	-2.7	0.04621	-3.2	0.00607	-3.8	0.00024
Selenop	-3.6	0.00000	-2.2	0.01424	-2.2	0.00766	-1.8	0.00595
Selp	-13.2	0.00000	-4.3	0.00751	-5.8	0.00213	-4.8	0.00725

Serpinb8	-6.5	0.00000	-2.6	0.01690	-4.1	0.00002	-3.0	0.00004
Sh3bp4	-1.8	0.02352	-2.1	0.01191	-2.5	0.00024	-2.1	0.00030
Shank3	-10.9	0.00014	-7.3	0.00481	-5.5	0.04361	-6.8	0.00182
Slc36a2	-78.2	0.01063	-10.1	0.01218	-13.4	0.00293	-15.4	0.00147
Snta1	-3.6	0.00002	-3.6	0.00002	-3.2	0.00004	-2.0	0.00624
Spink2	112.7	0.01328	976.2	0.00001	654.8	0.00019	698.1	0.00003
T	3.7	0.00002	3.3	0.00112	2.9	0.00139	5.4	0.00000
Tbc1d9	-1.6	0.04541	-2.7	0.00301	-2.7	0.00000	-1.8	0.00073
Tbx21	1.7	0.00075	2.8	0.00000	3.3	0.00000	2.9	0.00000
Tcam1	-16.1	0.00153	-5.8	0.03832	-5.6	0.03507	-6.5	0.01054
Tek	-8.4	0.00000	-3.2	0.01154	-4.2	0.00043	-3.4	0.00049
Tgfa	-7.0	0.00000	-3.1	0.00410	-5.6	0.00000	-1.9	0.01765
Tgfb2	-14.8	0.00000	-5.1	0.00662	-3.9	0.02909	-4.0	0.01640
Thbd	-2.0	0.03669	-3.9	0.00094	-7.8	0.00000	-5.5	0.00000
Timp2	-2.3	0.00024	-2.1	0.02511	-3.9	0.00000	-2.7	0.00000
Tmcc3	-2.6	0.00010	-3.4	0.00020	-2.4	0.00074	-1.7	0.01921
Tppp3	-5.1	0.00000	-2.8	0.00762	-6.6	0.00000	-4.3	0.00002
Vil1	-31.8	0.00000	-6.1	0.03108	-7.3	0.01144	-7.9	0.00276
Wnt4	-4.9	0.03437	-7.3	0.01413	-7.4	0.00979	-13.7	0.00081
Zbtb32	2.5	0.00001	3.5	0.00000	1.7	0.04941	1.7	0.00893
Zcchc24	1.7	0.00302	-2.0	0.02719	-2.0	0.00294	-1.7	0.00086
